# Mapping mosquitoes and their associated pathogens in West Africa

**DOI:** 10.1186/s40249-026-01492-z

**Published:** 2026-08-20

**Authors:** Qing Xu, Chen-Yu Wang, Jing Liu, Run-Ze Ye, Xiao-Yang Wang, Bao-Yu Wang, Shan-Shan Wang, Lin Zhao, Wu-Chun Cao

**Affiliations:** 1 Institute of EcoHealth, School of Public Health, Cheeloo College of Medicine, Shandong University, Jinan, Shandong China; 2 Shandong Academy of Preventive Medicine, Shandong Center for Disease Control and Prevention, Jinan, Shandong China; 3 Department of Emergency Medicine, Qilu Hospital of Shandong University, Jinan, Shandong China; 4 Shandong Provincial Key Laboratory of Intelligent Monitoring, Early Warning, Prevention and Control for Infectious Diseases, Jinan, Shandong China; 5 State Key Laboratory of Pathogen and Biosecurity, Beijing Institute of Microbiology and Epidemiology, Beijing, China

**Keywords:** Mosquito, West Africa, Mosquito-associated pathogen, Mosquito-borne disease

## Abstract

**Background:**

Mosquitoes transmit diverse pathogens, some of which cause mosquito-borne diseases (MBDs). Climate and socioeconomic changes, such as temperature fluctuations, altered rainfall patterns, and urbanization, may affect the distributions of key vectors, particularly *Aedes* and *Anopheles*, and increase the health burden of MBDs. West Africa is a historically high-endemic region for MBDs, including malaria and dengue. However, existing studies in this region remain limited in scope, making comprehensive, high-resolution spatial mapping of vectors and pathogens inadequate. This study aimed to map the distributions of mosquitoes and mosquito-associated pathogens and identify potentially suitable areas for key mosquito species in West Africa, thereby supporting regional surveillance and control efforts.

**Methods:**

We conducted a scoping review of English-language studies in PubMed, Web of Science, Embase, and Scopus through 7 March 2023. From eligible studies, relevant online databases, and GenBank, we extracted records of mosquito species, mosquito-associated pathogens, and human infections documented by outbreaks, pathogen isolation, molecular detection, or serology. Geographic records were standardized to WGS84 and mapped in ArcGIS 10.7. Potential distributions of key mosquito species were modeled in Maxent 3.4.1 using 24 climatic, topographic, population, land-cover, and related variables. Model performance was evaluated using the area under the receiver operating characteristic curve (AUC).

**Results:**

We compiled 19,578 occurrence records for 302 mosquito species in 16 genera and 598 records for 124 mosquito-associated pathogens from 22 families; 20 pathogens were reported to infect humans. *Aedes*, *Anopheles* and *Culex* occurred in all 16 countries, and *Anopheles* harbored the most pathogen species (*n* = 55). DENV was detected in mosquitoes in five countries but human infections were reported in 13. Models for nine key mosquito species yielded AUC values of 0.763–0.912. Land cover contributed more than 10% to six models, and suitable habitats were predicted in Cape Verde for five species without occurrence records in the country.

**Conclusions:**

We established a geospatial database of mosquitoes and their associated pathogens in West Africa. Current records remain incomplete and unevenly distributed, with gaps in key vector occurrence and limited mosquito-based detection of viral and parasitic pathogens relative to human infection evidence. Ecological niche modeling suggests that suitable habitats of some important mosquito species may extend beyond their reported ranges. These findings can guide targeted field surveys and coordinated mosquito–pathogen surveillance in under-sampled areas of West Africa.

**Supplementary Information:**

The online version contains supplementary material available at 10.1186/s40249-026-01492-z.

## Background

Mosquitoes (Diptera: Culicidae) are among the most important arthropod vectors of pathogens affecting humans and animals [1]. Mosquito-borne diseases (MBDs), including malaria, dengue, Zika, chikungunya, and yellow fever, continue to cause substantial morbidity and mortality worldwide [2]. Climate and socioeconomic changes, such as rising temperatures, altered rainfall patterns, rapid urbanization, population growth, and increased international travel and trade, are reshaping the distribution of mosquito vectors and the pathogens they carry [3]. For example, *Anopheles stephensi* has expanded beyond its native range into several African countries, with confirmed occurrences in Djibouti [4, 5], Sudan, Somalia, Nigeria [6], and Ghana [7], raising concern about malaria transmission in newly affected areas [6]. *Aedes aegypti*, a major arbovirus vector with African origins [8], has also expanded in many regions over the past decade [9, 10]. Its continued spread is expected to widen the range of populations susceptible to arbovirus transmission in the coming decades [11]. These changes highlight the need to better understand the geographic distributions of mosquitoes, their associated pathogens, and the ecological and socioeconomic factors that shape these patterns.

West Africa has long been recognized as a hotspot for MBDs, with repeated reports of malaria [12], dengue [13], Zika [14], yellow fever [15], and other mosquito-borne infections [16]. Previous studies have summarized mosquito-associated viruses and their related mosquitoes in West Africa [3], and Tandina et al. reviewed medically important mosquitoes and mosquito-borne diseases in Mali [17]. These studies have provided important baseline information, but they have generally focused on selected pathogens, specific mosquito groups, or individual countries [3]. As a result, regional-scale evidence on mosquito species, pathogens detected in mosquitoes, and human infection evidence remains fragmented [16, 18]. In particular, comprehensive high-resolution spatial mapping of mosquito species and their associated pathogens across West Africa is still inadequate [18], and the potential distributions of key vector species and their environmental predictors remain insufficiently characterized. For example, the study in Enugu State, Nigeria, modeled *Aedes* spp. using 18 occurrence points and climatic and topographic variables, without incorporating land cover or population density [19].

Here, we conducted a scoping review with geospatial mapping and ecological niche modeling to assess the distributions of mosquito species and their associated pathogens in West Africa. We compiled records of mosquitoes, pathogens detected in mosquitoes, and national-level evidence of human infection with mosquito-borne pathogens; mapped their spatial distributions; estimated pathogen positivity rates where data were available; and predicted suitable habitats for key mosquito species. This work aims to identify current evidence gaps and provide a spatial basis for future mosquito and pathogen surveillance in West Africa.

## Methods

### Data collection

We conducted a scoping review combined with geospatial mapping and ecological niche modeling to summarize the distributions of mosquito species, mosquito-associated pathogens, and evidence of human infection with mosquito-borne pathogens in West Africa. Data were collected from multiple sources, including published literature, public databases, and GenBank.

For the literature search, we systematically retrieved English-language articles from four electronic databases: PubMed, Web of Science, Embase, and Scopus. The search covered the period from database inception to March 7, 2023. The search strategy combined mosquito-related terms, including “mosquito,” “mosquitoes,” “mosquitos,” and “Culicidae,” with geographic terms referring to West Africa and individual West African countries and territories. The full search strategy is provided in Supplementary Table 2.

After duplicate removal, eligibility was assessed independently by two reviewers. Studies were included if they reported mosquito field surveys or detection of mosquito-associated pathogens conducted in West Africa. Studies were excluded if they were unrelated to the study topic, duplicated, or had unreliable or inaccurate mosquito species identification. Extracted variables included mosquito taxonomy, pathogen name and type, sampling year, sampling location, geographic coordinates, administrative unit, number of mosquitoes tested and positive, detection method, positivity rate, and evidence of human infection. Detailed procedures for literature screening and data extraction are described in the appendix (Supplementary Fig. 33 and Text 3).

In addition to the literature review, we extracted mosquito occurrence records from the Global Biodiversity Information Facility (GBIF) [20], the Malaria Threat Map [21], and VectorMap [22]. Records of mosquito-associated pathogens in West Africa were also obtained from GenBank [23]. All records from the literature and public sources were consolidated into a unified database for subsequent analyses.

Evidence of mosquito-borne pathogen infections in humans across West African countries was collected from EM-DAT [24], ProMED [25], WHO publications, and related literature. Evidence was included if it met at least one of the following criteria: (1) outbreak or epidemic caused by the pathogen; (2) pathogen isolation from human samples, or molecular detection with sequence confirmation; or (3) serological evidence of pathogen infection in humans.

Environmental variables potentially associated with mosquito ecology were collected for ecological niche modeling. Nineteen bioclimatic variables (Bio1–Bio19), representing temperature and precipitation conditions, and elevation data were obtained from WorldClim version 2.1 [26]. Slope and aspect were derived from elevation data using the Spatial Analyst Tool in ArcGIS (version 10.7; ESRI, Redlands, CA, USA). A 2010 global population density grid was obtained from LandScan [27], and land cover data were downloaded from Global Map [28] (Supplementary Tables 3 and 4). In total, 24 environmental variables were resampled to 2.5 arc-minute resolution, approximately 5 × 5 km near the equator, and cropped to the West African study area using ArcGIS (version 10.7; ESRI, Redlands, CA, USA).

### Mapping of mosquitoes and associated pathogens

Thematic maps showing the spatial distributions of mosquitoes and their associated pathogens were produced using ArcGIS (version 10.7; ESRI, Redlands, CA, USA). Geographic coordinates were obtained from the included literature and public databases, including GBIF, Malaria Threat Map, VectorMap, and GenBank. Human infection records were compiled from EM-DAT, ProMED, WHO publications, and related literature. Administrative boundary data for West Africa were obtained from the GADM database, version 3.6 [29], and were used for study-area definition and spatial validation.

All geographic records were standardized to the WGS84 geographic coordinate system (EPSG: 4326). No additional map projection was applied. Coordinates were checked for invalid values, reversed latitude and longitude, duplicate records, and locations falling outside the reported country or the West African study area. Records with vague locality descriptions that could not be reliably georeferenced were excluded from point-level mapping. For records with specific locality names but without coordinates, locations were georeferenced using Google Earth according to the reported locality and administrative unit.

For mosquito species, records with exact or reliably georeferenced latitude and longitude coordinates were used to map species distributions. For mosquito-associated pathogens, exact collection locations were used when available. When only administrative-level locations were reported, the centroids of the corresponding administrative units were used for mapping. Environmental raster layers used for ecological niche modeling were standardized to 2.5 arc-minute spatial resolution.

### Meta-analysis of mosquito-associated pathogens

The meta-analysis included studies that reported the number of mosquitoes tested and positive, or percentages from which raw numbers could be calculated. Detection methods included PCR, virus isolation in cell culture, enzyme-linked immunosorbent assay (ELISA), and microscopy. Positivity rates were expressed as percentages.

For each study, pathogen positivity rates were calculated for mosquito genera by summing the numbers of tested and positive mosquitoes within each genus. When a pathogen species was reported by only one study, the positivity rate was calculated by dividing the number of positive mosquitoes by the total number of tested mosquitoes, and the 95% confidence interval (*CI*) was not calculated. When a pathogen was reported in multiple studies, meta-analysis was conducted using R version 4.2.3 (R Core Team, R Foundation for Statistical Computing, Vienna, Austria) with the meta package to estimate the pooled positivity rate and 95% *CI*. Heterogeneity was assessed using the *I*^2^ statistic. A random-effects model was used when *I*^2^ was greater than 50%; otherwise, a fixed-effect model was applied.

### Model establishment and evaluation

To assess potentially suitable areas beyond currently reported mosquito distributions, nine key mosquito species were selected for ecological niche modeling: *Aedes aegypti*, *Aedes luteocephalus*, *Aedes vittatus*, *Anopheles funestus* s.l., *Anopheles gambiae* s.l., *Culex quinquefasciatus*, *Culex nebulosus*, *Mansonia africana*, and *Mansonia uniformis*. These species were selected because they are widely recorded and/or medically important in West Africa, are associated with pathogens of public health relevance, and have sufficiently precise occurrence records for modeling.

Maxent software (version 3.4.1; American Museum of Natural History, New York, USA) was used to model the potential distributions of these species based on presence-only occurrence records. Predictive models were based exclusively on records with precise geographic coordinates. To reduce spatial autocorrelation, duplicate occurrence records within the same environmental grid cell were removed using ENMTools 1.4.4 [30].

Pre-modeling was conducted in Maxent software (version 3.4.1; American Museum of Natural History, New York, USA) using the 24 environmental variables for each mosquito species. During the preliminary modeling step, 75% of occurrence records were used for training and 25% for testing. The jackknife test was used to assess the relative contribution of environmental variables. To reduce multicollinearity, pairwise correlations among the 24 variables were assessed using ENMTools. When two variables were highly correlated, defined as |r|> 0.90, the variable with the lower contribution in the preliminary model was removed. Variables contributing less than 1% were also excluded. Therefore, each mosquito species had a species-specific final set of environmental variables.

The R package kuenm in R version 4.2.3 (R Core Team, R Foundation for Statistical Computing, Vienna, Austria) was used to select the best feature classes (FCs) and regularization multiplier (RM) based on the lowest corrected Akaike Information Criterion (AICc) [31–33]. Final models were implemented in Maxent software (version 3.4.1; American Museum of Natural History, New York, USA) using the selected parameters and species-specific variable sets. Models were replicated 10 times using tenfold cross-validation, and the maximum number of iterations was set to 10,000. Model performance was evaluated using the area under the receiver operating characteristic curve (AUC), which ranges from 0 to 1 [34].

## Results

### Characteristics of mosquito database

**Fig. 1:**
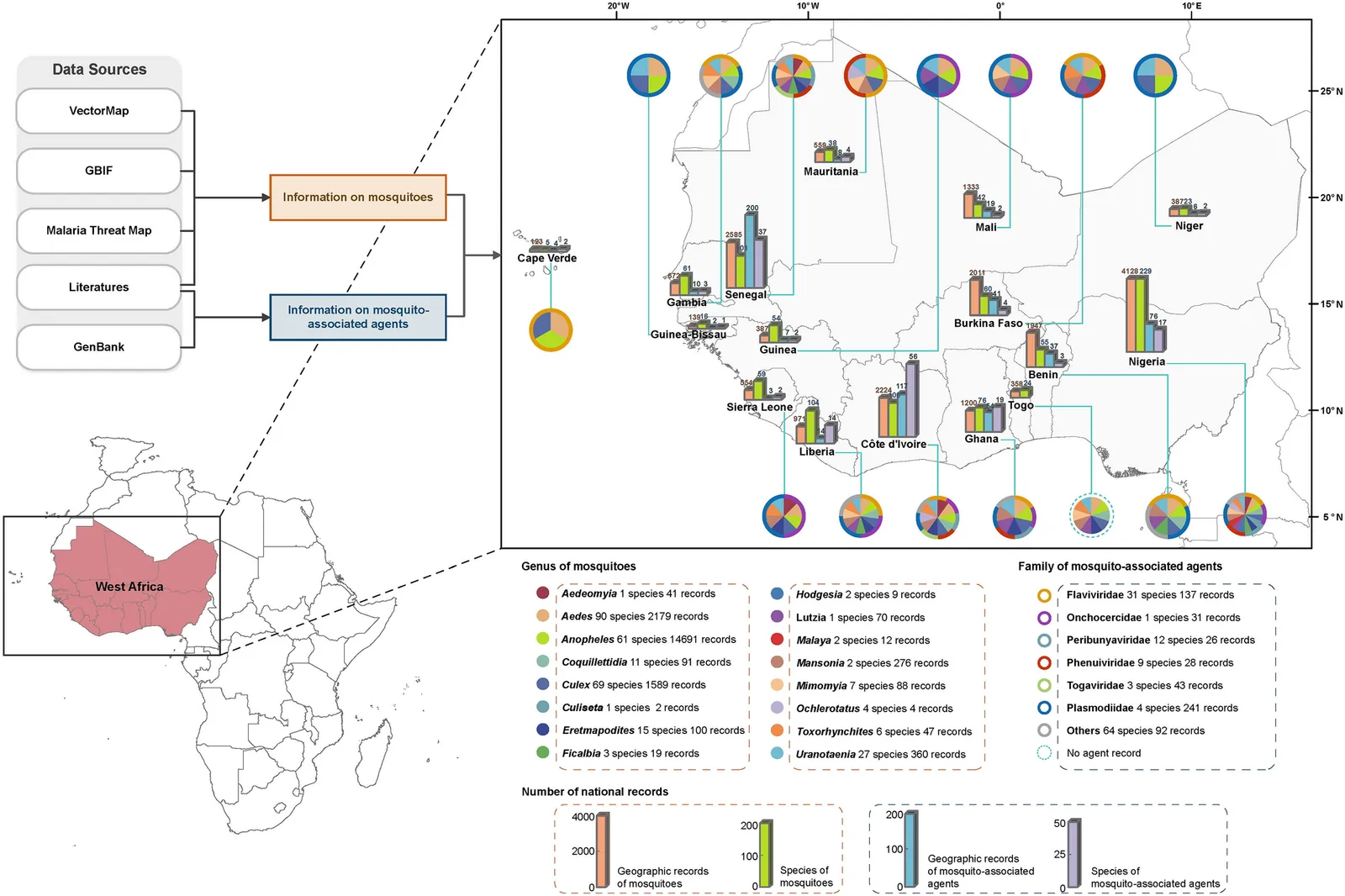
Overview of mosquito database. The database of mosquitoes was collected and integrated from multiple data sources, including literature review, related websites, and GenBank, and a comprehensive analysis was conducted. The pies indicate the genus composition of mosquitoes, with the color of circle outlines representing the family composition of mosquito-associated pathogens. The bars represent the number of national records: orange, geographic records of mosquitoes; blue, species of mosquitoes; green, geographic records of mosquito-associated pathogens; purple, species of mosquito-associated pathogens

After removing duplication, we ultimately compiled a database containing 19,578 unique records on geographic distributions of 302 known mosquito species across 16 mosquito genera and 598 unique records on their associated pathogens of 124 pathogens belonging to 22 families (Fig. 1 and Supplementary Text 1). The most widely distributed mosquito genera were *Aedes*, *Anopheles*, and *Culex*, which had 2179, 14,691 and 1589 occurrence records respectively. These three genera were found in all countries in West Africa (Fig. 1, Supplementary Figs. 1–7 and Supplementary Table 1). Nigeria had the highest number of geographical records and the greatest variety of mosquito species, with 4128 geographical records and 229 mosquito species in 16 genera, while records from Cape Verde were comparatively limited, with 123 geographical records and five species in three genera (Fig. 1). At the species level, *An. gambiae* sensu lato (s.l.) and *Ae. aegypti* were widely distributed across all countries. *An. squamosus*, *An. funestus* s.l*.*, *An. coustani* s.l., and *Cx. quinquefasciatus were* reported in 15 countries (Supplementary Figs. 1–7 and Supplementary Table 1).

A total of 124 pathogens belonging to 22 families were found in mosquitoes. Of the 22 pathogen families, six with a relatively substantial number of records were depicted in Fig. 1, including *Flaviviridae* (reported in 10 countries), *Onchocercidae* (7), *Peribunyaviridae* (4), *Phenuiviridae* (6), *Togaviridae* (2), *Plasmodiidae* (13). Senegal exhibited the highest number of pathogen records (200 records), and Côte d'Ivoire had the greatest diversity of pathogens (56 species). Togo lacked documented records of pathogen detection, and a relatively limited diversity of mosquito species was observed, encompassing 358 geographical records for 24 mosquito species (Fig. 1).

### Diversity and positivity rate of pathogens

**Fig. 2:**
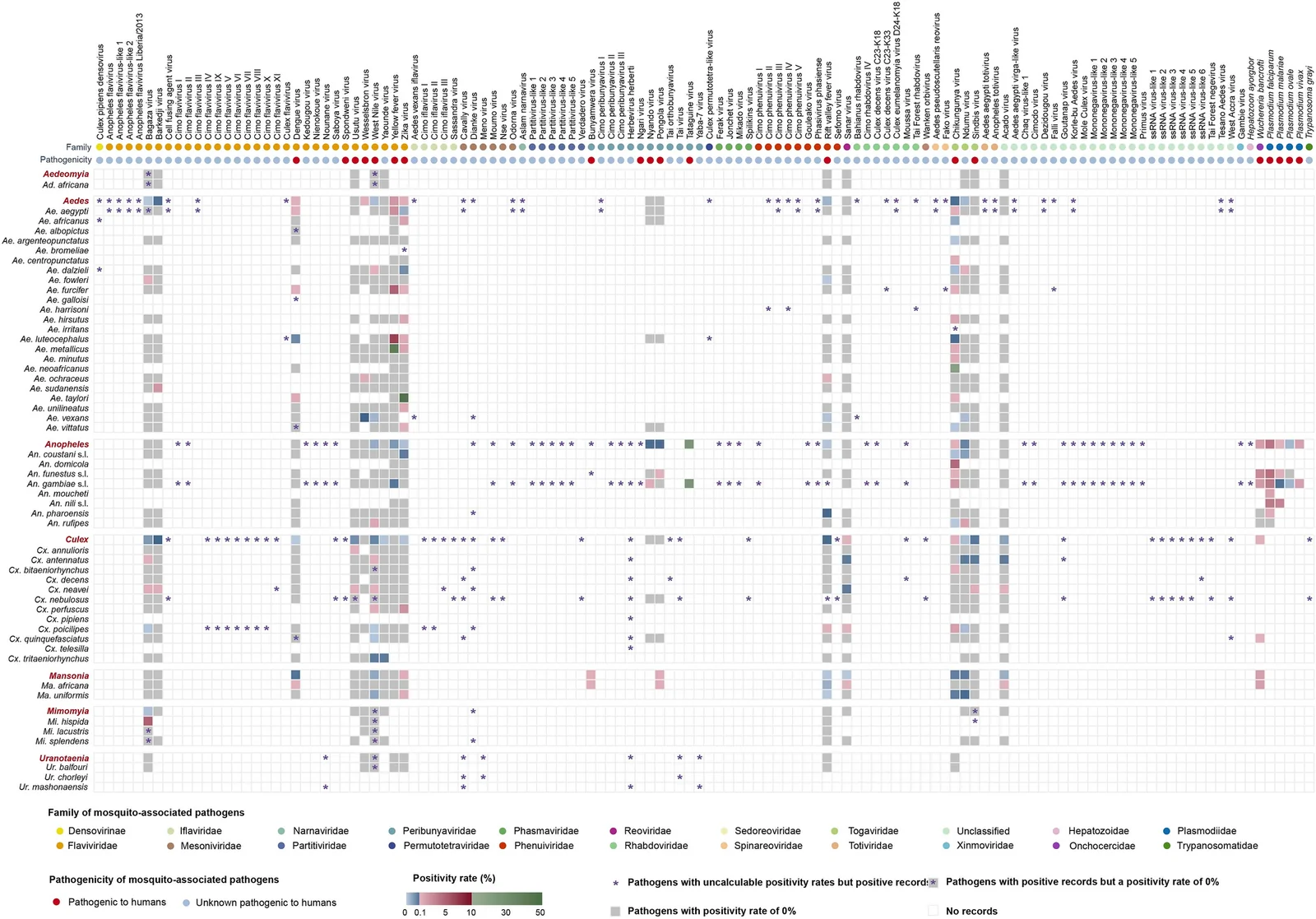
Prevalence of mosquito-associated pathogens in West Africa. The top row of circles represents the family of mosquito-associated pathogens, and the bottom row of circles represents the pathogenicity of mosquito-associated pathogens, including definite pathogenicity to humans (Red), and unknown pathogenicity to humans (Blue). The columns from left to right are positivity rates and positive records of mosquito-associated pathogens

Among the 124 pathogens identified, 117 were viruses and seven were parasites. Twenty pathogens have been reported to infect humans, including DENV, Spondweni virus, Usutu virus, Wesselsbron virus, West Nile virus (WNV), Yellow fever virus (YFV), and ZIKV in *Flaviviridae*, Bunyamwera virus, Ngari virus, Nyando virus, Pongola virus, and Tataguine virus in *Peribunyaviridae,* Rift Valley fever virus (RVFV) in *Phenuiviridae*, CHIKV and Sindbis virus in *Togaviridae*, *Wuchereria bancrofti* in *Onchocercidae*, and four *Plasmodium* species in *Plasmodiidae* (Fig. 2).

As depicted in Fig. 2, one species of *Aedeomyia*, twenty-three species of *Aedes*, eight species of *Anopheles*, twelve species of *Culex*, two species of *Mansonia*, three species of *Mimomyia*, and three species of *Uranotaenia* were identified as potential vectors capable of carrying mosquito-associated pathogens. *Anopheles* was the genus harboring the highest number of mosquito-associated pathogens (55 species), followed by *Culex* (51), *Aedes* (44), and *Mansonia* (11). *An. gambiae* s.l. was the species carrying the highest diversity of pathogens, with as many as 49 species. *Ae. aegypti* acted as a vector for 27 known species, which consisted of four major human pathogens including CHIKV, DENV, YFV, and ZIKV. Other competent mosquito species that harbored ten or more pathogens are *Cx. nebulosus* (25 pathogens), *Cx. poicilipes* (16), and *Cx. neavei* (10) (Fig. 2). It is worth noting that *Ma. uniformis* could carry WNV, ZIKV, RVFV, and CHIKV (Fig. 2).

In *Flaviviridae*, DENV was reported primarily and had the highest positivity rate in *Aedes* (0.231%, 95% *CI* 0.000–1.117), followed by *Mansonia* (0.084%, 95% *CI* 0.054–0.114) and *Culex* (0.001%, 95% *CI* 0.000–0.003). DENV was predominantly identified in *Ae. aegypti*, exhibiting a positivity rate of 0.245% (95% *CI* 0.000–1.531). WNV was mainly associated with *Culex* (0.067%, 95% *CI* 0.000–0.190), including *Cx. antennatus* (0.235%, 95% *CI* 0.000–0.494), *Cx. neavei* (0.134%, 95% *CI* 0.000–1.017), and *Cx. poicilipes* (0.001%, 95% *CI* 0.000–0.007). YFV and ZIKV could be detected from *Aedes* (1.683%, 95% *CI* 0.292–3.075; 0.310%, 95% *CI* 0.055–0.564, respectively). YFV was primarily reported in *Ae. aegypti* (2.057%, 95% *CI* 0.679–3.435) and *Ae. furcifer* (4.560%, 95% *CI* 3.098–6.020), as the number of studies recording positive results was five and three. ZIKV was predominantly detected in *Ae. aegypti* (0.024%, 95% *CI* 0.000–0.121) and *Ae. luteocephalus* (0.554%, 95% *CI* 0.276–0.833), as the number of studies reporting positive results in both mosquito species was three. As the exclusive pathogen in *Phenuiviridae* capable of causing human disease, RVFV was often detected from *Ae. ochraceus*, *An. gambiae* s.l., and *Cx. poicilipes*, and had the highest positivity rate among *Culex* (0.091%, 95% *CI* 0.005–0.178). CHIKV, which belongs to *Togaviridae*, could be carried by 22 species of mosquitoes and was primarily associated with *Aedes* (0.089%, 95% *CI* 0.000–0.177), while having the highest positivity rate in *Anopheles* (0.108%, 95% *CI* 0.000–0.285). The positivity rate of *Wuchereria bancrofti* was highest among *Anopheles* (1.322%, 95% *CI* 0.374–2.837), followed by *Mansonia* (1.198%) and *Culex* (0.173%, 95% *CI* 0.000–0.490). *Plasmodiidae*, including *P. falciparum* (3.530%, 95% *CI* 2.621–4.568), *P. malariae* (0.206%, 95% *CI* 0.014–0.621), *P. ovale* (0.017%, 95% *CI* 0.000–0.035), and *P. vivax* (1.075%), was only detected in *Anopheles* according to the present dataset of our study (Fig. 2, Supplementary Figs. 8–13).

### Distribution of pathogens and human infections

**Fig. 3:**
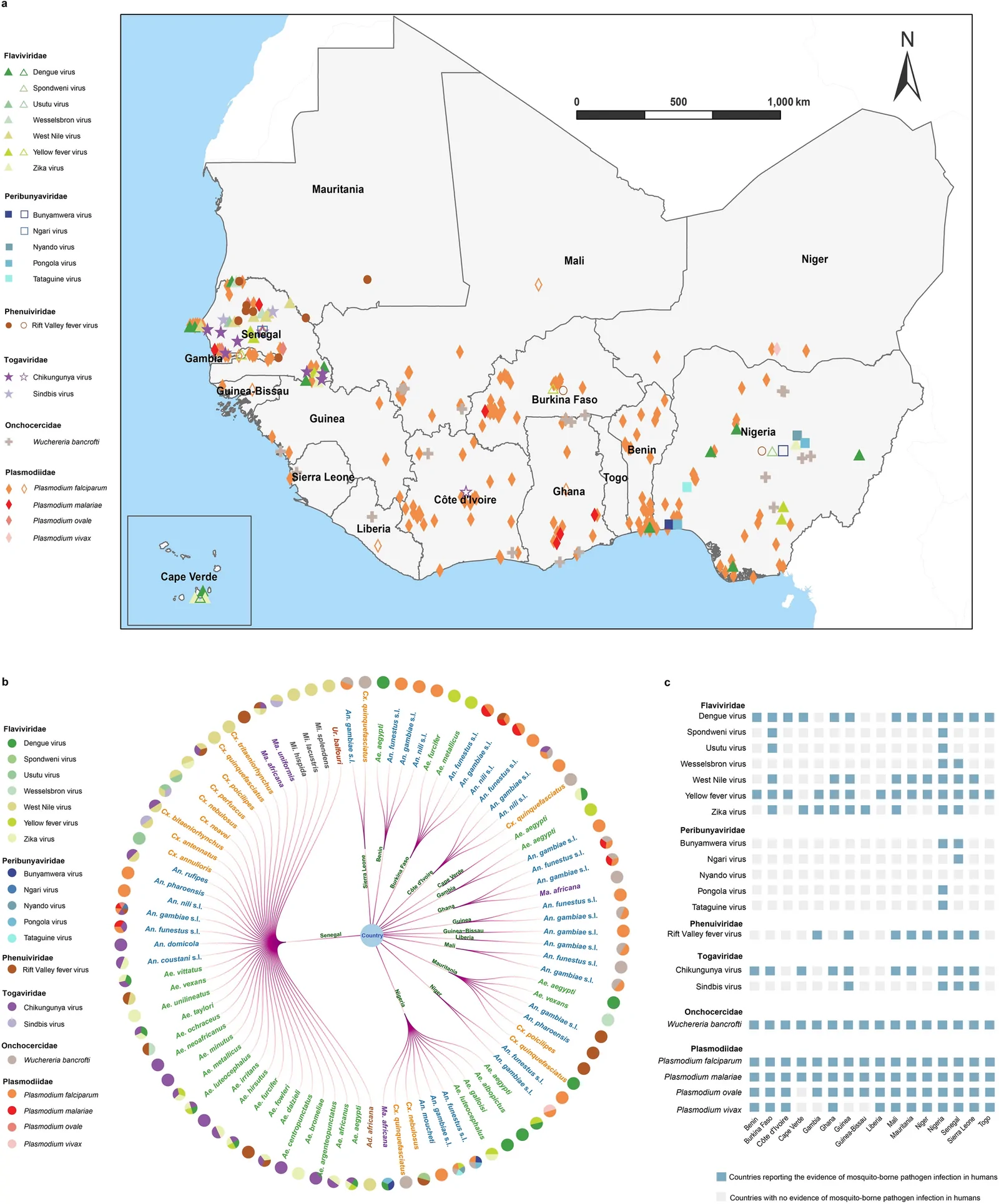
Distribution and human infection evidence of mosquito-borne pathogens in West Africa. **a**, Geographical distribution of pathogens detected from mosquitoes. Pathogens with precise geographical coordinates are depicted using solid graphics, while those with only country-level information are indicated with hollow graphics at the center of the country. **b**, Pathogens carried by mosquito species in West African countries. The inner circle represents the various West African countries. The outer circle shows the mosquito species that can carry disease-causing pathogens, which are colored according to mosquito genera. Small circles outside represent the pathogens detected from the country's corresponding mosquito species. **c**, Evidence of mosquito-borne pathogen infection in humans at the national level. The blue grids indicate that the country has reported evidence of mosquito-borne pathogen infection in humans. In contrast, gray grids indicate a lack of evidence of human mosquito-borne pathogen infection in the country

We mapped the geographical distribution of pathogens and the occurrence of pathogen infections in humans in West Africa (Fig. 3, Supplementary Text 2). Overall, various disease-causing viruses were mainly reported in Senegal (10 species) and Nigeria (9), and *Plasmodium* was widely distributed throughout West Africa (Fig. 3a, b). In Senegal, there were 42 mosquito species (mainly *Aedes*) with the potential to serve as vectors for pathogens affecting humans. *Ae. aegypti* was the exclusive mosquito species capable of harboring pathogens in Cape Verde. The predominant mosquito genus carrying pathogens was *Anopheles* (mainly *An. funestus* s.l. and *An. gambiae* s.l.) in the other 13 West African countries (Fig. 3b). Nigeria had the highest diversity of documented pathogens with evidence of human infections, followed by Senegal (Fig. 3c).

DENV was the most widely distributed pathogen detected from mosquitoes, covering Benin (detected from *Ae. aegypti*), Cape Verde (*Ae. aegypti*), Mauritania (*Ae. aegypti* and *Cx. quinquefasciatus*), Nigeria (*Ae. aegypti*, *Ae. albopictus*, *Ae. galloisi*, and *Ma. africana*), and Senegal (*Ae. aegypti*, *Ae. furcifer*, *Ae. luteocephalus*, *Ae. taylori*, and *Ae. vittatus*). Human cases infected with DENV were reported in the majority of West African countries, except for Gambia, Guinea-Bissau, and Liberia. YFV was only detected from mosquitoes in four countries, including Burkina Faso (detected from *Ae. furcifer* and *Ae. metallicus*), Gambia (*Ae. aegypti*), Nigeria (*Ae. aegypti*, *Ae. luteocephalus* and *An. gambiae* s.l.), and Senegal (*Ae. aegypti*, *Ae. furcifer*, *Ae. luteocephalus*, and *Ae. metallicus*). However, most countries had clear evidence of YFV infection in humans, with the exception of Guinea-Bissau and Cape Verde. We found evidence of ZIKV infection in mosquitoes (mainly *Aedes*) from Cape Verde, Nigeria, and Senegal. RVFV has been identified in mosquitoes in the following countries: Burkina Faso (detected from *An. gambiae* s.l.), Mauritania (*An. gambiae* s.l., *An. pharoensis*, and *Cx. poicilipes*), Nigeria (*Cx. nebulosus*), and Senegal (*Ae. fowleri*, *Ae. ochraceus*, *Ae. vexans*, *An. gambiae* s.l., *Cx. poicilipes*, *Ma. africana*, and *Ma. uniformis*). Evidence of infection in humans was not documented in Burkina Faso (Fig. 3b, c, and Supplementary Text 2). We have not uncovered any evidence substantiating the infection of humans by Nyando virus in West Africa (Fig. 3c and Supplementary Text 2).

### Ecological modeling for key mosquitoes

We performed ecological modeling for each of the nine key mosquito species. The ecological niche modeling (ENM) results demonstrated high accuracy, with average testing Area Under the Curve (AUC) values ranging from 0.763 to 0.912, indicating decent predictive power (Fig. 4a–i). The predictive factors influencing the geographic distribution of mosquitoes varied across species, even within the same genus (Fig. 4 and Supplementary Figs. 14–31).

**Fig. 4:**
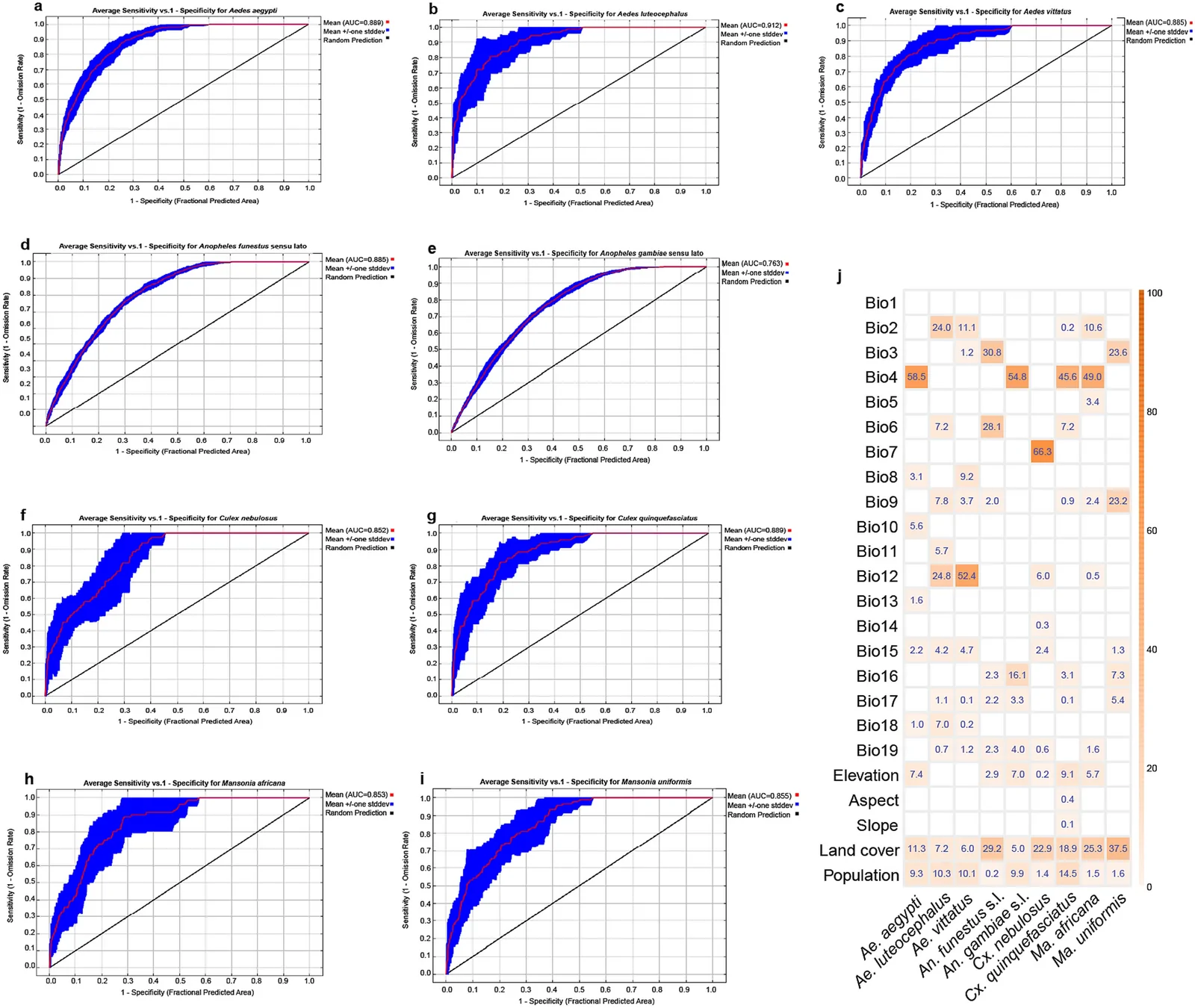
The average testing areas under the curve and relative contributions of the environmental variables. Receiver operating characteristic (ROC) curve’s area under the curve (AUC) of the Maxent model for **a**, *Aedes aegypti*. **b**, *Aedes luteocephalus*. **c**, *Aedes vittatus*. **d**, *Anopheles funestus* sensu lato. **e**, *Anopheles gambiae* sensu lato. **f**, *Culex nebulosus*. **g**, *Culex quinquefasciatus*. **h**, *Mansonia africana*. **i**, *Mansonia uniformis*. **j**, Relative contributions of the environmental variables to the Maxent model. The colors and numbers of each grid in the heat map represent the value of contribution percentage

Land cover was an important driver included in the variable set for all mosquitoes, as it contributed more than 10.00% to the ensemble models of six mosquito species (Fig. 4j). Compared with other land cover types, mosquitoes preferred urban construction land for survival (Supplementary Figs. 14–31). Population density could also influence the distribution of all mosquito species with relatively lower contributions (Fig. 4j). The MaxEnt response curves generally showed a positive association between the population variable and predicted habitat suitability, although the shape and rate of increase varied among species. For several species, predicted habitat suitability increased rapidly at lower population values and then approached a plateau at higher values (Supplementary Figs. 14–31). For *Ae. aegypti*, *An. gambiae* s.l., *Cx. quinquefasciatus*, and *Ma. africana*, temperature seasonality (Bio4) had the highest percentage contribution to the Maxent models, contributing 58.50, 54.80, 45.60, and 49.00%, respectively (Fig. 4j). These percentages represent the relative contribution of Bio4 to each species-specific model prediction. Habitat suitability was predicted to have a peak and then decrease with increasing temperature seasonality (Supplementary Figs. 15, 23, 27, and  29). Annual precipitation (Bio12) had the highest percentage contribution to the Maxent models for *Ae. luteocephalus* (24.80%) and *Ae. vittatus* (52.40%) among the environmental variables included in their respective models (Fig. 4j).

**Fig. 5:**
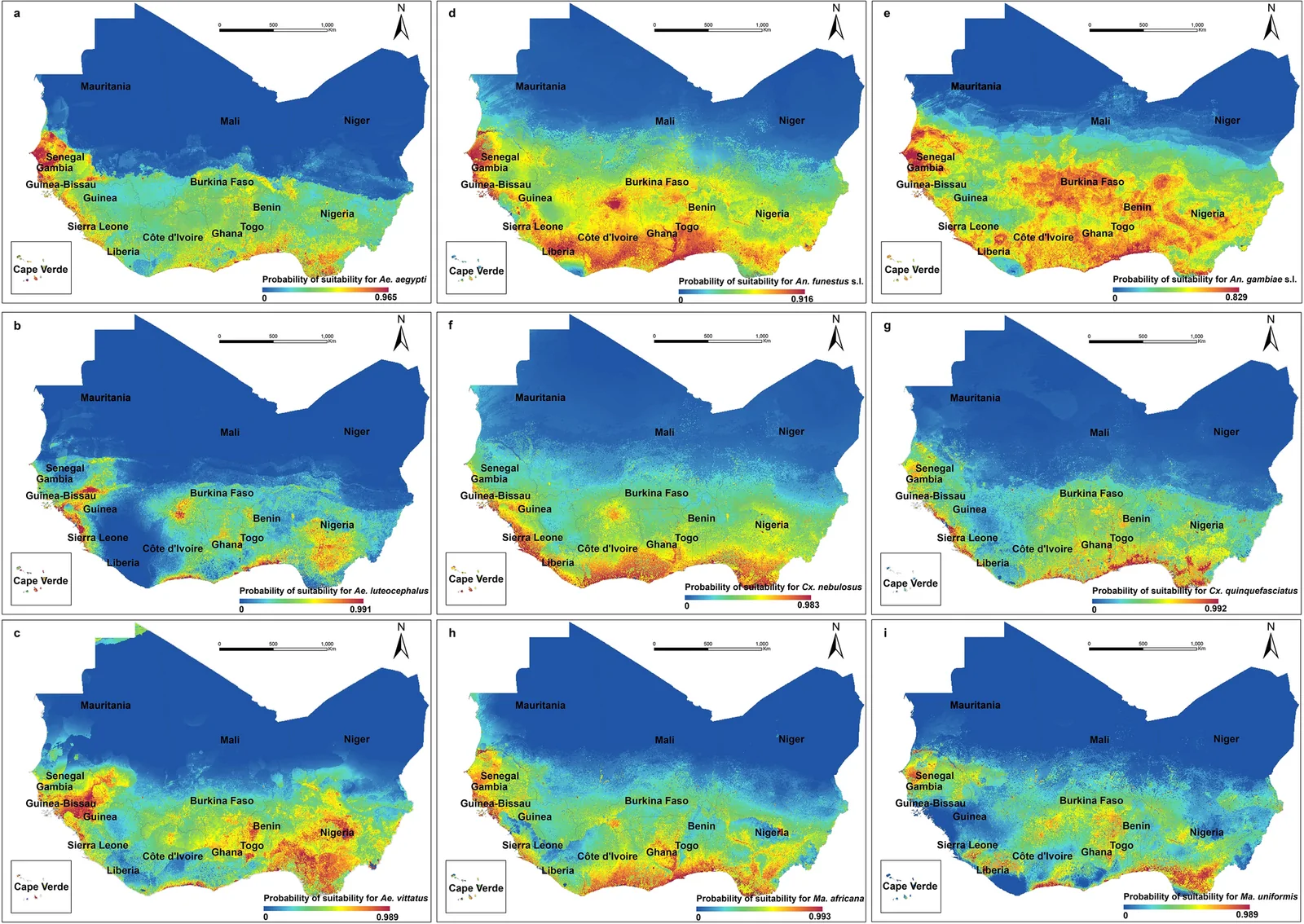
Potential distribution map of nine key mosquitoes in West Africa. **a**, *Aedes aegypti*. **b**, *Aedes luteocephalus*. **c**, *Aedes vittatus*. **d**, *Anopheles funestus* sensu lato. **e**, *Anopheles gambiae* sensu lato. **f**, *Culex nebulosus*. **g**, *Culex quinquefasciatus*. **h**, *Mansonia africana*. **i**, *Mansonia uniformis.* The predicted probabilities of suitability for mosquitoes were shown in continuous outputs from 0 to 1 by Maxent

Areas with high predicted habitat suitability for *Ae. aegypti* widely covered southern West Africa, with the central and northwestern parts of Senegal showing particularly high suitability (Fig. 5a). Areas with high suitability for *Ae. luteocephalus* and *Ae. vittatus* were mainly located in central Nigeria and northwestern Guinea (Fig. 5b, c). Significantly, *Ae. vittatus* exhibited a broader suitable zone despite having fewer distribution points (Fig. 5c, Supplementary Fig. 32). *An. funestus* s.l. and *An. gambiae* s.l. exhibited broad distribution across numerous regions in West Africa, encompassing both observed distribution points and predicted ranges (Fig. 5d, e and Supplementary Fig. 32). However, in contrast to other mosquito species, these two species (*An. funestus* s.l. and *An. gambiae* s.l.) are notably concentrated in central West African countries, including Burkina Faso, Ghana, and Côte d'Ivoire (Fig. 5d, e). For *Culex*, the high-risk areas are primarily situated along the southern and western coastal regions of West Africa (Fig. 5f, g). The most suitable habitats for *Ma. africana* and *Ma. uniformis* were in southern Nigeria, southern Benin, southern Côte d'Ivoire, western Senegal, and Ghana. Guinea-Bissau and Sierra Leone had predicted suitability for these two mosquitoes (Fig. 5h, i). Suitable habitats were also predicted in Cape Verde for five mosquito species, including *Ae. luteocephalus*, *Ae. vittatus*, *An. funestus* s.l., *Cx. nebulosus*, and *Ma. africana*, although no occurrence records for these species were available in our dataset (Fig. 5, Supplementary Fig. 32).

## Discussion

In this study, we integrated evidence on mosquito occurrence, mosquito-associated pathogens, and human infection records to characterize the mosquito–pathogen landscape in West Africa. Rather than simply showing where mosquitoes or pathogens have been reported, our results highlight several linked patterns: mosquito and pathogen records are unevenly distributed across West Africa, medically important genera are widely present, mosquito-based pathogen detection remains sparse compared with human infection evidence, and several key mosquito species may have suitable habitats beyond their currently reported ranges. Together, these findings suggest that the apparent distribution of mosquitoes and their associated pathogens in West Africa reflects both ecological suitability and uneven surveillance effort.

The wide distribution of *Anopheles*, *Aedes* and *Culex* is consistent with their ecological adaptability and medical importance in tropical and subtropical settings [35–37]. In particular, *Anopheles* was associated with the greatest diversity of detected pathogens in our dataset. This pattern is not unexpected, given the abundance, anthropophilic behavior, and high vectorial capacity of major African malaria vectors such as *Anopheles gambiae* s.l. and *Anopheles funestus* s.l [35, 38]. However, the importance of *Anopheles* in this study should not be interpreted only through the lens of malaria. The genus was also linked with other mosquito-associated pathogens, indicating that surveillance focused exclusively on malaria may miss a broader pathogen spectrum [39, 40]. Similarly, the broad occurrence of *Aedes aegypti* is important because this species is a well-recognized vector of several arboviruses, including dengue, yellow fever, Zika and chikungunya viruses [41, 42]. These findings support the need to view mosquito surveillance in West Africa as a multi-pathogen issue, rather than as a set of separate disease-specific programs.

The country-level differences observed in our database also require careful interpretation. Nigeria had the richest mosquito records and a high diversity of reported pathogens, which may partly reflect its large population, ecological heterogeneity, and greater volume of health-related research [43, 44]. Senegal, by contrast, had particularly rich records of mosquito-associated viruses, likely reflecting its long-standing arbovirus surveillance capacity and the role of the Institut Pasteur de Dakar and national surveillance programs [45, 46]. Therefore, countries with fewer records should not be considered low-risk by default. In many cases, the absence of mosquito or pathogen records may indicate limited sampling, weak laboratory capacity, or incomplete reporting rather than true absence. This is especially relevant for countries where human infections have been reported, but mosquito-based pathogen detection remains limited [47, 48].

One important finding is the mismatch between human infection evidence and pathogen detection in mosquito samples. Human infections, including outbreaks, pathogen isolation, molecular detection, and serological evidence, were documented across more countries than mosquito-based detection records. This pattern is not necessarily contradictory. Human infection data and mosquito infection data are generated through different surveillance systems, with different sensitivities and biases [49]. Human outbreaks may be detected through clinical or public health reporting, whereas detecting pathogens in mosquitoes requires well-timed entomological sampling, accurate species identification, cold-chain preservation, and laboratory testing [50]. Arboviral infections are also difficult to diagnose in many settings because they often resemble malaria or other febrile illnesses [51]. As a result, mosquito-borne viral infections may be underdiagnosed, while mosquito infection rates may be underestimated due to limited field and laboratory surveillance [50]. This gap emphasizes that human case reports alone cannot fully describe transmission ecology, and mosquito-based surveillance remains essential for identifying local vector–pathogen associations.

The uneven documentation of mosquito-associated viruses and parasites also reflects broader differences in disease attention and diagnostic capacity [52]. Malaria remains the dominant mosquito-borne disease burden in sub-Saharan Africa, and substantial resources have historically been directed toward malaria control and surveillance [7]. This focus is necessary, but it may also contribute to under-recognition of arboviruses and other mosquito-associated pathogens. In areas where febrile illnesses are routinely attributed to malaria, dengue, chikungunya, Zika, yellow fever, or Rift Valley fever may be missed without targeted laboratory testing [52]. Previous work has also suggested that climate change could alter the relative burden of malaria and arboviral diseases in Africa [53]. In this context, strengthening arbovirus detection should not be seen as competing with malaria control, but as a necessary extension of integrated mosquito-borne disease surveillance.

Our ecological niche models further show that mosquito distributions in West Africa are shaped by both environmental and human-related factors. Land cover was important across the modeled species, while temperature seasonality, annual precipitation, and population density contributed strongly to the distributions of several key mosquitoes. These results are biologically plausible. Temperature affects mosquito survival, biting activity and pathogen development, while rainfall influences the availability and persistence of larval habitats [54]. Land cover reflects breeding and resting environments, and population density may capture the influence of urbanization, water storage, waste accumulation, and human blood-meal availability. Rapid urban growth in West Africa, often accompanied by inadequate drainage, unreliable water supply and environmental pollution, can create favorable conditions for container-breeding mosquitoes such as *Ae. aegypti* [55, 56]. The contribution of population density in our models is therefore important because it shows that mosquito distribution cannot be explained by climate alone. Human settlement patterns and local infrastructure are also part of the ecological context in which mosquito populations persist [57, 58].

The model outputs suggest that several key mosquito species may occur beyond their currently reported ranges. This should be interpreted as a hypothesis for field validation, not as proof of confirmed presence. For example, species with few occurrence records but broad predicted suitability may be genuinely under-sampled, or they may occupy suitable habitats only under certain local ecological conditions [59]. *Aedes vittatus* is a useful example because it has received less attention than *Aedes aegypti*, yet it has been implicated in the transmission of several arboviruses [60, 61]. Predicted suitability beyond known records therefore helps identify areas where additional entomological surveys would be most informative [62]. Similar caution applies to predicted suitable areas for *Mansonia* and *Culex* species, whose occurrence may depend on local aquatic habitats, vegetation and sampling methods [63, 64]. In this sense, the models are most useful as tools for prioritizing surveillance rather than as definitive maps of species presence [65].

These findings have practical implications for mosquito and pathogen surveillance in West Africa. First, surveillance should prioritize areas where models predict high suitability, but occurrence records are absent or scarce. Second, countries with human infection evidence but limited mosquito-based pathogen detection should be considered important targets for integrated vector and pathogen surveys. Third, surveillance should include both medically dominant genera and less frequently reported species that may be overlooked by routine sampling. Finally, pathogen testing in field-collected mosquitoes should cover both viruses and parasites, especially in areas where clinical evidence suggests transmission but the local vector–pathogen relationship remains unclear. Such an approach would help move from passive documentation of outbreaks toward more proactive risk mapping.

Our study has several limitations. First, some relevant evidence may not have been captured because studies without linked entomological data or reliable species-level mosquito identification were excluded. Accordingly, the absence of records in some areas should not be interpreted as true absence. Second, the literature search was restricted to English-language databases. This restriction helped ensure a consistent and reproducible screening and data-extraction process across PubMed, Web of Science, Embase, and Scopus, but may have led to the omission of relevant local studies from Francophone West African countries. Third, the ecological niche modeling results should be interpreted with caution, because AUC alone cannot fully assess model performance and the mostly cross-sectional data limited our ability to evaluate long-term temporal changes in mosquito distributions and pathogen activity [66].

## Conclusions

By integrating mosquito occurrence records, mosquito-associated pathogen detections, and human infection evidence, this study establishes a regional database and evidence base for West Africa. Overall, the results indicate that current records are incomplete and unevenly distributed across the region: occurrence data for key vector species remain absent in many areas, and mosquito-based detection of viral and parasitic pathogens is limited relative to evidence of human infection. Ecological niche models further suggest that some important mosquito species may have suitable habitats beyond their reported ranges, implying that their current distributions are likely underestimated. These findings point to the need for targeted field surveys in under-sampled and model-predicted suitable areas, broader pathogen testing in field-collected mosquitoes, and more coordinated regional data sharing to support mosquito-borne disease surveillance and control in West Africa.

## Supplementary Information

Supplementary Material 1

## Data Availability

Data were collected from multiple sources, including literature review, related websites, and GenBank (https://www.ncbi.nlm.nih.gov/genbank/). Sources of evidence for human infection of mosquito-borne pathogens in countries of West Africa are shown in Supplementary Text 2. Source data are provided with this paper.

## Abbreviations

MBDs: Mosquito-borne diseases
MAVs: Mosquito-associated viruses
WNV: West Nile virus
YFV: Yellow fever virus
RVFV: Rift Valley fever virus
ENM: Ecological niche modeling
AUC: Area under the curve
