# Supplementary material for: Mapping mosquitoes and their associated pathogens in West Africa
Source: Infect Dis Poverty. 2026 Aug 20;15:91. doi: 10.1186/s40249-026-01492-z (PMC13491993; doi:10.1186/s40249-026-01492-z)
Supplement: Supplementary file 1 [file 40249_2026_1492_MOESM1_ESM.pdf]

Supplementary information

Table of contents

Page	Item
1	Supplementary Text 1. Basic characteristics of data collection and mapping
2	Supplementary Fig. 1. The spatial distribution of the mosquito genus *Aedes* in West Africa
3	Supplementary Fig. 2. The spatial distribution of the mosquito genus *Anopheles* in West Africa
4	Supplementary Fig. 3. The spatial distribution of the mosquito genus *Coquillettidia* in West Africa
5	Supplementary Fig. 4. The spatial distribution of the mosquito genus *Culex* in West Africa
6	Supplementary Fig. 5. The spatial distribution of the mosquito genus *Eretmapodites* in West Africa
7	Supplementary Fig. 6. The spatial distribution of the mosquito genus *Uranotaenia* in West Africa
8	Supplementary Fig. 7. The spatial distribution of the other relatively rare mosquito genus (<10 species) in West Africa
9	Supplementary Table 1. Summary of mosquito species in various countries in West Africa
14	Supplementary Fig. 8. Literature number with recording positive of the mosquito-associated pathogens in West Africa
15	Supplementary Fig. 9. Meta-analysis of the positivity rate of each *Aedes*-associated pathogen
27	Supplementary Fig. 10. Meta-analysis of the positivity rate of each *Anopheles*-associated pathogen
38	Supplementary Fig. 11. Meta-analysis of the positivity rate of each *Culex*-associated pathogen
45	Supplementary Fig. 12. Meta-analysis of the positivity rate of each *Mansonia*-associated pathogen
48	Supplementary Fig. 13. Meta-analysis of the positivity rate of each *Mimomyia*-associated pathogen
49	Supplementary Text 2. Evidence for human infection of mosquito-borne pathogens in countries of West Africa
60	Supplementary Fig. 14. Jackknife plots of Maxent model for *Aedes aegypti* prediction.
61	Supplementary Fig. 15. Response curves of environmental variables to probability of *Aedes aegypti* presence
62	Supplementary Fig. 16. Jackknife plots of Maxent model for *Aedes luteocephalus* prediction
63	Supplementary Fig. 17. Response curves of environmental variables to probability of *Aedes luteocephalus* presence
64	Supplementary Fig. 18. Jackknife plots of Maxent model for *Aedes vittatus* prediction
65	Supplementary Fig. 19. Response curves of environmental variables to probability of *Aedes vittatus* presence
66	Supplementary Fig. 20. Jackknife plots of Maxent model for *Anopheles funestus* sensu lato prediction
67	Supplementary Fig. 21. Response curves of environmental variables to probability of *Anopheles funestus* sensu lato presence
68	Supplementary Fig. 22. Jackknife plots of Maxent model for *Anopheles gambiae* sensu lato prediction

69	Supplementary Fig. 23. Response curves of environmental variables to probability of *Anopheles gambiae* sensu lato presence
70	Supplementary Fig. 24. Jackknife plots of Maxent model for *Culex nebulosus* prediction
71	Supplementary Fig. 25. Response curves of environmental variables to probability of *Culex nebulosus* presence
72	Supplementary Fig. 26. Jackknife plots of Maxent model for *Culex quinquefasciatus* prediction
73	Supplementary Fig. 27. Response curves of environmental variables to probability of *Culex quinquefasciatus* presence
74	Supplementary Fig. 28. Jackknife plots of Maxent model for *Mansonia africana* prediction
75	Supplementary Fig. 29. Response curves of environmental variables to probability of *Mansonia africana* presence
76	Supplementary Fig. 30. Jackknife plots of Maxent model for *Mansonia uniformis* prediction
77	Supplementary Fig. 31. Response curves of environmental variables to probability of *Mansonia uniformis* presence
78	Supplementary Fig. 32. Geographical distribution of nine key mosquitoes in West Africa
79	Supplementary Table 2. Details of search strategy for literature review
80	Supplementary Fig. 33. PRISMA flow diagram of study selection process
81	Supplementary Text 3. References for mosquitoes and mosquito-associated pathogens in West Africa
129	Supplementary Table 3. Characteristics of environmental data used for ecological niche modeling
131	Supplementary Table 4. Legend for Land cover type

Supplementary Text 1. Basic characteristics of data collection and mapping

By cleaning and aggregating data from diverse sources, a total of 21,011 records of distribution of mosquitoes were collected, of which 6770 were from literature review, 4205 from GBIF, three from the Malaria Threat Map, and 10033 from VectorMap. After removing duplicates, we ultimately compiled a database comprising 19,578 records on geographic distributions of 302 known mosquito species in 16 mosquito genera.

A total of 744 records of mosquito-associated pathogens were obtained, of which 626 were from literature and 118 from GenBank. De-duplication was performed based on geographical location, vector species and pathogen species, and finally 598 records were obtained. A total of 124 pathogens belonging to 22 families were proved in mosquitoes, including 117 viruses and seven parasites, among which 20 are known to be pathogenic to humans.

The latitude and longitude of the mosquito with the exact collection location were applied for mapping and subsequent ecological niche modeling. There were a total of 16879 pieces of data with precise geographical location, including 283 mosquito species. In order to show the distribution of each mosquito species clearly and in detail, we plotted genera with more than ten mosquito species separately and merged genera with less than ten species into one map. Mosquito species that were more distributed are represented by filling or shading of different colors, while mosquito species that were less distributed are represented by points of different shapes and colors.

For mosquito-associated pathogens, we used exact locations to map as well and the centroids of administrative regions were utilized if the specific collection locations were not available.

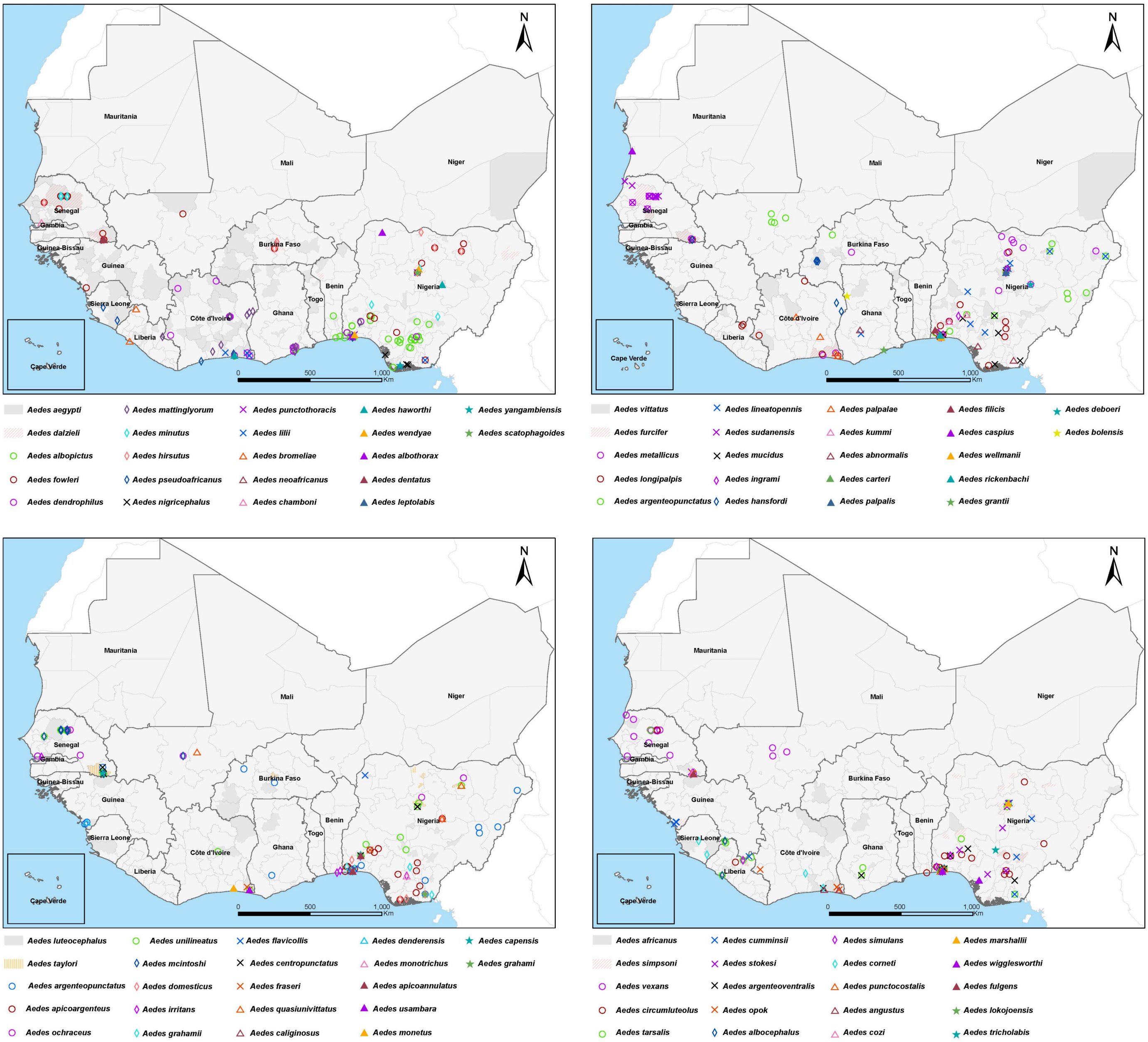

Supplementary Fig. 1. The spatial distribution of the mosquito genus *Aedes* in West Africa.

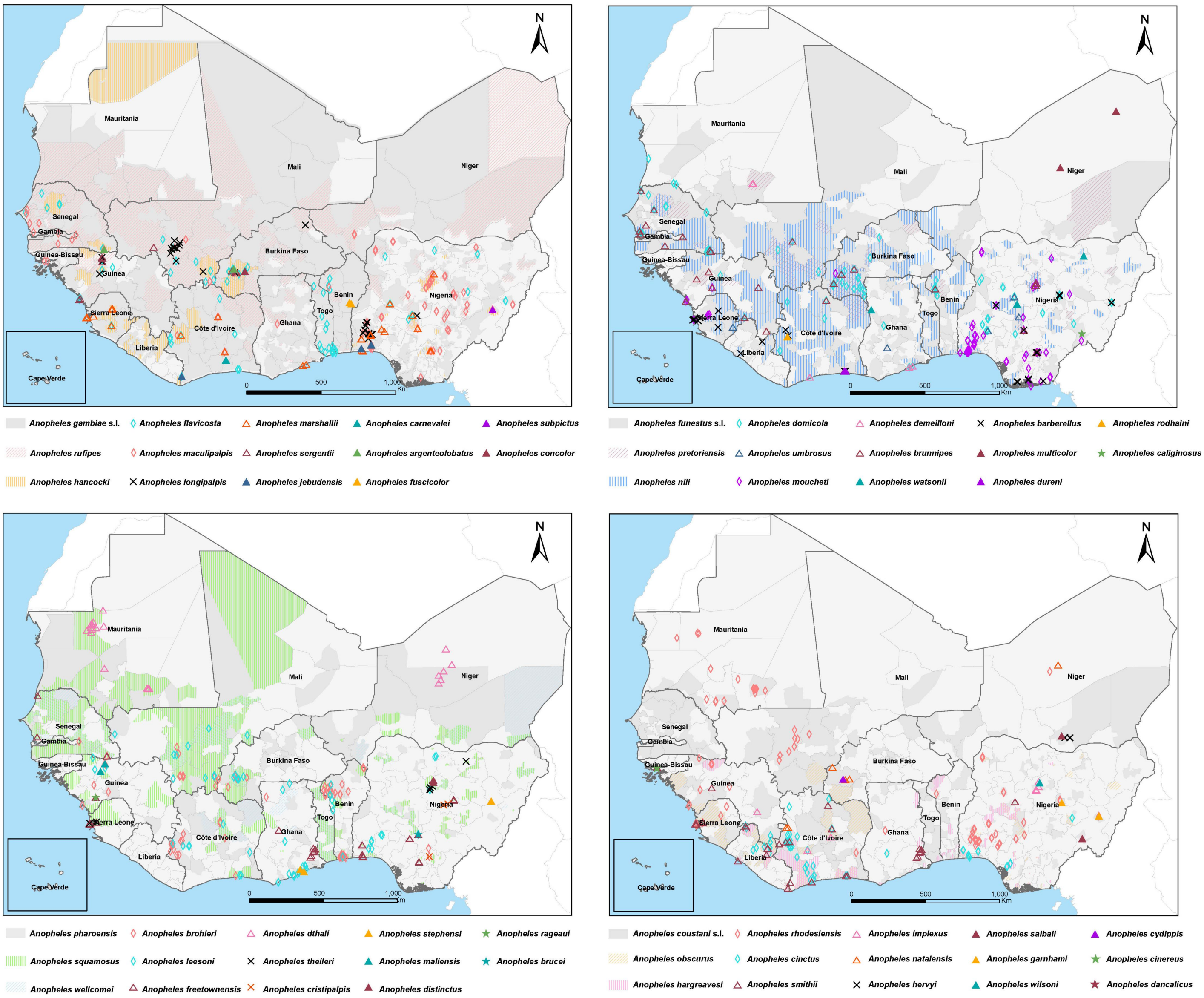

Supplementary Fig. 2. The spatial distribution of the mosquito genus *Anopheles* in West Africa.

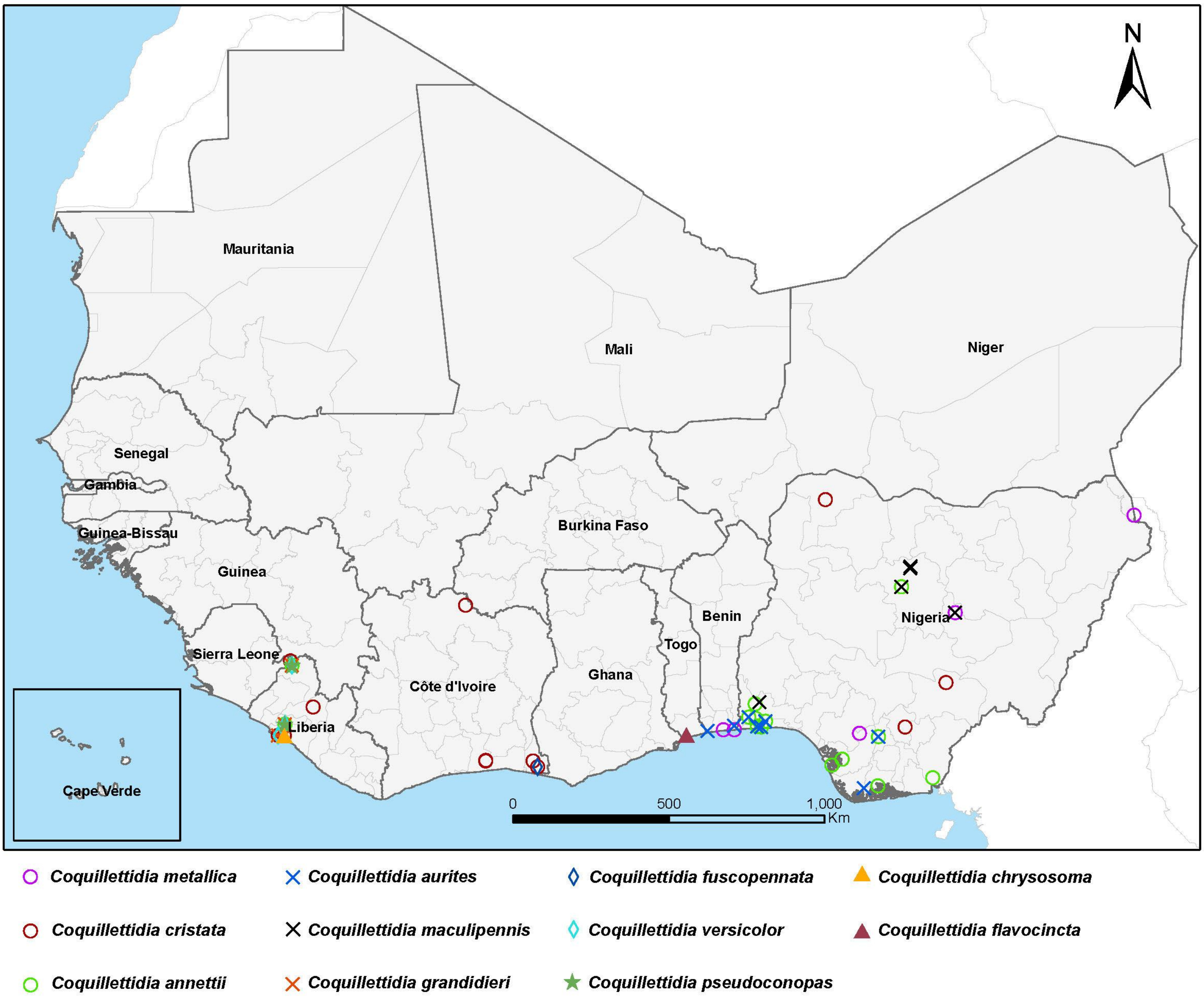

Supplementary Fig. 3. The spatial distribution of the mosquito genus *Coquillettidia* in West Africa.

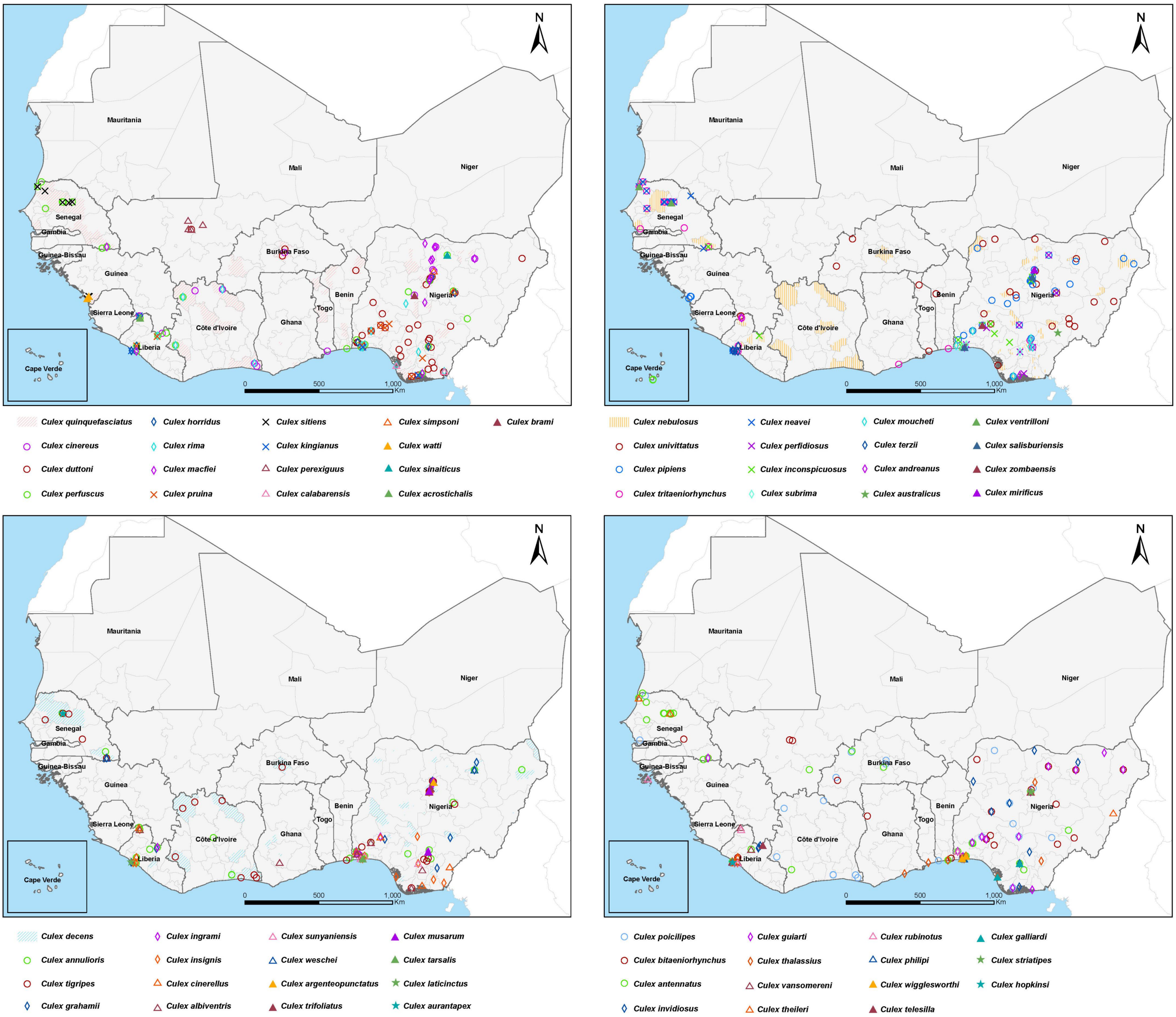

Supplementary Fig. 4. The spatial distribution of the mosquito genus *Culex* in West Africa.

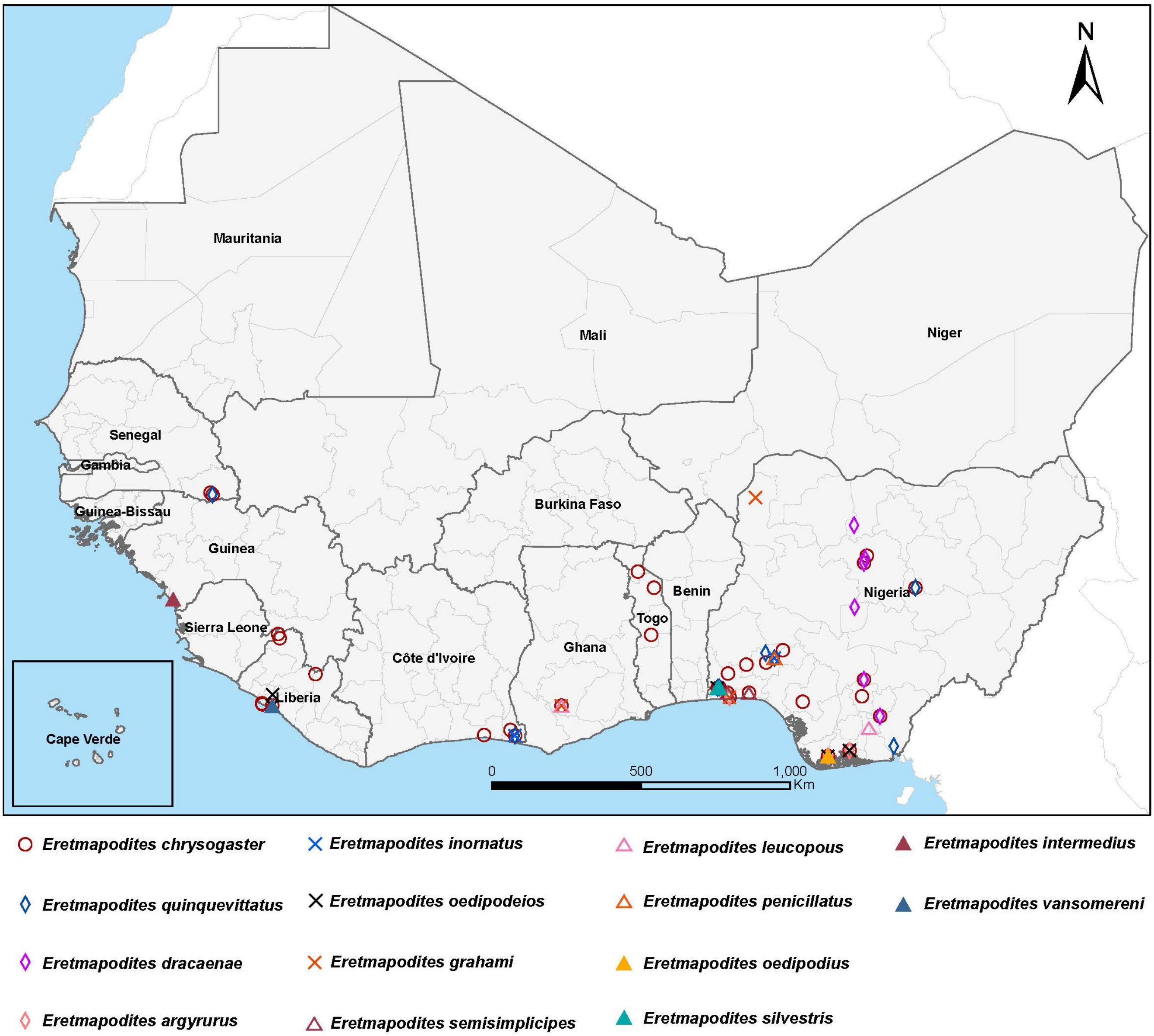

Supplementary Fig. 5. The spatial distribution of the mosquito genus *Eretmapodites* in West Africa.

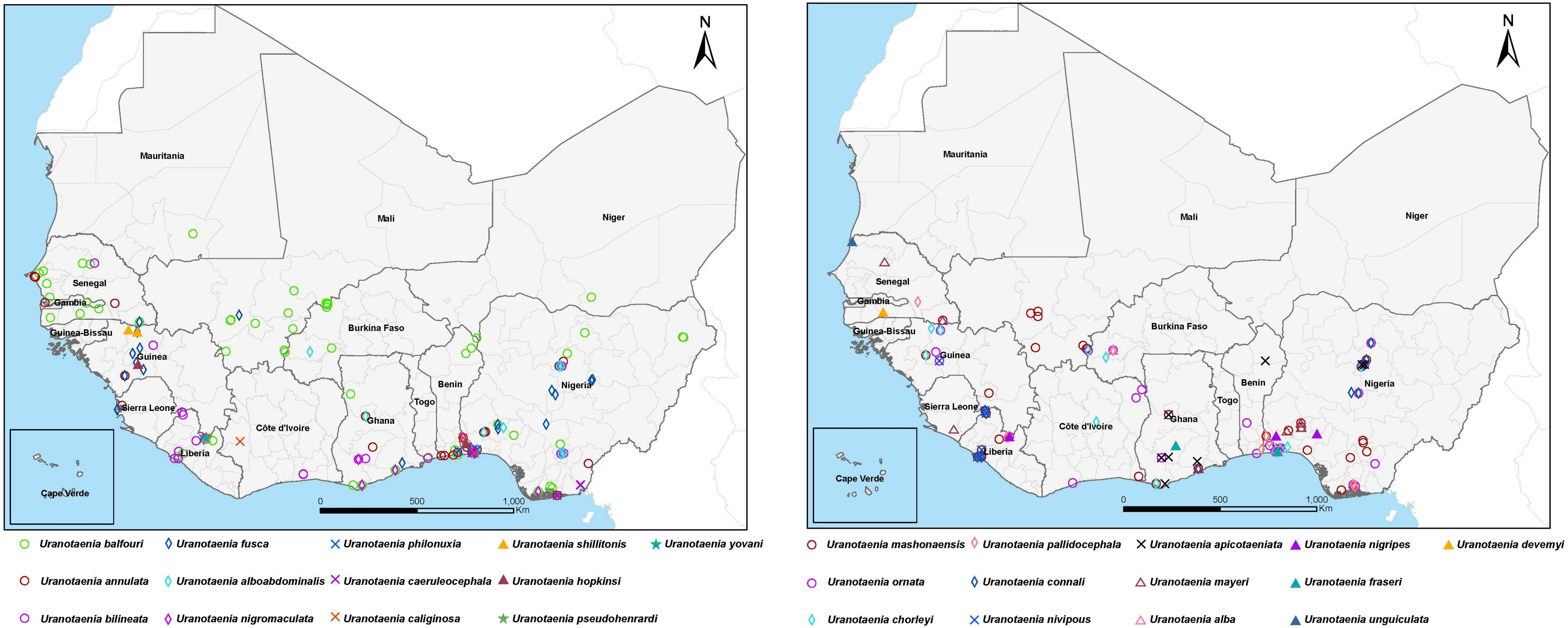

Supplementary Fig. 6. The spatial distribution of the mosquito genus *Uranotaenia* in West Africa.

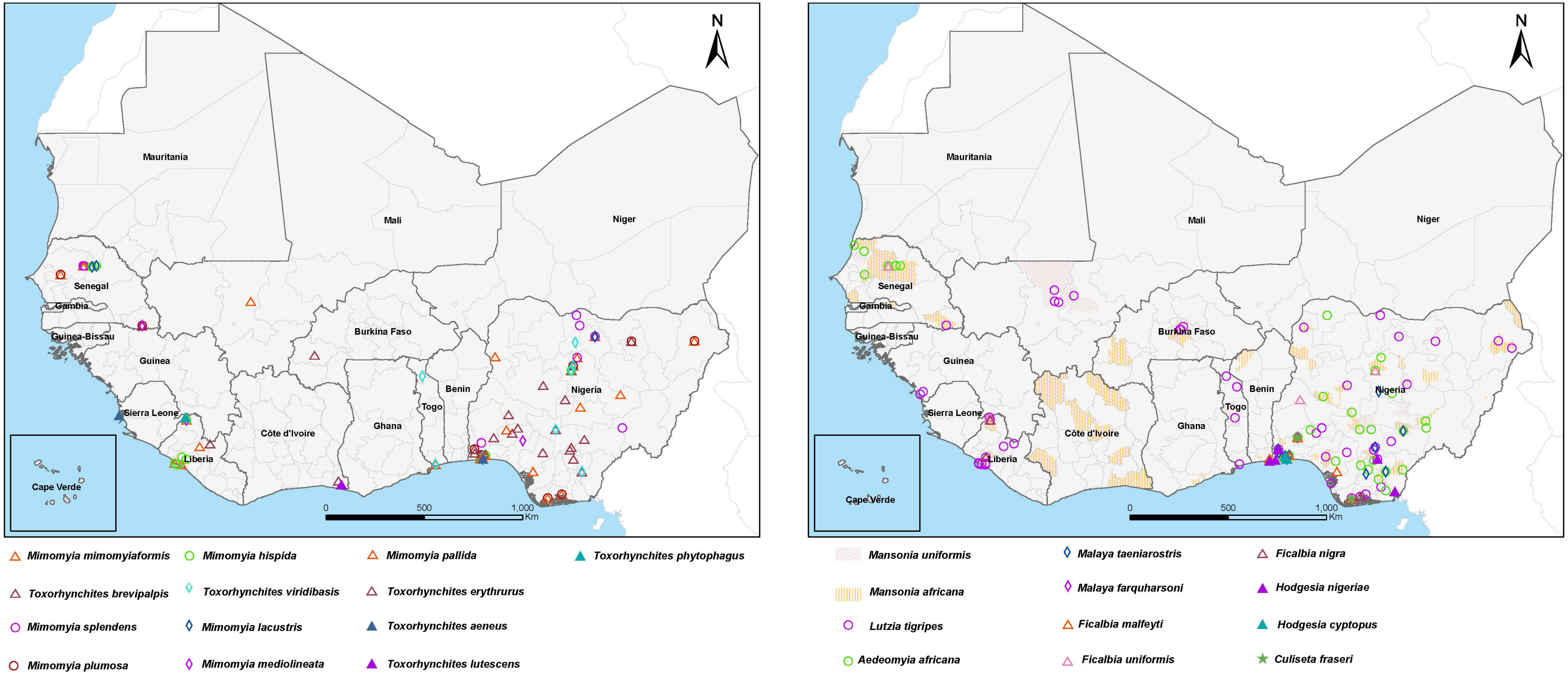

Supplementary Fig. 7. The spatial distribution of the other relatively rare mosquito genus (<10 species) in West Africa.

Supplementary Table 1. Summary of mosquito species in various countries in West Africa

Country	Genus	Species
Benin	*Aedes*	*Ae. aegypti, Ae. albopictus, Ae. circumluteolus, Ae. dalzieli, Ae. irritans, Ae. luteocephalus, Ae. palpalae, Ae. simpsoni, Ae. tarsalis, Ae. vittatus*
	Anopheles	*An. brohieri, An. brunnipes, An. coustani* s.l., *An. domicola, An. flavicosta, An. funestus* s.l., *An. fuscicolor, An. gambiae* s.l., *An. hargreavesi, An. leesoni, An. maculipalpis, An. nili* s.l., *An. obscurus, An. pharoensis, An. pretoriensis, An. rhodesiensis, An. rufipes, An. squamosus, An. wellcomei*
	Coquillettidia	*Cq. aurites, Cq. cristata, Cq. metallica*
	Culex	*Cx. annulioris, Cx. antennatus, Cx. bitaeniorhynchus, Cx. decens, Cx. duttoni, Cx. nebulosus, Cx. perfuscus, Cx. poicilipes, Cx. quinquefasciatus, Cx. thalassius, Cx. tigripes, Cx. tritaeniorhynchus, Cx. univittatus*
	Ficalbia	*Fi. malfeyti*
	Hodgesia	*Ho. nigeriae*
	Mansonia	*Ma. africana, Ma. uniformis*
	Uranotaenia	*Ur. annulata, Ur. apicotaeniata, Ur. balfouri, Ur. ornata, Ur. pallidocephala, Ur. philonuxia*
Burkina Faso	*Aedes*	*Ae. aegypti, Ae. africanus, Ae. argenteopunctatus, Ae. dalzieli, Ae. fowleri, Ae. furcifer, Ae. hansfordi, Ae. hirsutus, Ae. luteocephalus, Ae. metallicus, Ae. opok, Ae. taylori, Ae. vexans, Ae. vittatus*
	Anopheles	*An. argenteolobatus, An. brohieri, An. brunnipes, An. coustani* s.l., *An. cydippis, An. domicola, An. flavicosta, An. funestus* s.l., *An. gambiae* s.l., *An. hancocki, An. leesoni, An. longipalpis, An. maculipalpis, An. moucheti, An. natalensis, An. nili* s.l., *An. obscurus, An. pharoensis, An. pretoriensis, An. rufipes, An. sergentii, An. squamosus, An. subpictus, An. wellcomei*
	Culex	*Cx. antennatus, Cx. bitaeniorhynchus, Cx. cinereus, Cx. decens, Cx. duttoni, Cx. nebulosus, Cx. pipiens, Cx. poicilipes, Cx. quinquefasciatus, Cx. tigripes, Cx. univittatus*
	Lutzia	*Lt. tigripes*
	Mansonia	*Ma. africana, Ma. uniformis*
	Toxorhynchites	*Tx. brevipalpis*
	Uranotaenia	*Ur. alba, Ur. alboabdominalis, Ur. balfouri, Ur. chorleyi, Ur. mashonaensis, Ur. ornata, Ur. pallidocephala*
Côte d'Ivoire	*Aedeomyia*	*Ad. africana*
	Aedes	*Ae. aegypti, Ae. africanus, Ae. albopictus, Ae. angustus, Ae. apicoargenteus, Ae. argenteopunctatus, Ae. argenteoventralis, Ae. calceatus, Ae. corneti, Ae. denderensis, Ae. dendrophilus, Ae. fascipalpis, Ae. fraseri, Ae. furcifer, Ae. hansfordi, Ae. haworthi, Ae. hirsutus, Ae. lilii, Ae. longipalpis, Ae. luteocephalus, Ae. mattinglyorum, Ae. metallicus, Ae. monetus, Ae. mutilus, Ae. ochraceus, Ae. opok, Ae. palpalae, Ae. pseudoafricanus, Ae. unilineatus, Ae. usambara, Ae. vittatus*
	Anopheles	*An. azaniae, An. barberellus, An. brohieri, An. brunnipes, An. buxtoni, An. carnevalei, An. cinctus, An. coustani* s.l., *An. dancalicus, An. demeilloni, An. domicola, An. dureni, An. flavicosta, An. funestus* s.l., *An. gambiae* s.l., *An. hancocki, An. hargreavesi, An. implexus, An. jebudensis, An. keniensis, An. leesoni, An. maculipalpis, An. marshallii, An. natalensis, An. nili* s.l., *An. obscurus, An. pharoensis, An. pretoriensis, An. rhodesiensis, An. rodhaini, An. rufipes, An. salbaii, An. smithii, An. squamosus, An. wellcomei*
	Coquillettidia	*Cq. cristata, Cq. fuscopennata, Cq. metallica*
	Culex	*Cx. annulioris, Cx. antennatus, Cx. argenteopunctatus, Cx. bitaeniorhynchus, Cx. cinerellus, Cx. cinereus, Cx. decens, Cx. giganteus, Cx. horridus, Cx. ingrami, Cx. moucheti, Cx. nebulosus, Cx. perfuscus, Cx. pipiens, Cx. poicilipes, Cx. quinquefasciatus, Cx. rima, Cx. rubinotus, Cx. simpliciforceps, Cx. telesilla, Cx. tigripes, Cx. zombaensis*
	Eretmapodites	*Er. chrysogaster, Er. inornatus, Er. quinquevittatus, Er. subsimplicipes*
	Mansonia	*Ma. africana, Ma. uniformis*
	Ochlerotatus	*Oc. harrisoni, Oc. subalbirostris*
	Toxorhynchites	*Tx. brevipalpis, Tx. lutescens*
	Uranotaenia	*Ur. balfouri, Ur. bilineata, Ur. caliginosa, Ur. chorleyi, Ur. mashonaensis, Ur. ornata*
Cape Verde	*Aedes*	*Ae. aegypti*
	Anopheles	*An. gambiae* s.l., *An. pretoriensis*
	Culex	*Cx. pipiens, Cx. quinquefasciatus*
Gambia	*Aedes*	*Ae. aegypti, Ae. albocephalus, Ae. argenteopunctatus, Ae. chamboni, Ae. circumluteolus, Ae. dalzieli, Ae. fowleri, Ae. furcifer, Ae. irritans, Ae. luteocephalus, Ae. metallicus, Ae.*

		minutus, Ae. nigricephalus, Ae. ochraceus, Ae. pseudoafricanus, Ae. punctothoracis, Ae. scatophagoides, Ae. simpsoni, Ae. unilineatus, Ae. vexans, Ae. vittatus
	Anopheles	*An. brohieri, An. brunnipes, An. coustani* s.l., *An. funestus* s.l., *An. gambiae* s.l., *An. maculipalpis, An. nili* s.l., *An. pharoensis, An. rufipes, An. squamosus, An. wellcomei*
	Coquillettidia	*Cq. metallica*
	Culex	*Cx. annulioris, Cx. antennatus, Cx. bitaeniorhynchus, Cx. cinereus, Cx. decens, Cx. duttoni, Cx. grahamii, Cx. inconspicuosus, Cx. invidiosus, Cx. neavei, Cx. nebulosus, Cx. perexiguus, Cx. pipiens, Cx. poicilipes, Cx. quinquefasciatus, Cx. telesilla, Cx. thalassius, Cx. tigripes, Cx. tritaeniorhynchus, Cx. univittatus, Cx. weschei*
	Mansonia	*Ma. africana, Ma. uniformis*
	Mimomyia	*Mi. hispida, Mi. mediolineata*
	Toxorhynchites	*Tx. brevipalpis*
	Uranotaenia	*Ur. annulata, Ur. balfouri*
Ghana	*Aedes*	*Ae. abnormalis, Ae. aegypti, Ae. africanus, Ae. albopictus, Ae. argenteopunctatus, Ae. argenteoventralis, Ae. bolensis, Ae. dendrophilus, Ae. grantii, Ae. hirsutus, Ae. lilii, Ae. lineatopennis, Ae. luteocephalus, Ae. mattinglyorum, Ae. simpsoni, Ae. tarsalis, Ae. vittatus*
	Anopheles	*An. brohieri, An. cinctus, An. coustani* s.l., *An. demeilloni, An. domicola, An. freetownensis, An. funestus* s.l., *An. gambiae* s.l., *An. hargreavesi, An. leesoni, An. maculipalpis, An. marshallii, An. nili* s.l., *An. obscurus, An. pharoensis, An. pretoriensis, An. rhodesiensis, An. rufipes, An. smithii, An. squamosus, An. stephensi, An. umbrosus, An. watsonii, An. wellcomei*
	Culex	*Cx. albiventris, Cx. annulioris, Cx. antennatus, Cx. bitaeniorhynchus, Cx. decens, Cx. duttoni, Cx. ingrami, Cx. nebulosus, Cx. pipiens, Cx. quinquefasciatus, Cx. rima, Cx. thalassius, Cx. tritaeniorhynchus, Cx. watti*
	Eretmapodites	*Er. chrysogaster, Er. grahami, Er. intermedius, Er. leucopous, Er. quinquevittatus*
	Lutzia	*Lt. tigripes*
	Mansonia	*Ma. africana, Ma. uniformis*
	Uranotaenia	*Ur. alboabdominalis, Ur. annulata, Ur. apicotaeniata, Ur. balfouri, Ur. bilineata, Ur. chorleyi, Ur. connali, Ur. fraseri, Ur. fusca, Ur. mashonaensis, Ur. mayeri, Ur. nigromaculata, Ur. ornata*
Guinea	*Aedes*	*Ae. aegypti, Ae. africanus, Ae. argenteopunctatus, Ae. cumminsii, Ae. denderensis, Ae. fowleri, Ae. longipalpis, Ae. luteocephalus, Ae. simpsoni, Ae. vittatus*
	Anopheles	*An. brohieri, An. brunnipes, An. cinctus, An. coustani* s.l., *An. domicola, An. flavicosta, An. freetownensis, An. funestus* s.l., *An. gambiae* s.l., *An. hancocki, An. hargreavesi, An. implexus, An. leesoni, An. longipalpis, An. maliensis, An. marshallii, An. moucheti, An. nili* s.l., *An. obscurus, An. pharoensis, An. pretoriensis, An. rageaui, An. rhodesiensis, An. rufipes, An. sergentii, An. smithii, An. squamosus*
	Culex	*Cx. invidiosus, Cx. pipiens, Cx. quinquefasciatus, Cx. sitiens, Cx. telesilla, Cx. watti*
	Eretmapodites	*Er. intermedius*
	Lutzia	*Lt. tigripes*
	Uranotaenia	*Ur. annulata, Ur. bilineata, Ur. chorleyi, Ur. fusca, Ur. hopkinsi, Ur. mashonaensis, Ur. nivipous, Ur. ornata, Ur. shillitonis*
Guinea-Bissau	*Aedes*	*Ae. aegypti*
	Anopheles	*An. cinereus, An. coustani* s.l., *An. dancalicus, An. funestus* s.l., *An. gambiae* s.l., *An. hargreavesi, An. maculipalpis, An. nili* s.l., *An. pharoensis, An. rufipes, An. smithii, An. squamosus*
	Culex	*Cx. rubinotus, Cx. theileri*
	Uranotaenia	*Ur. balfouri*
Liberia	*Aedes*	*Ae. aegypti, Ae. africanus, Ae. albocephalus, Ae. apicoargenteus, Ae. argenteopunctatus, Ae. bromeliae, Ae. circumluteolus, Ae. cumminsii, Ae. fuscinervis, Ae. galloisi, Ae. longipalpis, Ae. simpsoni, Ae. simulans, Ae. tarsalis, Ae. vexans, Ae. vittatus*
	Anopheles	*An. barberellus, An. cinctus, An. coustani* s.l., *An. funestus* s.l., *An. gambiae* s.l., *An. hancocki, An. hargreavesi, An. nili* s.l., *An. obscurus, An. smithii, An. squamosus, An. theileri*
	Coquillettidia	*Cq. annettii, Cq. aurites, Cq. chrysosoma, Cq. cristata, Cq. fuscopennata, Cq. grandidieri, Cq. maculipennis, Cq. metallica, Cq. pseudoconopas, Cq. versicolor*
	Culex	*Cx. acrostichalis, Cx. albiventris, Cx. andreanus, Cx. annulioris, Cx. antennatus, Cx. bitaeniorhynchus, Cx. cinerellus, Cx. cinereus, Cx. decens, Cx. grahamii, Cx. hopkinsi, Cx. horridus, Cx. inconspicuosus, Cx. ingrami, Cx. insignis, Cx. invidiosus, Cx. kingianus, Cx. laticinctus, Cx. macfiei, Cx. moucheti, Cx. neavei, Cx. nebulosus, Cx. perfuscus, Cx. pipiens, Cx. pruina, Cx. quinquefasciatus, Cx. rima, Cx. rubinotus, Cx. terzii, Cx. thalassius, Cx. theileri, Cx. tigripes, Cx. tritaeniorhynchus, Cx. univittatus, Cx. vansomereni*
	Eretmapodites	*Er. chrysogaster, Er. oedipodeios, Er. vansomereni*
	Ficalbia	*Fi. malfeyti, Fi. nigra, Fi. uniformis*

	Lutzia	*Lt. tigripes*
	Mansonia	*Ma. africana, Ma. Uniformis*
	Mimomyia	*Mi. hispida, Mi. mediolineata, Mi.mimomviaformis*
	Toxorhynchites	*Tx. brevipalpis, Tx. phytophagus*
	Uranotaenia	*Ur. annulata, Ur. balfouri, Ur. bilineata, Ur. caeruleocephala, Ur. caliginosa, Ur. chorleyi, Ur. connali, Ur. mashonaensis, Ur. nigripes, Ur. nigromaculata, Ur. nivipous, Ur. ornata, Ur. pallidocephala, Ur. philonuxia, Ur. pseudohenrardi, Ur. shillitonis, Ur. yovani*
Mali	*Aedes*	*Ae. aegypti, Ae. argenteopunctatus, Ae. fowleri, Ae. mcintoshi, Ae. metallicus, Ae. ochraceus, Ae. quasiunivittatus, Ae. vexans*
	Anopheles	*An. brohieri, An. brunnipes, An. coustani* s.l., *An. domicola, An. flavicosta, An. funestus* s.l., *An. gambiae* s.l., *An. hancocki, An. leesoni, An. longipalpis, An. maculipalpis, An. nili* s.l., *An. obscurus, An. pharoensis, An. pretoriensis, An. rhodesiensis, An. rufipes, An. sergentii, An. squamosus, An. wellcomei*
	Culex	*Cx. antennatus, Cx. bitaeniorhynchus, Cx. duttoni, Cx. neavei, Cx. perexiguus, Cx. quinquefasciatus*
	Lutzia	*Lt. tigripes*
	Mansonia	*Ma. uniformis*
	Mimomyia	*Mi. mediolineata*
	Uranotaenia	*Ur. balfouri, Ur. chorleyi, Ur. fusca, Ur. mashonaensis, Ur. ornata*
Mauritania	*Aedes*	*Ae. aegypti, Ae. caspius, Ae. dalzieli, Ae. fowleri, Ae. minutus, Ae. ochraceus, Ae. sudanensis, Ae. vexans, Ae. vittatus*
	Anopheles	*An. coustani* s.l., *An. demeilloni, An. domicola, An. dthali, An. freetownensis, An. funestus* s.l., *An. gambiae* s.l., *An. hancocki, An. pharoensis, An. pretoriensis, An. rhodesiensis, An. rufipes, An. squamosus*
	Culex	*Cx. antennatus, Cx. aurantapex, Cx. bitaeniorhynchus, Cx. decens, Cx. neavei, Cx. perfuscus, Cx. poicilipes, Cx. quinquefasciatus, Cx. sitiens, Cx. tritaeniorhynchus, Cx. univittatus*
	Mansonia	*Ma. africana, Ma. uniformis*
	Mimomyia	*Mi. mediolineata*
	Ochlerotatus	*Oc. caspius*
	Uranotaenia	*Ur. balfouri*
Niger	*Aedes*	*Ae. aegypti, Ae. vittatus*
	Anopheles	*An. coustani* s.l., *An. dthali, An. funestus* s.l., *An. gambiae* s.l., *An. hervyi, An. maculipalpis, An. multicolor, An. natalensis, An. nili* s.l., *An. pharoensis, An. pretoriensis, An. rhodesiensis, An. rufipes, An. salbaii, An. squamosus, An. wellcomei*
	Culex	*Cx. antennatus, Cx. bitaeniorhynchus, Cx. perexiguus, Cx. quinquefasciatus*
	Uranotaenia	*Ur. balfouri*
Nigeria	*Aedeomyia*	*Ad. africana*
	Aedes	*Ae. abnormalis, Ae. aegypti, Ae. africanus, Ae. albocephalus, Ae. albopictus, Ae. albothorax, Ae. apicoannulatus, Ae. apicoargenteus, Ae. argenteopunctatus, Ae. argenteoventralis, Ae. caliginosus, Ae. carteri, Ae. centropunctatus, Ae. circumluteolus, Ae. cumminsii, Ae. dalzieli, Ae. deboeri, Ae. dendrophilus, Ae. dentatus, Ae. domesticus, Ae. filicis, Ae. flavicollis, Ae. fowleri, Ae. fraseri, Ae. furcifer, Ae. galloisi, Ae. grahami, Ae. grahamii, Ae. haworthi, Ae. hirsutus, Ae. ingrami, Ae. irritans, Ae. kummi, Ae. leptolabis, Ae. lilii, Ae. lineatopennis, Ae. lokojoensis, Ae. longipalpis, Ae. luteocephalus, Ae. marshallii, Ae. mattinglyorum, Ae. mcintoshi, Ae. metallicus, Ae. minutus, Ae. monotrichus, Ae. mucidus, Ae. nigricephalus, Ae. ochraceus, Ae. palpalae, Ae. palpalis, Ae. pseudoafricanus, Ae. punctocostalis, Ae. punctothoracis, Ae. quasiunivittatus, Ae. rickenbachi, Ae. scatophagoides, Ae. simpsoni, Ae. simulans, Ae. stokesi, Ae. tarsalis, Ae. taylori, Ae. tricholabis, Ae. unilineatus, Ae. vittatus, Ae. wellmanii, Ae. wendyae, Ae. wigglesworthi, Ae. yangambiensis*
	Anopheles	*An. barberellus, An. brohieri, An. brucei, An. brunnipes, An. caliginosus, An. cinctus, An. concolor, An. coustani* s.l., *An. cristipalpis, An. distinctus, An. domicola, An. flavicosta, An. freetownensis, An. funestus* s.l., *An. gambiae* s.l., *An. garnhami, An. hancocki, An. hargreavesi, An. implexus, An. jebudensis, An. leesoni, An. longipalpis, An. maculipalpis, An. marshallii, An. mauritianus, An. moucheti, An. nili* s.l., *An. obscurus, An. pharoensis, An. pretoriensis, An. rhodesiensis, An. rufipes, An. salbaii, An. smithii, An. squamosus, An. stephensi, An. theileri, An. umbrosus, An. watsonii, An. wellcomei, An. wilsoni*
	Coquillettidia	*Cq. annettii, Cq. aurites, Cq. cristata, Cq. fuscopennata, Cq. maculipennis, Cq. metallica*
	Culex	*Cx. albiventris, Cx. andreanus, Cx. annulioris, Cx. antennatus, Cx. argenteopunctatus, Cx. australicus, Cx. bitaeniorhynchus, Cx. brami, Cx. calabarensis, Cx. cinerellus, Cx. cinereus, Cx. decens, Cx. duttoni, Cx. galliardi, Cx. grahamii, Cx. guiarti, Cx. horridus, Cx. inconspicuosus, Cx. ingrami, Cx. insignis, Cx. invidiosus, Cx. kingianus, Cx. macfiei, Cx. mirificus, Cx. moucheti, Cx. musarum, Cx. nebulosus, Cx. nigripalpus, Cx. perfidiosus, Cx. perfuscus, Cx. philipi, Cx. pipiens, Cx. poicilipes, Cx. pruina, Cx. quinquefasciatus, Cx. rima, Cx. rubinotus, Cx. salinarius, Cx. salisburiensis, Cx. simpsoni, Cx. sinaiticus, Cx. striatipes, Cx. subrima, Cx. sunyaniensis, Cx. tarsalis, Cx. telesilla, Cx. thalassius, Cx.*

		theileri, Cx. tigripes, Cx. trifoliatus, Cx. tritaeniorhynchus, Cx. uniformis, Cx. univittatus, Cx. weschei, Cx. wigglesworthi, Cx. zombaensis
	Culiseta	*Cs. fraseri*
	Eretmapodites	*Er. argyrurus, Er. chrysogaster, Er. dracaenae, Er. grahami, Er. inornatus, Er. leucopous, Er. oedipodeios, Er. oedipodius, Er. penicillatus, Er. quinquevittatus, Er. semisimplicipes, Er. silvestris*
	Ficalbia	*Fi. malfeyti, Fi. nigra, Fi. uniformis*
	Hodgesia	*Ho. cyptopus, Ho. nigeriae*
	Lutzia	*Lt. tigripes*
	Malaya	*Ml. farquharsoni, Ml. taeniarostris*
	Mansonia	*Ma. africana, Ma. uniformis*
	Mimomyia	*Mi. hispida, Mi. lacustris, Mi. mediolineata, Mi. mediolineata, Mi. pallida, Mi. plumosa, Mi. splendens*
	Ochlerotatus	*Oc. vigilax*
	Toxorhynchites	*Tx. aeneus, Tx. brevipalpis, Tx. erythrurus, Tx. viridibasis*
	Uranotaenia	*Ur. alba, Ur. alboabdominalis, Ur. annulata, Ur. apicotaeniata, Ur. balfouri, Ur. bilineata, Ur. caeruleocephala, Ur. caliginosa, Ur. chorleyi, Ur. connali, Ur. fraseri, Ur. fusca, Ur. hopkinsi, Ur. mashonaensis, Ur. mayeri, Ur. nigripes, Ur. nigromaculata, Ur. nivipes, Ur. nivipous, Ur. ornata, Ur. pallidocephala, Ur. philonuxia*
Senegal	*Aedeomyia*	*Ad. africana*
	Aedes	*Ae. aegypti, Ae. africanus, Ae. apicoargenteus, Ae. argenteopunctatus, Ae. bromeliae, Ae. capensis, Ae. centropunctatus, Ae. chamboni, Ae. circumluteolus, Ae. cozi, Ae. cumminsii, Ae. dalzieli, Ae. dentatus, Ae. fowleri, Ae. fulgens, Ae. furcifer, Ae. hansfordi, Ae. hirsutus, Ae. irritans, Ae. longipalpis, Ae. luteocephalus, Ae. mcintoshi, Ae. metallicus, Ae. minutus, Ae. neoafricanus, Ae. ochraceus, Ae. opok, Ae. simulans, Ae. stokesi, Ae. sudanensis, Ae. tarsalis, Ae. taylori, Ae. unilineatus, Ae. vexans, Ae. vittatus*
	Anopheles	*An. argenteolobatus, An. brohieri, An. brunnipes, An. coustani* s.l., *An. domicola, An. flavicosta, An. freetownensis, An. funestus* s.l., *An. gambiae* s.l., *An. hancocki, An. maculipalpis, An. nili* s.l., *An. pharoensis, An. pretoriensis, An. rufipes, An. squamosus, An. wellcomei*
	Culex	*Cx. annulioris, Cx. antennatus, Cx. aurantapex, Cx. bitaeniorhynchus, Cx. cinerellus, Cx. cinereus, Cx. decens, Cx. duttoni, Cx. grahamii, Cx. guiarti, Cx. inconspicuosus, Cx. invidiosus, Cx. macfiei, Cx. neavei, Cx. nebulosus, Cx. perfuscus, Cx. poicilipes, Cx. quinquefasciatus, Cx. sitiens, Cx. striatipes, Cx. theileri, Cx. tigripes, Cx. tritaeniorhynchus, Cx. univittatus, Cx. ventrilloni, Cx. weschei*
	Eretmapodites	*Er. chrysogaster, Er. quinquevittatus*
	Ficalbia	*Fi. uniformis*
	Lutzia	*Lt. tigripes*
	Mansonia	*Ma. africana, Ma. uniformis*
	Mimomyia	*Mi. hispida, Mi. lacustris, Mi. mediolineata, Mi. mediolineata, Mi. plumosa, Mi. splendens*
	Toxorhynchites	*Tx. brevipalpis*
	Uranotaenia	*Ur. annulata, Ur. balfouri, Ur. bilineata, Ur. devemyi, Ur. fusca, Ur. mashonaensis, Ur. mayeri, Ur. pallidocephala, Ur. unguiculata*
Sierra Leone	*Aedeomyia*	*Ad. africana*
	Aedes	*Ae. aegypti, Ae. africanus, Ae. apicoargenteus, Ae. argenteopunctatus, Ae. corneti, Ae. longipalpis, Ae. luteocephalus, Ae. mattinglyorum, Ae. pseudoafricanus, Ae. simulans, Ae. tarsalis, Ae. vittatus*
	Anopheles	*An. barberellus, An. brunnipes, An. cinctus, An. coustani* s.l., *An. domicola, An. flavicosta, An. freetownensis, An. funestus* s.l., *An. gambiae* s.l., *An. hancocki, An. hargreavesi, An. marshallii, An. mauritianus, An. moucheti, An. nili* s.l., *An. obscurus, An. pharoensis, An. rhodesiensis, An. rufipes, An. smithii, An. squamosus, An. theileri, An. umbrosus*
	Culex	*Cx. albiventris, Cx. annulioris, Cx. cinerellus, Cx. cinereus, Cx. decens, Cx. ingrami, Cx. invidiosus, Cx. macfiei, Cx. nebulosus, Cx. quinquefasciatus, Cx. tigripes, Cx. weschei, Cx. wigglesworthi*
	Eretmapodites	*Er. chrysogaster, Er. oedipodius*
	Mansonia	*Ma. uniformis*
	Toxorhynchites	*Tx. aeneus, Tx. brevipalpis*
	Uranotaenia	*Ur. annulata, Ur. fusca, Ur. mashonaensis, Ur. mayeri, Ur. nigripes*
Togo	*Aedes*	*Ae. aegypti, Ae. vittatus*
	Anopheles	*An. coustani* s.l., *An. flavicosta, An. funestus* s.l., *An. gambiae* s.l., *An. hargreavesi, An. nili* s.l., *An. pharoensis, An. rufipes, An. squamosus, An. wellcomei*

	Coquillettidia	*Cq. flavocincta*
	Culex	*Cx. cinereus, Cx. quinquefasciatus, Cx. thalassius, Cx. univittatus*
	Eretmapodites	*Er. chrysogaster*
	Lutzia	*Lt. tigripes*
	Mansonia	*Ma. uniformis*
	Mimomyia	*Mi. mediolineata*
	Toxorhynchites	*Tx. viridibasis*
	Uranotaenia	*Ur. balfouri, Ur. bilineata*

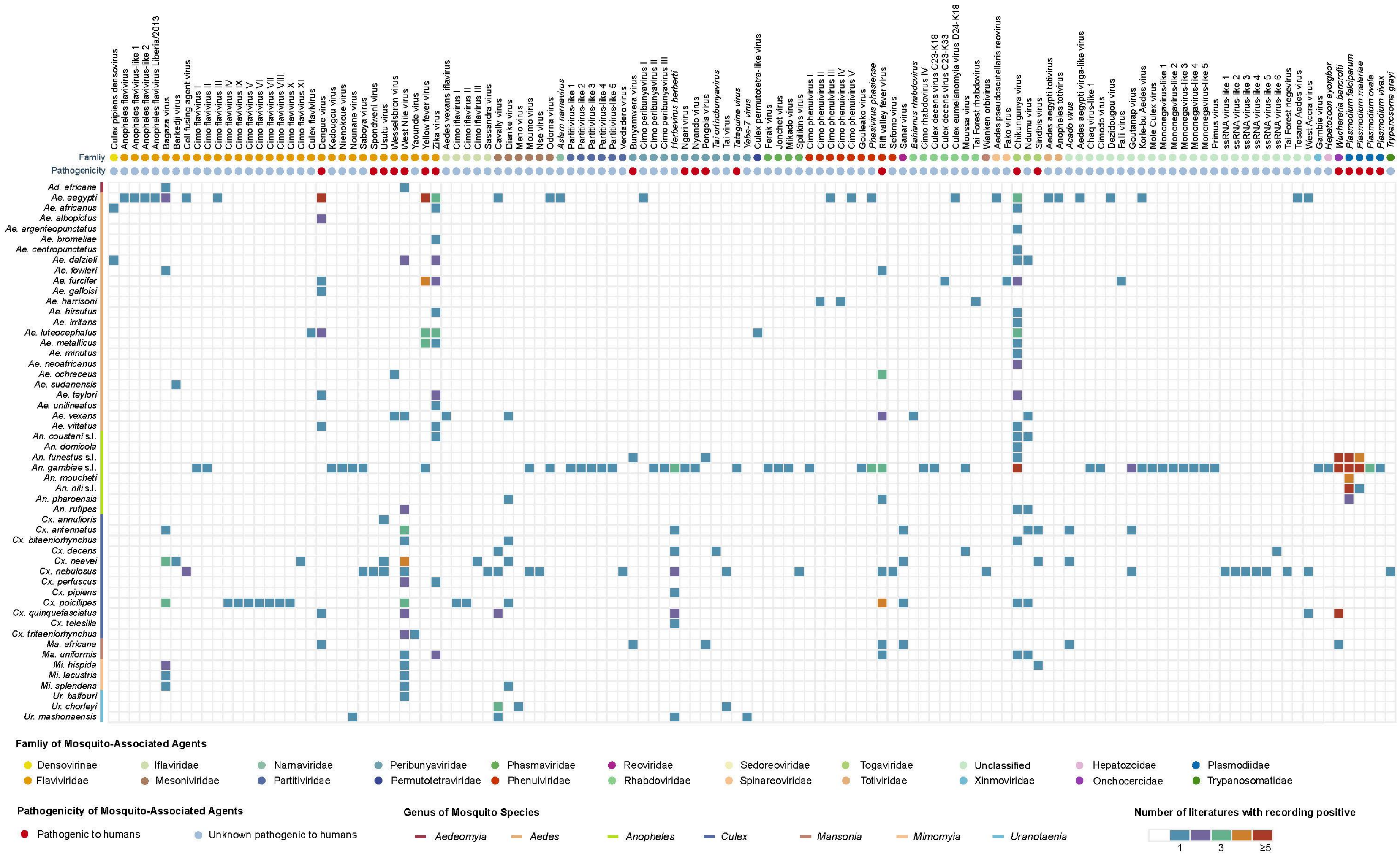

Supplementary Fig. 8. Literature number with recording positive of the mosquito-associated pathogens in West Africa. The top row of circles represents the family of mosquito-associated pathogens, and the bottom row of circles represents the pathogenicity of mosquito-associated pathogens, including definite pathogenicity to humans (Red), and unknown pathogenicity to humans (Blue). The genera of mosquitoes are presented on the left side of picture through bars of distinct colors. The columns from left to right are mosquito-associated pathogens, and the colors of each grid represent the number of literatures recording positive pathogen.

Supplementary Fig. 9. Meta-analysis of the positivity rate of each *Aedes*-associated pathogen. The heterogeneity of combined studies was quantified by I^2 statistic. The random effect model would be applied if $I^2 > 50\%$; Otherwise, the fixed effect model would be applied.

*Aedes*_Bagaza virus

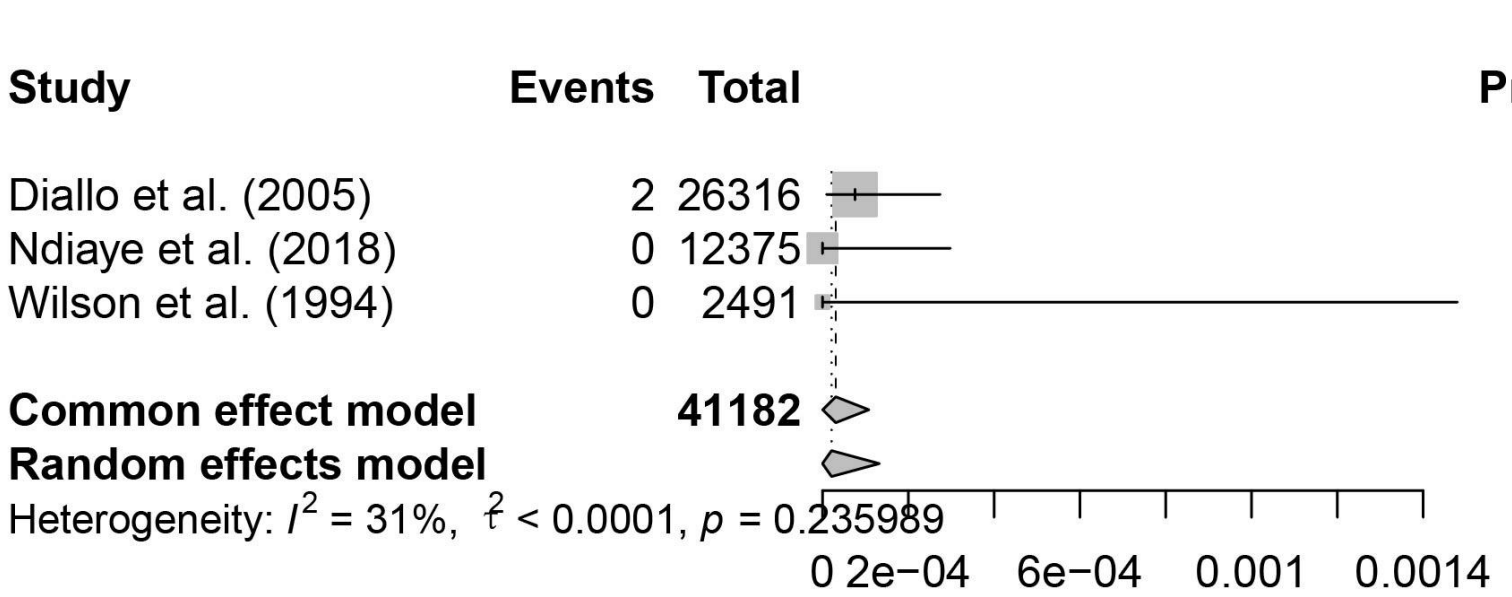

*Aedes*_Chikungunya virus

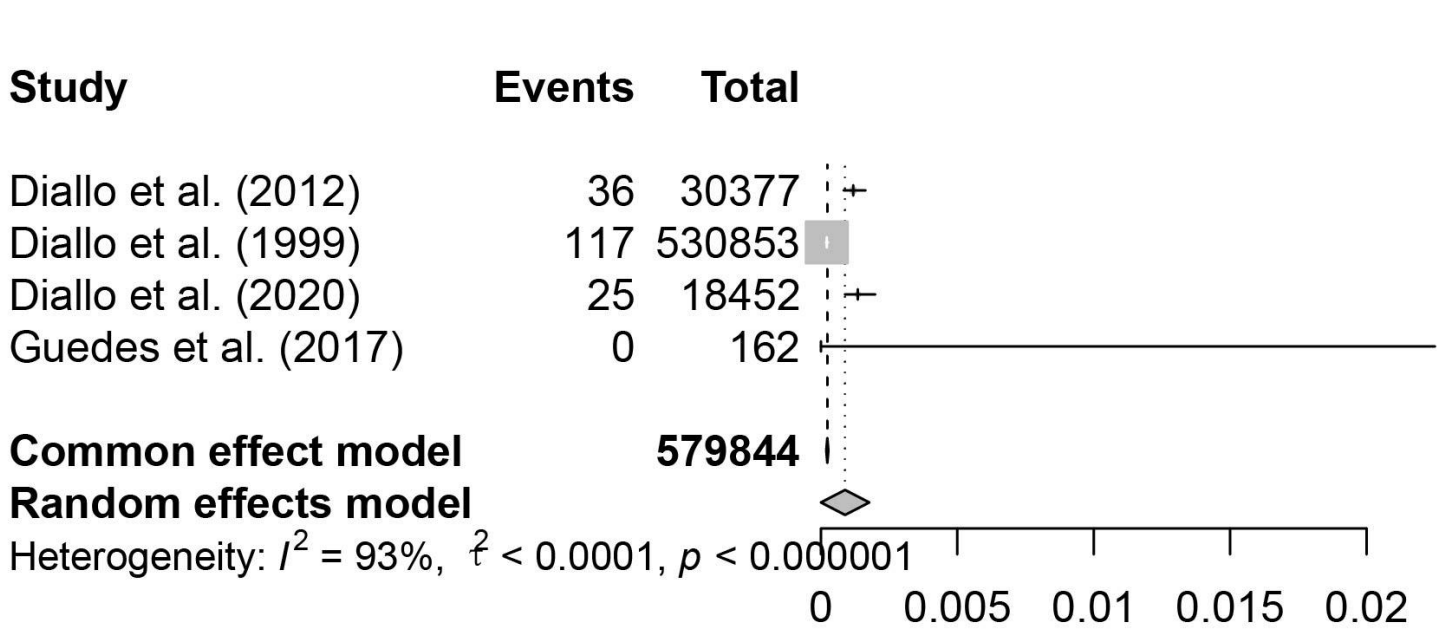

*Aedes*_Dengue virus

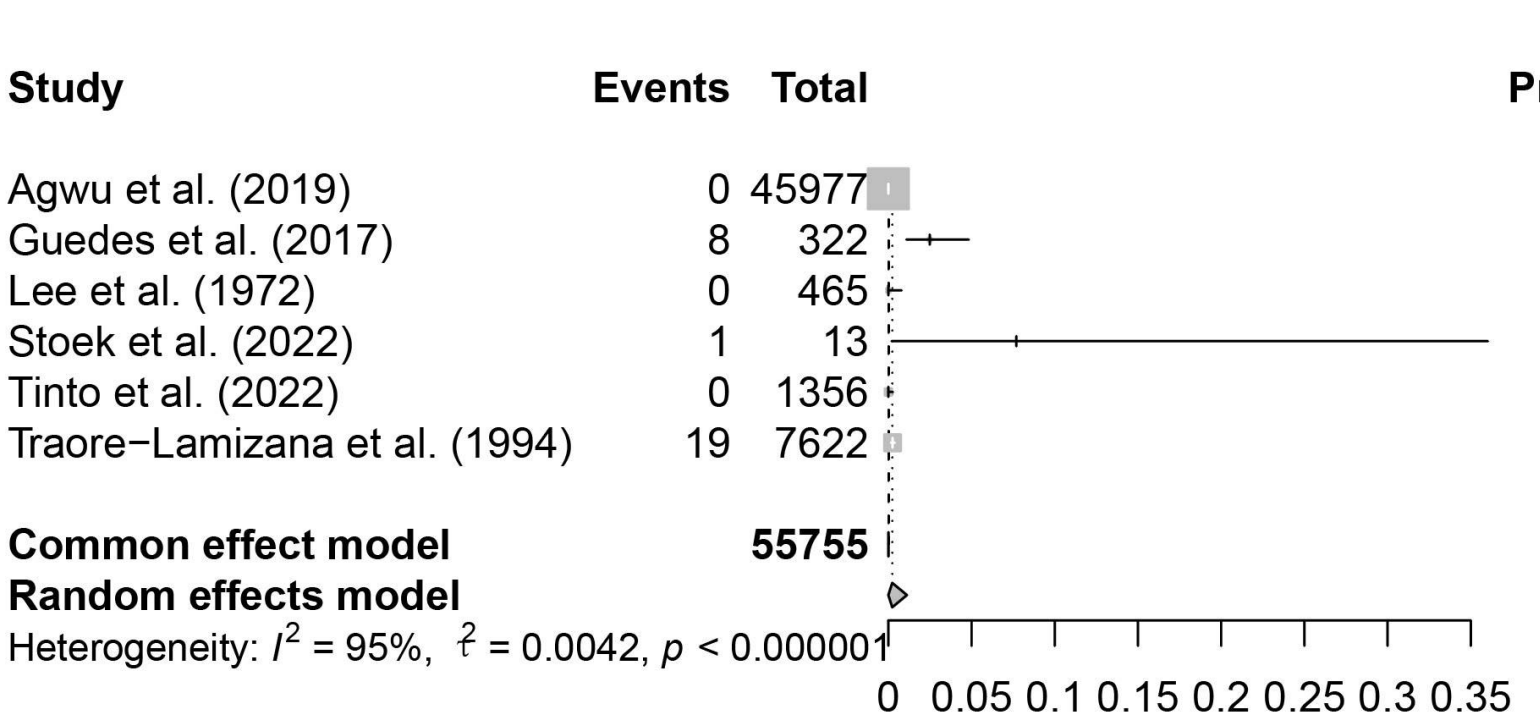

*Aedes*_Rift valley fever virus

Study	Events	Total	Proportion	95% *CI*	Weight (common)	Weight (random)
Ba et al. (2012)	11	56992	0.000193	[0.000096–0.000345]	16.8%	29.2%
Diallo et al. (2005)	0	26316	0.000000	[0.000000–0.000140]	78.7%	32.2%
Fontenille et al. (1995)	13	21580	0.000602	[0.000321–0.001030]	2.0%	15.9%
Ndiaye et al. (2018)	5	12379	0.000404	[0.000131–0.000942]	1.7%	14.6%
Stoek et al. (2022)	0	13	0.000000	[0.000000–0.247053]	0.0%	0.0%
Thabet et al. (2019)	0	66	0.000000	[0.000000–0.054359]	0.0%	0.0%
Wilson et al. (1994)	0	2491	0.000000	[0.000000–0.001480]	0.7%	8.1%
**Common effect model**		**119837**	**0.000052**	**[0.000005–0.000098]**	**100.0%**	**--**
**Random effects model**			**0.000211**	**[0.000030–0.000393]**	**--**	**100.0%**

Heterogeneity: I^2 = 75%, τ^2 < 0.0001, *p* = 0.000458

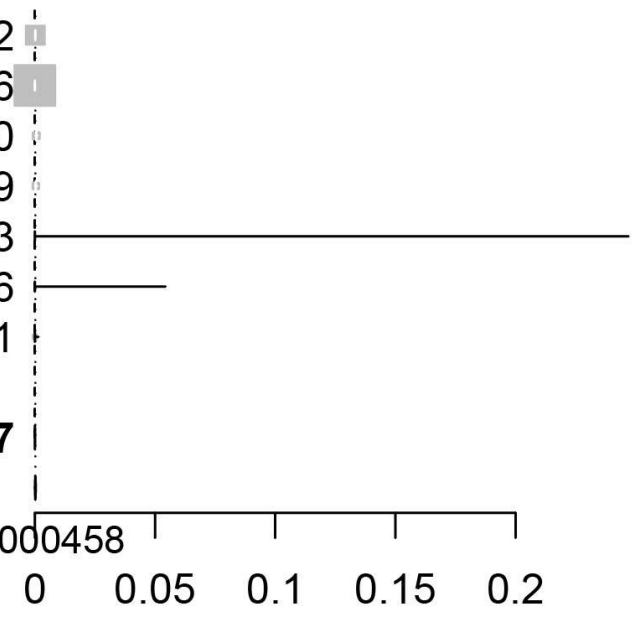

*Aedes*_Wesselsbron virus

Study	Events	Total	Proportion	95% *CI*	Weight (common)	Weight (random)
Diallo et al. (2005)	51	25741	0.001981	[0.001476–0.002604]	67.7%	51.7%
Wilson et al. (1994)	1	2491	0.000401	[0.000010–0.002235]	32.3%	48.3%
**Common effect model**		**28232**	**0.001471**	**[0.001024–0.001918]**	**100.0%**	**--**
**Random effects model**			**0.001218**	**[0.000000–0.002765]**	**--**	**100.0%**

Heterogeneity: I^2 = 90%, τ^2 < 0.0001, *p* = 0.001200

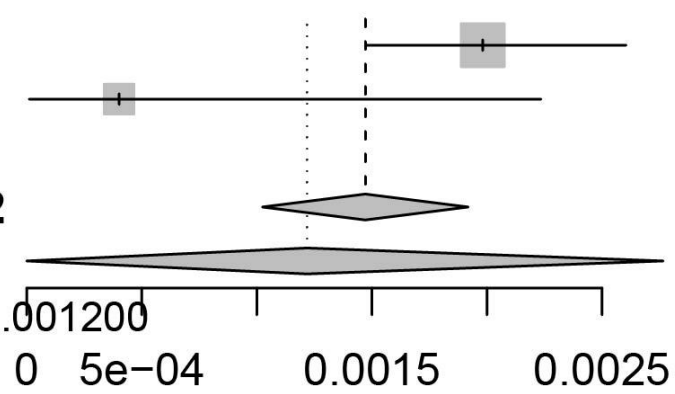

*Aedes*_West Nile virus

Study	Events	Total	Proportion	95% *CI*	Weight (common)	Weight (random)
Diallo et al. (2005)	0	575	0.000000	[0.000000–0.006395]	3.5%	7.4%
Ndiaye et al. (2018)	1	12375	0.000081	[0.000002–0.000450]	74.6%	54.6%
Tinto et al. (2022)	0	1143	0.000000	[0.000000–0.003222]	6.9%	13.5%
Wilson et al. (1994)	2	2491	0.000803	[0.000097–0.002897]	15.0%	24.5%
**Common effect model**		**16584**	**0.000120**	**[0.000011–0.000345]**	**100.0%**	**--**
**Random effects model**			**0.000140**	**[0.000000–0.000553]**	**--**	**100.0%**

Heterogeneity: I^2 = 26%, τ^2 < 0.0001, *p* = 0.258547

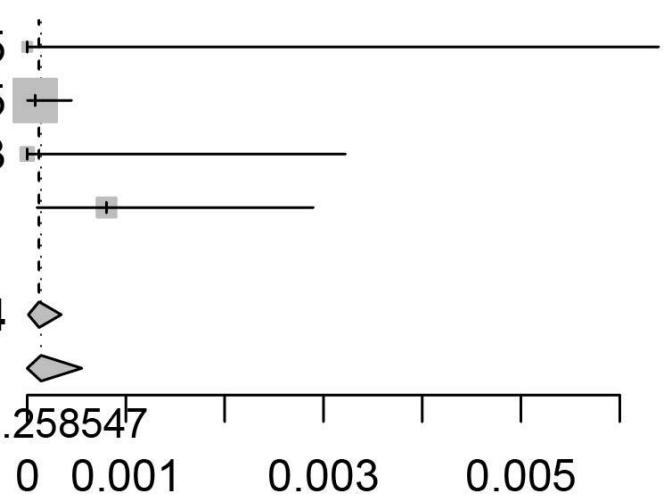

*Aedes*_Yellow fever virus

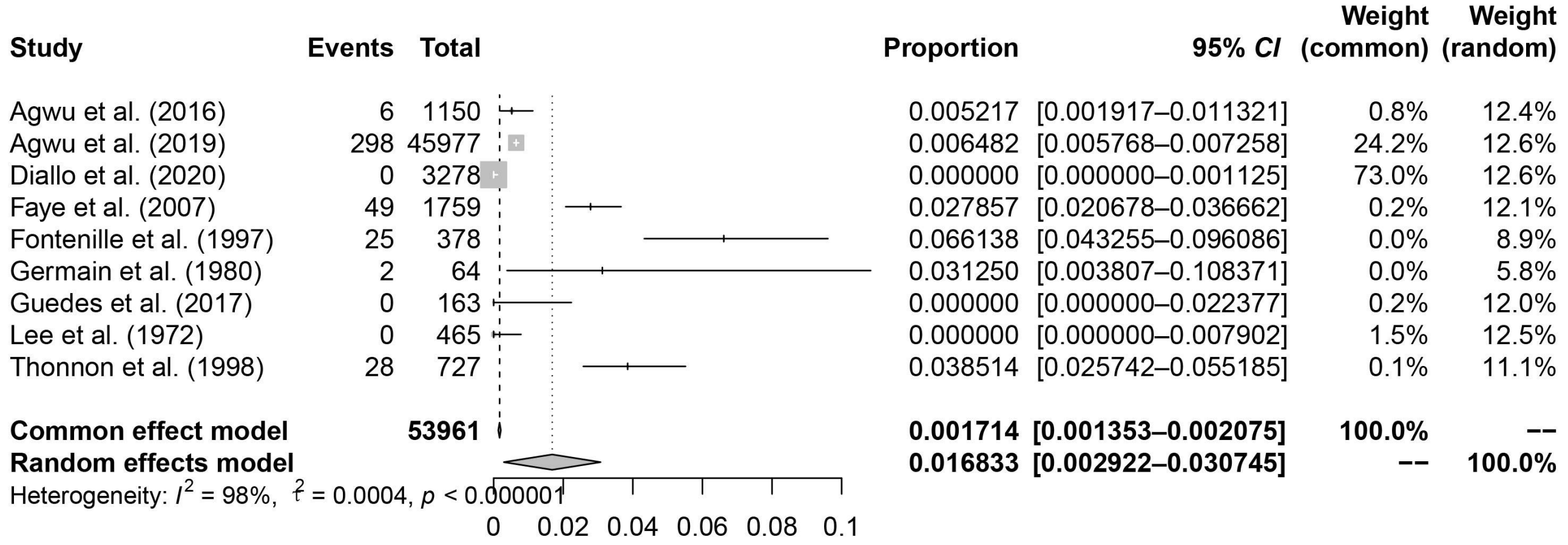

*Aedes*_Zika virus

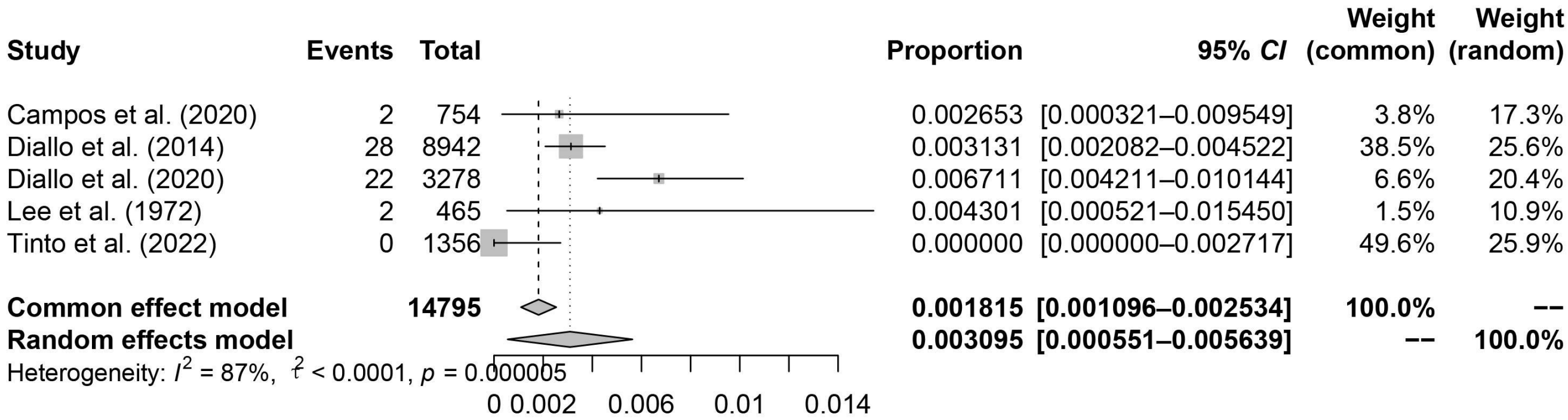

*Aedes aegypti*_Chikungunya virus

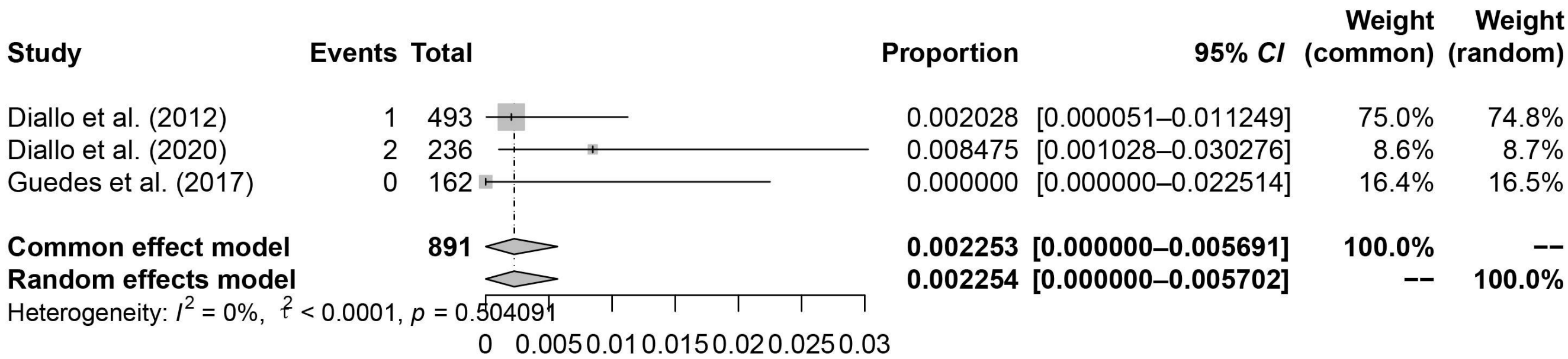

*Aedes aegypti*_Dengue virus

Study	Events	Total	Proportion	95% *CI*	Weight (common)	Weight (random)
Agwu et al. (2019)	0	24157	0.000000	[0.000000–0.000153]	92.9%	24.8%
Guedes et al. (2017)	8	322	0.024845	[0.010786–0.048365]	1.2%	21.9%
Lee et al. (1972)	0	10	0.000000	[0.000000–0.308497]	0.0%	4.6%
Stoek et al. (2022)	1	11	0.090909	[0.002299–0.412780]	0.0%	5.0%
Tinto et al. (2022)	0	1356	0.000000	[0.000000–0.002717]	5.2%	24.1%
Traore−Lamizana et al. (1994)	0	161	0.000000	[0.000000–0.022652]	0.6%	19.6%
**Common effect model**		**26017**	**0.000004**	**[0.000000–0.000067]**	**100.0%**	**--**
**Random effects model**			**0.002499**	**[0.000000–0.015311]**	**--**	**100.0%**

Heterogeneity: $I^2 = 86\%$, $\tau^2 = 0.0057$, $p < 0.000001$

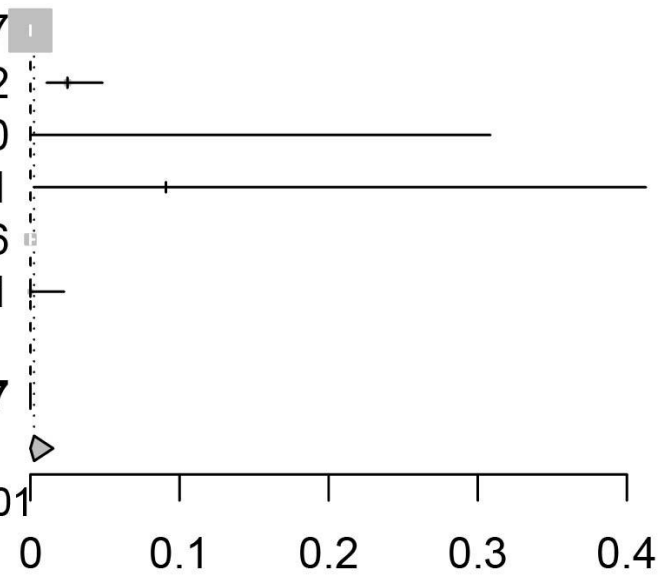

*Aedes aegypti*_Yellow fever virus

Study	Events	Total	Proportion	95% *CI*	Weight (common)	Weight (random)
Agwu et al. (2016)	6	200	0.030000	[0.011087–0.064151]	0.2%	10.6%
Agwu et al. (2019)	223	24157	0.009231	[0.008064–0.010519]	91.9%	15.3%
Diallo et al. (2020)	0	236	0.000000	[0.000000–0.015509]	3.9%	14.9%
Faye et al. (2007)	16	672	0.023810	[0.013669–0.038378]	1.0%	13.8%
Fontenille et al. (1997)	21	345	0.060870	[0.038070–0.091543]	0.2%	10.1%
Germain et al. (1980)	2	64	0.031250	[0.003807–0.108371]	0.1%	6.2%
Guedes et al. (2017)	0	163	0.000000	[0.000000–0.022377]	1.9%	14.5%
Lee et al. (1972)	0	10	0.000000	[0.000000–0.308497]	0.0%	1.2%
Thonnon et al. (1998)	24	699	0.034335	[0.022120–0.050658]	0.7%	13.4%
**Common effect model**		**26546**	**0.009201**	**[0.008044–0.010357]**	**100.0%**	**--**
**Random effects model**			**0.020573**	**[0.006793–0.034353]**	**--**	**100.0%**

Heterogeneity: $I^2 = 85\%$, $\tau^2 = 0.0003$, $p < 0.000001$

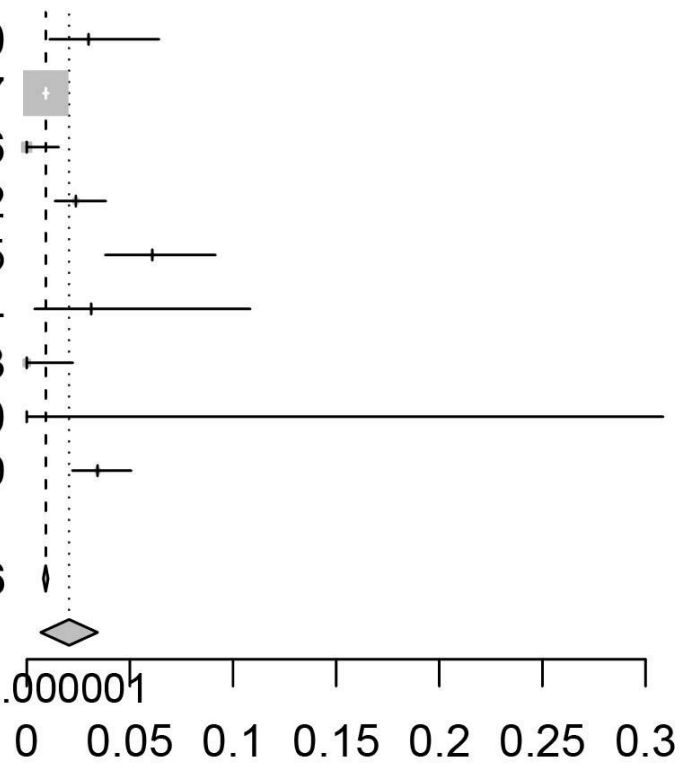

*Aedes aegypti*_Zika virus

Study	Events	Total	Proportion	95% *CI*	Weight (common)	Weight (random)
Campos et al. (2020)	2	754	0.002653	[0.000321–0.009549]	6.9%	20.1%
Diallo et al. (2014)	1	245	0.004082	[0.000103–0.022530]	1.5%	5.3%
Diallo et al. (2020)	0	236	0.000000	[0.000000–0.015509]	2.7%	9.4%
Lee et al. (1972)	0	10	0.000000	[0.000000–0.308497]	0.0%	0.0%
Tinto et al. (2022)	0	1356	0.000000	[0.000000–0.002717]	88.9%	65.1%
**Common effect model**		**2601**	**0.000242**	**[0.000000–0.001205]**	**100.0%**	**--**
**Random effects model**			**0.000751**	**[0.000000–0.002655]**	**--**	**100.0%**

Heterogeneity: $I^2 = 0\%$, $\tau^2 < 0.0001$, $p = 0.597536$

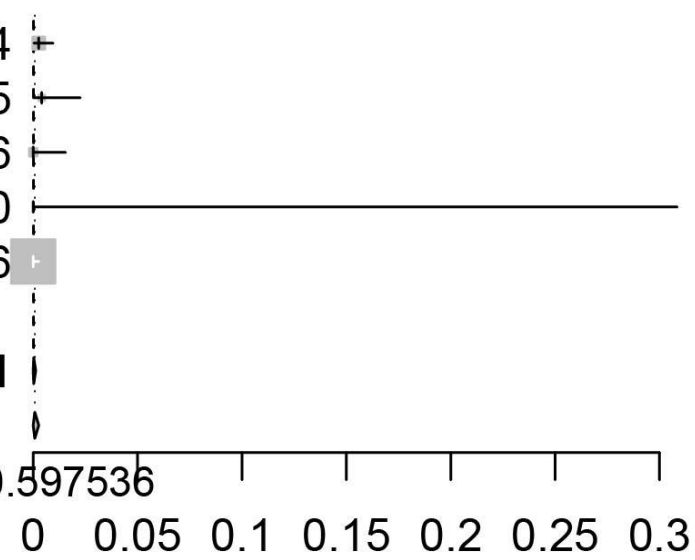

*Aedes africanus*_Chikungunya virus

Study	Events	Total	Proportion	95% *CI*	Weight (common)	Weight (random)
Diallo et al. (2012)	2	682	0.002933	[0.000355–0.010553]	1.4%	17.6%
Diallo et al. (1999)	1	4088	0.000245	[0.000006–0.001362]	98.5%	80.3%
Diallo et al. (2020)	0	106	0.000000	[0.000000–0.034202]	0.1%	2.1%
**Common effect model**		**4876**	**0.000281**	**[0.000000–0.000757]**	**100.0%**	**--**
**Random effects model**			**0.000712**	**[0.000000–0.002623]**	**--**	**100.0%**

Heterogeneity: $I^2 = 0\%$, $\tau^2 < 0.0001$, $p = 0.435215$

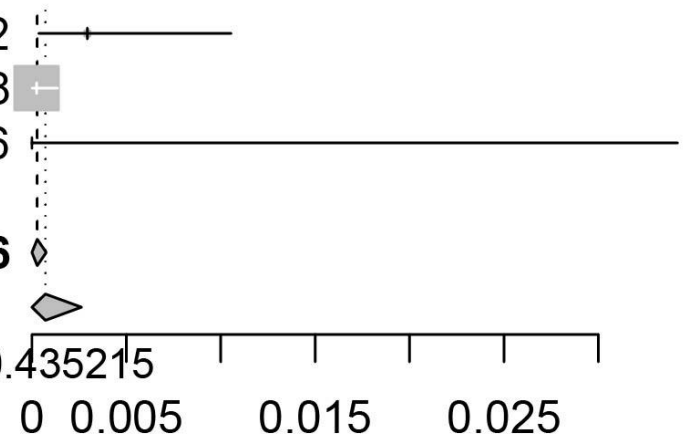

*Aedes africanus*_Zika virus

Study	Events	Total	Proportion	95% *CI*	Weight (common)	Weight (random)
Diallo et al. (2014)	5	505	0.009901	[0.003222–0.022953]	97.3%	97.3%
Diallo et al. (2020)	0	4	0.000000	[0.000000–0.602365]	0.8%	0.8%
Lee et al. (1972)	0	10	0.000000	[0.000000–0.308497]	1.9%	1.9%
**Common effect model**		**519**	**0.009376**	**[0.002909–0.019471]**	**100.0%**	**--**
**Random effects model**			**0.009376**	**[0.002909–0.019471]**	**--**	**100.0%**

Heterogeneity: $I^2 = 0\%$, $\tau^2 = 0$, $p = 0.762888$

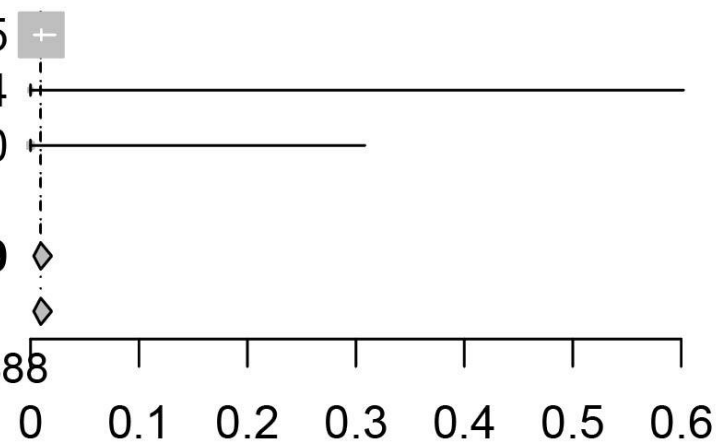

*Aedes argenteopunctatus*_Chikungunya virus

Study	Events	Total	Proportion	95% *CI*	Weight (common)	Weight (random)
Diallo et al. (1999)	1	26356	0.000038	[0.000001–0.000211]	100.0%	100.0%
Diallo et al. (2020)	0	264	0.000000	[0.000000–0.013876]	0.0%	0.0%
**Common effect model**		**26620**	**0.000038**	**[0.000000–0.000112]**	**100.0%**	**--**
**Random effects model**			**0.000038**	**[0.000000–0.000112]**	**--**	**100.0%**

Heterogeneity: $I^2 = 0\%$, $\tau^2 = 0$, $p = 0.988645$

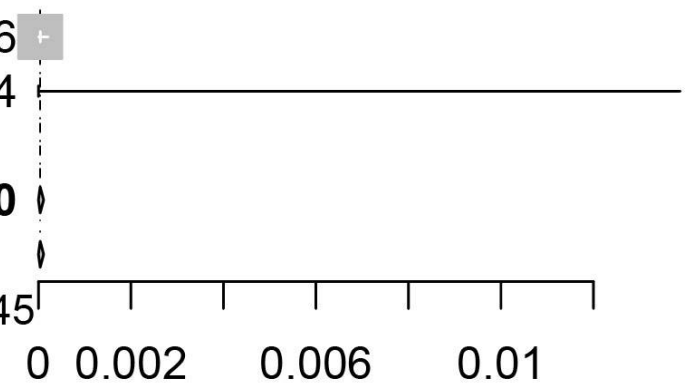

*Aedes centropunctatus*_Chikungunya virus

Study	Events	Total	Proportion	95% *CI*	Weight (common)	Weight (random)
Diallo et al. (2012)	1	68	0.014706	[0.000372–0.079234]	23.6%	23.6%
Diallo et al. (2020)	0	86	0.000000	[0.000000–0.041987]	76.4%	76.4%
**Common effect model**		**154**	**0.003465**	**[0.000000–0.017352]**	**100.0%**	**--**
**Random effects model**			**0.003465**	**[0.000000–0.017352]**	**--**	**100.0%**

Heterogeneity: $I^2 = 0\%$, $\tau^2 = 0$, $p = 0.378429$

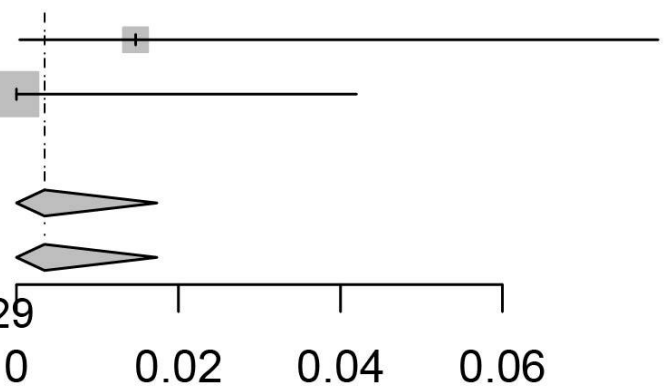

*Aedes dalzieli*_Chikungunya virus

Study	Events	Total	Proportion	95% *CI*	Weight (common)	Weight (random)
Diallo et al. (2012)	4	6219	0.000643	[0.000175–0.001646]	1.3%	11.2%
Diallo et al. (1999)	12	93330	0.000129	[0.000066–0.000225]	97.3%	76.8%
Diallo et al. (2020)	0	2286	0.000000	[0.000000–0.001612]	1.4%	12.0%
**Common effect model**		**101835**	**0.000133**	**[0.000062–0.000205]**	**100.0%**	**--**
**Random effects model**			**0.000171**	**[0.000000–0.000398]**	**--**	**100.0%**

Heterogeneity: I^2 = 26%, τ^2 < 0.0001, p = 0.256977

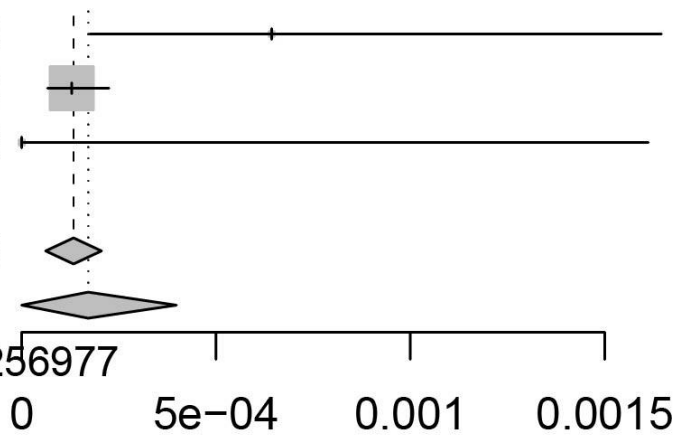

*Aedes dalzieli*_West Nile virus

Study	Events	Total	Proportion	95% *CI*	Weight (common)	Weight (random)
Ndiaye et al. (2018)	1	482	0.002075	[0.000053–0.011505]	90.2%	90.2%
Wilson et al. (1994)	0	111	0.000000	[0.000000–0.032687]	9.8%	9.8%
**Common effect model**		**593**	**0.001872**	**[0.000000–0.005731]**	**100.0%**	**--**
**Random effects model**			**0.001872**	**[0.000000–0.005731]**	**--**	**100.0%**

Heterogeneity: I^2 = 0%, τ^2 = 0, p = 0.754393

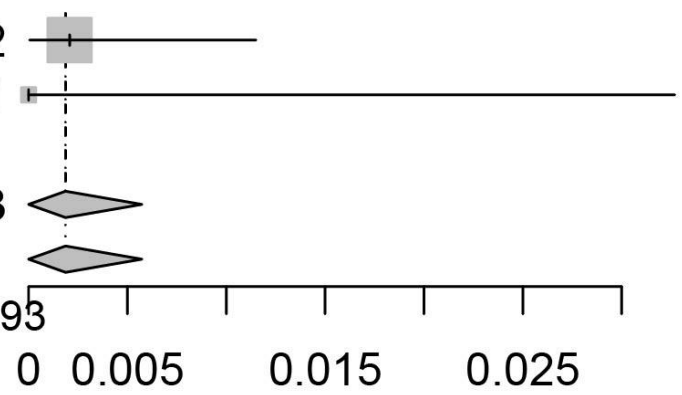

*Aedes dalzieli*_Zika virus

Study	Events	Total	Proportion	95% *CI*	Weight (common)	Weight (random)
Diallo et al. (2014)	2	1718	0.001164	[0.000141–0.004199]	22.0%	22.0%
Diallo et al. (2020)	1	2286	0.000437	[0.000011–0.002435]	78.0%	78.0%
**Common effect model**		**4004**	**0.000598**	**[0.000000–0.001354]**	**100.0%**	**--**
**Random effects model**			**0.000598**	**[0.000000–0.001354]**	**--**	**100.0%**

Heterogeneity: I^2 = 0%, τ^2 = 0, p = 0.435418

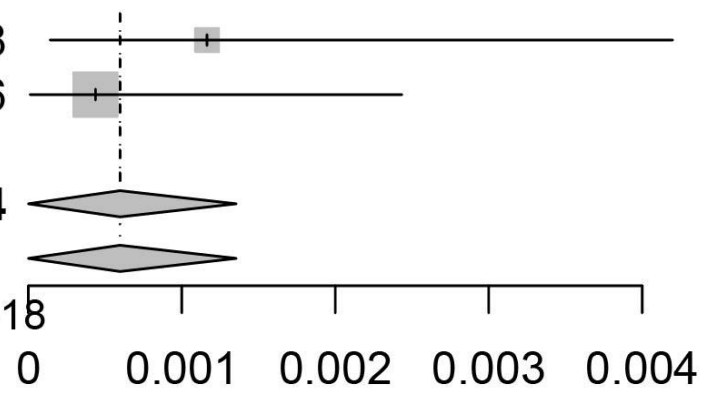

*Aedes fowleri*_Bagaza virus

Study	Events	Total	Proportion	95% *CI*	Weight (common)	Weight (random)
Diallo et al. (2005)	2	576	0.003472	[0.000421–0.012486]	72.5%	59.9%
Ndiaye et al. (2018)	0	193	0.000000	[0.000000–0.018932]	24.3%	33.7%
Wilson et al. (1994)	0	26	0.000000	[0.000000–0.132275]	3.3%	6.4%
**Common effect model**		**795**	**0.001824**	**[0.000063–0.005990]**	**100.0%**	**--**
**Random effects model**			**0.001245**	**[0.000000–0.007340]**	**--**	**100.0%**

Heterogeneity: I^2 = 9%, τ^2 = 0.0007, p = 0.331819

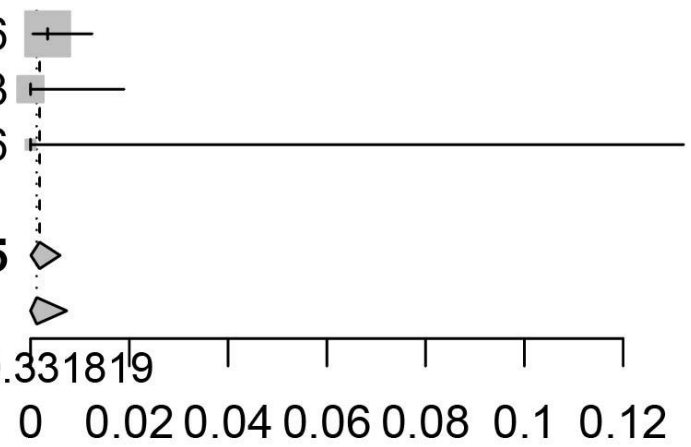

*Aedes fowleri*_Rift Valley fever virus

Study	Events	Total	Proportion	95% *CI*	Weight (common)	Weight (random)
Diallo et al. (2005)	0	576	0.000000	[0.000000–0.006384]	70.5%	70.5%
Ba et al. (2012)	1	1	1.000000	[0.025000–1.000000]	0.1%	0.1%
Fontenille et al. (1995)	0	21	0.000000	[0.000000–0.161098]	2.6%	2.6%
Ndiaye et al. (2018)	0	193	0.000000	[0.000000–0.018932]	23.6%	23.6%
Wilson et al. (1994)	0	26	0.000000	[0.000000–0.132275]	3.2%	3.2%
**Common effect model**		**817**	**0.000004**	**[0.000000–0.001310]**	**100.0%**	**--**
**Random effects model**			**0.000004**	**[0.000000–0.001311]**	**--**	**100.0%**

Heterogeneity: I^2 = 59%, τ^2 < 0.0001, p = 0.042897

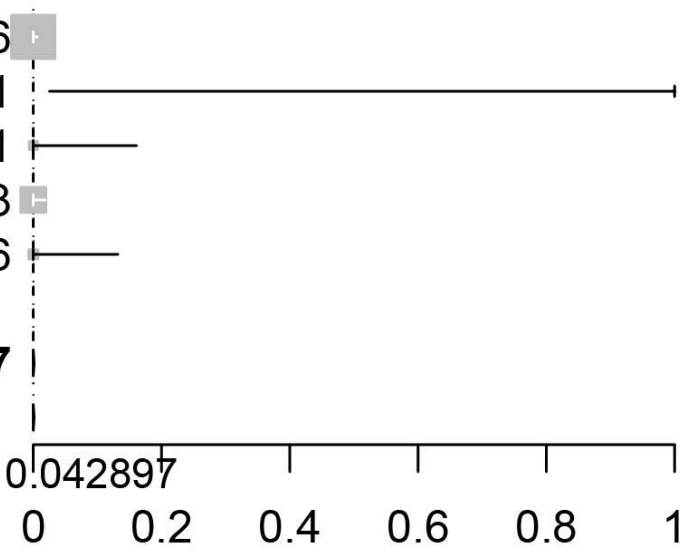

*Aedes furcifer*_Chikungunya virus

Study	Events	Total	Proportion	95% *CI*	Weight (common)	Weight (random)
Diallo et al. (2012)	15	7427	0.002020	[0.001131–0.003329]	1.1%	35.3%
Diallo et al. (1999)	63	143460	0.000439	[0.000337–0.000562]	98.8%	36.5%
Diallo et al. (2020)	18	2823	0.006376	[0.003783–0.010058]	0.1%	28.2%
**Common effect model**		**153710**	**0.000465**	**[0.000357–0.000572]**	**100.0%**	**--**
**Random effects model**			**0.002671**	**[0.000000–0.005933]**	**--**	**100.0%**

Heterogeneity: I^2 = 92%, τ^2 < 0.0001, p = 0.000004

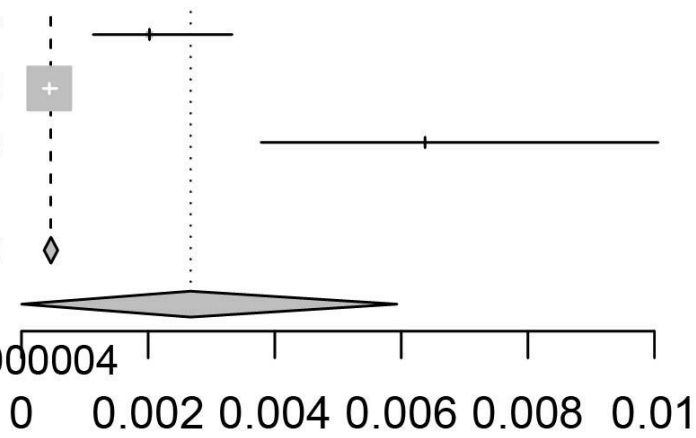

*Aedes furcifer*_Yellow fever virus

Study	Events	Total	Proportion	95% *CI*	Weight (common)	Weight (random)
Diallo et al. (2020)	0	8	0.000000	[0.000000–0.369417]	1.0%	1.0%
Faye et al. (2007)	33	739	0.044655	[0.030935–0.062143]	96.3%	96.3%
Fontenille et al. (1997)	2	21	0.095238	[0.011749–0.303774]	1.4%	1.4%
Thonnon et al. (1998)	2	21	0.095238	[0.011749–0.303774]	1.4%	1.4%
**Common effect model**		**789**	**0.045600**	**[0.030984–0.060216]**	**100.0%**	**--**
**Random effects model**			**0.045600**	**[0.030984–0.060216]**	**--**	**100.0%**

Heterogeneity: I^2 = 0%, τ^2 = 0, p = 0.665500

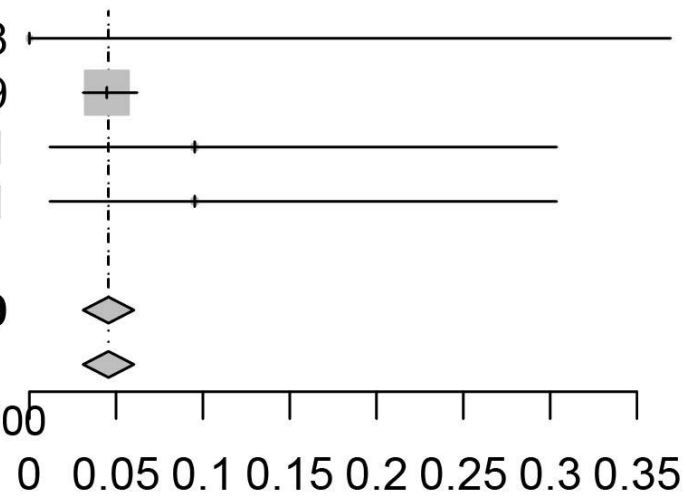

*Aedes furcifer*_Zika virus

Study	Events	Total	Proportion	95% *CI*	Weight (common)	Weight (random)
Diallo et al. (2014)	5	2939	0.001701	[0.000553–0.003966]	66.1%	62.4%
Diallo et al. (2020)	9	2823	0.003188	[0.001459–0.006043]	33.9%	37.6%
**Common effect model**		**5762**	**0.002206**	**[0.000994–0.003417]**	**100.0%**	**--**
**Random effects model**			**0.002260**	**[0.000849–0.003672]**	**--**	**100.0%**

Heterogeneity: I^2 = 23%, τ^2 < 0.0001, p = 0.254642

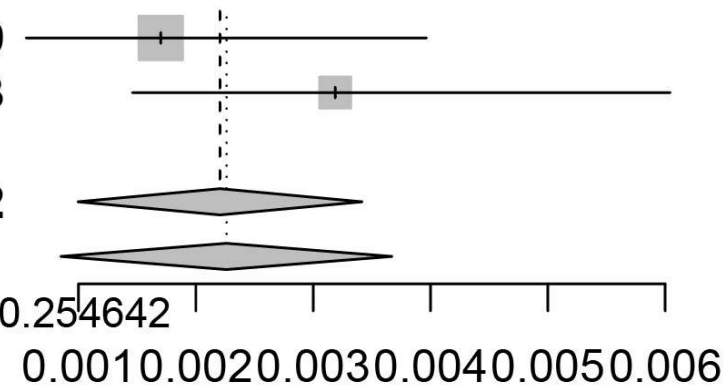

*Aedes hirsutus*_Chikungunya virus

Study	Events	Total	Proportion	95% *CI*	Weight (common)	Weight (random)
Diallo et al. (2012)	1	91	0.010989	[0.000278–0.059711]	66.2%	66.2%
Diallo et al. (2020)	0	45	0.000000	[0.000000–0.078705]	33.8%	33.8%
**Common effect model**		**136**	**0.007273**	**[0.000000–0.024698]**	**100.0%**	**--**
**Random effects model**			**0.007273**	**[0.000000–0.024698]**	**--**	**100.0%**

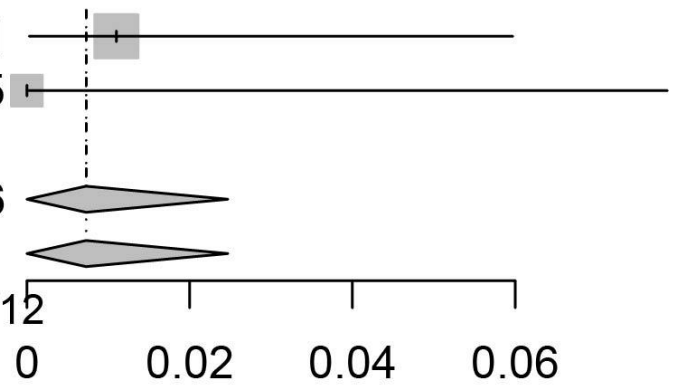

Heterogeneity: I^2 = 0%, τ^2 = 0, p = 0.558712

*Aedes hirsutus*_Zika virus

Study	Events	Total	Proportion	95% *CI*	Weight (common)	Weight (random)
Diallo et al. (2014)	2	34	0.058824	[0.007205–0.196773]	12.6%	29.8%
Diallo et al. (2020)	0	45	0.000000	[0.000000–0.078705]	87.4%	70.2%
**Common effect model**		**79**	**0.007384**	**[0.000000–0.035404]**	**100.0%**	**--**
**Random effects model**			**0.017558**	**[0.000000–0.070314]**	**--**	**100.0%**

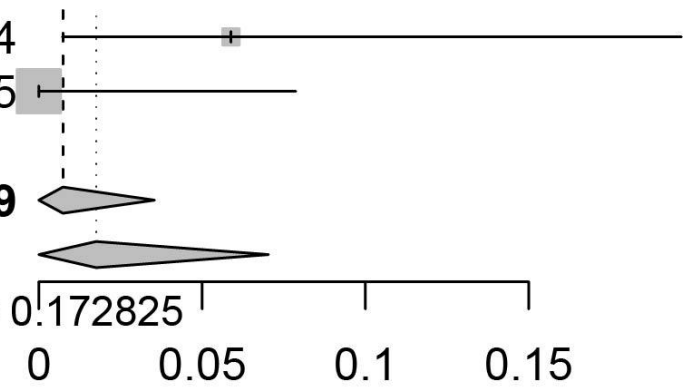

Heterogeneity: I^2 = 46%, τ^2 = 0.0008, p = 0.172825

*Aedes luteocephalus*_Chikungunya virus

Study	Events	Total	Proportion	95% *CI*	Weight (common)	Weight (random)
Diallo et al. (2012)	5	5194	0.000963	[0.000313–0.002245]	1.4%	40.6%
Diallo et al. (1999)	27	100469	0.000269	[0.000177–0.000391]	98.5%	48.8%
Diallo et al. (2020)	4	1107	0.003613	[0.000985–0.009226]	0.1%	10.6%
**Common effect model**		**106770**	**0.000281**	**[0.000181–0.000382]**	**100.0%**	**--**
**Random effects model**			**0.000906**	**[0.000000–0.002208]**	**--**	**100.0%**

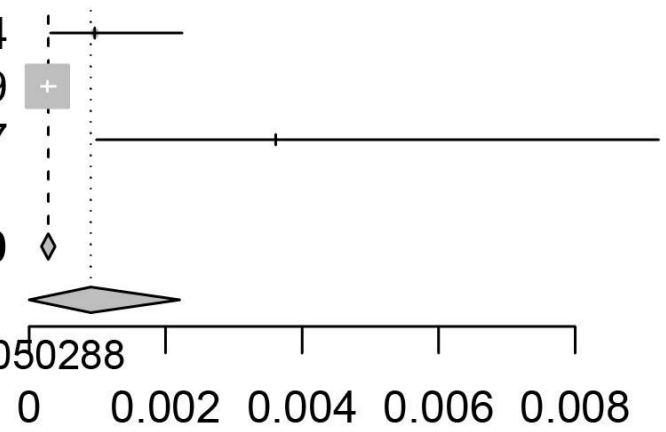

Heterogeneity: I^2 = 67%, τ^2 < 0.0001, p = 0.050288

*Aedes luteocephalus*_Dengue virus

Study	Events	Total	Proportion	95% *CI*	Weight (common)	Weight (random)
Agwu et al. (2019)	0	5583	0.000000	[0.000000–0.000661]	76.6%	38.0%
Lee et al. (1972)	0	354	0.000000	[0.000000–0.010366]	4.9%	27.0%
Traore−Lamizana et al. (1994)	7	1353	0.005174	[0.002083–0.010631]	18.6%	35.0%
**Common effect model**		**7290**	**0.000179**	**[0.000004–0.000617]**	**100.0%**	**--**
**Random effects model**			**0.000634**	**[0.000000–0.005437]**	**--**	**100.0%**

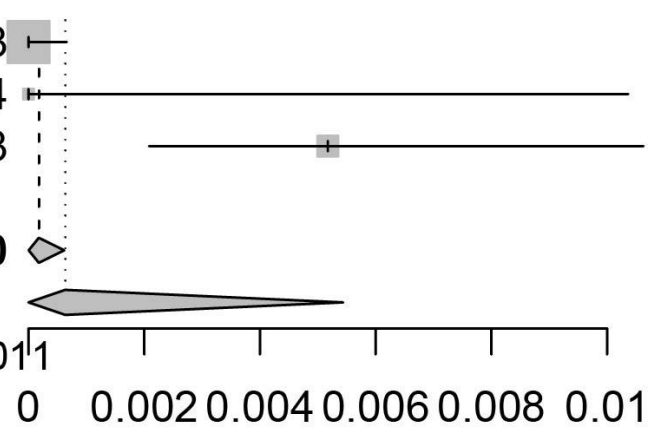

Heterogeneity: I^2 = 91%, τ^2 = 0.0016, p = 0.000011

*Aedes luteocephalus*_Yellow fever virus

Study	Events	Total	Proportion	95% *CI*	Weight (common)	Weight (random)
Agwu et al. (2016)	0	200	0.000000	[0.000000–0.018275]	3.2%	17.5%
Agwu et al. (2019)	75	5583	0.013434	[0.010581–0.016810]	88.5%	17.6%
Diallo et al. (2020)	0	7	0.000000	[0.000000–0.409616]	0.1%	14.8%
Faye et al. (2007)	0	160	0.000000	[0.000000–0.022792]	2.5%	17.5%
Fontenille et al. (1997)	1	1	1.000000	[0.025000–1.000000]	0.0%	7.5%
Lee et al. (1972)	0	354	0.000000	[0.000000–0.010366]	5.6%	17.6%
Thonnon et al. (1998)	1	1	1.000000	[0.025000–1.000000]	0.0%	7.5%
**Common effect model**		**6306**	**0.010642**	**[0.008259–0.013323]**	**100.0%**	**--**
**Random effects model**			**0.064331**	**[0.000000–0.329043]**	**--**	**100.0%**

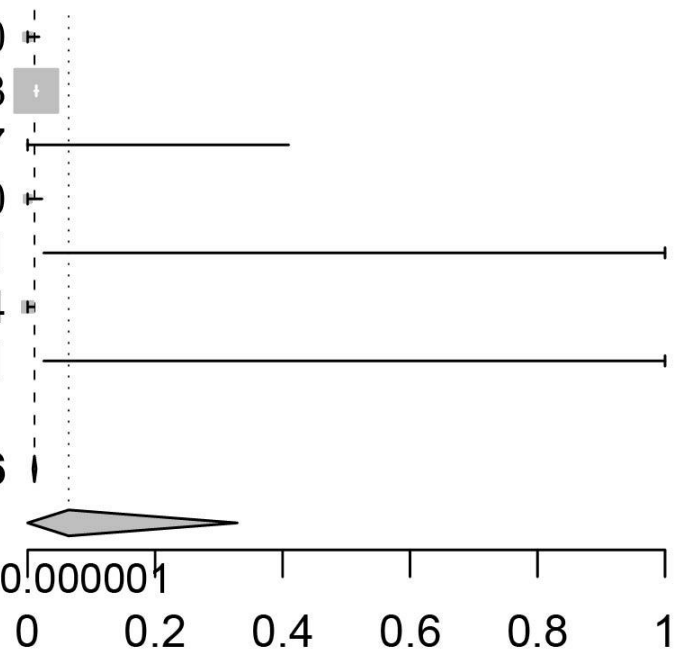

Heterogeneity: I^2 = 88%, τ^2 = 0.1855, p < 0.000001

*Aedes luteocephalus*_Zika virus

Study	Events	Total	Proportion	95% *CI*	Weight (common)	Weight (random)
Diallo et al. (2014)	5	1259	0.003971	[0.001291–0.009243]	64.5%	48.8%
Diallo et al. (2020)	11	1107	0.009937	[0.004971–0.017710]	22.8%	30.4%
Lee et al. (1972)	2	354	0.005650	[0.000685–0.020259]	12.8%	20.8%
**Common effect model**		**2720**	**0.005545**	**[0.002756–0.008334]**	**100.0%**	**--**
**Random effects model**			**0.006132**	**[0.001922–0.010342]**	**--**	**100.0%**

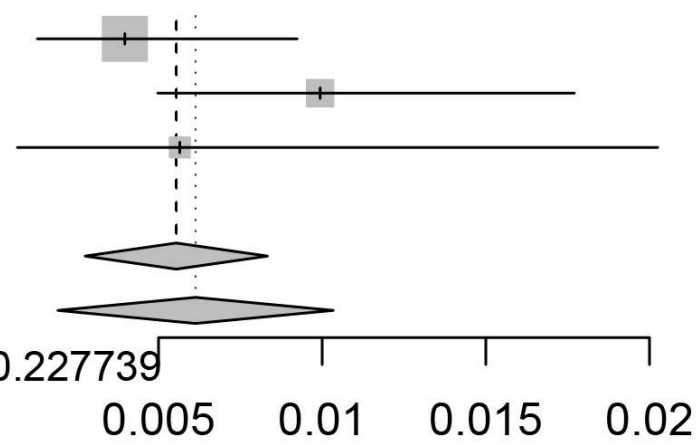

Heterogeneity: I^2 = 32%, τ^2 < 0.0001, p = 0.227739

*Aedes metallicus*_Chikungunya virus

Study	Events	Total	Proportion	95% *CI*	Weight (common)	Weight (random)
Diallo et al. (2012)	1	186	0.005376	[0.000136–0.029590]	85.1%	85.1%
Diallo et al. (2020)	0	54	0.000000	[0.000000–0.066032]	14.9%	14.9%
**Common effect model**		**240**	**0.004574**	**[0.000000–0.014266]**	**100.0%**	**--**
**Random effects model**			**0.004574**	**[0.000000–0.014266]**	**--**	**100.0%**

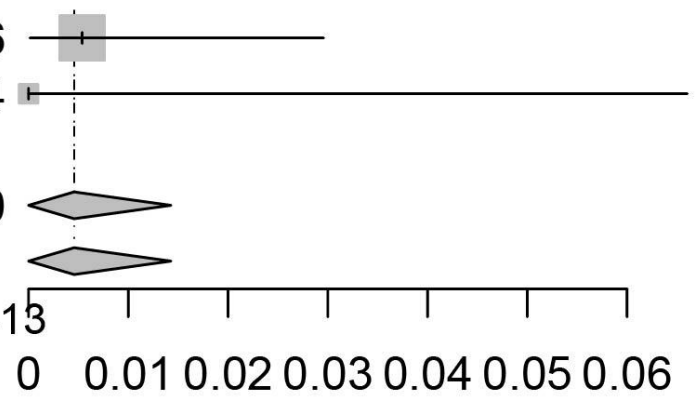

Heterogeneity: I^2 = 0%, τ^2 = 0, p = 0.698413

*Aedes metallicus*_Yellow fever virus

Study	Events	Total	Proportion	95% *CI*	Weight (common)	Weight (random)
Diallo et al. (2020)	0	54	0.000000	[0.000000–0.066032]	90.0%	29.5%
Faye et al. (2007)	0	4	0.000000	[0.000000–0.602365]	6.7%	27.2%
Fontenille et al. (1997)	1	1	1.000000	[0.025000–1.000000]	1.7%	21.7%
Thonnon et al. (1998)	1	1	1.000000	[0.025000–1.000000]	1.7%	21.7%
**Common effect model**		**60**	**0.002739**	**[0.000000–0.031656]**	**100.0%**	**--**
**Random effects model**			**0.395729**	**[0.000000–0.999791]**	**--**	**100.0%**

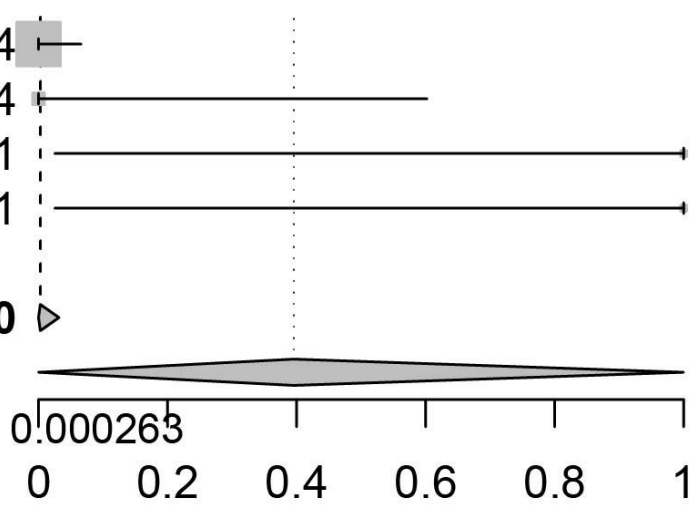

Heterogeneity: I^2 = 84%, τ^2 = 0.6724, p = 0.000263

*Aedes metallicus*_Zika virus

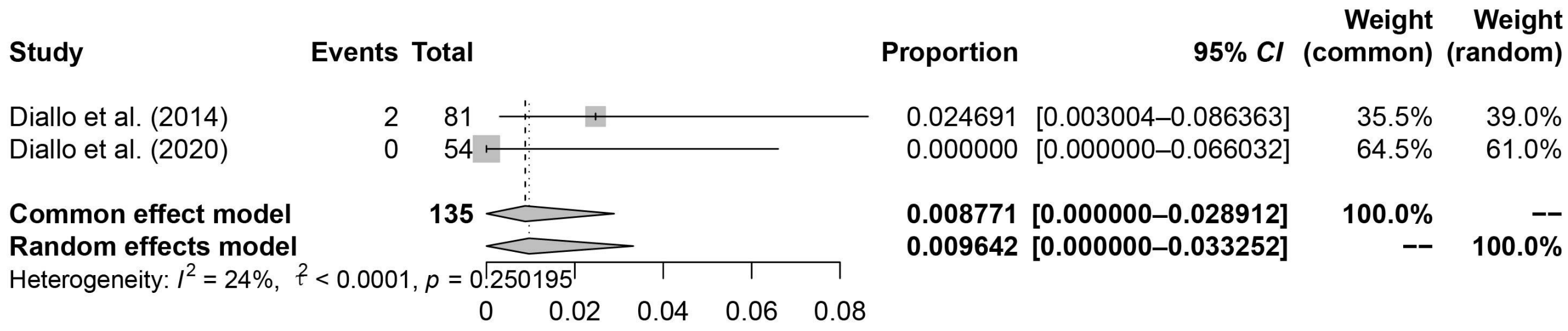

*Aedes neoafricanus*_Chikungunya virus

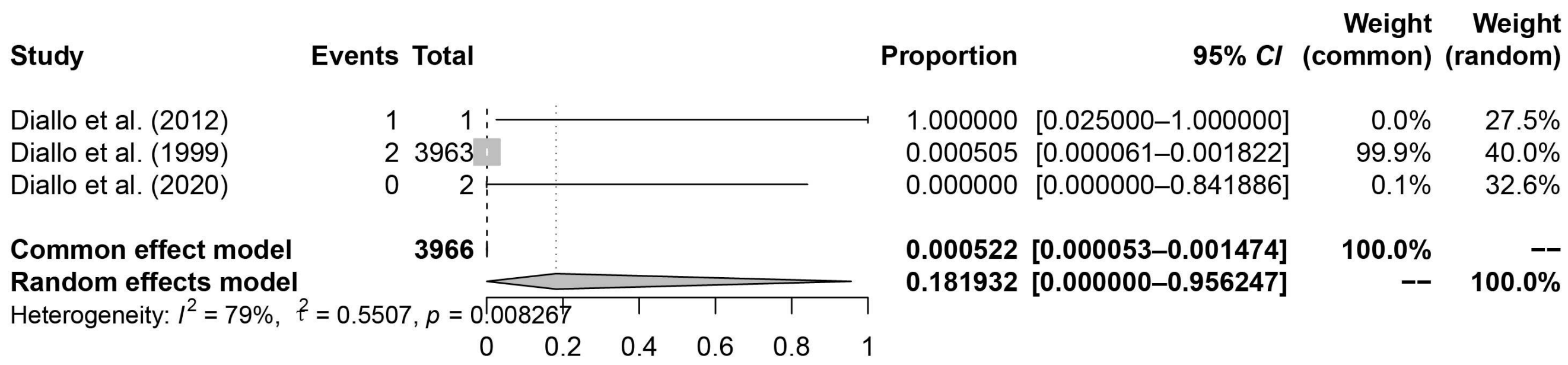

*Aedes ochraceus*_Rift valley fever virus

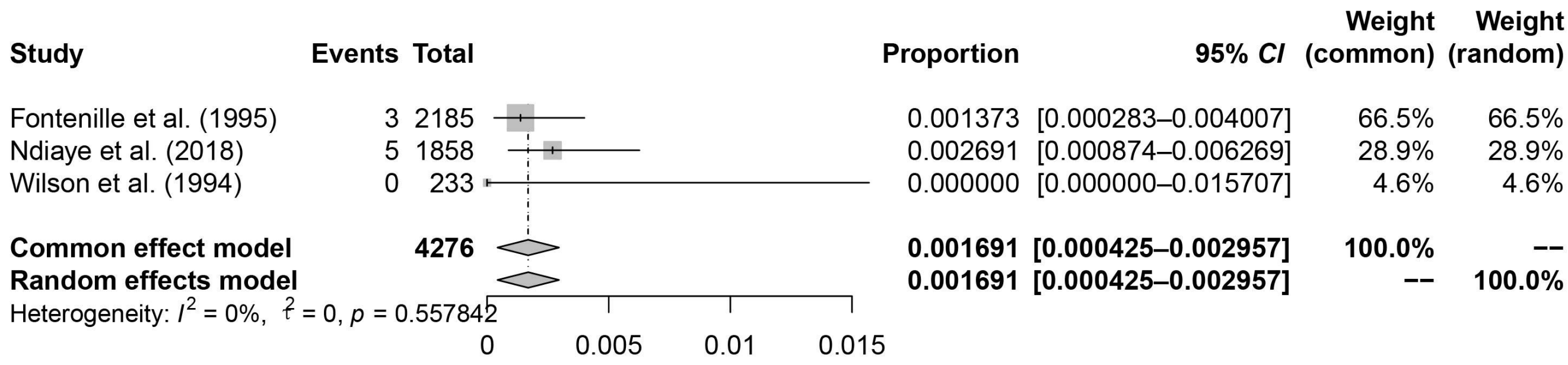

*Aedes taylori*_Chikungunya virus

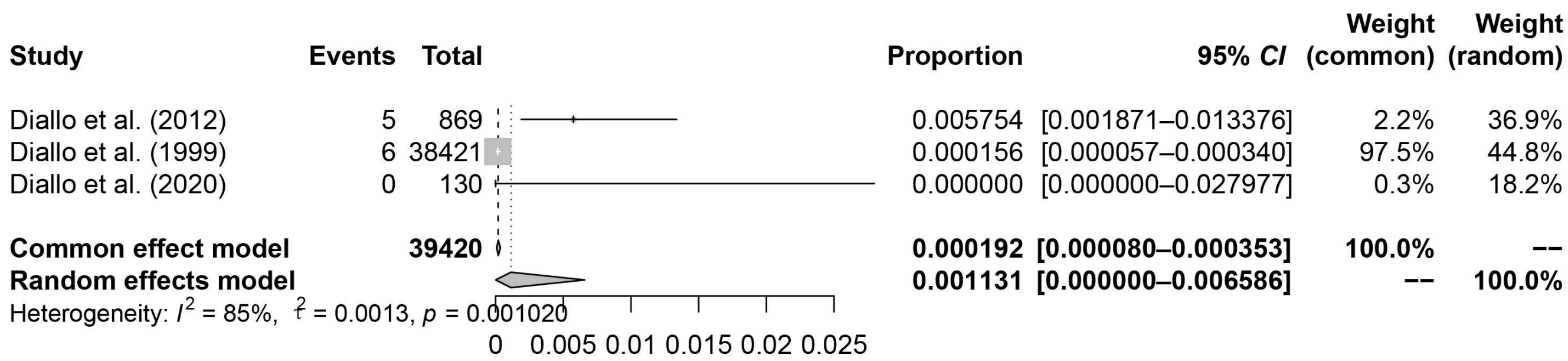

*Aedes taylori*_Zika virus

Study	Events	Total	Proportion	95% *CI*	Weight (common)	Weight (random)
Diallo et al. (2014)	2	395	0.005063	[0.000614–0.018170]	100.0%	54.7%
Diallo et al. (2020)	1	1	1.000000	[0.025000–1.000000]	0.0%	45.3%
**Common effect model**		**396**	**0.005199**	**[0.000000–0.012198]**	**100.0%**	**--**
**Random effects model**			**0.455425**	**[0.000000–1.000000]**	**--**	**100.0%**

Heterogeneity: $I^2 = 91\%$, $\tau^2 = 0.4481$, $p = 0.001157$

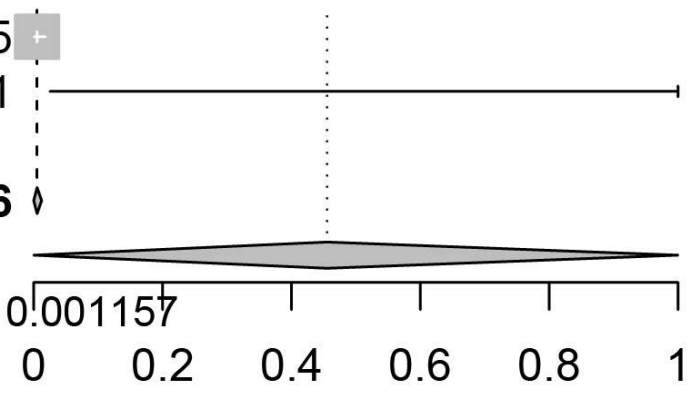

*Aedes unilineatus*_Zika virus

Study	Events	Total	Proportion	95% *CI*	Weight (common)	Weight (random)
Diallo et al. (2014)	1	38	0.026316	[0.000666–0.138099]	22.7%	22.7%
Diallo et al. (2020)	0	49	0.000000	[0.000000–0.072519]	77.3%	77.3%
**Common effect model**		**87**	**0.005973**	**[0.000000–0.030221]**	**100.0%**	**--**
**Random effects model**			**0.005973**	**[0.000000–0.030221]**	**--**	**100.0%**

Heterogeneity: $I^2 = 0\%$, $\tau^2 = 0$, $p = 0.372922$

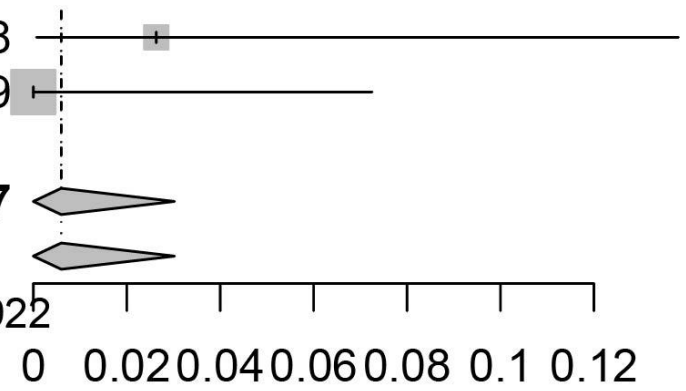

*Aedes vexans*_Rift Valley fever virus

Study	Events	Total	Proportion	95% *CI*	Weight (common)	Weight (random)
Diallo et al. (2005)	0	25740	0.000000	[0.000000–0.000143]	23.1%	23.1%
Ba et al. (2012)	10	56991	0.000175	[0.000084–0.000323]	51.1%	24.3%
Fontenille et al. (1995)	10	18578	0.000538	[0.000258–0.000990]	16.7%	22.3%
Ndiaye et al. (2018)	0	8293	0.000000	[0.000000–0.000445]	7.4%	19.4%
Stoek et al. (2022)	0	2	0.000000	[0.000000–0.841886]	0.0%	0.0%
Wilson et al. (1994)	0	1941	0.000000	[0.000000–0.001899]	1.7%	10.9%
**Common effect model**		**111545**	**0.000113**	**[0.000059–0.000184]**	**100.0%**	**--**
**Random effects model**			**0.000070**	**[0.000000–0.000329]**	**--**	**100.0%**

Heterogeneity: $I^2 = 83\%$, $\tau^2 < 0.0001$, $p = 0.000018$

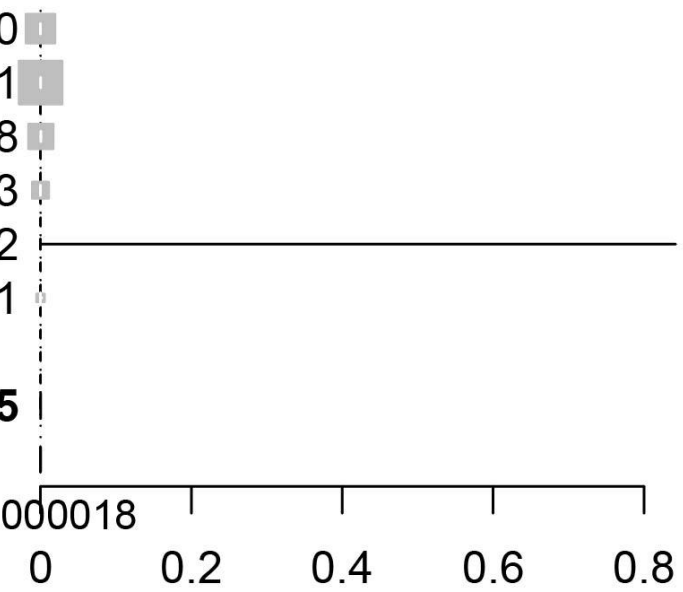

*Aedes vexans*_Wesselsbron virus

Study	Events	Total	Proportion	95% *CI*	Weight (common)	Weight (random)
Diallo et al. (2005)	51	25730	0.001982	[0.001476–0.002605]	63.3%	50.7%
Wilson et al. (1994)	0	1941	0.000000	[0.000000–0.001899]	36.7%	49.3%
**Common effect model**		**27671**	**0.001254**	**[0.000822–0.001687]**	**100.0%**	**--**
**Random effects model**			**0.001005**	**[0.000000–0.002947]**	**--**	**100.0%**

Heterogeneity: $I^2 = 95\%$, $\tau^2 < 0.0001$, $p = 0.000015$

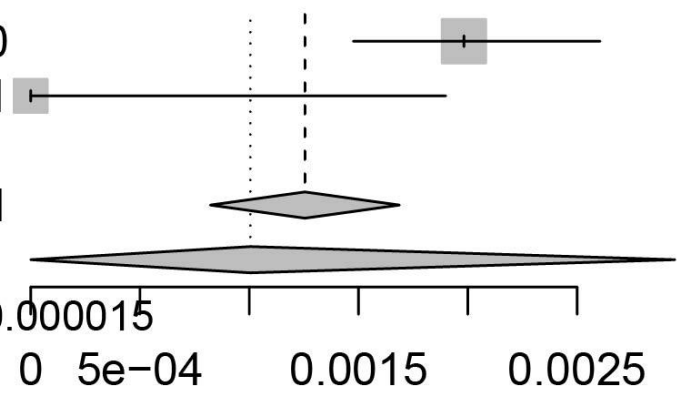

*Aedes vexans*_West Nile virus

Study	Events	Total	Proportion	95% *CI*	Weight (common)	Weight (random)
Diallo et al. (2005)	0	10	0.000000	[0.000000–0.308497]	0.1%	1.0%
Ndiaye et al. (2018)	0	8293	0.000000	[0.000000–0.000445]	81.0%	54.4%
Wilson et al. (1994)	2	1941	0.001030	[0.000125–0.003717]	18.9%	44.6%
**Common effect model**		**10244**	**0.000037**	**[0.000000–0.000249]**	**100.0%**	**--**
**Random effects model**			**0.000205**	**[0.000000–0.002025]**	**--**	**100.0%**

Heterogeneity: I^2 = 69%, τ^2 = 0.0004, p = 0.039038

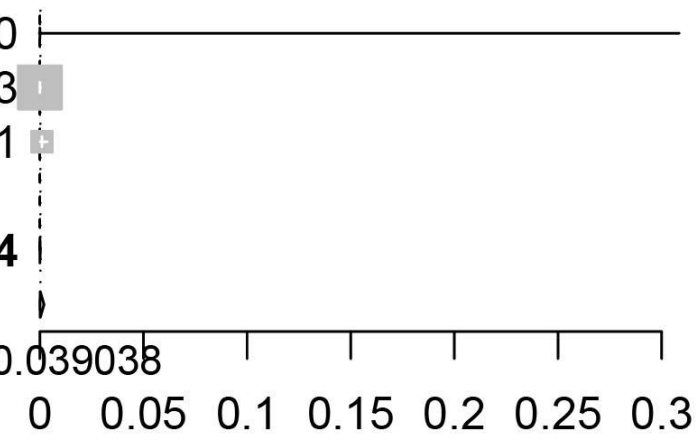

*Aedes vittatus*_Chikungunya virus

Study	Events	Total	Proportion	95% *CI*	Weight (common)	Weight (random)
Diallo et al. (2012)	0	9147	0.000000	[0.000000–0.000403]	11.6%	22.3%
Diallo et al. (1999)	3	67682	0.000044	[0.000009–0.000130]	85.8%	71.8%
Diallo et al. (2020)	0	2034	0.000000	[0.000000–0.001812]	2.6%	5.9%
**Common effect model**		**78863**	**0.000033**	**[0.000005–0.000085]**	**100.0%**	**--**
**Random effects model**			**0.000023**	**[0.000000–0.000104]**	**--**	**100.0%**

Heterogeneity: I^2 = 0%, τ^2 < 0.0001, p = 0.427125

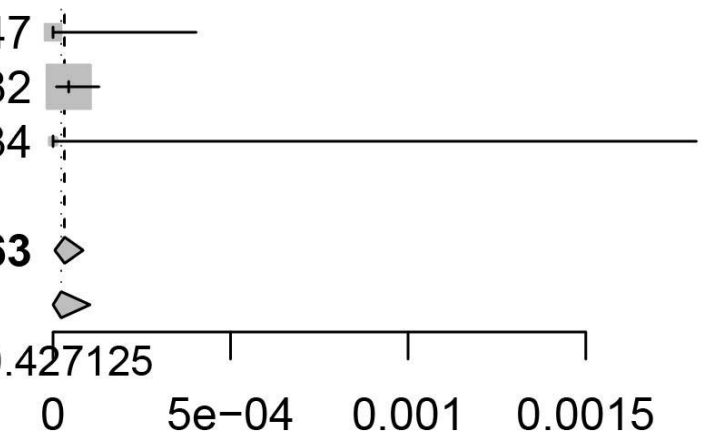

*Aedes vittatus*_Zika virus

Study	Events	Total	Proportion	95% *CI*	Weight (common)	Weight (random)
Diallo et al. (2014)	3	1728	0.001736	[0.000358–0.005065]	99.7%	99.7%
Diallo et al. (2020)	0	4	0.000000	[0.000000–0.602365]	0.2%	0.2%
Lee et al. (1972)	0	1	0.000000	[0.000000–0.975000]	0.1%	0.1%
**Common effect model**		**1733**	**0.001726**	**[0.000325–0.004232]**	**100.0%**	**--**
**Random effects model**			**0.001726**	**[0.000325–0.004232]**	**--**	**100.0%**

Heterogeneity: I^2 = 0%, τ^2 = 0, p = 0.982828

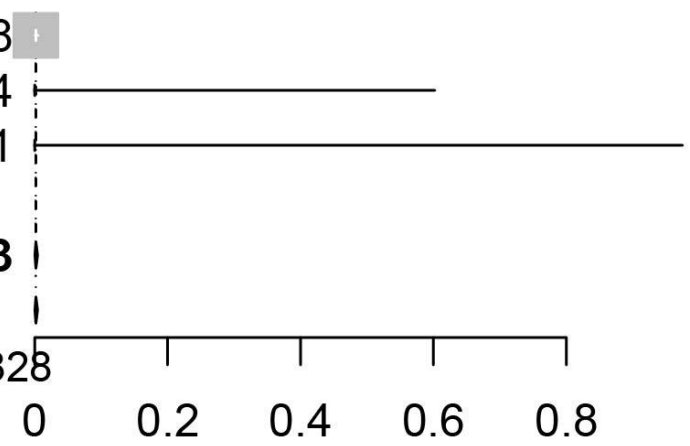

Supplementary Fig. 10. Meta-analysis of the positivity rate of each *Anopheles*-associated pathogen. The heterogeneity of combined studies was quantified by I^2 statistic. The random effect model would be applied if $I^2 > 50\%$; Otherwise, the fixed effect model would be applied.

***Anopheles*_Chikungunya virus**

Study	Events	Total	Proportion	95% *CI*	Weight (common)	Weight (random)
Diallo et al. (2012)	3	1761	0.001704	[0.000351–0.004970]	0.1%	32.8%
Diallo et al. (1999)	2	53084	0.000038	[0.000005–0.000136]	99.9%	53.7%
Diallo et al. (2020)	3	814	0.003686	[0.000761–0.010733]	0.0%	13.5%
**Common effect model**		**55659**	**0.000039**	**[0.000000–0.000092]**	**100.0%**	**--**
**Random effects model**			**0.001078**	**[0.000000–0.002848]**	**--**	**100.0%**

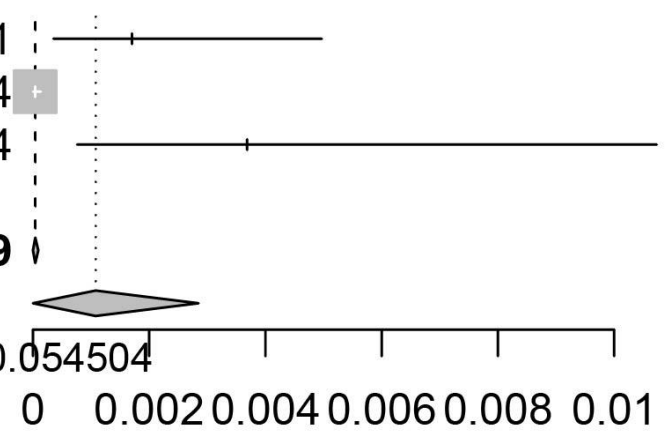

Heterogeneity: $I^2 = 66\%$, $\tau^2 < 0.0001$, $p = 0.054504$

Anopheles_Plasmodium falciparum

Study	Events	Total	Proportion	95% *CI*	Weight (common)	Weight (random)
Adja et al. (2022)	127	3668	0.034624	[0.028944–0.041059]	1.0%	1.2%
Akogb..to et al. (2015)	157	3462	0.045350	[0.038661–0.052819]	0.9%	1.2%
Appawu et al. (2004)	564	8112	0.069527	[0.064085–0.075279]	2.1%	1.2%
Appawu et al. (2003)	284	2545	0.111591	[0.099614–0.124470]	0.7%	1.2%
Assouho et al. (2020)	229	4045	0.056613	[0.049690–0.064185]	1.1%	1.2%
Awolola et al. (2005)	19	1637	0.011607	[0.007002–0.018066]	0.4%	1.2%
Awolola et al. (2014)	48	2015	0.023821	[0.017615–0.031461]	0.5%	1.2%
Awolola et al. (2002)	17	1002	0.016966	[0.009914–0.027026]	0.3%	1.2%
Basing et al. (2014)	975	2022	0.482196	[0.460208–0.504235]	0.5%	1.2%
Bimbile Somdal et al. (2018)	0	506	0.000000	[0.000000–0.007264]	0.1%	1.2%
Boudin et al. (1991)	70	1118	0.062612	[0.049131–0.078447]	0.3%	1.2%
Carnevale et al. (2010)	35	140	0.250000	[0.180724–0.330160]	0.0%	1.1%
Carrara et al. (1990)	5	1010	0.004950	[0.001609–0.011515]	0.3%	1.2%
Collins et al. (2019)	30	968	0.030992	[0.021006–0.043949]	0.3%	1.2%
Coulibaly et al. (2022)	121	7684	0.015747	[0.013083–0.018787]	2.0%	1.2%
Czeher et al. (2006)	90	2092	0.043021	[0.034733–0.052618]	0.5%	1.2%
Dabire et al. (2008)	1	84	0.011905	[0.000301–0.064552]	0.0%	1.0%
Dabire et al. (2012)	29	658	0.044073	[0.029712–0.062685]	0.2%	1.2%
Dabir.. et al. (2008)	118	1097	0.107566	[0.089847–0.127420]	0.3%	1.2%
Dia et al. (2008)	5	4067	0.001229	[0.000399–0.002867]	1.1%	1.2%
Dia et al. (2003)	45	1741	0.025847	[0.018914–0.034434]	0.5%	1.2%
Dia et al. (2005)	54	2592	0.020833	[0.015688–0.027096]	0.7%	1.2%
Diakit.. et al. (2015)	130	8018	0.016214	[0.013564–0.019223]	2.1%	1.2%
Djouaka et al. (2016)	66	549	0.120219	[0.094209–0.150396]	0.1%	1.2%
Dj..nontin et al. (2021)	71	3848	0.018451	[0.014438–0.023217]	1.0%	1.2%
Ebenezer et al. (2016)	67	617	0.108590	[0.085152–0.135849]	0.2%	1.2%
Epopa et al. (2019)	152	3891	0.039065	[0.033197–0.045637]	1.0%	1.2%
Fagbohun et al. (2020)	0	5117	0.000000	[0.000000–0.000721]	1.3%	1.2%
Fofana et al. (2020)	0	39	0.000000	[0.000000–0.090251]	0.0%	0.9%
Fontenille et al. (1997)	165	13258	0.012445	[0.010628–0.014481]	3.5%	1.3%
Forson et al. (2022)	4	620	0.006452	[0.001761–0.016436]	0.2%	1.2%
Fournet et al. (2010)	6	74	0.081081	[0.030338–0.168171]	0.0%	1.0%
Fournet et al. (2022)	12	2142	0.005602	[0.002898–0.009766]	0.6%	1.2%
Fryxell et al. (2012)	78	671	0.116244	[0.092979–0.142945]	0.2%	1.2%
Gadiaga et al. (2011)	54	44628	0.001210	[0.000909–0.001578]	11.6%	1.3%
Girod et al. (2006)	4	1061	0.003770	[0.001028–0.009624]	0.3%	1.2%
Gnanguenon et al. (2014)	80	1652	0.048426	[0.038583–0.059911]	0.4%	1.2%
Gomes et al. (2017)	135	7068	0.019100	[0.016038–0.022568]	1.8%	1.2%
Govoetchan et al. (2014)	9	286	0.031469	[0.014488–0.058895]	0.1%	1.2%
Hanemaaijer et al. (2019)	18	95	0.189474	[0.116335–0.282819]	0.0%	1.1%
Hogg et al. (1996)	6	801	0.007491	[0.002754–0.016232]	0.2%	1.2%
Horton et al. (2010)	134	1961	0.068332	[0.057562–0.080414]	0.5%	1.2%
Ibrahim et al. (2019)	13	93	0.139785	[0.076582–0.227169]	0.0%	1.1%
Jaenson et al. (1994)	139	1956	0.071063	[0.060071–0.083361]	0.5%	1.2%
Ke?ta et al. (2021)	92	7782	0.011822	[0.009541–0.014479]	2.0%	1.2%
Kouassi et al. (2023)	103	4199	0.024530	[0.020065–0.029671]	1.1%	1.2%
Labbo et al. (2010)	8	2169	0.003688	[0.001594–0.007254]	0.6%	1.2%
Lemasson et al. (1997)	139	7692	0.018071	[0.015213–0.021302]	2.0%	1.2%
Lombardi et al. (1986)	13	263	0.049430	[0.026578–0.083043]	0.1%	1.2%
MacHault et al. (2009)	7	1101	0.006358	[0.002560–0.013055]	0.3%	1.2%
Mugenzi et al. (2022)	16	161	0.099379	[0.057878–0.156366]	0.0%	1.1%
Ndiath et al. (2011)	201	5387	0.037312	[0.032410–0.042723]	1.4%	1.2%
Ndiath et al. (2008)	29	897	0.032330	[0.021757–0.046103]	0.2%	1.2%
Ngwu et al. (2021)	8	565	0.014159	[0.006132–0.027708]	0.1%	1.2%
Obembe et al. (2022)	8	668	0.011976	[0.005184–0.023461]	0.2%	1.2%
Obembe et al. (2018)	52	1757	0.029596	[0.022181–0.038632]	0.5%	1.2%
Oduwole et al. (2023)	0	104	0.000000	[0.000000–0.034848]	0.0%	1.1%
Okorie et al. (2014)	0	31	0.000000	[0.000000–0.112189]	0.0%	0.8%
Okwa et al. (2009)	630	44820	0.014056	[0.012987–0.015189]	11.7%	1.3%
Orlandi−Pradines et al. (2009)	141	2656	0.053087	[0.044869–0.062307]	0.7%	1.2%
Oss.. et al. (2019)	8	539	0.014842	[0.006429–0.029035]	0.1%	1.2%
Oyewole et al. (2006)	32	792	0.040404	[0.027798–0.056562]	0.2%	1.2%
Pag..s et al. (2008)	14	4939	0.002835	[0.001551–0.004751]	1.3%	1.2%
Riveron et al. (2016)	12	126	0.095238	[0.050185–0.160453]	0.0%	1.1%
Salako et al. (2018)	305	3788	0.080517	[0.072047–0.089642]	1.0%	1.2%
Soma et al. (2018)	0	63	0.000000	[0.000000–0.056872]	0.0%	1.0%
Soma et al. (2020)	119	1808	0.065819	[0.054824–0.078246]	0.5%	1.2%
Sougoufara et al. (2018)	158	12122	0.013034	[0.011091–0.015216]	3.2%	1.3%
Sovi et al. (2013)	120	1621	0.074028	[0.061755–0.087870]	0.4%	1.2%
Sovi et al. (2013)	18	160	0.112500	[0.068054–0.171962]	0.0%	1.1%
Straif et al. (1998)	45	530	0.084906	[0.062604–0.111961]	0.1%	1.2%
Tchouassi et al. (2012)	15	990	0.015152	[0.008504–0.024867]	0.3%	1.2%
Thomson et al. (1995)	366	26131	0.014006	[0.012617–0.015506]	6.8%	1.3%
Traor.. et al. (2019)	110	2178	0.050505	[0.041689–0.060554]	0.6%	1.2%
Trape et al. (2014)	2160	87590	0.024660	[0.023643–0.025709]	22.9%	1.3%
Vezenegho et al. (2009)	43	465	0.092473	[0.067732–0.122538]	0.1%	1.2%
Wahedi et al. (2020)	21	597	0.035176	[0.021904–0.053269]	0.2%	1.2%
Wilson et al. (1998)	6	562	0.010676	[0.003928–0.023092]	0.1%	1.2%
Yokoly et al. (2021)	28	475	0.058947	[0.039524–0.084071]	0.1%	1.2%
Yovogan et al. (2021)	141	3605	0.039112	[0.033022–0.045964]	0.9%	1.2%
Zhao et al. (2021)	6	192	0.031250	[0.011553–0.066774]	0.1%	1.1%
Zogo et al. (2019)	58	855	0.067836	[0.051910–0.086815]	0.2%	1.2%
Zoh et al. (2020)	43	1992	0.021586	[0.015665–0.028967]	0.5%	1.2%
**Common effect model**		**383082**	**0.019476**	**[0.019041–0.019916]**	**100.0%**	**--**
**Random effects model**			**0.035298**	**[0.026208–0.045683]**	**--**	**100.0%**

Heterogeneity: $I^2 = 99\%$, $\tau^2 = 0.0145$, $p = 0$

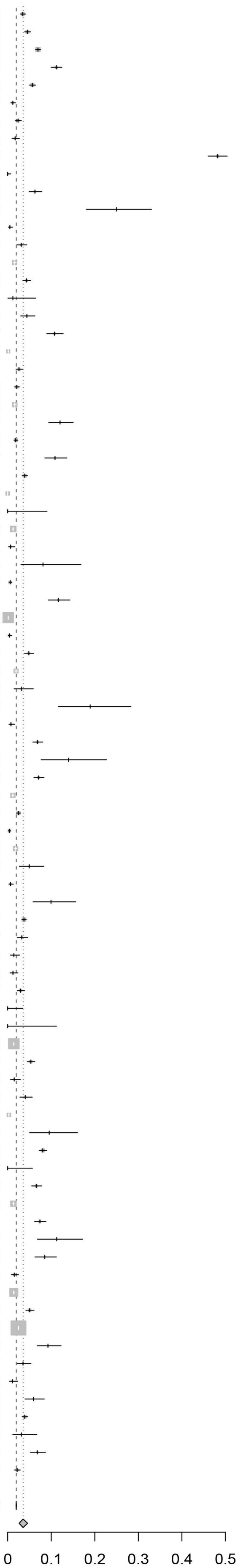

*Anopheles*_*Plasmodium malariae*

Study	Events	Total	Proportion	95% *CI*	Weight (common)	Weight (random)
Awolola et al. (2005)	0	1637	0.000000	[0.000000–0.002251]	6.6%	15.6%
Boudin et al. (1991)	4	1118	0.003578	[0.000976–0.009135]	4.5%	15.1%
Fontenille et al. (1997)	13	13258	0.000981	[0.000522–0.001676]	53.8%	16.8%
Lemasson et al. (1997)	4	7692	0.000520	[0.000142–0.001331]	31.2%	16.7%
Mugenzi et al. (2022)	5	161	0.031056	[0.010159–0.070983]	0.7%	8.9%
Riveron et al. (2016)	1	40	0.025000	[0.000633–0.131586]	0.2%	3.7%
Wilson et al. (1998)	1	562	0.001779	[0.000045–0.009874]	2.3%	13.5%
Zhao et al. (2021)	0	192	0.000000	[0.000000–0.019030]	0.8%	9.7%
**Common effect model**		**24660**	**0.000843**	**[0.000520–0.001244]**	**100.0%**	**--**
**Random effects model**			**0.002061**	**[0.000143–0.006208]**	**--**	**100.0%**

Heterogeneity: I^2 = 76%, τ^2 = 0.0017, p = 0.000139

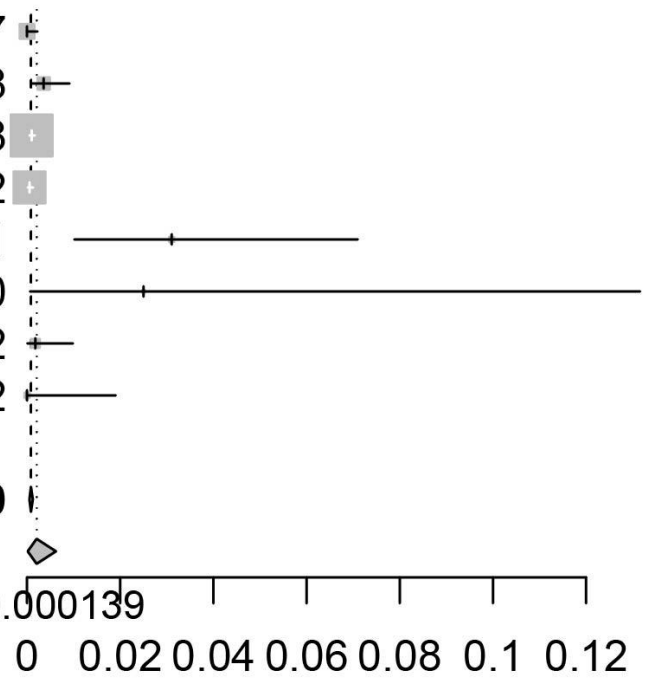

*Anopheles*_*Plasmodium ovale*

Study	Events	Total	Proportion	95% *CI*	Weight (common)	Weight (random)
Awolola et al. (2005)	0	1637	0.000000	[0.000000–0.002251]	4.4%	4.4%
Fontenille et al. (1997)	3	13258	0.000226	[0.000047–0.000661]	47.6%	47.6%
Lemasson et al. (1997)	1	7692	0.000130	[0.000003–0.000724]	48.0%	48.0%
Zhao et al. (2021)	0	192	0.000000	[0.000000–0.019030]	0.1%	0.1%
**Common effect model**		**22779**	**0.000170**	**[0.000000–0.000347]**	**100.0%**	**--**
**Random effects model**			**0.000170**	**[0.000000–0.000347]**	**--**	**100.0%**

Heterogeneity: I^2 = 0%, τ^2 = 0, p = 0.932379

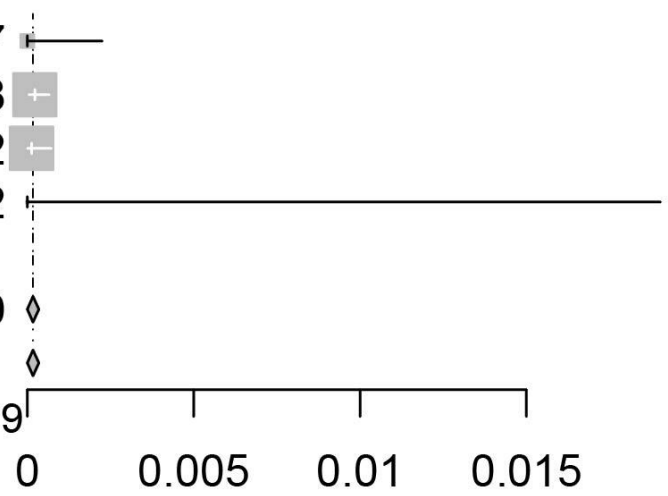

*Anopheles*_Rift valley fever virus

Study	Events	Total	Proportion	95% *CI*	Weight (common)	Weight (random)
Ndiaye et al. (2018)	0	2980	0.000000	[0.000000–0.001237]	82.0%	57.4%
Stoek et al. (2022)	1	537	0.001862	[0.000047–0.010331]	14.8%	31.9%
Wilson et al. (1994)	0	116	0.000000	[0.000000–0.031300]	3.2%	10.7%
**Common effect model**		**3633**	**0.000041**	**[0.000000–0.000512]**	**100.0%**	**--**
**Random effects model**			**0.000189**	**[0.000000–0.002133]**	**--**	**100.0%**

Heterogeneity: I^2 = 41%, τ^2 = 0.0004, p = 0.181693

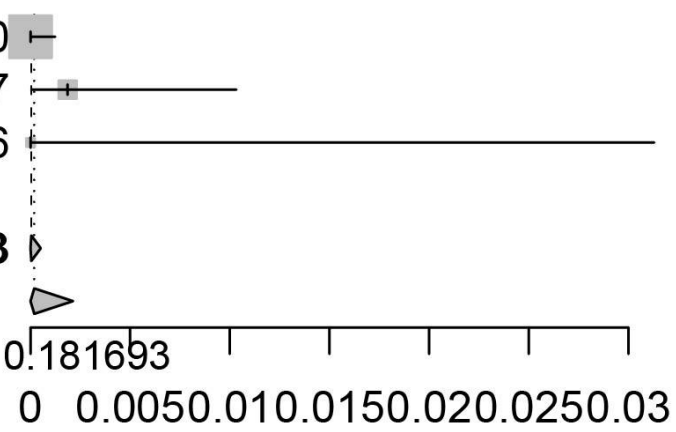

*Anopheles*_West Nile virus

Study	Events	Total	Proportion	95% *CI*	Weight (common)	Weight (random)
Fall et al. (2021)	0	92	0.000000	[0.000000–0.039303]	2.9%	2.9%
Fall et al. (2021)	0	4	0.000000	[0.000000–0.602365]	0.1%	0.1%
Ndiaye et al. (2018)	1	2980	0.000336	[0.000008–0.001868]	93.4%	93.4%
Wilson et al. (1994)	0	116	0.000000	[0.000000–0.031300]	3.6%	3.6%
**Common effect model**		**3192**	**0.000292**	**[0.000000–0.001186]**	**100.0%**	**--**
**Random effects model**			**0.000292**	**[0.000000–0.001186]**	**--**	**100.0%**

Heterogeneity: I^2 = 0%, τ^2 = 0, p = 0.966346

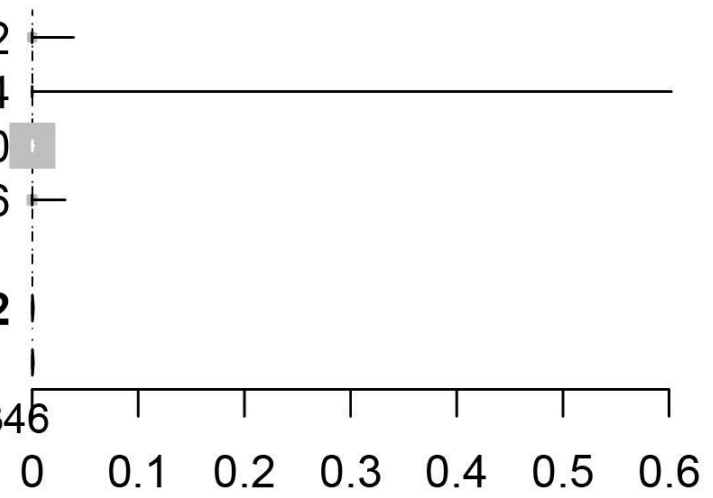

Anopheles_Wuchereria bancrofti

Study	Events	Total	Proportion	95% *CI*	Weight (common)	Weight (random)
Aboagye−Antwi et al. (2015)	67	584	0.114726	[0.090025–0.143400]	0.9%	8.0%
Appawu et al. (2001)	96	4616	0.020797	[0.016877–0.025338]	7.4%	8.4%
Coulibaly et al. (2015)	718	17701	0.040563	[0.037704–0.043574]	28.5%	8.4%
Coulibaly et al. (2016)	88	12951	0.006795	[0.005453–0.008365]	20.8%	8.4%
Dorkenoo et al. (2018)	0	9191	0.000000	[0.000000–0.000401]	14.8%	8.4%
Gbakima et al. (2005)	3	522	0.005747	[0.001187–0.016703]	0.8%	8.0%
Jr. Richards et al. (2005)	50	4278	0.011688	[0.008687–0.015380]	6.9%	8.4%
Kouassi et al. (2015)	2	161	0.012422	[0.001508–0.044152]	0.3%	7.2%
Lenhart et al. (2007)	155	4933	0.031421	[0.026730–0.036676]	7.9%	8.4%
Okorie et al. (2014)	0	7	0.000000	[0.000000–0.409616]	0.0%	1.7%
Pam et al. (2017)	20	1047	0.019102	[0.011706–0.029348]	1.7%	8.2%
Pi−Bansa et al. (2019)	0	4478	0.000000	[0.000000–0.000823]	7.2%	8.4%
de Souza et al. (2014)	1	1731	0.000578	[0.000015–0.003214]	2.8%	8.3%
**Common effect model**		**62200**	**0.013028**	**[0.012152–0.013934]**	**100.0%**	**--**
**Random effects model**			**0.013215**	**[0.003737–0.028373]**	**--**	**100.0%**

Heterogeneity: I^2 = 99%, τ^2 = 0.0090, p = 0

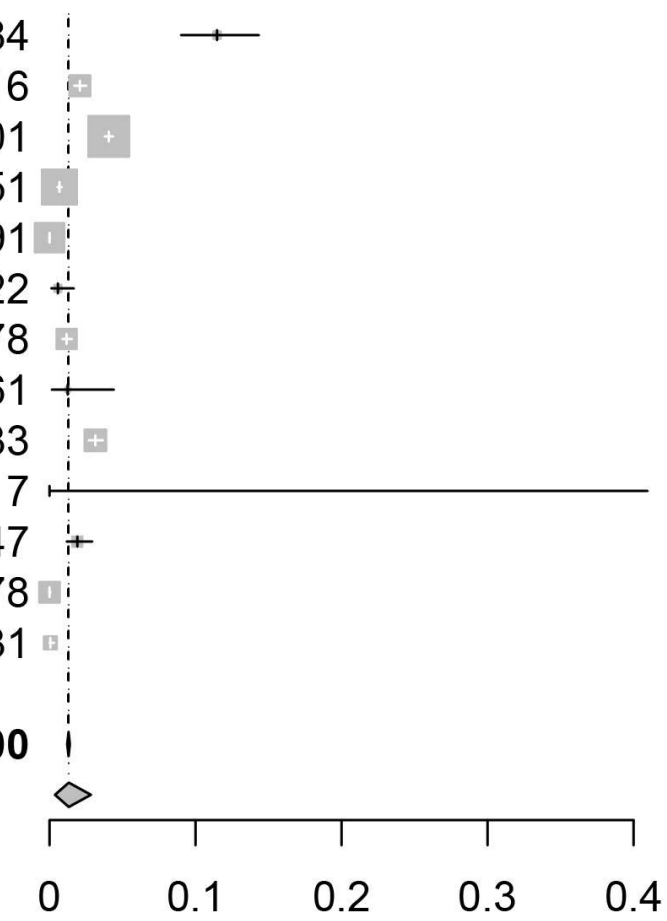

*Anopheles*_Yellow fever virus

Study	Events	Total	Proportion	95% *CI*	Weight (common)	Weight (random)
Agwu et al. (2019)	200	45424	0.004403	[0.003815–0.005056]	95.8%	33.6%
Diallo et al. (2020)	0	814	0.000000	[0.000000–0.004522]	1.7%	27.0%
Fall et al. (2021)	0	96	0.000000	[0.000000–0.037697]	0.2%	10.9%
Lee et al. (1972)	0	1094	0.000000	[0.000000–0.003366]	2.3%	28.5%
**Common effect model**		**47428**	**0.004039**	**[0.003489–0.004630]**	**100.0%**	**--**
**Random effects model**			**0.000497**	**[0.000000–0.003887]**	**--**	**100.0%**

Heterogeneity: I^2 = 91%, τ^2 = 0.0012, p < 0.000001

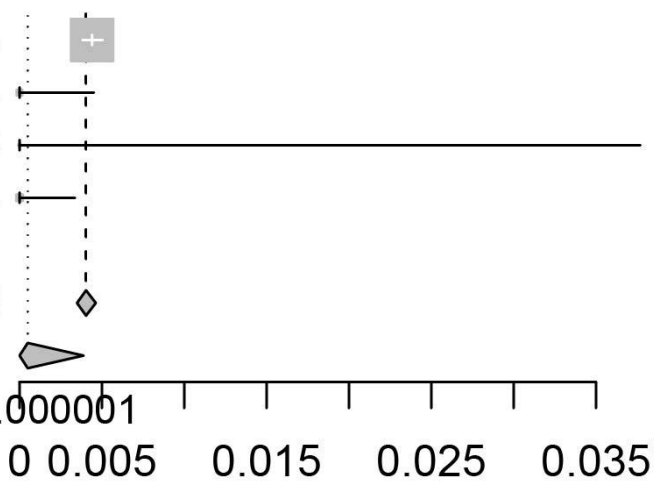

*Anopheles*_Zika virus

Study	Events	Total	Proportion	95% *CI*	Weight (common)	Weight (random)
Diallo et al. (2014)	1	710	0.001408	[0.000036–0.007822]	26.2%	27.2%
Diallo et al. (2020)	0	814	0.000000	[0.000000–0.004522]	30.0%	30.3%
Fall et al. (2021)	0	96	0.000000	[0.000000–0.037697]	3.5%	4.3%
Lee et al. (1972)	0	1094	0.000000	[0.000000–0.003366]	40.3%	38.2%
**Common effect model**		**2714**	**0.000096**	**[0.000000–0.000820]**	**100.0%**	**--**
**Random effects model**			**0.000104**	**[0.000000–0.000983]**	**--**	**100.0%**

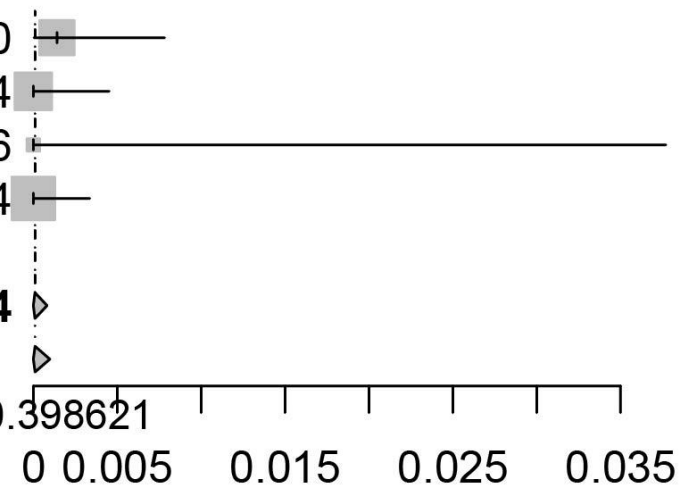

Heterogeneity: I^2 = 0%, τ^2 < 0.0001, *p* = 0.398621

Anopheles coustani s.l._Chikungunya virus

Study	Events	Total	Proportion	95% *CI*	Weight (common)	Weight (random)
Diallo et al. (2012)	1	1376	0.000727	[0.000018–0.004042]	0.9%	0.9%
Diallo et al. (1999)	1	14589	0.000069	[0.000002–0.000382]	98.9%	98.9%
Diallo et al. (2020)	0	463	0.000000	[0.000000–0.007936]	0.2%	0.2%
**Common effect model**		**16428**	**0.000074**	**[0.000000–0.000208]**	**100.0%**	**--**
**Random effects model**			**0.000074**	**[0.000000–0.000208]**	**--**	**100.0%**

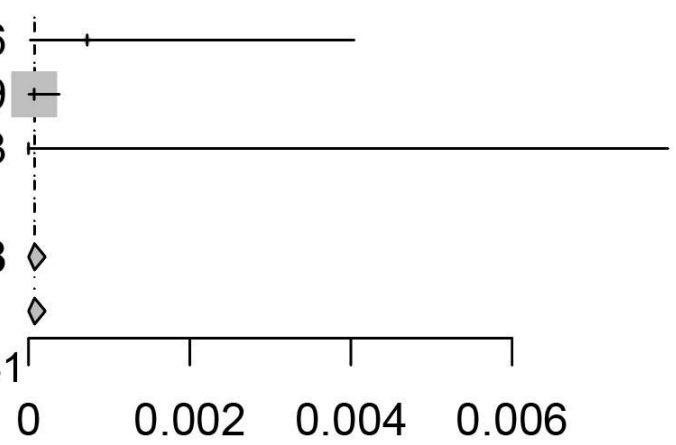

Heterogeneity: I^2 = 0%, τ^2 = 0, *p* = 0.664981

Anopheles coustani s.l._Zika virus

Study	Events	Total	Proportion	95% *CI*	Weight (common)	Weight (random)
Diallo et al. (2014)	1	710	0.001408	[0.000036–0.007822]	53.9%	53.9%
Diallo et al. (2020)	0	463	0.000000	[0.000000–0.007936]	46.1%	46.1%
**Common effect model**		**1173**	**0.000760**	**[0.000000–0.002786]**	**100.0%**	**--**
**Random effects model**			**0.000760**	**[0.000000–0.002786]**	**--**	**100.0%**

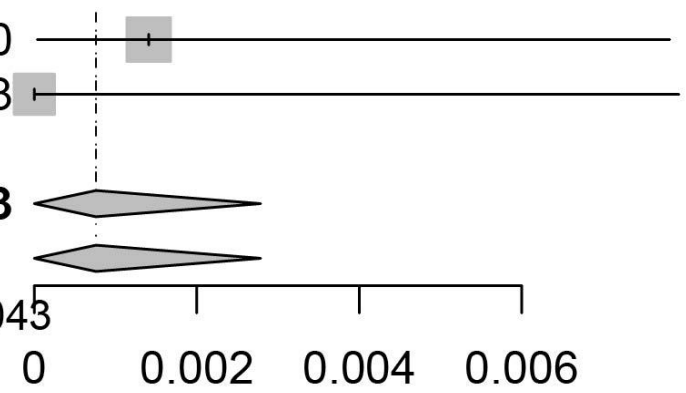

Heterogeneity: I^2 = 0%, τ^2 = 0, *p* = 0.497043

*Anopheles domicola*_Chikungunya virus

Study	Events	Total	Proportion	95% *CI*	Weight (common)	Weight (random)
Diallo et al. (2012)	1	22	0.045455	[0.001150–0.228444]	93.3%	93.3%
Diallo et al. (2020)	0	3	0.000000	[0.000000–0.707598]	6.7%	6.7%
**Common effect model**		**25**	**0.042397**	**[0.000000–0.126459]**	**100.0%**	**--**
**Random effects model**			**0.042397**	**[0.000000–0.126459]**	**--**	**100.0%**

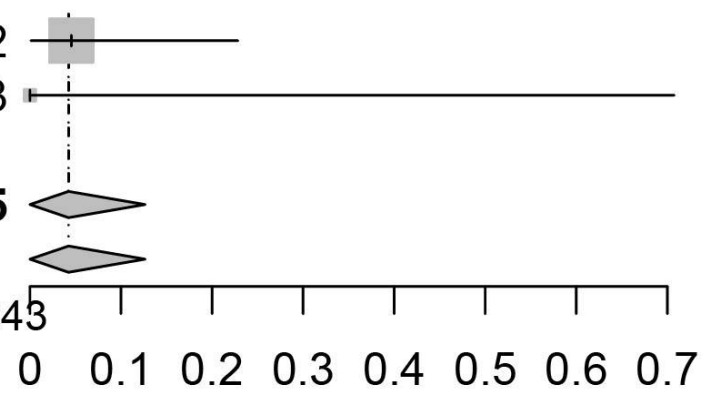

Heterogeneity: I^2 = 0%, τ^2 = 0, *p* = 0.790643

Anopheles funestus s.l._Chikungunya virus

Study	Events	Total	Proportion	95% *CI*	Weight (common)	Weight (random)
Diallo et al. (2012)	1	363	0.002755	[0.000070–0.015253]	92.1%	92.1%
Diallo et al. (2020)	0	74	0.000000	[0.000000–0.048628]	7.9%	7.9%
**Common effect model**		**437**	**0.002537**	**[0.000000–0.007712]**	**100.0%**	**--**
**Random effects model**			**0.002537**	**[0.000000–0.007712]**	**--**	**100.0%**

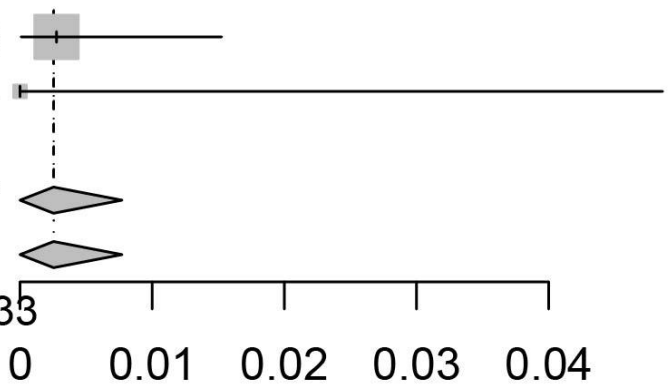

Heterogeneity: $I^2 = 0\%$, $\tau^2 = 0$, $p = 0.778433$

Anopheles funestus s.l._*Plasmodium falciparum*

Study	Events	Total	Proportion	95% *CI*	Weight (common)	Weight (random)
Adja et al. (2022)	12	166	0.072289	[0.037909–0.122869]	0.2%	3.1%
Appawu et al. (2004)	269	3773	0.071296	[0.063287–0.079976]	4.6%	3.4%
Appawu et al. (2003)	134	971	0.138002	[0.116917–0.161309]	1.2%	3.4%
Assouho et al. (2020)	55	459	0.119826	[0.091558–0.153100]	0.6%	3.3%
Awolola et al. (2005)	19	1476	0.012873	[0.007767–0.020029]	1.8%	3.4%
Boudin et al. (1991)	31	480	0.064583	[0.044299–0.090421]	0.6%	3.3%
Carnevale et al. (2010)	4	44	0.090909	[0.025328–0.216687]	0.1%	2.4%
Czeher et al. (2006)	42	1172	0.035836	[0.025947–0.048133]	1.4%	3.4%
Dabir.. et al. (2008)	118	1097	0.107566	[0.089847–0.127420]	1.3%	3.4%
Dia et al. (2008)	1	3076	0.000325	[0.000008–0.001810]	3.7%	3.4%
Dia et al. (2003)	20	519	0.038536	[0.023695–0.058890]	0.6%	3.3%
Dia et al. (2005)	24	472	0.050847	[0.032847–0.074715]	0.6%	3.3%
Diakit.. et al. (2015)	20	1791	0.011167	[0.006834–0.017194]	2.2%	3.4%
Djouaka et al. (2016)	66	498	0.132530	[0.104002–0.165504]	0.6%	3.3%
Fagbohun et al. (2020)	0	385	0.000000	[0.000000–0.009536]	0.5%	3.3%
Fontenille et al. (1997)	95	3617	0.026265	[0.021301–0.032013]	4.4%	3.4%
Fournet et al. (2010)	0	2	0.000000	[0.000000–0.841886]	0.0%	0.3%
Kouassi et al. (2023)	16	215	0.074419	[0.043133–0.118039]	0.3%	3.2%
Labbo et al. (2010)	7	1047	0.006686	[0.002692–0.013726]	1.3%	3.4%
Mugenzi et al. (2022)	12	120	0.100000	[0.052748–0.168165]	0.1%	3.0%
Ndiath et al. (2011)	142	3454	0.041112	[0.034737–0.048278]	4.2%	3.4%
Okwa et al. (2009)	170	9420	0.018047	[0.015455–0.020942]	11.5%	3.4%
Orlandi−Pradines et al. (2009)	24	652	0.036810	[0.023725–0.054277]	0.8%	3.3%
Oss.. et al. (2019)	2	161	0.012422	[0.001508–0.044152]	0.2%	3.1%
Oyewole et al. (2006)	0	90	0.000000	[0.000000–0.040159]	0.1%	2.8%
Riveron et al. (2016)	9	80	0.112500	[0.052752–0.202847]	0.1%	2.8%
Soma et al. (2020)	0	3	0.000000	[0.000000–0.707598]	0.0%	0.5%
Sougoufara et al. (2018)	41	3733	0.010983	[0.007893–0.014871]	4.5%	3.4%
Tchouassi et al. (2012)	0	5	0.000000	[0.000000–0.521824]	0.0%	0.7%
Traor.. et al. (2019)	1	51	0.019608	[0.000496–0.104475]	0.1%	2.5%
Trape et al. (2014)	1271	42686	0.029776	[0.028184–0.031432]	52.0%	3.4%
Wilson et al. (1998)	0	239	0.000000	[0.000000–0.015316]	0.3%	3.2%
Yokoly et al. (2021)	4	68	0.058824	[0.016258–0.143818]	0.1%	2.7%
Zogo et al. (2019)	8	48	0.166667	[0.074811–0.302222]	0.1%	2.4%
**Common effect model**		**82070**	**0.028367**	**[0.027242–0.029513]**	**100.0%**	**--**
**Random effects model**			**0.038563**	**[0.024364–0.055851]**	**--**	**100.0%**

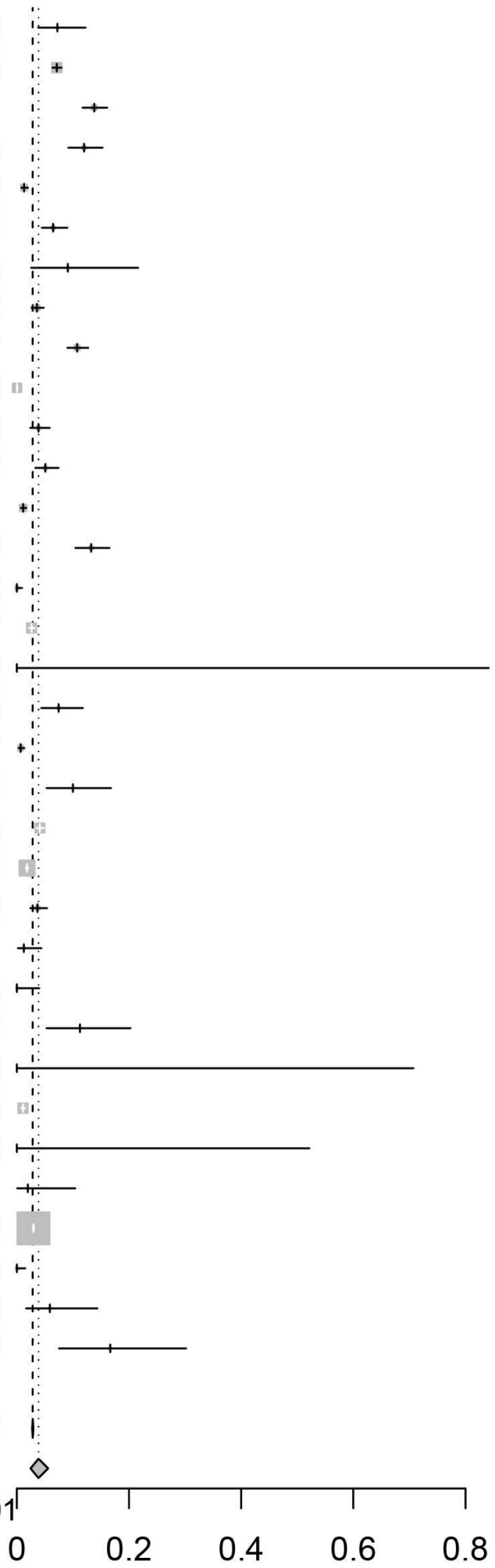

Heterogeneity: $I^2 = 97\%$, $\tau^2 = 0.0127$, $p < 0.000001$

Anopheles funestus s.l._*Plasmodium malariae*

Study	Events	Total	Proportion	95% *CI*	Weight (common)	Weight (random)
Awolola et al. (2005)	0	1476	0.000000	[0.000000–0.002496]	24.7%	22.1%
Boudin et al. (1991)	2	480	0.004167	[0.000505–0.014970]	8.0%	19.4%
Fontenille et al. (1997)	3	3617	0.000829	[0.000171–0.002422]	60.6%	23.0%
Mugenzi et al. (2022)	3	120	0.025000	[0.005186–0.071323]	2.0%	12.6%
Riveron et al. (2016)	1	40	0.025000	[0.000633–0.131586]	0.7%	6.5%
Wilson et al. (1998)	0	239	0.000000	[0.000000–0.015316]	4.0%	16.4%
**Common effect model**		**5972**	**0.000723**	**[0.000202–0.001565]**	**100.0%**	**--**
**Random effects model**			**0.002449**	**[0.000009–0.009183]**	**--**	**100.0%**

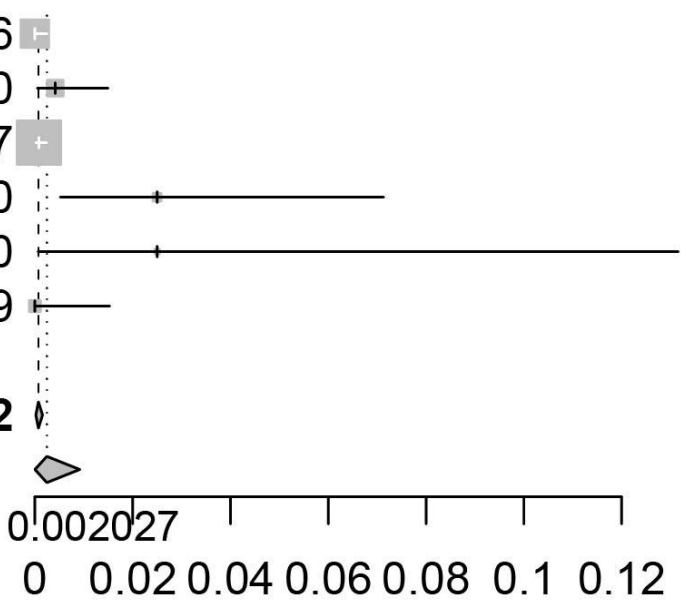

Heterogeneity: $I^2 = 74\%$, $\tau^2 = 0.0024$, $p = 0.002027$

Anopheles funestus s.l._*Wuchereria bancrofti*

Study	Events	Total	Proportion	95% *CI*	Weight (common)	Weight (random)
Aboagye−Antwi et al. (2015)	23	144	0.159722	[0.104026–0.229960]	2.4%	17.2%
Appawu et al. (2001)	6	773	0.007762	[0.002854–0.016817]	13.1%	18.4%
Coulibaly et al. (2015)	72	1471	0.048946	[0.038491–0.061246]	25.0%	18.6%
Coulibaly et al. (2016)	0	11	0.000000	[0.000000–0.284914]	0.2%	8.6%
Jr. Richards et al. (2005)	3	2139	0.001403	[0.000289–0.004093]	36.3%	18.6%
Lenhart et al. (2007)	15	1352	0.011095	[0.006223–0.018233]	23.0%	18.6%
**Common effect model**		**5890**	**0.013205**	**[0.010449–0.016279]**	**100.0%**	**--**
**Random effects model**			**0.023818**	**[0.001363–0.072683]**	**--**	**100.0%**

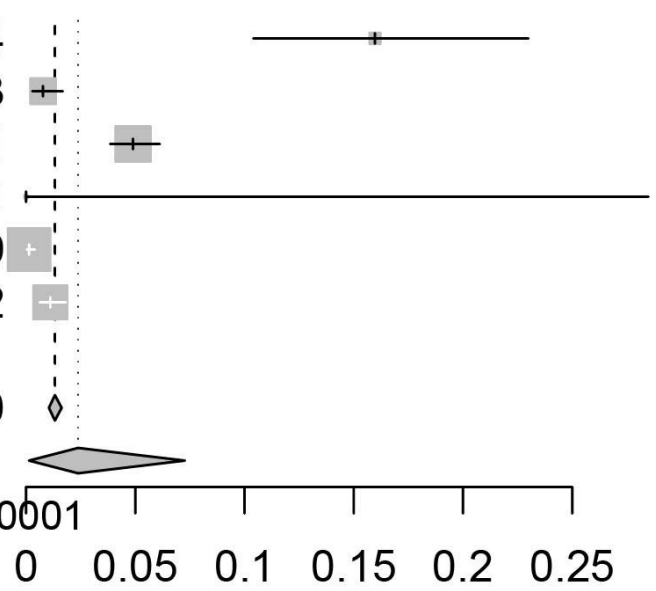

Heterogeneity: $I^2 = 97\%$, $\tau^2 = 0.0194$, $p < 0.000001$

Anopheles gambiae s.l._*Plasmodium falciparum*

Study	Events	Total	Proportion	95% *CI*	Weight (common)	Weight (random)
Adja et al. (2022)	115	3502	0.032838	[0.027186–0.039287]	1.2%	1.3%
Akogb..to et al. (2015)	157	3462	0.045350	[0.038661–0.052819]	1.2%	1.3%
Appawu et al. (2004)	295	4083	0.072251	[0.064495–0.080625]	1.4%	1.3%
Appawu et al. (2003)	150	1574	0.095299	[0.081240–0.110886]	0.6%	1.3%
Assouho et al. (2020)	72	2566	0.028059	[0.022018–0.035207]	0.9%	1.3%
Awolola et al. (2014)	48	2015	0.023821	[0.017615–0.031461]	0.7%	1.3%
Awolola et al. (2002)	10	617	0.016207	[0.007799–0.029604]	0.2%	1.3%
Basing et al. (2014)	975	2022	0.482196	[0.460208–0.504235]	0.7%	1.3%
Bimbile Somdal et al. (2018)	0	506	0.000000	[0.000000–0.007264]	0.2%	1.3%
Boudin et al. (1991)	35	601	0.058236	[0.040895–0.080065]	0.2%	1.3%
Carnevale et al. (2010)	31	96	0.322917	[0.231041–0.426058]	0.0%	1.1%
Carrara et al. (1990)	0	98	0.000000	[0.000000–0.036942]	0.0%	1.1%
Collins et al. (2019)	30	968	0.030992	[0.021006–0.043949]	0.3%	1.3%
Coulibaly et al. (2022)	121	7684	0.015747	[0.013083–0.018787]	2.7%	1.3%
Czeher et al. (2006)	48	920	0.052174	[0.038716–0.068583]	0.3%	1.3%
Dabire et al. (2008)	1	84	0.011905	[0.000301–0.064552]	0.0%	1.1%
Dabire et al. (2012)	29	658	0.044073	[0.029712–0.062685]	0.2%	1.3%
Dia et al. (2008)	0	595	0.000000	[0.000000–0.006181]	0.2%	1.3%
Dia et al. (2003)	3	191	0.015707	[0.003251–0.045213]	0.1%	1.2%
Dia et al. (2005)	22	1851	0.011885	[0.007463–0.017940]	0.6%	1.3%
Diakit.. et al. (2015)	110	6227	0.017665	[0.014540–0.021252]	2.2%	1.3%
Dj..nontin et al. (2021)	71	3848	0.018451	[0.014438–0.023217]	1.4%	1.3%
Ebenezer et al. (2016)	67	617	0.108590	[0.085152–0.135849]	0.2%	1.3%
Epopa et al. (2019)	152	3891	0.039065	[0.033197–0.045637]	1.4%	1.3%
Fagbohun et al. (2020)	0	4732	0.000000	[0.000000–0.000779]	1.7%	1.3%
Fofana et al. (2020)	0	39	0.000000	[0.000000–0.090251]	0.0%	0.9%
Fontenille et al. (1997)	70	9641	0.007261	[0.005664–0.009165]	3.4%	1.3%
Forson et al. (2022)	4	620	0.006452	[0.001761–0.016436]	0.2%	1.3%
Fournet et al. (2010)	6	72	0.083333	[0.031198–0.172609]	0.0%	1.1%
Fournet et al. (2022)	12	2142	0.005602	[0.002898–0.009766]	0.8%	1.3%
Fryxell et al. (2012)	78	671	0.116244	[0.092979–0.142945]	0.2%	1.3%
Gadiaga et al. (2011)	54	44628	0.001210	[0.000909–0.001578]	15.7%	1.3%
Girod et al. (2006)	4	1061	0.003770	[0.001028–0.009624]	0.4%	1.3%
Gnanguenon et al. (2014)	80	1652	0.048426	[0.038583–0.059911]	0.6%	1.3%
Gomes et al. (2017)	135	7068	0.019100	[0.016038–0.022568]	2.5%	1.3%
Govoetchan et al. (2014)	9	286	0.031469	[0.014488–0.058895]	0.1%	1.2%
Hanemaaijer et al. (2019)	18	95	0.189474	[0.116335–0.282819]	0.0%	1.1%
Hogg et al. (1996)	6	801	0.007491	[0.002754–0.016232]	0.3%	1.3%
Horton et al. (2010)	134	1961	0.068332	[0.057562–0.080414]	0.7%	1.3%
Ibrahim et al. (2019)	13	93	0.139785	[0.076582–0.227169]	0.0%	1.1%
Jaenson et al. (1994)	139	1956	0.071063	[0.060071–0.083361]	0.7%	1.3%
Ke?ta et al. (2021)	92	7782	0.011822	[0.009541–0.014479]	2.7%	1.3%
Kouassi et al. (2023)	87	3984	0.021837	[0.017527–0.026867]	1.4%	1.3%
Labbo et al. (2010)	1	122	0.008197	[0.000208–0.044823]	0.0%	1.2%
Lemasson et al. (1997)	139	7692	0.018071	[0.015213–0.021302]	2.7%	1.3%
Lombardi et al. (1986)	13	263	0.049430	[0.026578–0.083043]	0.1%	1.2%
MacHault et al. (2009)	7	1101	0.006358	[0.002560–0.013055]	0.4%	1.3%
Mugenzi et al. (2022)	4	41	0.097561	[0.027227–0.231315]	0.0%	0.9%
Ndiath et al. (2011)	59	1933	0.030523	[0.023315–0.039197]	0.7%	1.3%
Ndiath et al. (2008)	29	897	0.032330	[0.021757–0.046103]	0.3%	1.3%
Ngwu et al. (2021)	8	565	0.014159	[0.006132–0.027708]	0.2%	1.3%
Obembe et al. (2022)	8	668	0.011976	[0.005184–0.023461]	0.2%	1.3%
Obembe et al. (2018)	52	1757	0.029596	[0.022181–0.038632]	0.6%	1.3%
Oduwole et al. (2023)	0	101	0.000000	[0.000000–0.035865]	0.0%	1.1%
Okorie et al. (2014)	0	31	0.000000	[0.000000–0.112189]	0.0%	0.9%
Okwa et al. (2009)	370	25570	0.014470	[0.013042–0.016010]	9.0%	1.3%
Orlandi−Pradines et al. (2009)	107	1445	0.074048	[0.061078–0.088780]	0.5%	1.3%
Oss.. et al. (2019)	5	357	0.014006	[0.004563–0.032380]	0.1%	1.3%
Oyewole et al. (2006)	32	702	0.045584	[0.031385–0.063744]	0.2%	1.3%
Pag..s et al. (2008)	14	4939	0.002835	[0.001551–0.004751]	1.7%	1.3%
Riveron et al. (2016)	3	46	0.065217	[0.013657–0.178964]	0.0%	1.0%
Salako et al. (2018)	305	3788	0.080517	[0.072047–0.089642]	1.3%	1.3%
Soma et al. (2018)	0	63	0.000000	[0.000000–0.056872]	0.0%	1.0%
Soma et al. (2020)	119	1780	0.066854	[0.055692–0.079468]	0.6%	1.3%
Sougoufara et al. (2018)	117	8389	0.013947	[0.011548–0.016692]	2.9%	1.3%
Sovi et al. (2013)	120	1621	0.074028	[0.061755–0.087870]	0.6%	1.3%
Sovi et al. (2013)	18	160	0.112500	[0.068054–0.171962]	0.1%	1.2%
Straif et al. (1998)	45	530	0.084906	[0.062604–0.111961]	0.2%	1.3%
Tchouassi et al. (2012)	15	984	0.015244	[0.008556–0.025018]	0.3%	1.3%
Thomson et al. (1995)	366	26131	0.014006	[0.012617–0.015506]	9.2%	1.3%
Traor.. et al. (2019)	109	2127	0.051246	[0.042264–0.061488]	0.7%	1.3%
Trape et al. (2014)	889	44904	0.019798	[0.018529–0.021129]	15.8%	1.3%
Vezenegho et al. (2009)	43	465	0.092473	[0.067732–0.122538]	0.2%	1.3%
Wahedi et al. (2020)	21	597	0.035176	[0.021904–0.053269]	0.2%	1.3%
Wilson et al. (1998)	6	323	0.018576	[0.006847–0.039991]	0.1%	1.2%
Yokoly et al. (2021)	24	400	0.060000	[0.038818–0.087962]	0.1%	1.3%
Yovogan et al. (2021)	141	3605	0.039112	[0.033022–0.045964]	1.3%	1.3%
Zhao et al. (2021)	6	192	0.031250	[0.011553–0.066774]	0.1%	1.2%
Zogo et al. (2019)	49	743	0.065949	[0.049184–0.086254]	0.3%	1.3%
Zoh et al. (2020)	43	1992	0.021586	[0.015665–0.028967]	0.7%	1.3%
**Common effect model**		**284984**	**0.017267**	**[0.016792–0.017749]**	**100.0%**	**--**
**Random effects model**			**0.033322**	**[0.024143–0.043919]**	**--**	**100.0%**

Heterogeneity: $I^2 = 99\%$, $\tau^2 = 0.0151$, $p = 0$

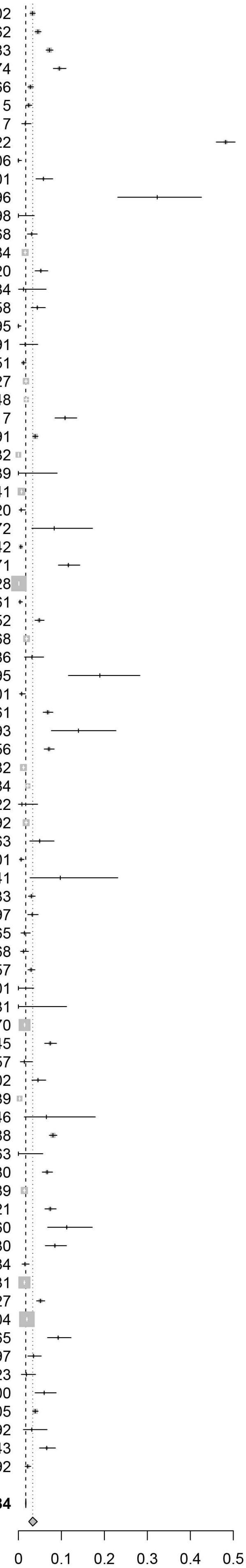

Anopheles gambiae s.l._*Plasmodium malariae*

Study	Events	Total	Proportion	95% *CI*	Weight (common)	Weight (random)
Boudin et al. (1991)	1	601	0.001664	[0.000042–0.009235]	3.3%	4.4%
Fontenille et al. (1997)	10	9641	0.001037	[0.000498–0.001907]	52.1%	49.4%
Lemasson et al. (1997)	4	7692	0.000520	[0.000142–0.001331]	41.6%	42.1%
Mugenzi et al. (2022)	2	41	0.048780	[0.005963–0.165333]	0.2%	0.3%
Wilson et al. (1998)	1	323	0.003096	[0.000078–0.017128]	1.7%	2.4%
Zhao et al. (2021)	0	192	0.000000	[0.000000–0.019030]	1.0%	1.4%
**Common effect model**		**18490**	**0.000845**	**[0.000478–0.001316]**	**100.0%**	**--**
**Random effects model**			**0.000859**	**[0.000434–0.001426]**	**--**	**100.0%**

Heterogeneity: $I^2 = 48\%$, $\tau^2 < 0.0001$, $p = 0.086400$

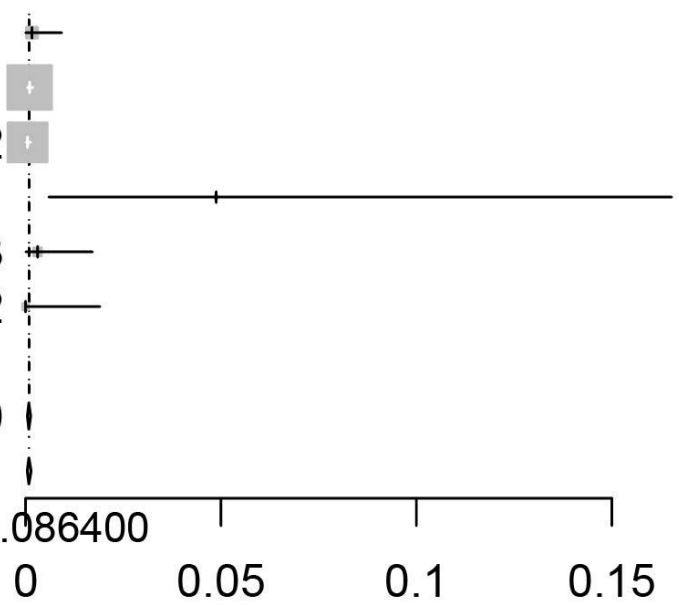

Anopheles gambiae s.l._*Plasmodium ovale*

Study	Events	Total	Proportion	95% *CI*	Weight (common)	Weight (random)
Fontenille et al. (1997)	3	9641	0.000311	[0.000064–0.000909]	34.3%	34.3%
Lemasson et al. (1997)	1	7692	0.000130	[0.000003–0.000724]	65.6%	65.6%
Zhao et al. (2021)	0	192	0.000000	[0.000000–0.019030]	0.1%	0.1%
**Common effect model**		**17525**	**0.000192**	**[0.000000–0.000398]**	**100.0%**	**--**
**Random effects model**			**0.000192**	**[0.000000–0.000398]**	**--**	**100.0%**

Heterogeneity: $I^2 = 0\%$, $\tau^2 = 0$, $p = 0.715220$

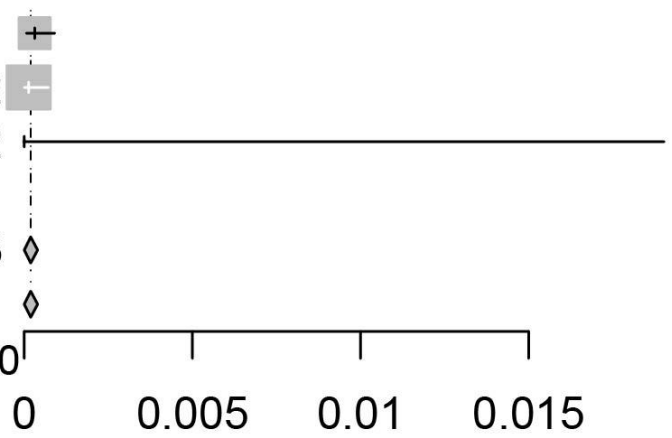

Anopheles gambiae s.l._*Wuchereria bancrofti*

Study	Events	Total	Proportion	95% *CI*	Weight (common)	Weight (random)
Aboagye−Antwi et al. (2015)	44	440	0.100000	[0.073604–0.131916]	0.8%	7.9%
Appawu et al. (2001)	90	3843	0.023419	[0.018873–0.028709]	6.8%	8.4%
Coulibaly et al. (2015)	646	16230	0.039803	[0.036847–0.042925]	28.9%	8.4%
Coulibaly et al. (2016)	88	12869	0.006838	[0.005488–0.008418]	22.9%	8.4%
Dorkenoo et al. (2018)	0	9191	0.000000	[0.000000–0.000401]	16.3%	8.4%
Gbakima et al. (2005)	3	522	0.005747	[0.001187–0.016703]	0.9%	8.0%
Jr. Richards et al. (2005)	47	2139	0.021973	[0.016188–0.029113]	3.8%	8.3%
Kouassi et al. (2015)	2	161	0.012422	[0.001508–0.044152]	0.3%	7.1%
Lenhart et al. (2007)	140	3581	0.039095	[0.032986–0.045970]	6.4%	8.4%
Okorie et al. (2014)	0	7	0.000000	[0.000000–0.409616]	0.0%	1.6%
Pam et al. (2017)	20	1047	0.019102	[0.011706–0.029348]	1.9%	8.2%
Pi−Bansa et al. (2019)	0	4478	0.000000	[0.000000–0.000823]	8.0%	8.4%
de Souza et al. (2014)	1	1731	0.000578	[0.000015–0.003214]	3.1%	8.3%
**Common effect model**		**56239**	**0.012626**	**[0.011720–0.013565]**	**100.0%**	**--**
**Random effects model**			**0.014052**	**[0.004412–0.029014]**	**--**	**100.0%**

Heterogeneity: $I^2 = 99\%$, $\tau^2 = 0.0084$, $p < 0.000001$

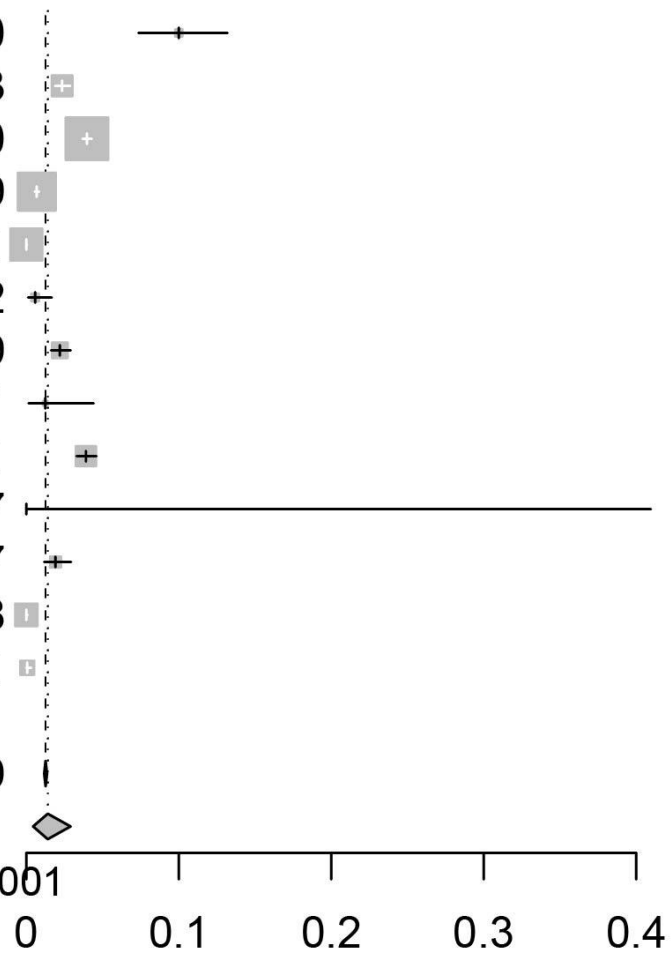

Anopheles gambiae s.l._Yellow fever virus

Study	Events	Total	Proportion	95% *CI*	Weight (common)	Weight (random)
Agwu et al. (2019)	200	45290	0.004416	[0.003826–0.005071]	98.8%	42.7%
Diallo et al. (2020)	0	183	0.000000	[0.000000–0.019956]	0.4%	20.0%
Fall et al. (2021)	0	92	0.000000	[0.000000–0.039303]	0.2%	13.1%
Lee et al. (1972)	0	271	0.000000	[0.000000–0.013520]	0.6%	24.2%
**Common effect model**		**45836**	**0.004312**	**[0.003733–0.004932]**	**100.0%**	**--**
**Random effects model**			**0.000807**	**[0.000000–0.005282]**	**--**	**100.0%**

Heterogeneity: I^2 = 69%, τ^2 = 0.0012, p = 0.022871

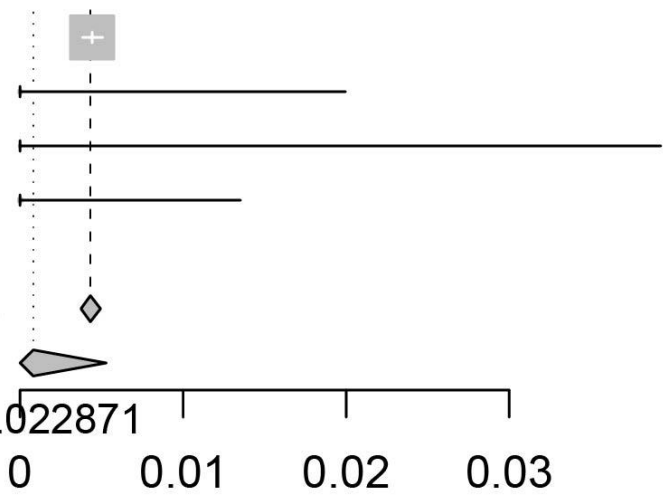

Anopheles moucheti_Plasmodium falciparum

Study	Events	Total	Proportion	95% *CI*	Weight (common)	Weight (random)
Awolola et al. (2002)	7	385	0.018182	[0.007341–0.037101]	2.0%	22.1%
Okwa et al. (2009)	90	9830	0.009156	[0.007368–0.011242]	98.0%	77.9%
**Common effect model**		**10215**	**0.009332**	**[0.007467–0.011196]**	**100.0%**	**--**
**Random effects model**			**0.011151**	**[0.003810–0.018493]**	**--**	**100.0%**

Heterogeneity: I^2 = 42%, τ^2 < 0.0001, p = 0.189330

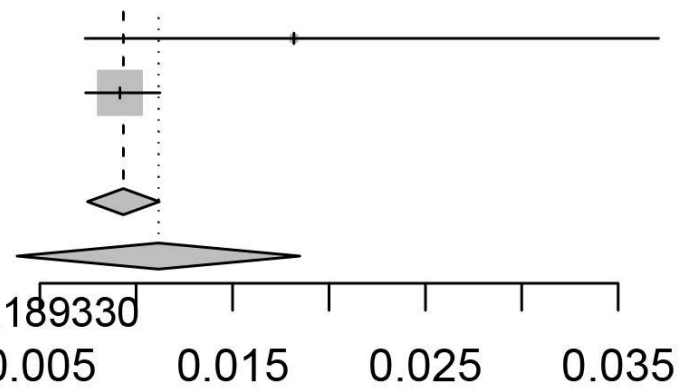

Anopheles nili s.l._*Plasmodium falciparum*

Study	Events	Total	Proportion	95% *CI*	Weight (common)	Weight (random)
Assouho et al. (2020)	102	1020	0.100000	[0.082274–0.120071]	33.7%	16.6%
Boudin et al. (1991)	4	37	0.108108	[0.030252–0.254176]	1.2%	8.7%
Dia et al. (2003)	22	1031	0.021339	[0.013420–0.032129]	34.0%	16.6%
Dia et al. (2005)	8	269	0.029740	[0.012925–0.057756]	8.9%	15.2%
Orlandi−Pradines et al. (2009)	10	559	0.017889	[0.008611–0.032652]	18.4%	16.2%
Oss.. et al. (2019)	1	21	0.047619	[0.001205–0.238160]	0.7%	6.3%
Soma et al. (2020)	0	23	0.000000	[0.000000–0.148185]	0.8%	6.7%
Yokoly et al. (2021)	0	7	0.000000	[0.000000–0.409616]	0.2%	2.8%
Zogo et al. (2019)	1	64	0.015625	[0.000396–0.084010]	2.1%	11.0%
**Common effect model**		**3031**	**0.042050**	**[0.035197–0.049484]**	**100.0%**	**--**
**Random effects model**			**0.032951**	**[0.013170–0.061236]**	**--**	**100.0%**

Heterogeneity: I^2 = 91%, τ^2 = 0.0069, p < 0.000001

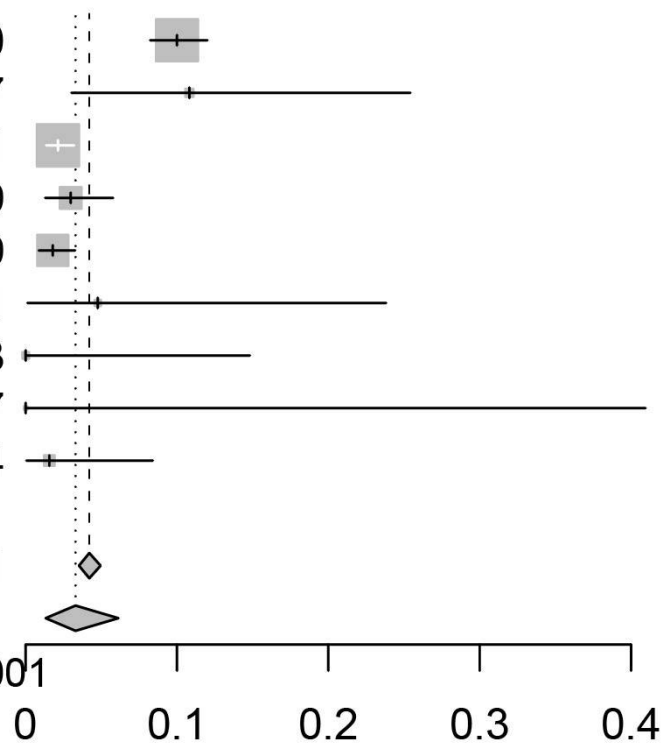

*Anopheles pharoensis*_*Plasmodium falciparum*

Study	Events	Total	Proportion	95% *CI*	Weight (common)	Weight (random)
Appawu et al. (2004)	0	256	0.000000	[0.000000–0.014306]	39.1%	38.8%
Carrara et al. (1990)	5	912	0.005482	[0.001782–0.012748]	49.4%	43.2%
Dia et al. (2008)	4	394	0.010152	[0.002773–0.025789]	11.6%	18.0%
Soma et al. (2020)	0	1	0.000000	[0.000000–0.975000]	0.0%	0.0%
Tchouassi et al. (2012)	0	1	0.000000	[0.000000–0.975000]	0.0%	0.0%
**Common effect model**		**1564**	**0.003881**	**[0.000514–0.007248]**	**100.0%**	**--**
**Random effects model**			**0.004194**	**[0.000000–0.009003]**	**--**	**100.0%**

Heterogeneity: $I^2 = 0\%$, $\tau^2 < 0.0001$, $p = 0.410786$

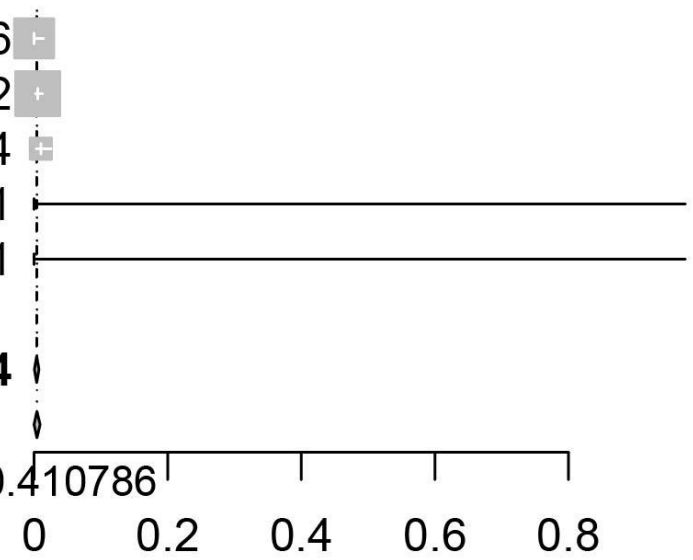

*Anopheles pharoensis*_Rift valley fever virus

Study	Events	Total	Proportion	95% *CI*	Weight (common)	Weight (random)
Ndiaye et al. (2018)	0	183	0.000000	[0.000000–0.019956]	24.6%	24.6%
Stoek et al. (2022)	1	526	0.001901	[0.000048–0.010547]	70.8%	70.8%
Wilson et al. (1994)	0	34	0.000000	[0.000000–0.102818]	4.6%	4.6%
**Common effect model**		**743**	**0.000953**	**[0.000000–0.004460]**	**100.0%**	**--**
**Random effects model**			**0.000953**	**[0.000000–0.004460]**	**--**	**100.0%**

Heterogeneity: $I^2 = 0\%$, $\tau^2 = 0$, $p = 0.557391$

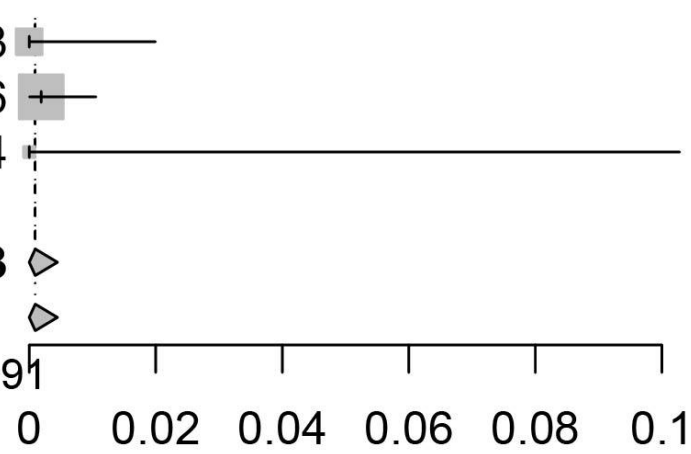

*Anopheles rufipes*_Chikungunya virus

Study	Events	Total	Proportion	95% *CI*	Weight (common)	Weight (random)
Diallo et al. (1999)	1	38495	0.000026	[0.000001–0.000145]	100.0%	100.0%
Diallo et al. (2020)	0	20	0.000000	[0.000000–0.168433]	0.0%	0.0%
**Common effect model**		**38515**	**0.000026**	**[0.000000–0.000077]**	**100.0%**	**--**
**Random effects model**			**0.000026**	**[0.000000–0.000077]**	**--**	**100.0%**

Heterogeneity: $I^2 = 0\%$, $\tau^2 = 0$, $p = 0.999377$

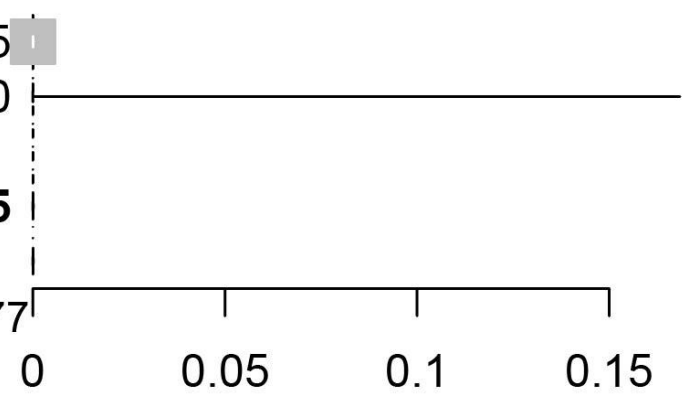

*Anopheles rufipes*_West nile virus

Study	Events	Total	Proportion	95% *CI*	Weight (common)	Weight (random)
Fall et al. (2021)	0	4	0.000000	[0.000000–0.602365]	3.2%	3.2%
Ndiaye et al. (2018)	1	111	0.009009	[0.000228–0.049173]	88.8%	88.8%
Wilson et al. (1994)	0	10	0.000000	[0.000000–0.308497]	8.0%	8.0%
**Common effect model**		**125**	**0.007109**	**[0.000000–0.029315]**	**100.0%**	**--**
**Random effects model**			**0.007109**	**[0.000000–0.029315]**	**--**	**100.0%**

Heterogeneity: $I^2 = 0\%$, $\tau^2 = 0$, $p = 0.798775$

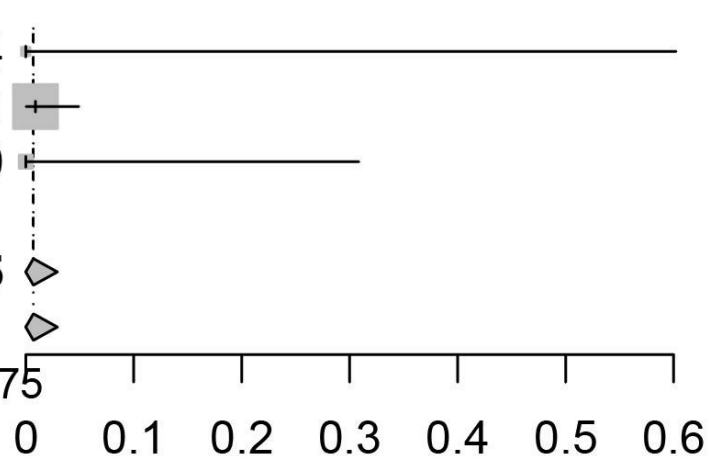

Supplementary Fig. 11. Meta-analysis of the positivity rate of each *Culex*-associated pathogen. The heterogeneity of combined studies was quantified by I^2 statistic. The random effect model would be applied if $I^2 > 50\%$; Otherwise, the fixed effect model would be applied.

*Culex*_Bagaza virus

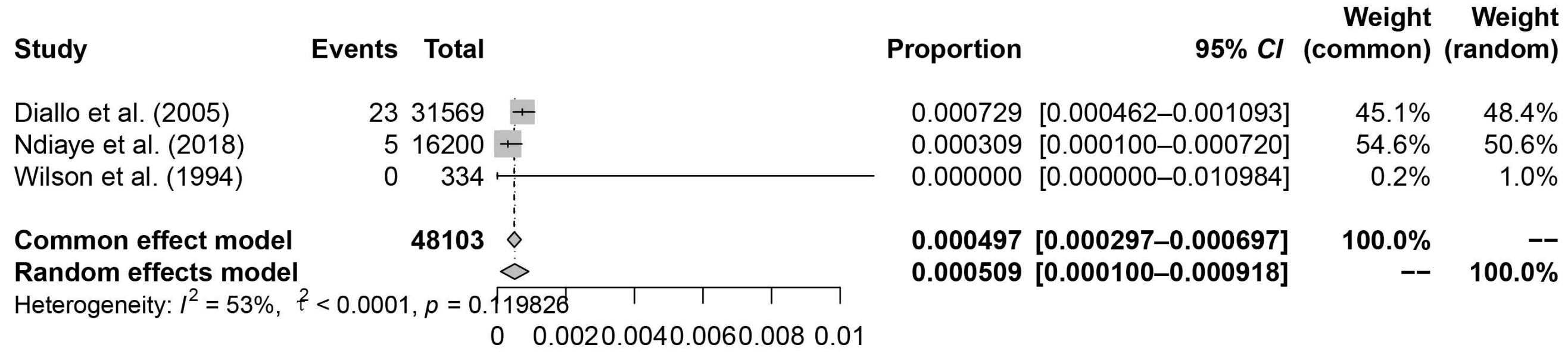

*Culex*_Chikungunya virus

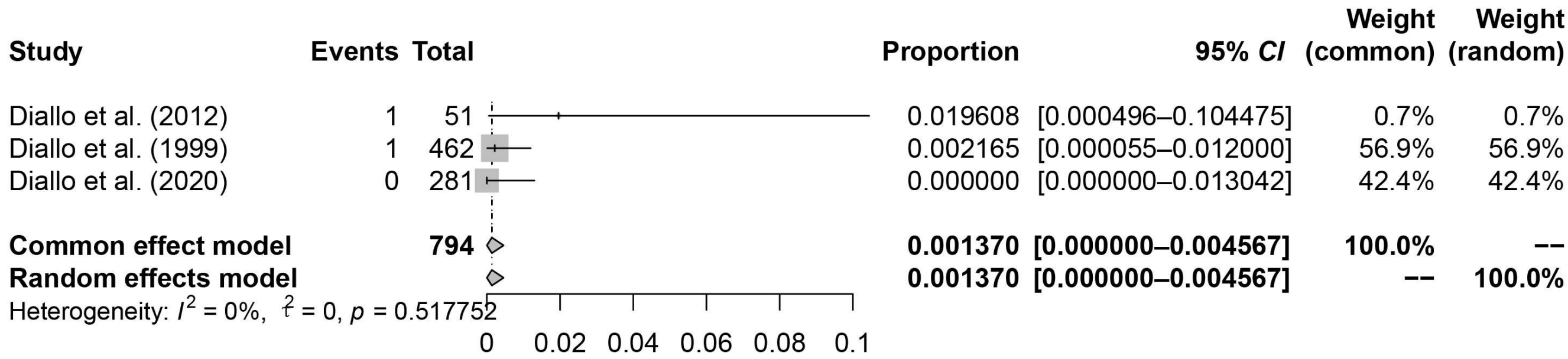

*Culex*_Dengue virus

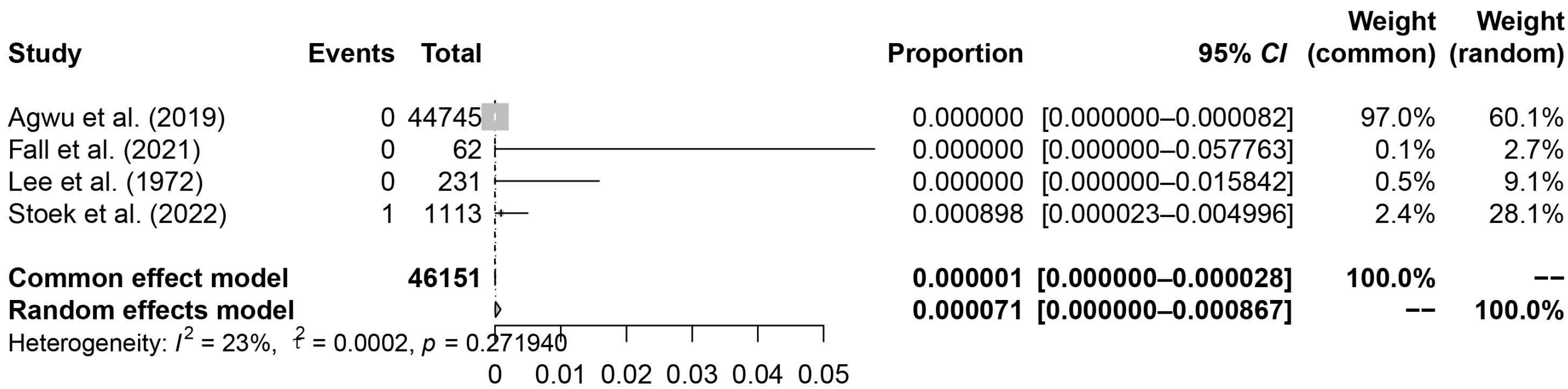

*Culex*_Rift valley fever virus

Study	Events	Total	Proportion	95% *CI*	Weight (common)	Weight (random)
Diallo et al. (2005)	0	2551	0.000000	[0.000000–0.001445]	2.2%	16.6%
Ba et al. (2012)	32	30761	0.001040	[0.000712–0.001468]	5.0%	17.2%
Diallo et al. (2000)	36	14937	0.002410	[0.001689–0.003335]	1.1%	15.5%
Diallo et al. (2005)	63	29018	0.002171	[0.001669–0.002777]	2.3%	16.6%
Ndiaye et al. (2018)	0	16200	0.000000	[0.000000–0.000228]	89.0%	17.7%
Stoek et al. (2022)	0	1113	0.000000	[0.000000–0.003309]	0.4%	13.0%
Wilson et al. (1994)	0	334	0.000000	[0.000000–0.010984]	0.0%	3.5%
**Common effect model**		**94914**	**0.000127**	**[0.000046–0.000208]**	**100.0%**	**--**
**Random effects model**			**0.000912**	**[0.000048–0.001775]**	**--**	**100.0%**

Heterogeneity: I^2 = 95%, τ^2 < 0.0001, p < 0.000001

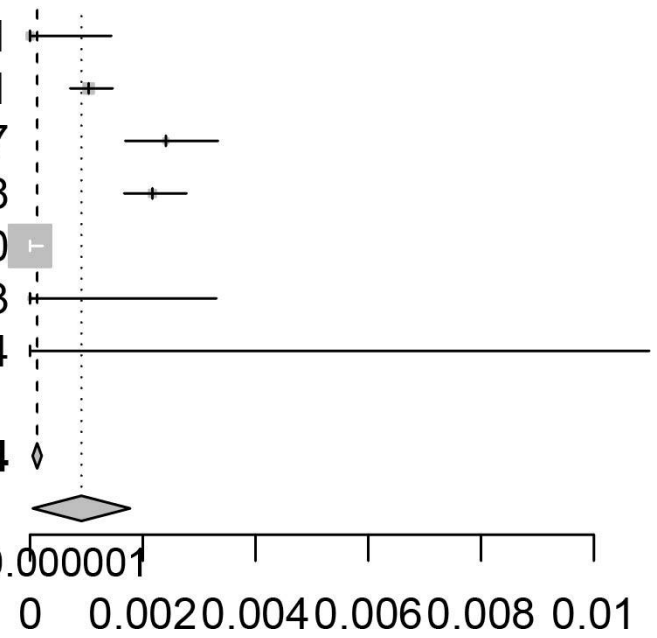

*Culex*_Sanar virus

Study	Events	Total	Proportion	95% *CI*	Weight (common)	Weight (random)
Diallo et al. (2005)	67	25158	0.002663	[0.002064–0.003381]	15.3%	49.2%
Ndiaye et al. (2018)	5	16200	0.000309	[0.000100–0.000720]	84.7%	50.8%
**Common effect model**		**41358**	**0.000668**	**[0.000420–0.000917]**	**100.0%**	**--**
**Random effects model**			**0.001468**	**[0.000000–0.003775]**	**--**	**100.0%**

Heterogeneity: I^2 = 98%, τ^2 < 0.0001, p < 0.000001

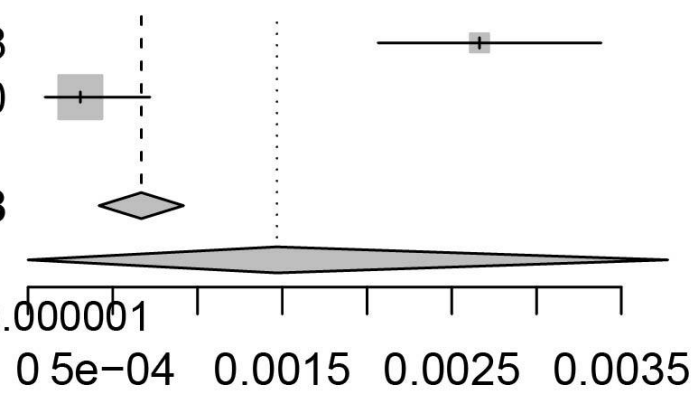

*Culex*_Usutu virus

Study	Events	Total	Proportion	95% *CI*	Weight (common)	Weight (random)
Ndiaye et al. (2018)	11	17491	0.000629	[0.000314–0.001125]	90.9%	90.9%
Tinto et al. (2022)	0	1176	0.000000	[0.000000–0.003132]	9.1%	9.1%
**Common effect model**		**18667**	**0.000572**	**[0.000218–0.000926]**	**100.0%**	**--**
**Random effects model**			**0.000572**	**[0.000218–0.000926]**	**--**	**100.0%**

Heterogeneity: I^2 = 0%, τ^2 = 0, p = 0.318041

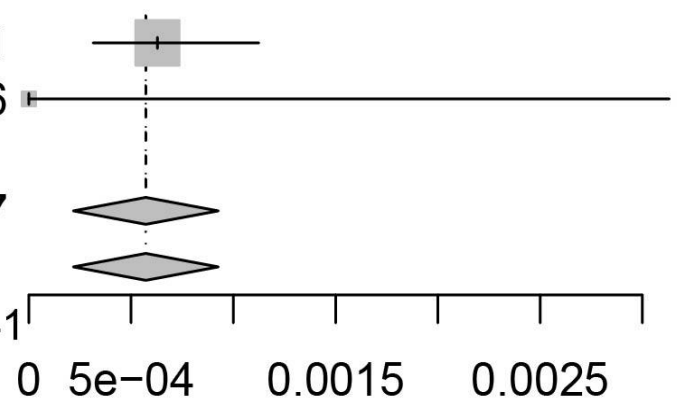

*Culex*_West Nile virus

Study	Events	Total	Proportion	95% *CI*	Weight (common)	Weight (random)
Fall et al. (2021)	0	43	0.000000	[0.000000–0.082211]	0.0%	0.2%
Diallo et al. (2005)	0	25158	0.000000	[0.000000–0.000147]	98.9%	35.8%
Fall et al. (2021)	0	19	0.000000	[0.000000–0.176467]	0.0%	0.0%
Ndiaye et al. (2018)	29	17491	0.001658	[0.001111–0.002380]	0.8%	33.0%
Tinto et al. (2022)	0	1176	0.000000	[0.000000–0.003132]	0.2%	27.0%
Wilson et al. (1994)	1	334	0.002994	[0.000076–0.016568]	0.0%	4.0%
**Common effect model**		**44221**	**0.000014**	**[0.000000–0.000069]**	**100.0%**	**--**
**Random effects model**			**0.000666**	**[0.000000–0.001903]**	**--**	**100.0%**

Heterogeneity: I^2 = 83%, τ^2 < 0.0001, p = 0.000016

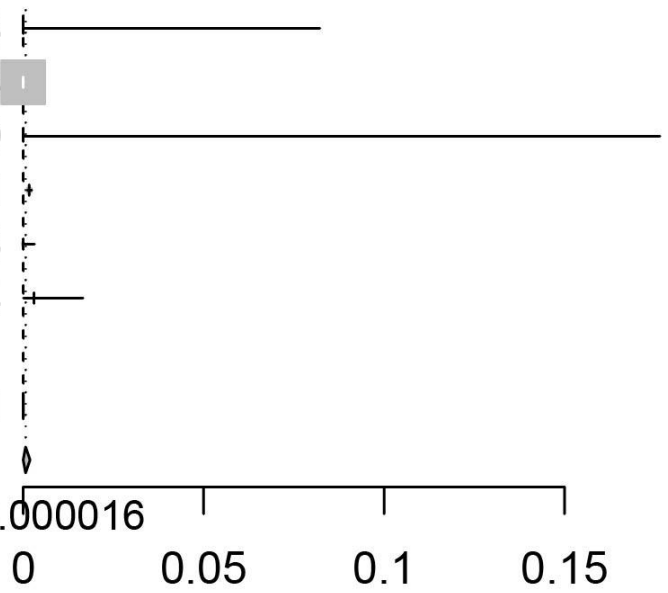

Culex_Wuchereria bancrofti

Study	Events	Total	Proportion	95% *CI*	Weight (common)	Weight (random)
Gbakima et al. (2005)	0	301	0.000000	[0.000000–0.012181]	0.2%	21.9%
Pam et al. (2017)	42	9481	0.004430	[0.003195–0.005983]	2.0%	37.7%
de Souza et al. (2014)	2	14342	0.000139	[0.000017–0.000504]	97.8%	40.3%
**Common effect model**		**24124**	**0.000227**	**[0.000036–0.000418]**	**100.0%**	**--**
**Random effects model**			**0.001727**	**[0.000000–0.004904]**	**--**	**100.0%**

Heterogeneity: I^2 = 95%, τ^2 < 0.0001, p < 0.000001

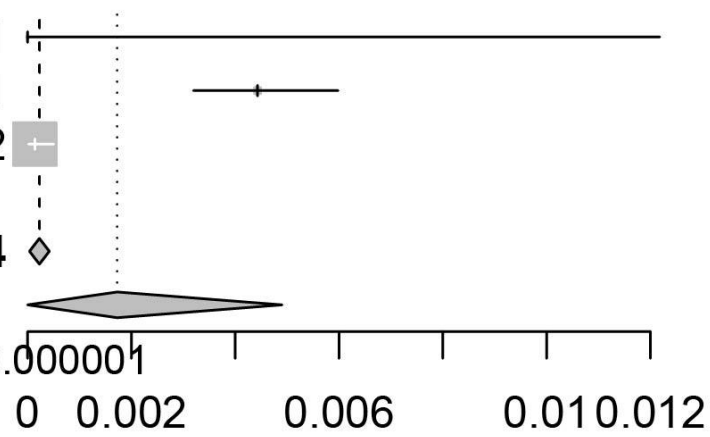

*Culex*_Zika virus

Study	Events	Total	Proportion	95% *CI*	Weight (common)	Weight (random)
Diallo et al. (2014)	1	22	0.045455	[0.001150–0.228444]	3.7%	3.7%
Diallo et al. (2020)	0	281	0.000000	[0.000000–0.013042]	47.1%	47.1%
Fall et al. (2021)	0	62	0.000000	[0.000000–0.057763]	10.4%	10.4%
Lee et al. (1972)	0	231	0.000000	[0.000000–0.015842]	38.8%	38.8%
**Common effect model**		**596**	**0.000063**	**[0.000000–0.002309]**	**100.0%**	**--**
**Random effects model**			**0.000063**	**[0.000000–0.002319]**	**--**	**100.0%**

Heterogeneity: I^2 = 23%, τ^2 < 0.0001, p = 0.271105

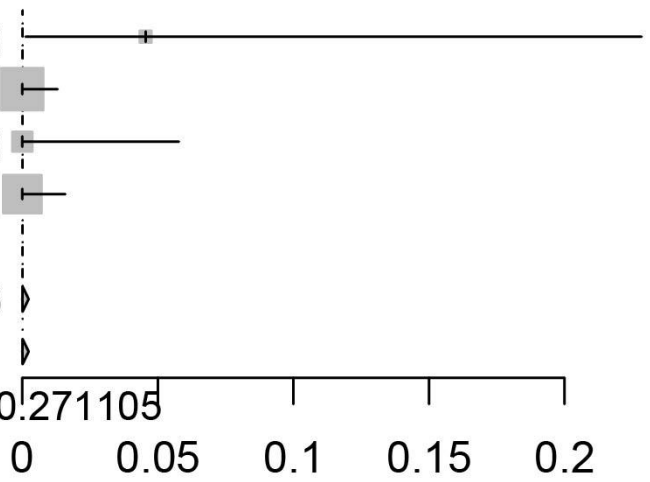

*Culex antennatus*_Bagaza virus

Study	Events	Total	Proportion	95% *CI*	Weight (common)	Weight (random)
Ndiaye et al. (2018)	3	1308	0.002294	[0.000473–0.006688]	99.5%	99.5%
Wilson et al. (1994)	0	36	0.000000	[0.000000–0.097394]	0.5%	0.5%
**Common effect model**		**1344**	**0.002282**	**[0.000000–0.004869]**	**100.0%**	**--**
**Random effects model**			**0.002282**	**[0.000000–0.004869]**	**--**	**100.0%**

Heterogeneity: I^2 = 0%, τ^2 = 0, p = 0.904055

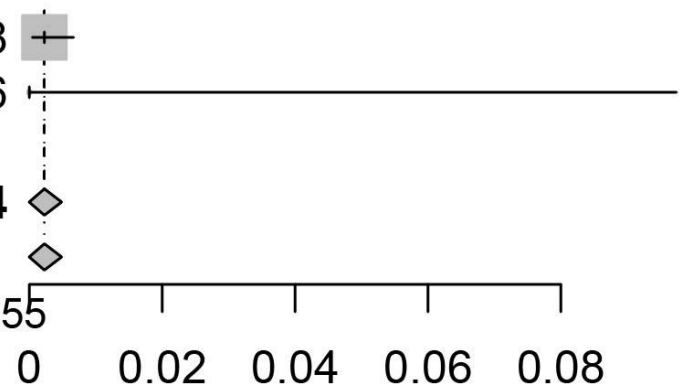

*Culex antennatus*_West Nile Virus

Study	Events	Total	Proportion	95% *CI*	Weight (common)	Weight (random)
Fall et al. (2021)	0	1	0.000000	[0.000000–0.975000]	0.0%	0.0%
Ndiaye et al. (2018)	3	1308	0.002294	[0.000473–0.006688]	99.8%	99.8%
Wilson et al. (1994)	1	36	0.027778	[0.000703–0.145289]	0.2%	0.2%
**Common effect model**		**1345**	**0.002353**	**[0.000000–0.004942]**	**100.0%**	**--**
**Random effects model**			**0.002353**	**[0.000000–0.004942]**	**--**	**100.0%**

Heterogeneity: $I^2 = 0\%$, $\tau^2 = 0$, $p = 0.649283$

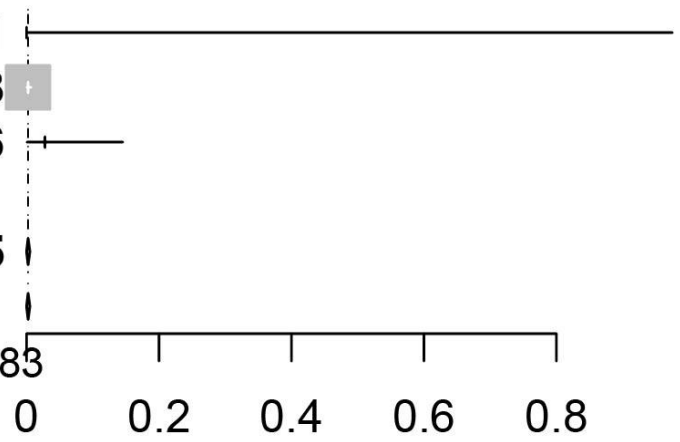

*Culex bitaeniorhynchus*_Chikungunya virus

Study	Events	Total	Proportion	95% *CI*	Weight (common)	Weight (random)
Diallo et al. (1999)	1	462	0.002165	[0.000055–0.012000]	97.9%	97.9%
Diallo et al. (2020)	0	47	0.000000	[0.000000–0.075486]	2.1%	2.1%
**Common effect model**		**509**	**0.002118**	**[0.000000–0.006311]**	**100.0%**	**--**
**Random effects model**			**0.002118**	**[0.000000–0.006311]**	**--**	**100.0%**

Heterogeneity: $I^2 = 0\%$, $\tau^2 = 0$, $p = 0.883826$

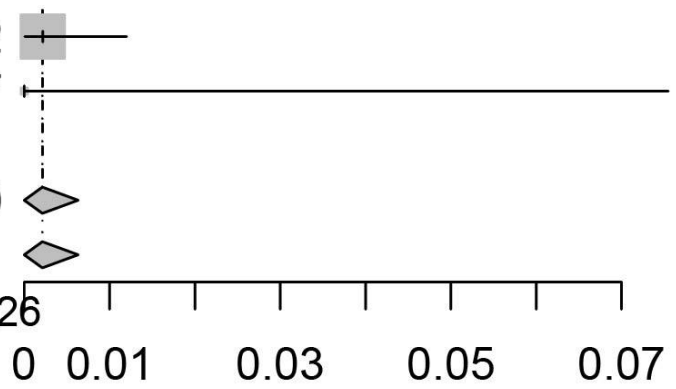

*Culex neavei*_Bagaza virus

Study	Events	Total	Proportion	95% *CI*	Weight (common)	Weight (random)
Diallo et al. (2005)	18	2551	0.007056	[0.004187–0.011129]	5.0%	46.0%
Ndiaye et al. (2018)	2	3699	0.000541	[0.000065–0.001952]	94.9%	52.4%
Wilson et al. (1994)	0	28	0.000000	[0.000000–0.123436]	0.0%	1.6%
**Common effect model**		**6278**	**0.000870**	**[0.000140–0.001599]**	**100.0%**	**--**
**Random effects model**			**0.003529**	**[0.000000–0.009680]**	**--**	**100.0%**

Heterogeneity: $I^2 = 86\%$, $\tau^2 < 0.0001$, $p = 0.000650$

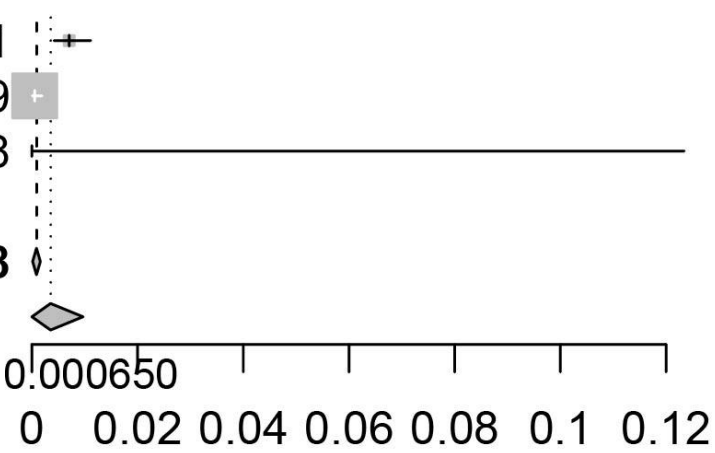

*Culex neavei*_Sanar virus

Study	Events	Total	Proportion	95% *CI*	Weight (common)	Weight (random)
Diallo et al. (2005)	0	831	0.000000	[0.000000–0.004429]	28.8%	31.6%
Ndiaye et al. (2018)	4	3699	0.001081	[0.000295–0.002766]	71.2%	68.4%
**Common effect model**		**4530**	**0.000770**	**[0.000000–0.001664]**	**100.0%**	**--**
**Random effects model**			**0.000739**	**[0.000000–0.001725]**	**--**	**100.0%**

Heterogeneity: $I^2 = 13\%$, $\tau^2 < 0.0001$, $p = 0.282852$

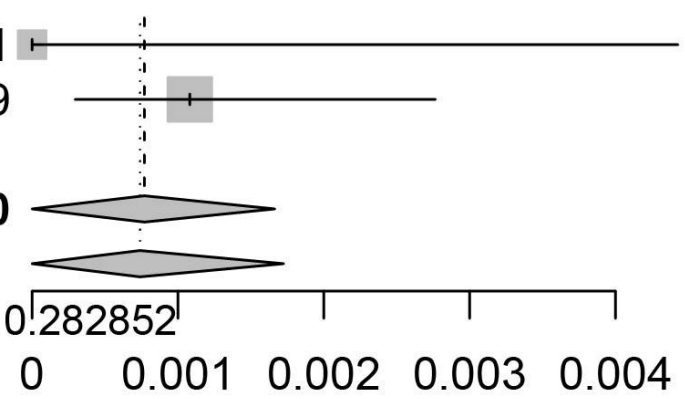

*Culex neavei*_West Nile virus

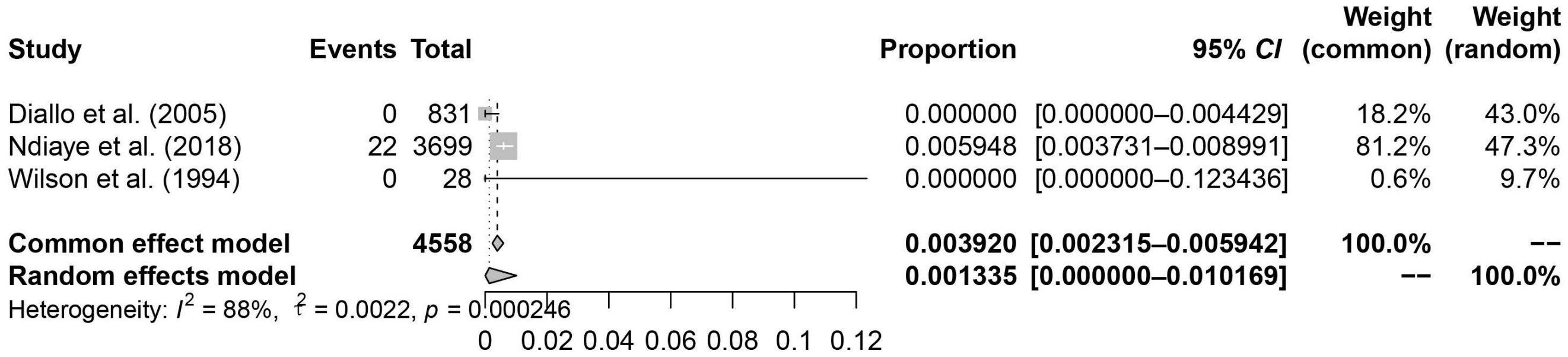

*Culex perfuscus*_Zika virus

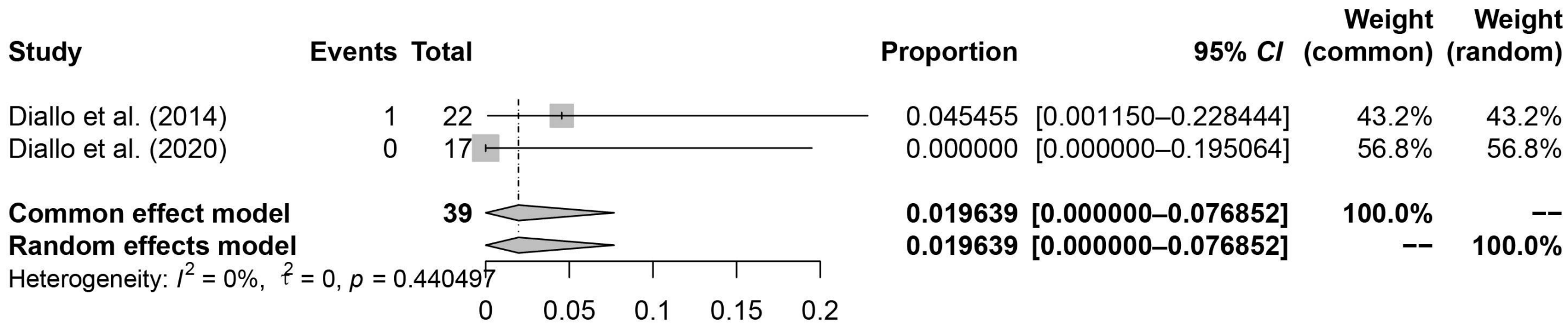

*Culex poicilipes*_Bagaza virus

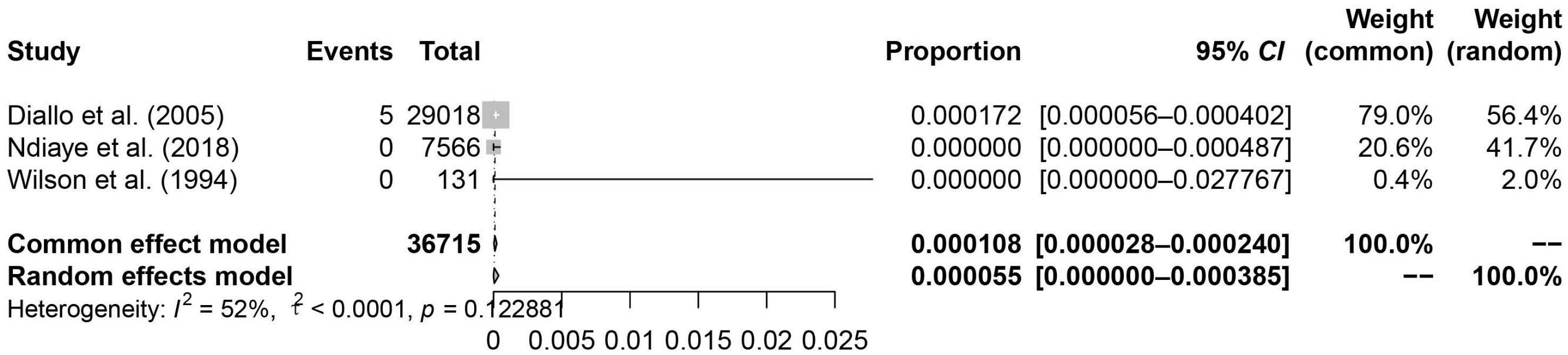

*Culex poicilipes*_Chikungunya virus

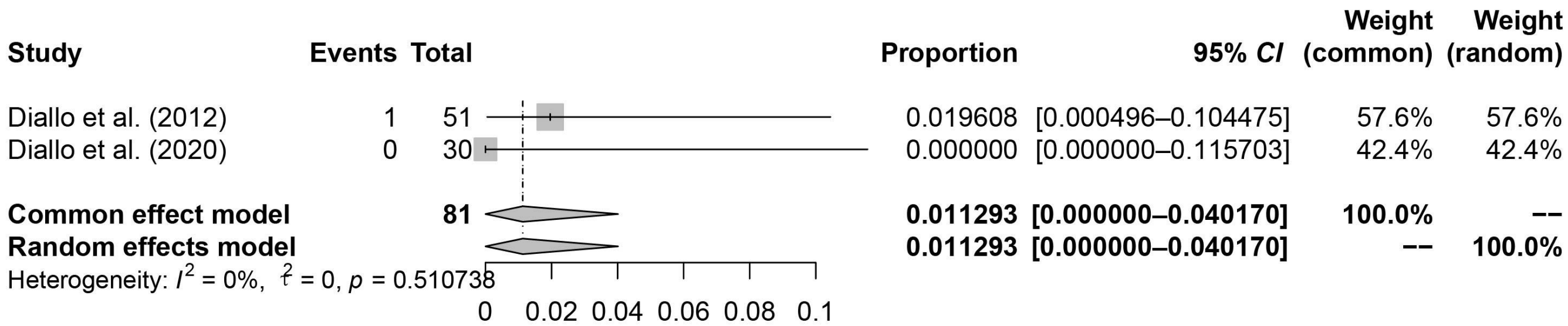

滚滚长江东逝水

*Culex poicilipes*_Rift Valley fever virus

Study	Events	Total	Proportion	95% *CI*	Weight (common)	Weight (random)
Diallo et al. (2005)	63	29018	0.002171	[0.001669–0.002777]	8.1%	22.1%
Ba et al. (2012)	32	30761	0.001040	[0.000712–0.001468]	18.0%	22.8%
Diallo et al. (2000)	36	14937	0.002410	[0.001689–0.003335]	3.8%	20.7%
Ndiaye et al. (2018)	0	7566	0.000000	[0.000000–0.000487]	69.7%	23.3%
Stoek et al. (2022)	0	570	0.000000	[0.000000–0.006451]	0.4%	10.2%
Wilson et al. (1994)	0	131	0.000000	[0.000000–0.027767]	0.0%	0.9%
**Common effect model**		**82983**	**0.000455**	**[0.000302; 0.000608]**	**100.0%**	**--**
**Random effects model**			**0.001215**	**[0.000183; 0.002246]**	**--**	**100.0%**

Heterogeneity: I^2 = 95%, τ^2 < 0.0001, p < 0.000001

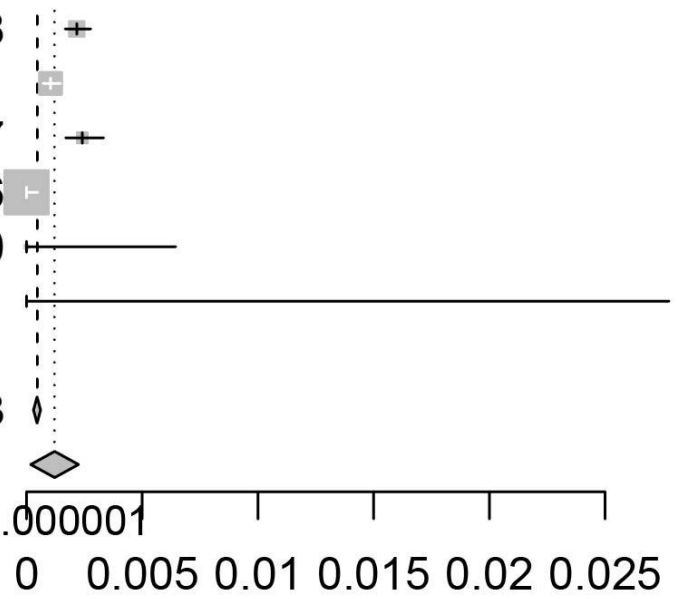

*Culex poicilipes*_Sanar virus

Study	Events	Total	Proportion	95% *CI*	Weight (common)	Weight (random)
Diallo et al. (2005)	67	24327	0.002754	[0.002135–0.003496]	7.2%	49.3%
Ndiaye et al. (2018)	0	7566	0.000000	[0.000000–0.000487]	92.8%	50.7%
**Common effect model**		**31893**	**0.000198**	**[0.000021–0.000374]**	**100.0%**	**--**
**Random effects model**			**0.001358**	**[0.000000–0.004057]**	**--**	**100.0%**

Heterogeneity: I^2 = 98%, τ^2 < 0.0001, p < 0.000001

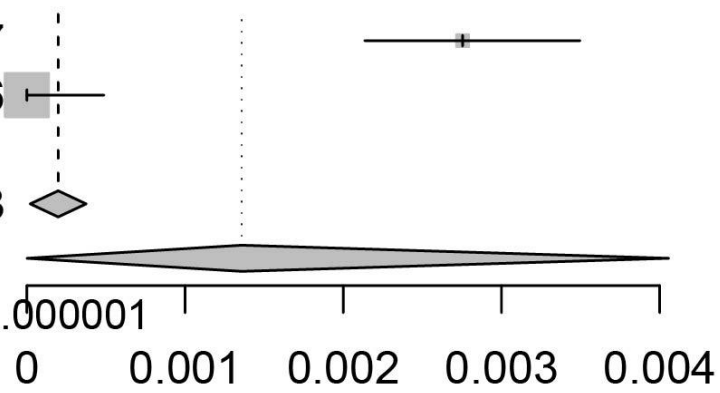

*Culex poicilipes*_West Nile virus

Study	Events	Total	Proportion	95% *CI*	Weight (common)	Weight (random)
Diallo et al. (2005)	0	24327	0.000000	[0.000000–0.000152]	76.0%	58.2%
Ndiaye et al. (2018)	1	7566	0.000132	[0.000003–0.000736]	23.6%	40.3%
Wilson et al. (1994)	0	131	0.000000	[0.000000–0.027767]	0.4%	1.5%
**Common effect model**		**32024**	**0.000007**	**[0.000000–0.000067]**	**100.0%**	**--**
**Random effects model**			**0.000021**	**[0.000000–0.000235]**	**--**	**100.0%**

Heterogeneity: I^2 = 35%, τ^2 < 0.0001, p = 0.217068

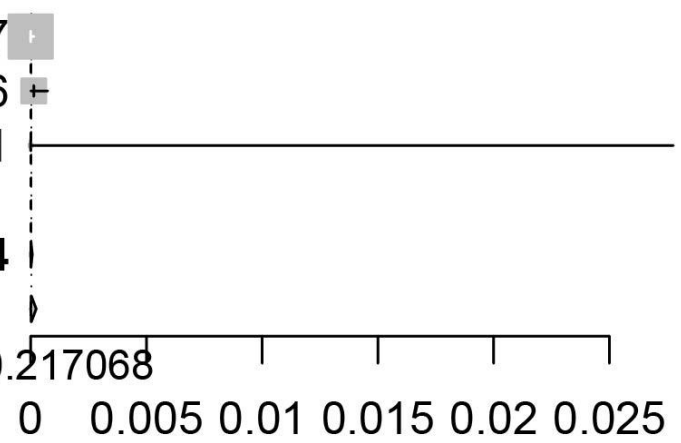

*Culex quinquefasciatus*_Dengue virus

Study	Events	Total	Proportion	95% *CI*	Weight (common)	Weight (random)
Agwu et al. (2019)	0	44476	0.000000	[0.000000–0.000083]	98.8%	61.1%
Fall et al. (2021)	0	18	0.000000	[0.000000–0.185302]	0.0%	2.4%
Lee et al. (1972)	0	44	0.000000	[0.000000–0.080420]	0.1%	5.5%
Stoek et al. (2022)	1	458	0.002183	[0.000055–0.012105]	1.0%	31.0%
**Common effect model**		**44996**	**0.000000**	**[0.000000–0.000026]**	**100.0%**	**--**
**Random effects model**			**0.000210**	**[0.000000–0.002565]**	**--**	**100.0%**

Heterogeneity: I^2 = 24%, τ^2 = 0.0006, p = 0.265578

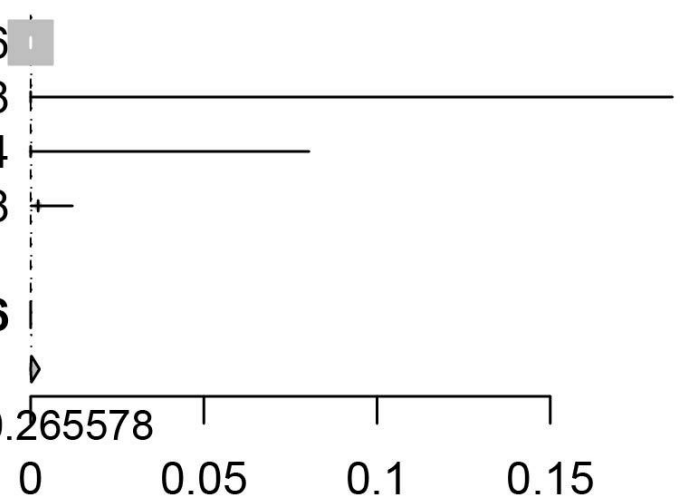

*Culex quinquefasciatus*_West Nile Virus

Study	Events	Total	Proportion	95% *CI*	Weight (common)	Weight (random)
Fall et al. (2021)	0	18	0.000000	[0.000000–0.185302]	1.4%	10.6%
Ndiaye et al. (2018)	1	80	0.012500	[0.000316–0.067688]	6.3%	30.0%
Tinto et al. (2022)	0	1176	0.000000	[0.000000–0.003132]	92.3%	59.4%
**Common effect model**		**1274**	**0.000049**	**[0.000000–0.001189]**	**100.0%**	**--**
**Random effects model**			**0.001130**	**[0.000000–0.013402]**	**--**	**100.0%**

Heterogeneity: I^2 = 47%, τ^2 = 0.0028, p = 0.152244

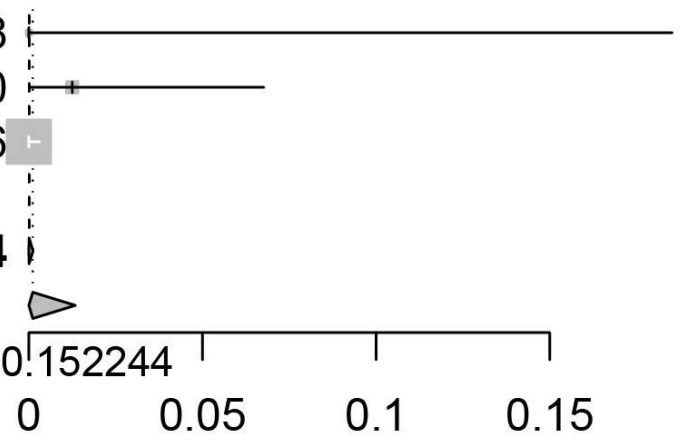

Culex quinquefasciatus_Wuchereria bancrofti

Study	Events	Total	Proportion	95% *CI*	Weight (common)	Weight (random)
Gbakima et al. (2005)	0	301	0.000000	[0.000000–0.012181]	0.2%	21.9%
Pam et al. (2017)	42	9481	0.004430	[0.003195–0.005983]	2.0%	37.7%
de Souza et al. (2014)	2	14342	0.000139	[0.000017–0.000504]	97.8%	40.3%
**Common effect model**		**24124**	**0.000227**	**[0.000036–0.000418]**	**100.0%**	**--**
**Random effects model**			**0.001727**	**[0.000000–0.004904]**	**--**	**100.0%**

Heterogeneity: I^2 = 95%, τ^2 < 0.0001, p < 0.000001

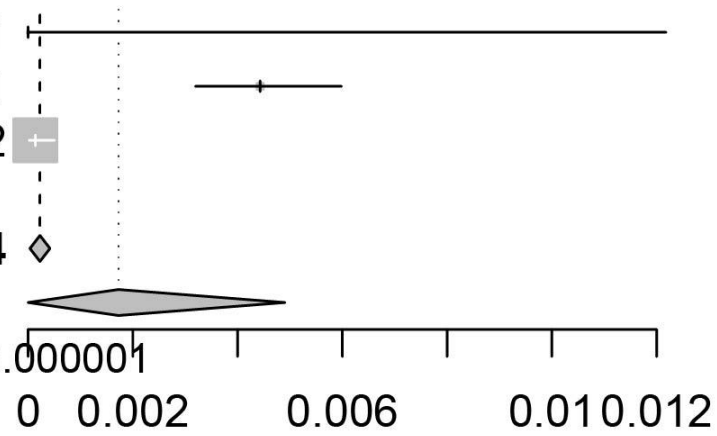

*Culex tritaeniorhynchus*_West Nile virus

Study	Events	Total	Proportion	95% *CI*	Weight (common)	Weight (random)
Ndiaye et al. (2018)	1	1297	0.000771	[0.000020–0.004288]	99.7%	99.7%
Wilson et al. (1994)	0	52	0.000000	[0.000000–0.068482]	0.3%	0.3%
**Common effect model**		**1349**	**0.000768**	**[0.000000–0.002276]**	**100.0%**	**--**
**Random effects model**			**0.000768**	**[0.000000–0.002276]**	**--**	**100.0%**

Heterogeneity: I^2 = 0%, τ^2 = 0, p = 0.953775

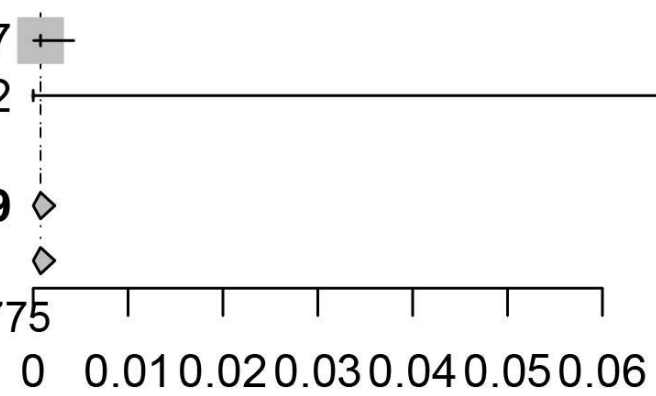

Supplementary Fig. 12. Meta-analysis of the positivity rate of each *Mansonia*-associated pathogen. The heterogeneity of combined studies was quantified by I^2 statistic. The random effect model would be applied if $I^2 > 50\%$; Otherwise, the fixed effect model would be applied.

***Mansonia*_Chikungunya virus**

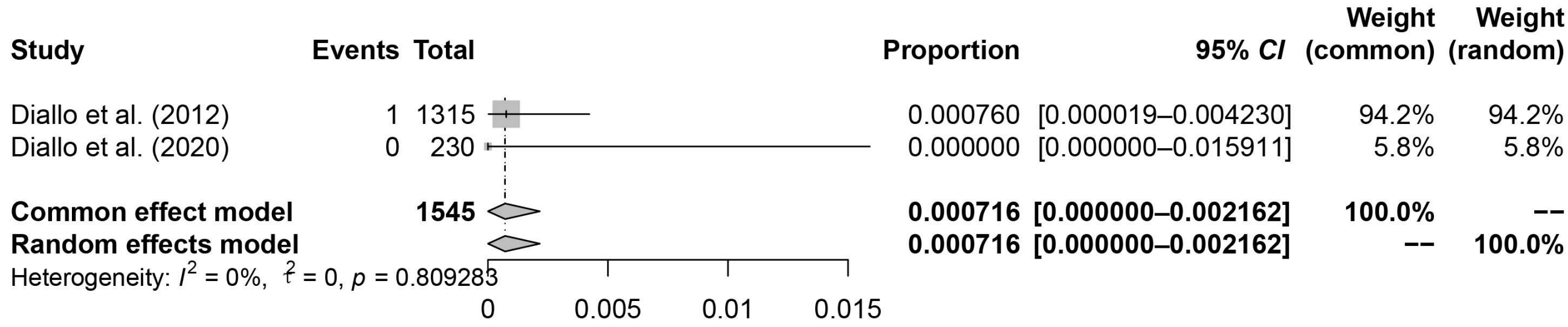

***Mansonia*_Dengue virus**

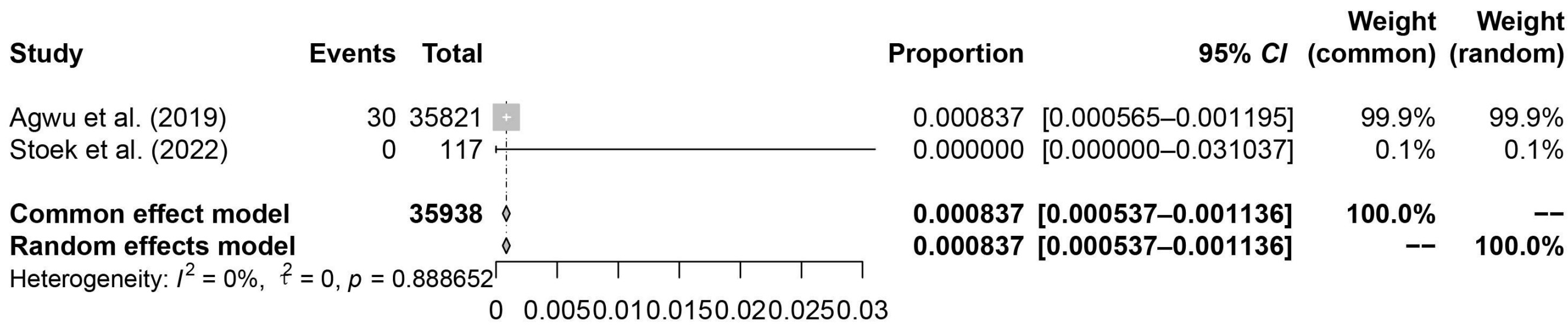

***Mansonia*_Rift valley fever virus**

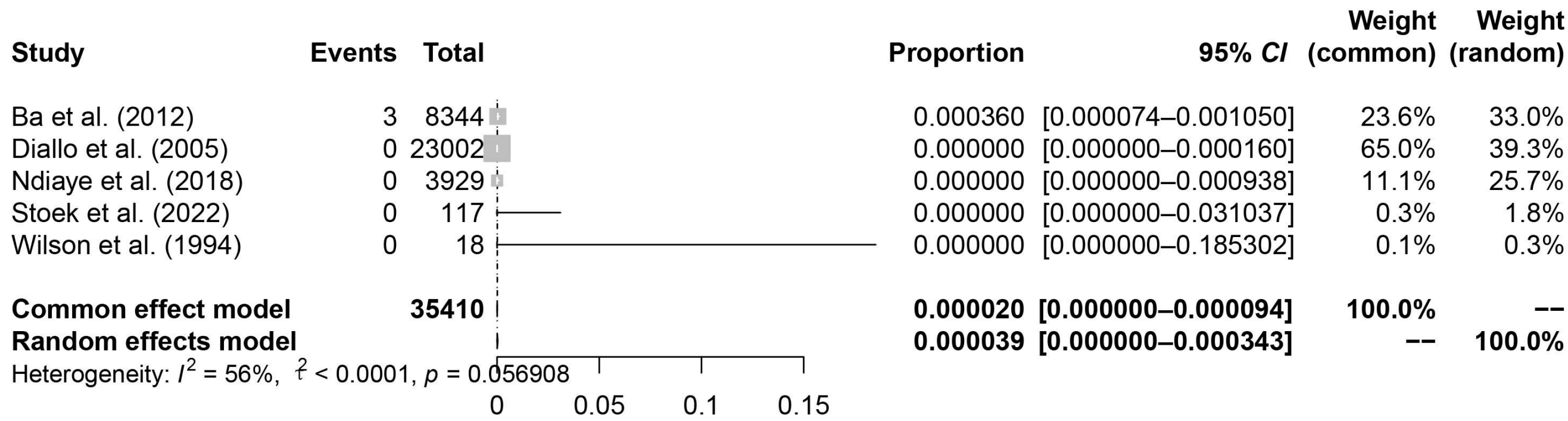

***Mansonia*_Sanar virus**

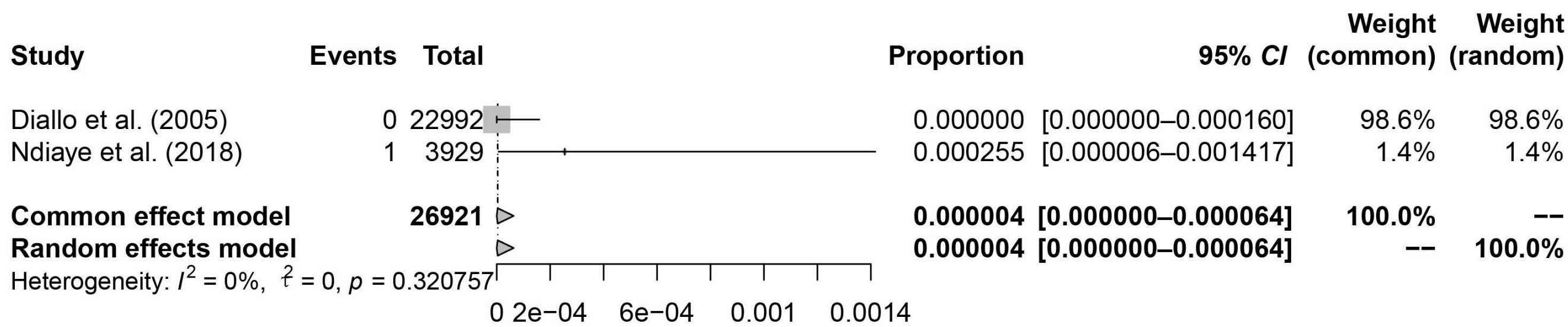

*Mansonia africana*_Rift valley fever virus

Study	Events	Total	Proportion	95% *CI*	Weight (common)	Weight (random)
Ba et al. (2012)	2	6285	0.000318	[0.000039–0.001149]	90.5%	90.5%
Ndiaye et al. (2018)	0	659	0.000000	[0.000000–0.005582]	9.5%	9.5%
Wilson et al. (1994)	0	4	0.000000	[0.000000–0.602365]	0.1%	0.1%
**Common effect model**		**6948**	**0.000260**	**[0.000019–0.000778]**	**100.0%**	**--**
**Random effects model**			**0.000260**	**[0.000019–0.000778]**	**--**	**100.0%**

Heterogeneity: $I^2 = 0\%$, $\tau^2 = 0$, $p = 0.682677$

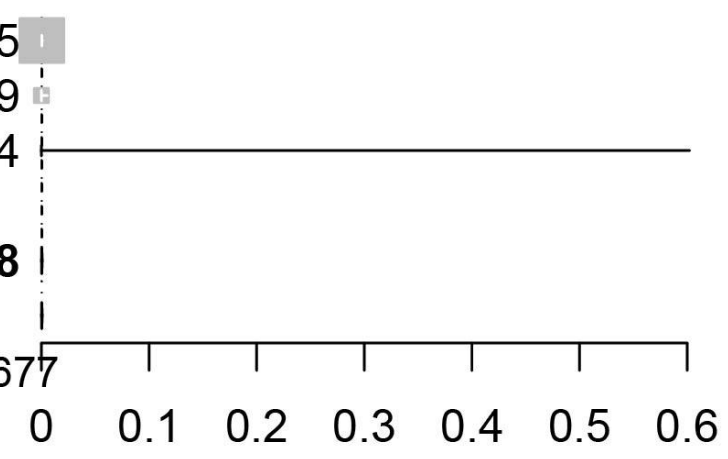

*Mansonia uniformis*_Chikungunya virus

Study	Events	Total	Proportion	95% *CI*	Weight (common)	Weight (random)
Diallo et al. (2012)	1	1315	0.000760	[0.000019–0.004230]	96.3%	96.3%
Diallo et al. (2020)	0	182	0.000000	[0.000000–0.020065]	3.7%	3.7%
**Common effect model**		**1497**	**0.000732**	**[0.000000–0.002194]**	**100.0%**	**--**
**Random effects model**			**0.000732**	**[0.000000–0.002194]**	**--**	**100.0%**

Heterogeneity: $I^2 = 0\%$, $\tau^2 = 0$, $p = 0.846677$

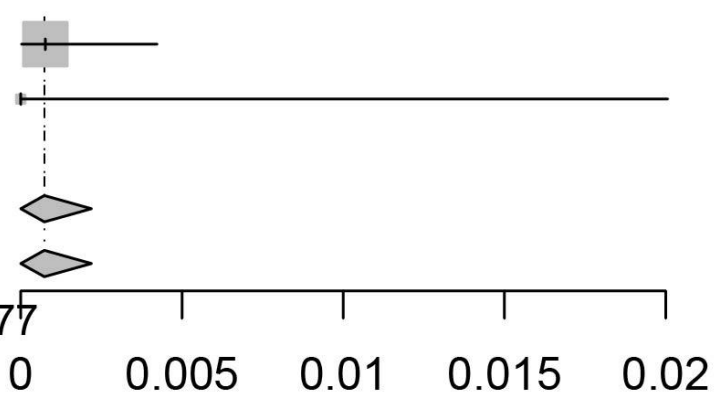

*Mansonia uniformis*_Rift Valley fever virus

Study	Events	Total	Proportion	95% *CI*	Weight (common)	Weight (random)
Diallo et al. (2005)	0	23002	0.000000	[0.000000–0.000160]	80.8%	61.8%
Ba et al. (2012)	1	2059	0.000486	[0.000012–0.002703]	7.2%	15.1%
Ndiaye et al. (2018)	0	3270	0.000000	[0.000000–0.001127]	11.5%	21.9%
Stoek et al. (2022)	0	117	0.000000	[0.000000–0.031037]	0.4%	1.0%
Wilson et al. (1994)	0	14	0.000000	[0.000000–0.231636]	0.0%	0.1%
**Common effect model**		**28462**	**0.000003**	**[0.000000–0.000055]**	**100.0%**	**--**
**Random effects model**			**0.000011**	**[0.000000–0.000158]**	**--**	**100.0%**

Heterogeneity: $I^2 = 0\%$, $\tau^2 < 0.0001$, $p = 0.446494$

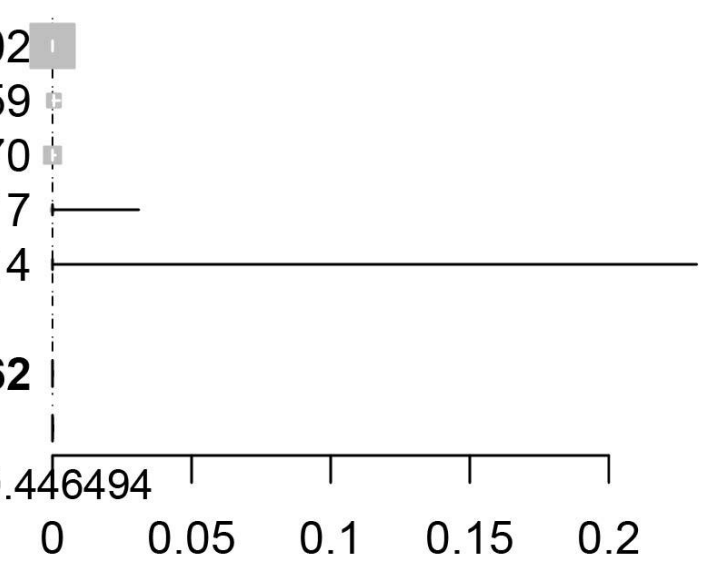

*Mansonia uniformis*_West Nile virus

Study	Events	Total	Proportion	95% *CI*	Weight (common)	Weight (random)
Diallo et al. (2005)	30	22992	0.001305	[0.000881–0.001862]	87.5%	51.8%
Ndiaye et al. (2018)	0	3270	0.000000	[0.000000–0.001127]	12.4%	46.6%
Wilson et al. (1994)	0	14	0.000000	[0.000000–0.231636]	0.1%	1.7%
**Common effect model**		**26276**	**0.000999**	**[0.000654–0.001418]**	**100.0%**	**--**
**Random effects model**			**0.000350**	**[0.000000–0.002804]**	**--**	**100.0%**

Heterogeneity: $I^2 = 87\%$, $\tau^2 = 0.0006$, $p = 0.000552$

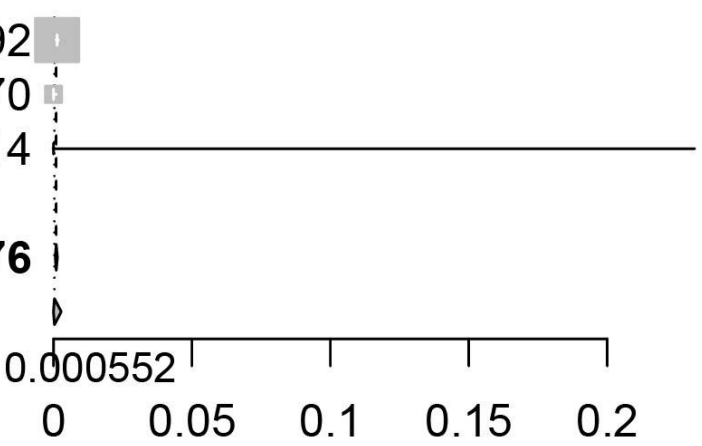

*Mansonia uniformis*_Zika virus

Study	Events	Total	Proportion	95% *CI*	Weight (common)	Weight (random)
Diallo et al. (2014)	1	281	0.003559	[0.000090–0.019667]	70.4%	70.4%
Diallo et al. (2020)	1	182	0.005495	[0.000139–0.030232]	29.6%	29.6%
**Common effect model**		**463**	**0.004132**	**[0.000000–0.009974]**	**100.0%**	**--**
**Random effects model**			**0.004132**	**[0.000000–0.009974]**	**--**	**100.0%**

Heterogeneity: $I^2 = 0\%$, $\tau^2 = 0$, $p = 0.766896$

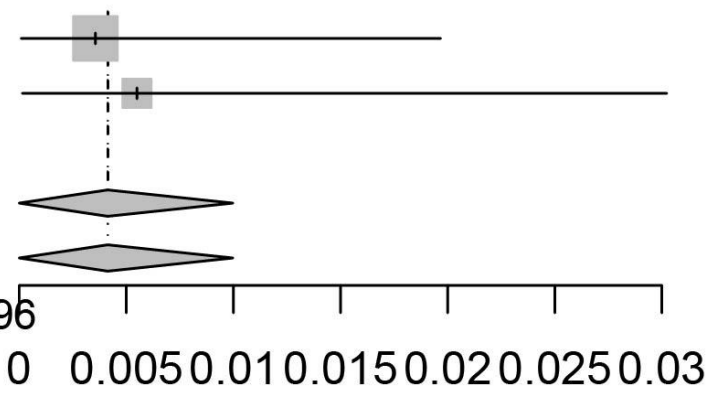

*Mansonia*_Zika virus

Study	Events	Total	Proportion	95% *CI*	Weight (common)	Weight (random)
Diallo et al. (2014)	1	281	0.003559	[0.000090–0.019667]	59.9%	59.9%
Diallo et al. (2020)	1	230	0.004348	[0.000110–0.023985]	40.1%	40.1%
**Common effect model**		**511**	**0.003875**	**[0.000000–0.009262]**	**100.0%**	**--**
**Random effects model**			**0.003875**	**[0.000000–0.009262]**	**--**	**100.0%**

Heterogeneity: $I^2 = 0\%$, $\tau^2 = 0$, $p = 0.888083$

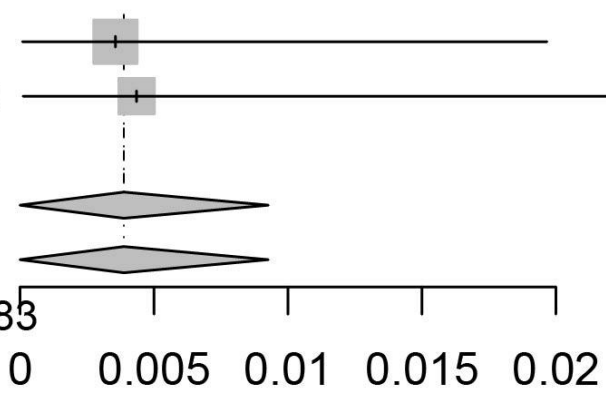

Supplementary Fig. 13. Meta-analysis of the positivity rate of each *Mimomyia*-associated pathogen. The heterogeneity of combined studies was quantified by I^2 statistic. The random effect model would be applied if $I^2 > 50\%$; Otherwise, the fixed effect model would be applied.

***Mimomyia*_Bagaza virus**

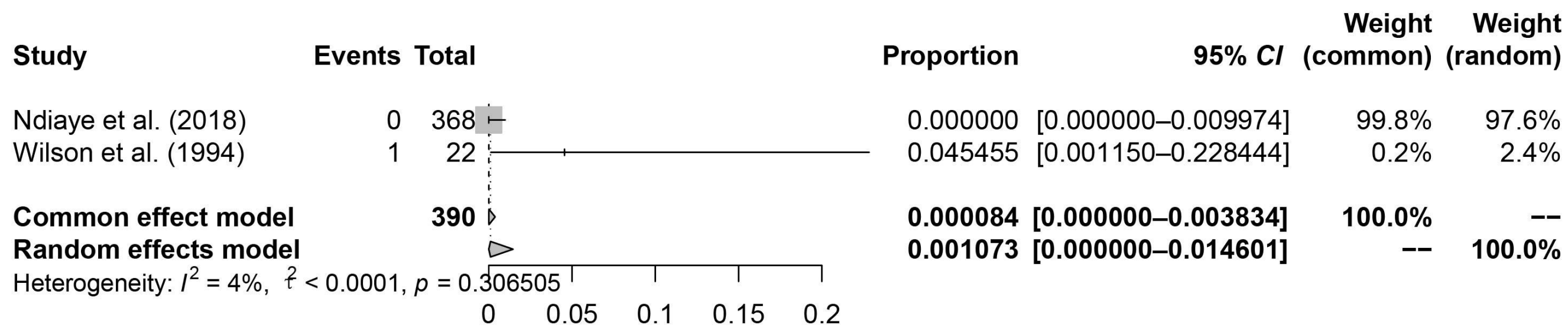

Supplementary Text 2. Evidence for human infection of mosquito-borne pathogens in countries of West Africa

	Dengue virus	Spondweni virus	Usutu virus	Wesselsbron virus	West Nile virus	Yellow fever virus	Zika virus
Benin	1,2					2-5	
Burkina Faso	2-4,6-9	10	11,12		12	2-4,12-14	15
Côte d'Ivoire	2,3,16					2-4,13,17	
Cape Verde	2,3,18						2,19,20
Gambia						3,21-23	24
Ghana	25-27				28,29	4,13,30	28,31,32
Guinea	33,34				33,35	2-4,13	36
Guinea-Bissau							37
Liberia						2-4,13,38	
Mali	2,13				39	2-4,13,40	2,24
Mauritania	2,41				3,42	3	
Niger	2,43				3	2-4	
Nigeria	44-47	48,49	50	51-53	54,55	3,4,13,56	45,57,58
Senegal	2,59-61			62,63	64	2-4,13,65,66	2
Sierra Leone	67,68				13,67	2-4,13,67	
Togo	2,69					2,4,13,70	

The evidence meeting any of the following criteria was included. (1) Outbreak or epidemic caused by the pathogen; (2) isolation of pathogens from human samples or molecular detection and sequence determination; (3) serological evidence of pathogen infection in humans.

	Bunyamwera virus	Ngari virus	Nyando virus	Pongola virus	Tataguine virus	Rift valley fever virus	Chikungunya virus	Sindbis virus
Benin							2,71	
Burkina Faso							71	
Côte d'Ivoire								
Cape Verde							2	
Gambia						2		
Ghana							72	
Guinea						33	33,34	34
Guinea-Bissau								
Liberia								
Mali						2,13,73	39	
Mauritania						4,13,74-76	77	
Niger						2,4,13,78		
Nigeria	45,79			45	80	81-84	45,85	53,86
Senegal	62	87				2,4,13,88	4,13,89,90	62
Sierra Leone						67	67,91	11,12
Togo								

	Wuchereria bancrofti	*Plasmodium falciparum*	*Plasmodium malariae*	*Plasmodium ovale*	*Plasmodium vivax*
Benin	92	93	94	95	96
Burkina Faso	97	93	98	98,99	100
Côte d'Ivoire	101	93	102	103	104
Cape Verde	105	93	106		
Gambia	107	93	108	99,109,110	
Ghana	111	93	112	112	104
Guinea	105	93	113	100	
Guinea-Bissau	105	93	114	114	
Liberia	105	93	115	116	104
Mali	105	93	117	99	118
Mauritania	119	120	121	121	122,123
Niger	124	93	125	125	125
Nigeria	13	93	126	104	126,127
Senegal	128	93	129,130	129	131
Sierra Leone	132	93	133	133	104
Togo	134	93	135	135	104

References

1. Gautret P, Botelho-Nevers E, Charrel RN, Parola P. Dengue virus infections in travellers returning from Benin to France, July-August 2010. *Euro Surveill* 2010; **15**(36).

2. WHO. Surveillance and control of arboviral diseases in the WHO African Region. https://www.who.int/publications/i/item/9789240052918 (accessed Oct 10, 2023)

3. EM-DAT. the Emergency Events Database. https://www.emdat.be/ (accessed Oct 10, 2023).

4. WHO. Disease Outbreak News (DONs). https://www.who.int/emergencies/disease-outbreak-news (accessed Oct 10, 2023)

5. WHO. Yellow fever, 1996-1997 : Part I = Fièvre jaune, 1996-1997 : Partie I. https://iris.who.int/handle/10665/230623 (accessed Oct 10, 2023)

6. Ridde V, Agier I, Bonnet E, et al. Presence of three dengue serotypes in Ouagadougou (Burkina Faso): research and public health implications. *Infectious Diseases of Poverty* 2016; **5**(1).

7. Letizia AG, Pratt CB, Wiley MR, et al. Retrospective Genomic Characterization of a 2017 Dengue Virus Outbreak, Burkina Faso. *Emerg Infect Dis* 2022; **28**(6): 1198-210.

8. Ridde V, Agier I, Bonnet E, et al. Presence of three dengue serotypes in Ouagadougou (Burkina Faso): research and public health implications. *Infectious Diseases of Poverty* 2016; **5**(1).

9. Letizia AG, Pratt CB, Wiley MR, et al. Retrospective Genomic Characterization of a 2017 Dengue Virus Outbreak, Burkina Faso. *Emerg Infect Dis* 2022; **28**(6): 1198-210.

10. Wolfe M, Calisher C, McGuire K. SPONDWENI VIRUS INFECTION IN A FOREIGN RESIDENT OF UPPER VOLTA. *The Lancet* 1982; **320**(8311): 1306-8.

11. Nikolay B, Diallo M, Boye CSB, Sall AA. Usutu Virus in Africa. *Vector-Borne and Zoonotic Diseases* 2011; **11**(11): 1417-23.

12. Tinto B, Kaboré DPA, Kagoné TS, et al. Screening of Circulation of Usutu and West Nile Viruses: A One Health Approach in Humans, Domestic Animals and Mosquitoes in Burkina Faso, West Africa. *Microorganisms* 2022; **10**(10).

13. PROMED: Program for Monitoring Emerging Diseases. https://promedmail.org/promed-posts/ (accessed Oct 10, 2023).

14. Baudon D, Robert V, Roux J, et al. [The 1983 yellow fever epidemic in Burkina Faso]. *Bull World Health Organ* 1986; **64**(6): 873-82.

15. Tinto B, Kaboré DPA, Kania D, et al. Serological Evidence of Zika Virus Circulation in Burkina Faso. *Pathogens* 2022; **11**(7).

16. Dengue in Africa: emergence of DENV-3, Côte d'Ivoire, 2008. *Wkly Epidemiol Rec* 2009; **84**(11-12): 85-8.

17. Lhuillier M, Sarthou JL, Cordellier R, et al. Epidémie rurale de fièvre jaune avec transmission interhumaine en Côte d'Ivoire en 1982. *Bulletin of The World Health Organization* 1985; **63**: 527-36.

18. Franco L, Di Caro A, Carletti F, et al. Recent expansion of dengue virus serotype 3 in West Africa. *Eurosurveillance* 2010; **15**(7).

19. Lourenço J, de Lourdes Monteiro M, Valdez T, Monteiro Rodrigues J, Pybus O, Rodrigues Faria N. Epidemiology of the Zika Virus Outbreak in the Cabo Verde Islands, West Africa. *PLoS Curr* 2018; **10**.

20. Faye O, de Lourdes Monteiro M, Vrancken B, et al. Genomic Epidemiology of 2015–2016 Zika Virus Outbreak in Cape Verde. *Emerging Infectious Diseases* 2020; **26**(6): 1084-90.

21. Port GR, Wilkes TJ. Aedes (Diceromyia) furcifer/taylori and a yellow fever outbreak in The Gambia. *Trans R Soc Trop Med Hyg* 1979; **73**(3): 341-4.

22. Germain M, Francy DB, Monath TP, et al. Yellow fever in the Gambia, 1978--1979: entomological aspects and epidemiological correlations. *Am J Trop Med Hyg* 1980; **29**(5): 929-40.
23. Colebunders R, Mariage JL, Coche JC, et al. A Belgian traveler who acquired yellow fever in the Gambia. *Clin Infect Dis* 2002; **35**(10): e113-6.
24. Marchi S, Viviani S, Montomoli E, et al. Zika Virus in West Africa: A Seroepidemiological Study between 2007 and 2012. *Viruses* 2020; **12**(6): 641.
25. Huhtamo E, Uzcátegui NY, Siikamäki H, et al. Molecular epidemiology of dengue virus strains from Finnish travelers. *Emerg Infect Dis* 2008; **14**(1): 80-3.
26. Amoako N, Duodu S, Dennis FE, et al. Detection of Dengue Virus among Children with Suspected Malaria, Accra, Ghana. *Emerging Infectious Diseases* 2018; **24**(8): 1544-7.
27. Bonney JHK, Hayashi T, Dadzie S, et al. Molecular detection of dengue virus in patients suspected of Ebola virus disease in Ghana. *PLoS One* 2018; **13**(12): e0208907.
28. Fabiyi A. Yellow Fever at Tema, Ghana, 1959: A Serological Survey by Complement Fixation. *Annals of Tropical Medicine & Parasitology* 2016; **55**(2): 235-41.
29. Wang W, Sarkodie F, Danso K, et al. Seroprevalence of west nile virus in ghana. *Viral Immunol* 2009; **22**(1): 17-22.
30. Agadzi VK, Boatin BA, Appawu MA, Mingle JAA, Addy PAK. Yellow fever in Ghana, 1977-80. *Bulletin of the World Health Organization* 1984; **62 4**: 577-83.
31. Sherman KE, Rouster SD, Kong LX, et al. Zika Virus Exposure in an HIV-Infected Cohort in Ghana. *J Acquir Immune Defic Syndr* 2018; **78**(5): e35-e8.
32. Ankrah GA, Bonney JHK, Agbosu EE, Pratt D, Adiku TK. Serological evidence of Zika virus infection in febrile patients at Greater Accra Regional Hospital, Accra Ghana. *BMC Res Notes* 2019; **12**(1): 326.
33. Jentes ES, Bausch DG, Diakite F, et al. Acute Arboviral Infections in Guinea, West Africa, 2006. *The American Journal of Tropical Medicine and Hygiene* 2010; **83**(2): 388-94.
34. Naidenova EV, Kartashov MY, Zakharov KS, et al. Study of the prevalence of antibodies to some arboviruses in the population of the Republic of Guinea. *Problems of Virology* 2021; **66**(5): 346-53.
35. Hwang J, Ryu H-S, Kim H, Lee S-A. The First Reported Case of West Nile Encephalitis in Korea. *Journal of Korean Medical Science* 2015; **30**(3).
36. Bayandin RB, Makenov MT, Boumbaly S, et al. The First Case of Zika Virus Disease in Guinea: Description, Virus Isolation, Sequencing, and Seroprevalence in Local Population. *Viruses* 2023; **15**(8): 1620.
37. Rosenstierne MW, Schaltz-Buchholzer F, Bruzadelli F, et al. Zika virus IgG in infants with microcephaly, Guinea-Bissau, 2016. *Emerging Infectious Diseases* 2018; **24**(5): 948.
38. Organization WH. Yellow fever outbreak in Liberia: update. *Weekly Epidemiological Record= Relevé épidémiologique hebdomadaire* 1995; **70**(50): 360-.
39. Safronetz D, Sacko M, Sogoba N, et al. Vectorborne Infections, Mali. *Emerging Infectious Diseases* 2016; **22**(2): 340-2.
40. KURZ X. The Yellow Fever Epidemic in Western Mali, September—November 1987: Why Did Epidemiological Surveillance Fail? *Disasters* 1990; **14**(1): 46-54.
41. Fourié T, El Bara A, Dubot-Pérès A, et al. Emergence of dengue virus serotype 2 in Mauritania and molecular characterization of its circulation in West Africa. *PLoS Neglected Tropical Diseases* 2021; **15**(10): e0009829.
42. Bob NS, Bâ H, Fall G, et al. Detection of the Northeastern African Rift Valley fever virus lineage

during the 2015 outbreak in Mauritania. Open forum infectious diseases; 2017: Oxford University Press US; 2017. p. ofx087.
43. Lagare A, Faye M, Fintan G, et al. First introduction of dengue virus type 3 in Niger, 2022. *IJID regions* 2023; **7**: 230-2.
44. Carey D, Causey O, Reddy S, Cooke A. Dengue viruses from febrile patients in Nigeria, 1964-68. *The Lancet* 1971; **297**(7690): 105-6.
45. Moore DL, Causey OR, Carey DE, et al. Arthropod-borne viral infections of man in Nigeria, 1964–1970. *Annals of Tropical Medicine & Parasitology* 2016; **69**(1): 49-64.
46. Kolawole OM, Seriki AA, Irekeola AA, Bello KE, Adeyemi OO. Dengue virus and malaria concurrent infection among febrile subjects within Ilorin metropolis, Nigeria. *Journal of medical virology* 2017; **89**(8): 1347-53.
47. Fagbami AH, Onoja AB. Dengue haemorrhagic fever: An emerging disease in Nigeria, West Africa. *Journal of Infection and Public Health* 2018; **11**(6): 757-62.
48. Macnamara F. Zika virus: a report on three cases of human infection during an epidemic of jaundice in Nigeria. *Transactions of the royal society of tropical medicine and hygiene* 1954; **48**(2): 139-45.
49. Draper C. Infection with the Chuku strain of Spondweni virus. *West African Medical Journal* 1965; **14**(1): 16-9.
50. Oderinde BS, Mora-Cárdenas E, Carletti T, Baba MM, Marcello A. Prevalence of locally undetected acute infections of Flaviviruses in North-Eastern Nigeria. *Virus research* 2020; **286**: 198060.
51. Fagbami A, Monath T, Fabiyi A. Dengue virus infections in Nigeria: a survey for antibodies in monkeys and humans. *Transactions of the Royal Society of Tropical Medicine and Hygiene* 1977; **71**(1): 60-5.
52. Tomori O, Monath TP, O'Connor EH, Lee VH, Cropp CB. Arbovirus infections among laboratory personnel in Ibadan, Nigeria. *The American Journal of Tropical Medicine and Hygiene* 1981; **30**(4): 855-61.
53. Fagbami A. Human arthropod-borne virus infections in Nigeria. Serological and virological investigations and Shaki, Oyo State. *Journal of hygiene, epidemiology, microbiology, and immunology* 1978; **22**(2): 184-9.
54. Tomori O, Fagbami A, Fabiyi A. Isolations of West Nile virus from man in Nigeria. *Transactions of the Royal Society of Tropical Medicine and Hygiene* 1978; **72**(1): 103-4.
55. Baba M, Saron M, Diop O, Mathiot C, Adeniji J, Olaleye O. West Nile virus in mosquitoes and febrile patients in a semi-arid zone, Nigeria. *J Am Sci* 2006; **2**(2): 28-34.
56. Nwachukwu WE, Yusuff H, Nwangwu U, et al. The response to re-emergence of yellow fever in Nigeria, 2017. *International Journal of Infectious Diseases* 2020; **92**: 189-96.
57. Fagbami A. Zika virus infections in Nigeria: virological and seroepidemiological investigations in Oyo State. *Epidemiology & Infection* 1979; **83**(2): 213-9.
58. Bird B, Faye O, Freire CCM, et al. Molecular Evolution of Zika Virus during Its Emergence in the 20th Century. *PLoS Neglected Tropical Diseases* 2014; **8**(1).
59. Traore-Lamizana M, Zeller H, Monlun E, et al. Dengue 2 outbreak in southeastern Senegal during 1990: virus isolations from mosquitoes (Diptera: Culicidae). *Journal of Medical Entomology* 1994; **31**(4): 623-7.
60. Faye O, Ba Y, Faye O, et al. Urban Epidemic of Dengue Virus Serotype 3 Infection, Senegal, 2009. *Emerging Infectious Diseases* 2014; **20**(3).

61. Robin Y, Cornet M, Heme G, Le Gonidec G. Isolement du virus de la dengue au Sénégal. Annales de l'Institut Pasteur/Virologie; 1980: Elsevier; 1980. p. 149-54.
62. Renaudet J, Jan C, Ridet J, Adam C, Robin Y. A serological survey of arboviruses in the human population of Senegal. *Bulletin de la Societe de Pathologie Exotique et de ses Filiales* 1978; **71**(2): 131-40.
63. Diagne MM, Faye M, Faye O, et al. Emergence of Wesselsbron virus among black rat and humans in Eastern Senegal in 2013. *One Health* 2017; **3**: 23-8.
64. Ndione MHD, Ndiaye EH, Faye M, et al. Re-Introduction of West Nile Virus Lineage 1 in Senegal from Europe and Subsequent Circulation in Human and Mosquito Populations between 2012 and 2021. *Viruses* 2022; **14**(12): 2720.
65. Sow A, Ba Y, Diallo D, et al. Re-emergence of yellow fever in Kedougou, Southeastern Senegal in 2010-2011. *International Journal of Infectious Diseases* 2014; **21**: 219.
66. Thonnon J, Fontenille D, Tall A, et al. Re-emergence of yellow fever in Senegal in 1995. *The American journal of tropical medicine and hygiene* 1998; **59**(1): 108-14.
67. Schoepp RJ, Rossi CA, Khan SH, Goba A, Fair JN. Undiagnosed Acute Viral Febrile Illnesses, Sierra Leone. *Emerging Infectious Diseases* 2014; **20**(7): 1176-82.
68. de Araújo Lobo JM, Mores CN, Bausch DG, Christofferson RC. Serological evidence of under-reported dengue circulation in Sierra Leone. *PLoS neglected tropical diseases* 2016; **10**(4): e0004613.
69. Amarasinghe A. Dengue Virus Infection in Africa. *Emerging Infectious Diseases* 2011.
70. Zida-Compaore WIC, Gbeasor-Komlanvi FA, Tchankoni MK, et al. Estimation of yellow fever incidence in Togo between 2010 and 2020. *Travel Medicine and Infectious Disease* 2022; **50**: 102470.
71. Eisenhut M, Schwarz T, Hegenscheid B. Seroprevalence of dengue, chikungunya and Sindbis virus infections in German aid workers. *Infection* 1999; **27**(2): 82-5.
72. Manu SK, Bonney JHK, Pratt D, et al. Arbovirus circulation among febrile patients at the greater Accra Regional Hospital, Ghana. *BMC Research Notes* 2019; **12**: 1-5.
73. Haneche F, Leparc-Goffart I, Simon F, et al. Rift Valley fever in kidney transplant recipient returning from Mali with viral RNA detected in semen up to four months from symptom onset, France, autumn 2015. *Eurosurveillance* 2016; **21**(18): 30222.
74. Nabeth P, Kane Y, Abdalahi MO, et al. Rift Valley fever outbreak, Mauritania, 1998: seroepidemiologic, virologic, entomologic, and zoologic investigations. *Emerging infectious diseases* 2001; **7**(6): 1052.
75. Faye O, Diallo M, Diop D, et al. Rift Valley fever outbreak with East-Central African virus lineage in Mauritania, 2003. *Emerging infectious diseases* 2007; **13**(7): 1016.
76. Faye O, Ba H, Ba Y, et al. Reemergence of rift valley Fever, mauritania, 2010. *Emerging infectious diseases* 2014; **20**(2): 300.
77. Abdoullah B, Durand GA, Basco LK, et al. Seroprevalence of Alphaviruses (Togaviridae) among Urban Population in Nouakchott, Mauritania, West Africa. *Viruses* 2023; **15**(7): 1588.
78. Lagare A, Fall G, Ibrahim A, et al. First occurrence of Rift Valley fever outbreak in Niger, 2016. *Veterinary medicine and science* 2019; **5**(1): 70-8.
79. Bearcroft W, Porterfield J, Sutton R. The isolation and identification of Ukauwa, a Bunyamwera group virus, from Nigeria. *Transactions of the Royal Society of Tropical Medicine and Hygiene* 1963; **57**(4): 308-12.
80. Fagbami A, Tomori O. Tataguine virus isolations from humans in Nigeria, 1971–1975. *Transactions of the Royal Society of Tropical Medicine and Hygiene* 1981; **75**(6): 788.

81. Olaleye O, Tomori O, Ladipo M, Schmitz H. Rift Valley fever in Nigeria: infections in humans. *Revue scientifique et technique (International Office of Epizootics)* 1996; **15**(3): 923-35.
82. Opayele A, Odaibo G, Olaleye O. Rift valley fever virus infection among livestock handlers in Ibadan, Nigeria. *Journal of Immunoassay and Immunochemistry* 2018; **39**(6): 609-21.
83. Tomori O. Rift Valley fever virus infection in man in Nigeria. *Journal of Medical Virology* 1980; **5**(4): 343-50.
84. Oragwa AO, Oragwa FC, Oluwayelu DO. Serologic evidence of silent Rift Valley fever virus infection among occupationally exposed persons in northern Nigeria. *The Journal of Infection in Developing Countries* 2022; **16**(05): 881-7.
85. Moore DL, Reddy S, Akinkugbe FM, et al. An epidemic of Chikungunya fever at Ibadan, Nigeria, 1969. *Annals of Tropical Medicine & Parasitology* 2016; **68**(1): 59-68.
86. Adesina O, Odelola H. Ecological distribution of Chikungunya haemagglutination inhibition antibodies in human and domestic animals in Nigeria. *Tropical and geographical medicine* 1991; **43**(3): 271-5.
87. Zeller H, Diallo M, Angel G, et al. Ngari virus (Bunyaviridae: Bunyavirus). First isolation from humans in Senegal, new mosquito vectors, its epidemiology. *Bulletin de la Societe de Pathologie Exotique (1990)* 1996; **89**(1): 12-6.
88. Sow A, Faye O, Ba Y, et al. Widespread Rift Valley fever emergence in Senegal in 2013–2014. Open Forum Infectious Diseases; 2016: Oxford University Press; 2016. p. ofw149.
89. Thonnon J, Spiegel A, Diallo M, Diallo A, Fontenille D. Epidémies à virus Chikungunya en 1996 et 1997 au Sénégal. *Bull Soc Pathol Exot* 1999; **92**(2): 79-82.
90. Sow A, Faye O, Diallo M, et al. Chikungunya outbreak in Kedougou, southeastern Senegal in 2009–2010. Open Forum Infectious Diseases; 2018: Oxford University Press US; 2018. p. ofx259.
91. Ansumana R, Jacobsen KH, Leski TA, et al. Reemergence of Chikungunya Virus in Bo, Sierra Leone. *Emerging Infectious Diseases* 2013; **19**(7): 1108-10.
92. Boko-Collins PM, Ogouyemi-Hounto A, Adjinacou-Badou EG, et al. Assessment of treatment impact on lymphatic filariasis in 13 districts of Benin: progress toward elimination in nine districts despite persistence of transmission in some areas. *Parasites & vectors* 2019; **12**(1): 1-8.
93. WHO.World malaria report 2022.Geneva: World Health Organization,2022.
94. Agonhossou R, Akoton R, Dossou YA, et al. Surveillance of Plasmodium malariae infection among inhabitants of rural areas in Ouidah–Kpomasse–Tori Bossito health district, Benin. *Parasitology research* 2022; **121**(1): 275-86.
95. Damien GB, Djènontin A, Rogier C, et al. Malaria infection and disease in an area with pyrethroid-resistant vectors in southern Benin. *Malaria journal* 2010; **9**(1): 1-11.
96. Poirier P, Doderer-Lang C, Atchade PS, et al. The hide and seek of Plasmodium vivax in West Africa: report from a large-scale study in Beninese asymptomatic subjects. *Malaria journal* 2016; **15**: 1-9.
97. Kyelem D, Sanou S, Boatin Ba, Medlock J, Coulibaly S, Molyneux D. Impact of long-term ivermectin (Mectizan®) on Wuchereria bancrofti and Mansonella perstans infections in Burkina Faso: strategic and policy implications. *Annals of Tropical Medicine & Parasitology* 2003; **97**(8): 827-38.
98. Gnémé A, Guelbéogo WM, Riehle MM, et al. Plasmodium species occurrence, temporal distribution and interaction in a child-aged population in rural Burkina Faso. *Malaria journal* 2013; **12**(1): 1-9.
99. Williams J, Njie F, Cairns M, et al. Non-falciparum malaria infections in pregnant women in West

Africa. *Malaria journal* 2016; **15**: 1-8.
100. Centers for Disease Control and Prevention. Malaria Surveillance — United States, 2015. https://www.cdc.gov/mmwr/volumes/67/ss/ss6707a1.htm (accessed Oct 10, 2023)
101. Ouattara AF, Bjerum CM, Aboulaye M, et al. Semiannual treatment of albendazole alone is efficacious for treatment of lymphatic filariasis: a randomized open-label trial in Cote d'Ivoire. *Clinical Infectious Diseases* 2022; **74**(12): 2200-8.
102. Ehounoud BCH, Koyo CSB, Bongue LD, et al. Assessment of the burden of malaria and bacteraemia by retrospective molecular diagnosis in febrile illnesses and first-line anti-infectives in Côte d'Ivoire. *Travel Medicine and Infectious Disease* 2021; **43**: 102105.
103. De Laval F, Simon F, Bogreau H, et al. Emergence of Plasmodium ovale malaria among the French Armed Forces in the Republic of Ivory Coast: 20 years of clinical and biological experience. Clinical Infectious Diseases 2014; 58(8): e122-e8.
104. Centers for Disease Control and Prevention. Malaria Surveillance — United States, 2014. https://www.cdc.gov/mmwr/volumes/66/ss/ss6612a1.htm (accessed Oct 10, 2023)
105. Deribe K, Bakajika DK, Zoure HM-G, Gyapong JO, Molyneux DH, Rebollo MP. African regional progress and status of the programme to eliminate lymphatic filariasis: 2000–2020. *International Health* 2021; **13**(Supplement_1): S22-S7.
106. Da Veiga Leal S, Ward D, Campino S, et al. Drug resistance profile and clonality of Plasmodium falciparum parasites in Cape Verde: the 2017 malaria outbreak. *Malaria journal* 2021; **20**: 1-11.
107. Rebollo MP, Sambou SM, Thomas B, et al. Elimination of lymphatic filariasis in the Gambia. *PLoS neglected tropical diseases* 2015; **9**(3): e0003642.
108. Greenwood BM, Bradley A, Greenwood A, et al. Mortality and morbidity from malaria among children in a rural area of The Gambia, West Africa. *Transactions of the Royal Society of Tropical Medicine and Hygiene* 1987; **81**(3): 478-86.
109. McGregor IA, Wilson M, Billewicz W. Malaria infection of the placenta in The Gambia, West Africa; its incidence and relationship to stillbirth, birthweight and placental weight. *Transactions of the Royal Society of Tropical Medicine and Hygiene* 1983; **77**(2): 232-44.
110. Welch S, McGregor I, Williams K. The Duffy blood group and malaria prevalence in Gambian West Africans. *Transactions of the Royal Society of Tropical Medicine and Hygiene* 1977; **71**(4): 295-6.
111. Biritwum N-K, de Souza DK, Marfo B, et al. Fifteen years of programme implementation for the elimination of Lymphatic Filariasis in Ghana: Impact of MDA on immunoparasitological indicators. *PLoS neglected tropical diseases* 2017; **11**(3): e0005280.
112. Dinko B, Oguike MC, Larbi JA, Bousema T, Sutherland CJ. Persistent detection of Plasmodium falciparum, P. malariae, P. ovale curtisi and P. ovale wallikeri after ACT treatment of asymptomatic Ghanaian school-children. *International Journal for Parasitology: Drugs and Drug Resistance* 2013; **3**: 45-50.
113. Beavogui AH, Delamou A, Camara BS, et al. Prevalence of malaria and factors associated with infection in children aged 6 months to 9 years in Guinea: results from a national cross-sectional study. *Parasite Epidemiology and Control* 2020; **11**: e00162.
114. Snounou G, Pinheiro L, Gonçalves A, et al. The importance of sensitive detection of malaria parasites in the human and insect hosts in epidemiological studies, as shown by the analysis of field samples from Guinea Bissau. *Transactions of the Royal Society of Tropical Medicine and Hygiene* 1993; **87**(6): 649-53.
115. Jackson LC. Malaria in Liberian children and mothers: biocultural perceptions of illness vs

clinical evidence of disease. *Social Science & Medicine* 1985; **20**(12): 1281-7.
116. Bray E. Plasmodium ovale in Liberia. *American Journal of Tropical Medicine and Hygiene* 1957; **6**(6): 961-70.
117. Dembele L, Aniweh Y, Diallo N, et al. Plasmodium malariae and Plasmodium falciparum comparative susceptibility to antimalarial drugs in Mali. *Journal of Antimicrobial Chemotherapy* 2021; **76**(8): 2079-87.
118. Bernabeu M, Gomez-Perez GP, Sissoko S, et al. Plasmodium vivax malaria in Mali: a study from three different regions. *Malaria journal* 2012; **11**: 1-6.
119. Travel Doctor Network. Lymphatic Filariasis in Mauritania. https://www.traveldoctor.network/country/mauritania/risk/lymphatic-filariasis/ (accessed Oct 10, 2023)
120. Jordan S, Jelinek T, Aida A, et al. Population structure of Plasmodium falciparum isolates during an epidemic in southern Mauritania. *Tropical Medicine & International Health* 2001; **6**(10): 761-6.
121. El Moustapha I, Ouldabdallahi Moukah M, Ould Ahmedou Salem MS, et al. Malaria prevalence in Mauritania: a systematic review and meta-analysis. *Malaria Journal* 2023; **22**(1): 146.
122. Mint Lekweiry K, Ould Mohamed Salem Boukhary A, Gaillard T, et al. Molecular surveillance of drug-resistant Plasmodium vivax using pvdhfr, pvdhps and pvmdr1 markers in Nouakchott, Mauritania. *Journal of antimicrobial chemotherapy* 2012; **67**(2): 367-74.
123. Ba H, Duffy CW, Ahouidi AD, et al. Widespread distribution of Plasmodium vivax malaria in Mauritania on the interface of the Maghreb and West Africa. *Malaria journal* 2016; **15**: 1-6.
124. Badia-Rius X, Adamou S, Taylor MJ, Kelly-Hope LA. Morbidity hotspot surveillance: A novel approach to detect lymphatic filariasis transmission in non-endemic areas of the Tillabéry region of Niger. *Parasite Epidemiology and Control* 2023; **21**: e00300.
125. Coldiron ME, Assao B, Guindo O, et al. Prevalence of malaria in an area receiving seasonal malaria chemoprevention in Niger. *Malaria Journal* 2021; **20**(1): 1-8.
126. Oboh MA, Badiane AS, Ntadom G, Ndiaye YD, Diongue K, Ndiaye D. Molecular identification of Plasmodium species responsible for malaria reveals Plasmodium vivax isolates in Duffy negative individuals from southwestern Nigeria. *Malaria journal* 2018; **17**(1): 1-12.
127. Ohanu M, Mbah A, Okonkwo P, Nwagbo F. Interference by malaria in the diagnosis of typhoid using Widal test alone. *West African journal of medicine* 2003; **22**(3): 250-2.
128. Faye O, Faye B, Ndiaye J, Tine R, Ndiaye D. Epidemiological study on lymphatic filariasis (Wuchereria bancrofti) in Sénégal. *Medecine Tropicale: Revue du Corps de Sante Colonial* 2009; **69**(1): 101-2.
129. Roucher C, Rogier C, Sokhna C, Tall A, Trape J-F. A 20-year longitudinal study of Plasmodium ovale and Plasmodium malariae prevalence and morbidity in a West African population. *PloS one* 2014; **9**(2): e87169.
130. Faye FB, Konaté L, Rogier C, Trape J-F. Plasmodium ovale in a highly malaria endemic area of Senegal. *Transactions of the Royal Society of Tropical Medicine and Hygiene* 1998; **92**(5): 522-5.
131. Niang M, Thiam LG, Sow A, et al. A molecular survey of acute febrile illnesses reveals Plasmodium vivax infections in Kedougou, southeastern Senegal. *Malaria journal* 2015; **14**(1): 1-8.
132. Koroma JB, Sesay S, Conteh A, et al. Progress on elimination of lymphatic filariasis in Sierra Leone. *Parasites & vectors* 2018; **11**(1): 1-10.
133. Leski TA, Taitt CR, Swaray AG, et al. Use of real-time multiplex PCR, malaria rapid diagnostic test and microscopy to investigate the prevalence of Plasmodium species among febrile hospital patients in Sierra Leone. *Malaria journal* 2020; **19**(1): 1-8.

134. Dorkenoo MA, de Souza DK, Apetogbo Y, et al. Molecular xenomonitoring for post-validation surveillance of lymphatic filariasis in Togo: no evidence for active transmission. *Parasites & vectors* 2018; **11**: 1-9.
135. Kassegne K, Fei S-W, Ananou K, et al. A Molecular Investigation of Malaria Infections From High-Transmission Areas of Southern Togo Reveals Different Species of Plasmodium Parasites. *Frontiers in Microbiology* 2021; **12**: 732923.

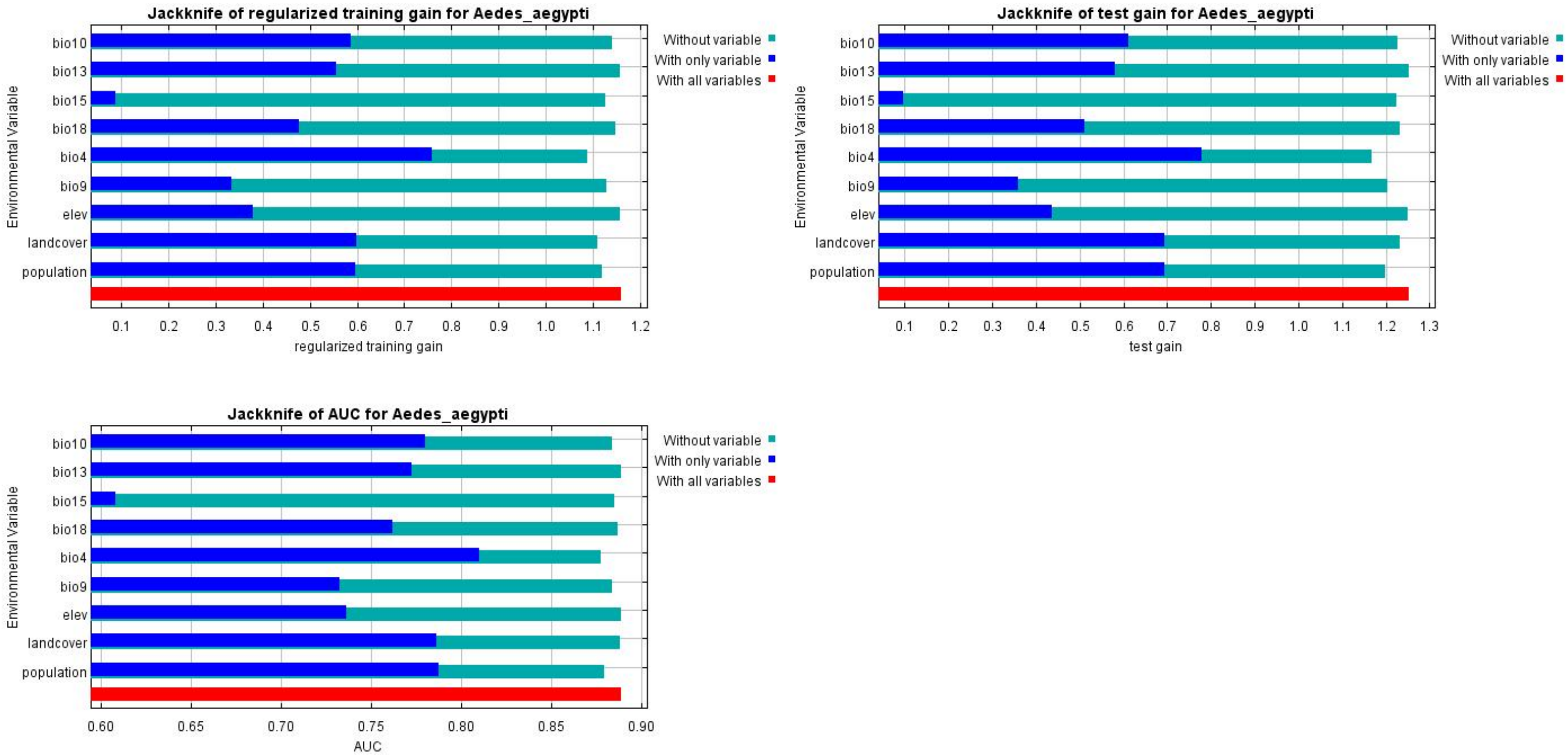

Supplementary Fig. 14. Jackknife plots of Maxent model for *Aedes aegypti* prediction. A. Training gain plot B. test gain plot C. AUC plot.

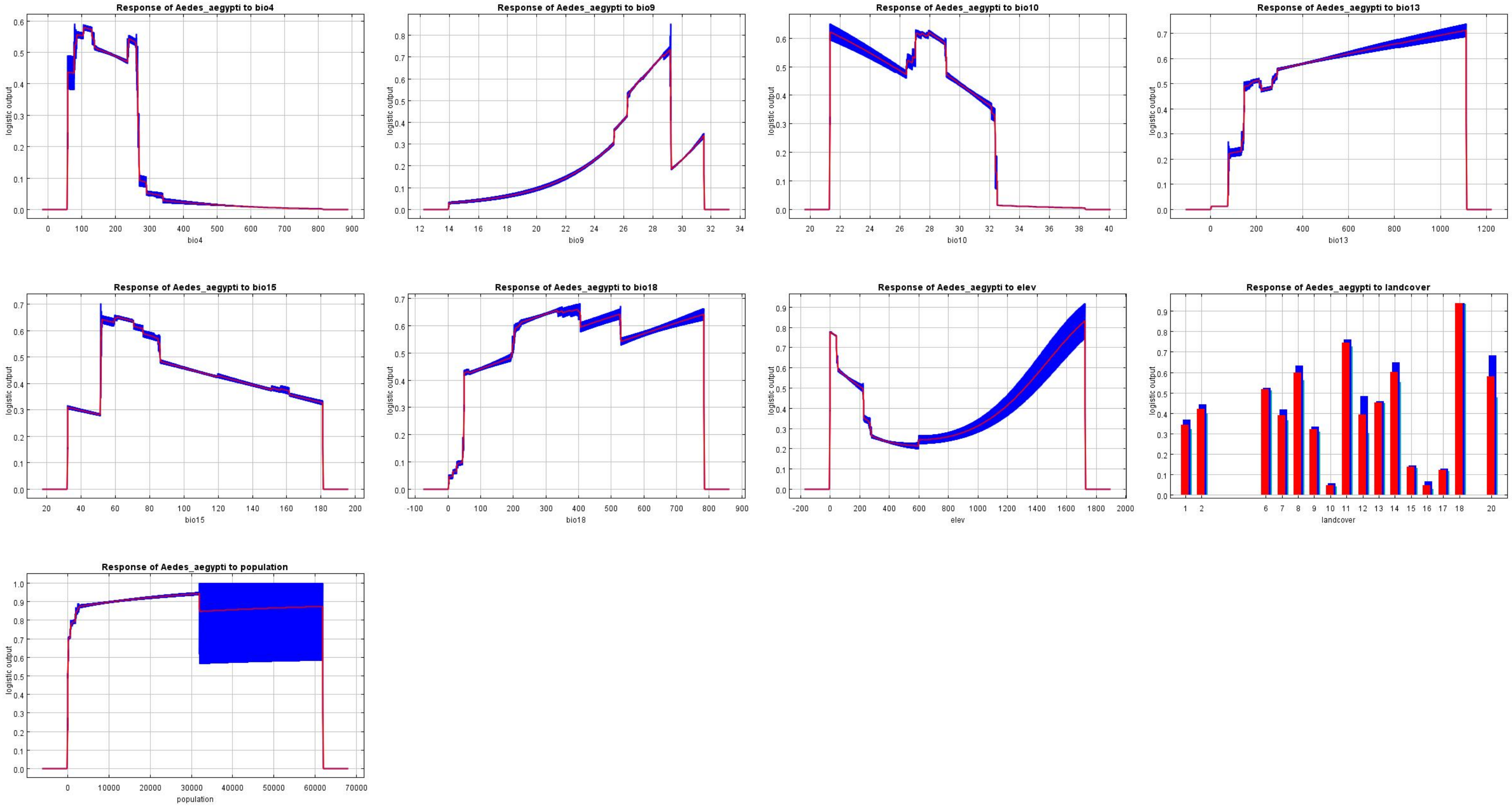

Supplementary Fig. 15. Response curves of environmental variables to probability of *Aedes aegypti* presence. The curves show the mean response of 10 replicate MaxEnt runs (red line) and standard deviation (blue shades).

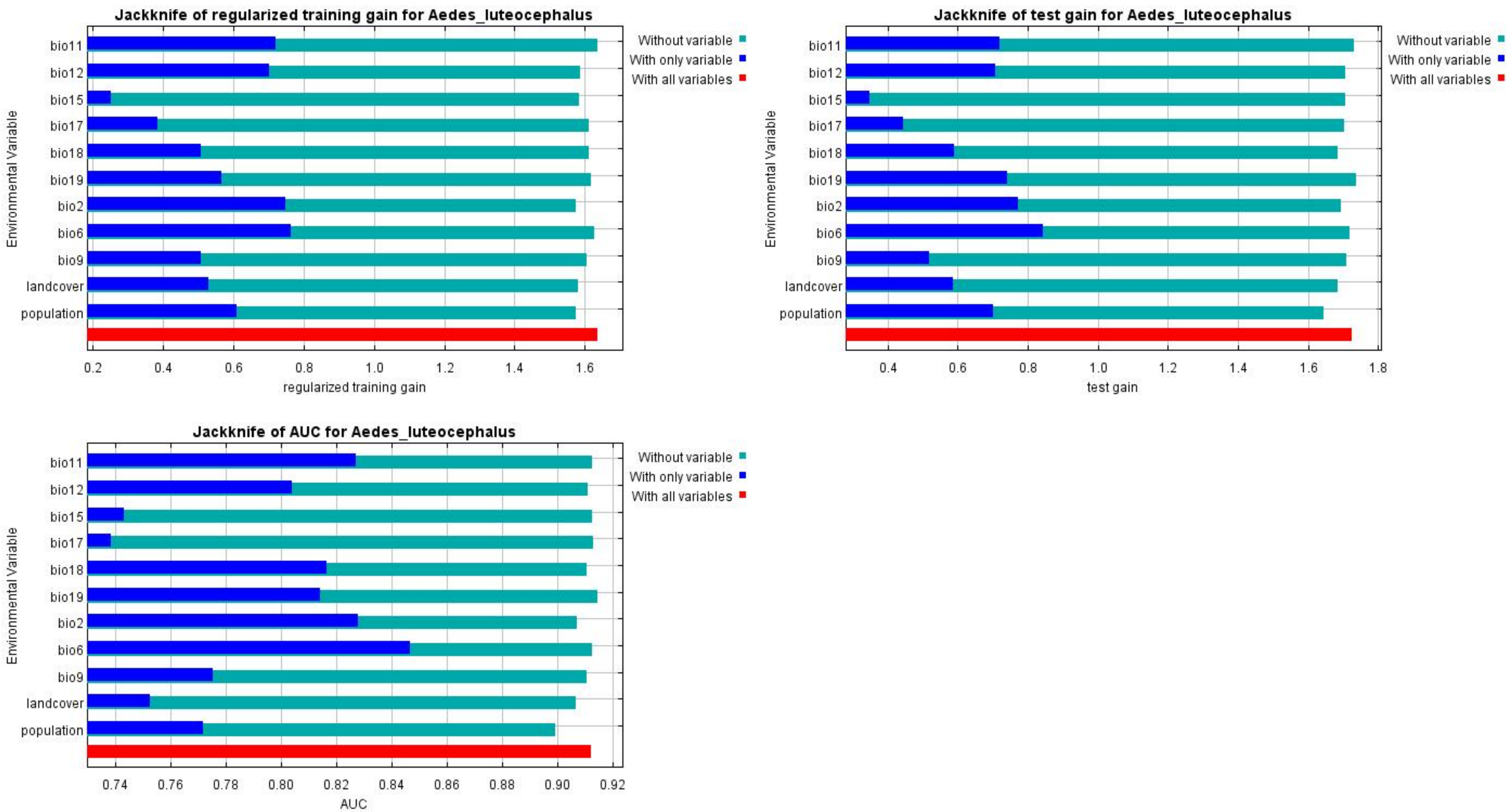

Supplementary Fig. 16. Jackknife plots of Maxent model for *Aedes luteocephalus* prediction. A. Training gain plot B. test gain plot C. AUC plot.

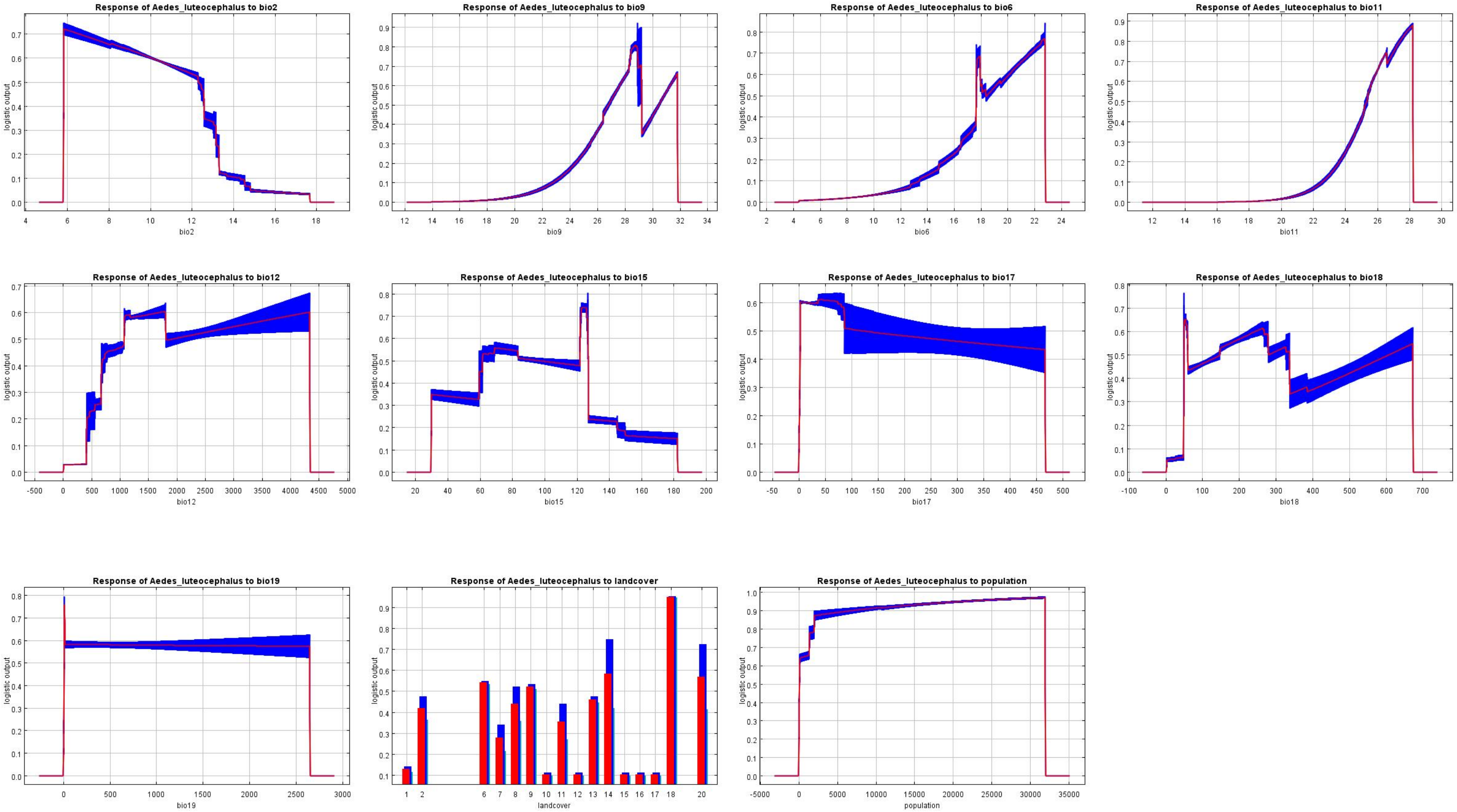

Supplementary Fig. 17. Response curves of environmental variables to probability of *Aedes luteocephalus* presence. The curves show the mean response of 10 replicate MaxEnt runs (red line) and standard deviation (blue shades).

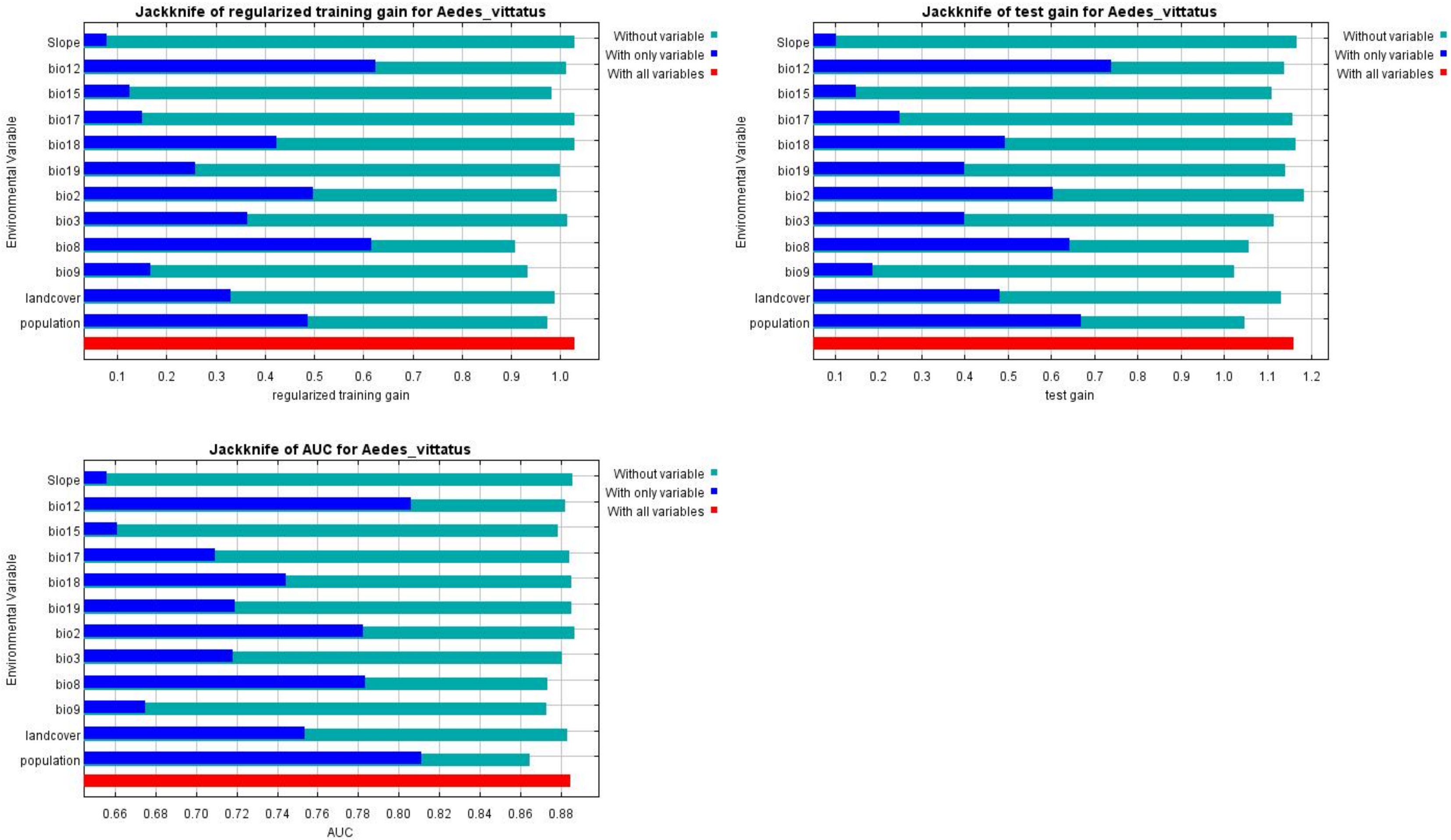

Supplementary Fig. 18. Jackknife plots of Maxent model for *Aedes vittatus* prediction. A. Training gain plot B. test gain plot C. AUC plot.

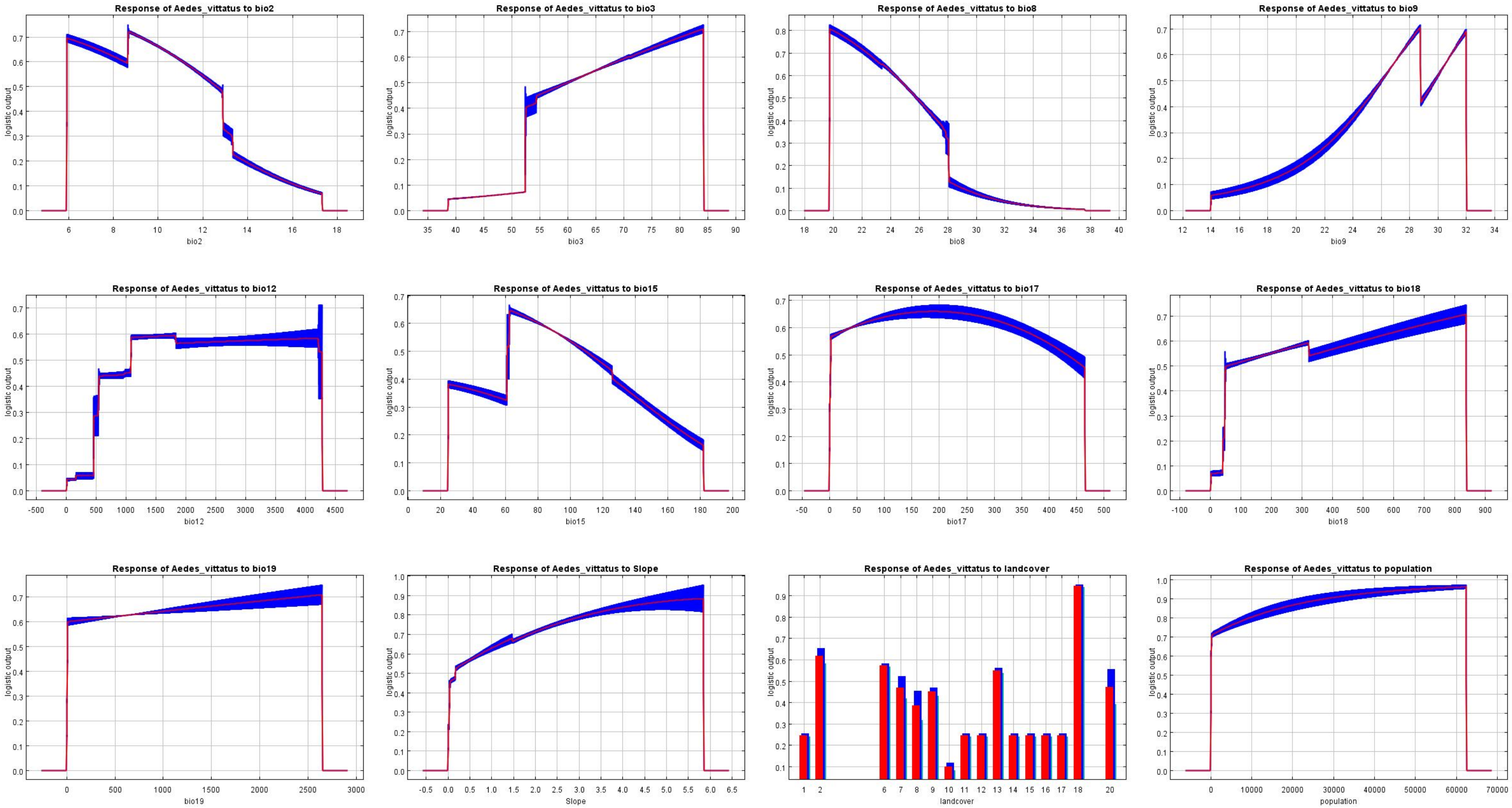

Supplementary Fig. 19. Response curves of environmental variables to probability of *Aedes vittatus* presence. The curves show the mean response of 10 replicate MaxEnt runs (red line) and standard deviation (blue shades).

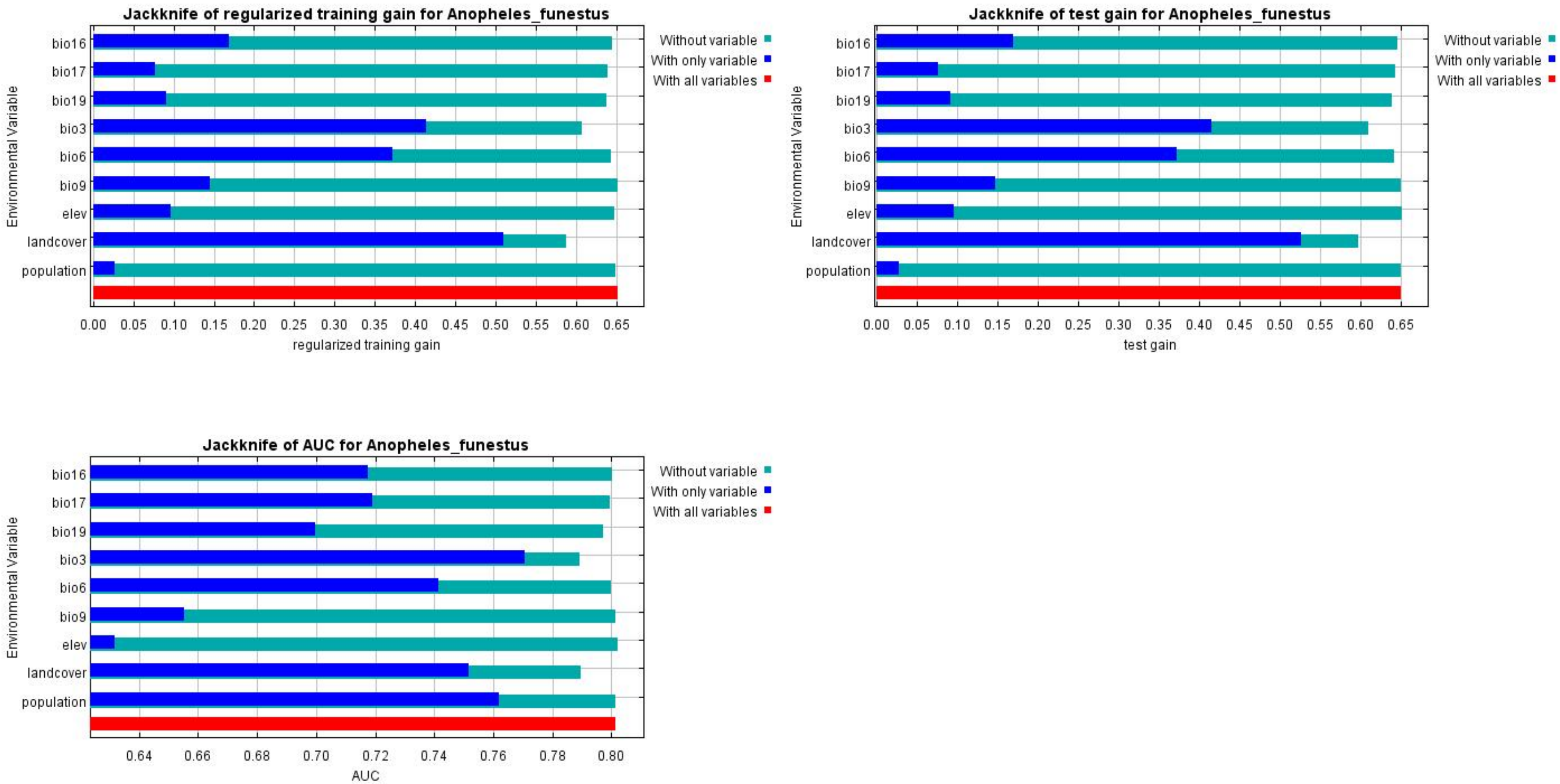

Supplementary Fig. 20. Jackknife plots of Maxent model for *Anopheles funestus* sensu lato prediction. A. Training gain plot B. test gain plot C. AUC plot.

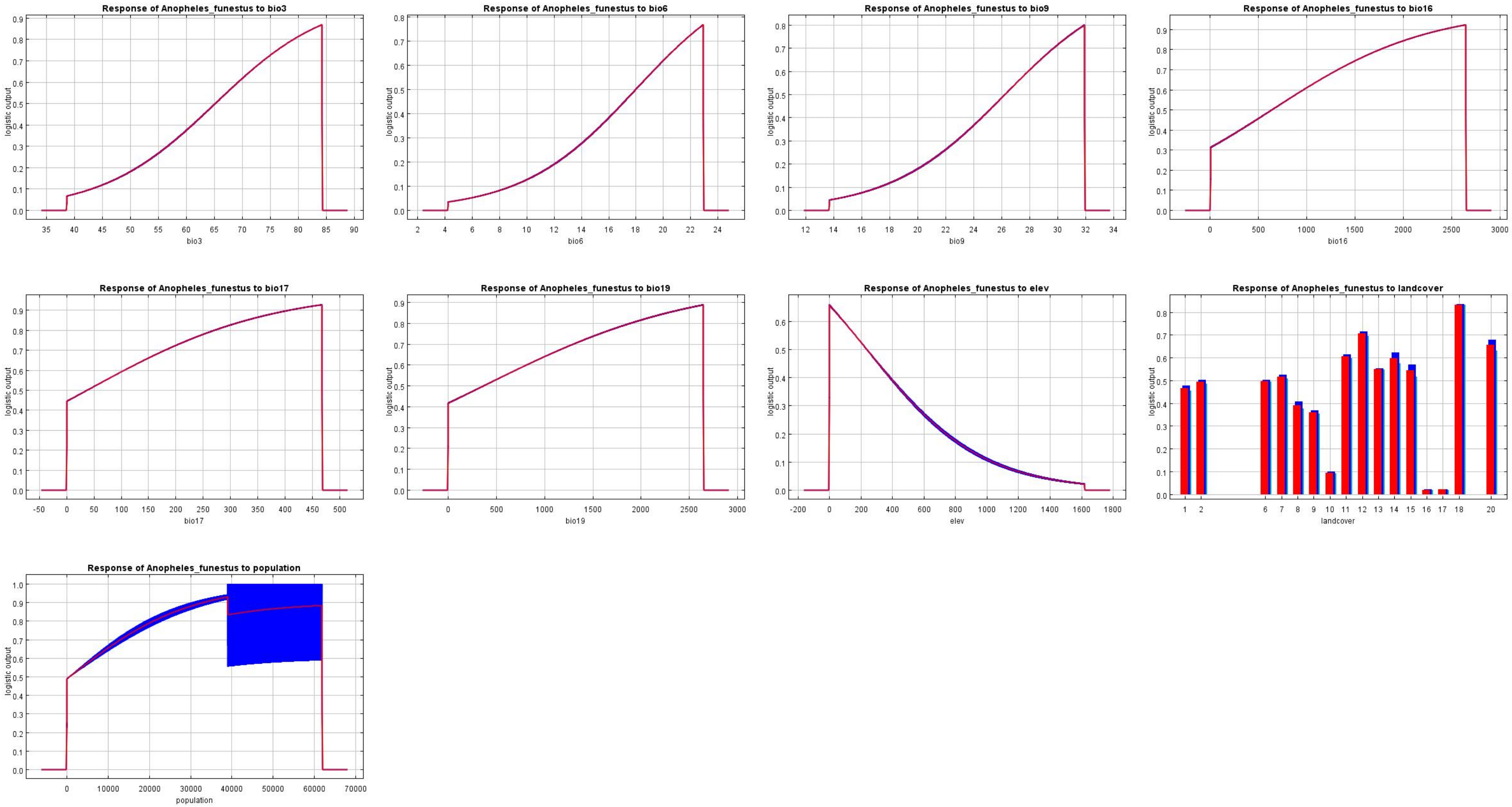

Supplementary Fig. 21. Response curves of environmental variables to probability of *Anopheles funestus* sensu lato presence. The curves show the mean response of 10 replicate MaxEnt runs (red line) and standard deviation (blue shades).

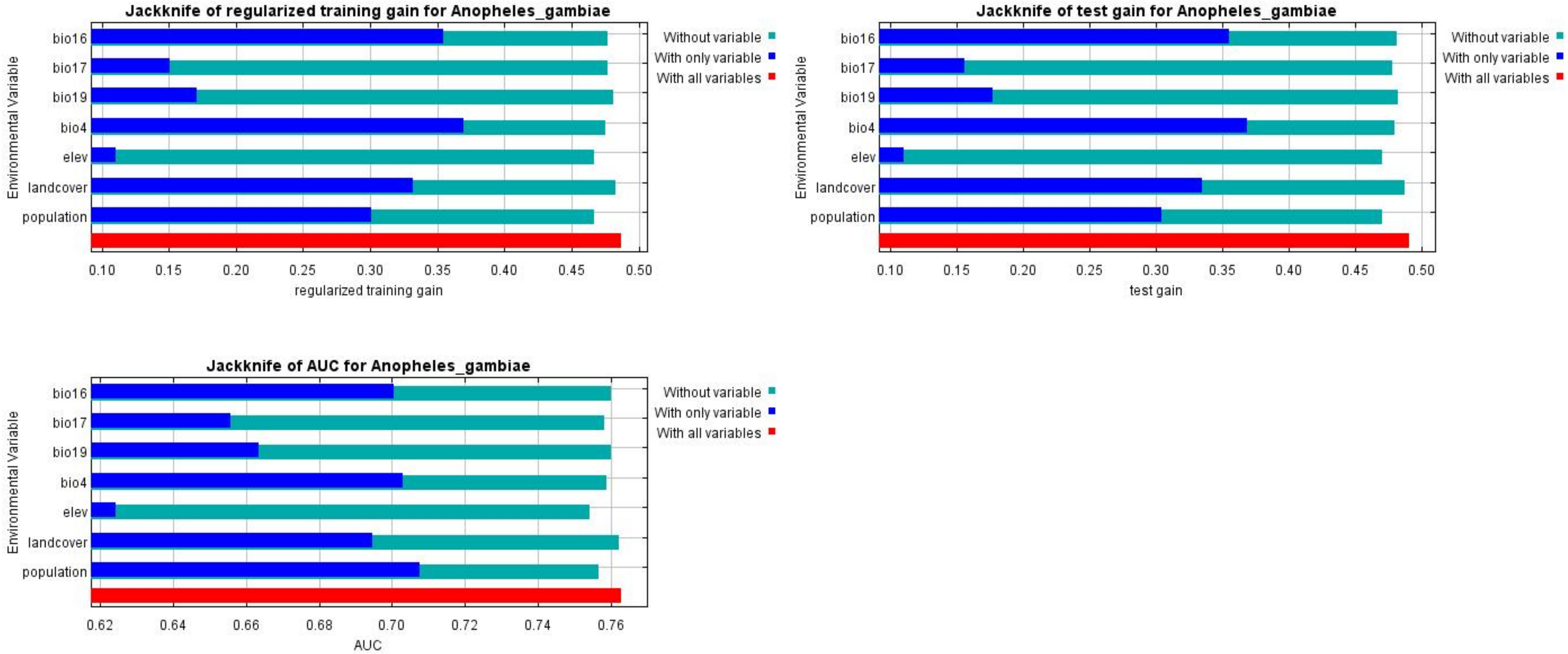

Supplementary Fig. 22. Jackknife plots of Maxent model for *Anopheles gambiae* sensu lato prediction. A. Training gain plot B. test gain plot C. AUC plot.

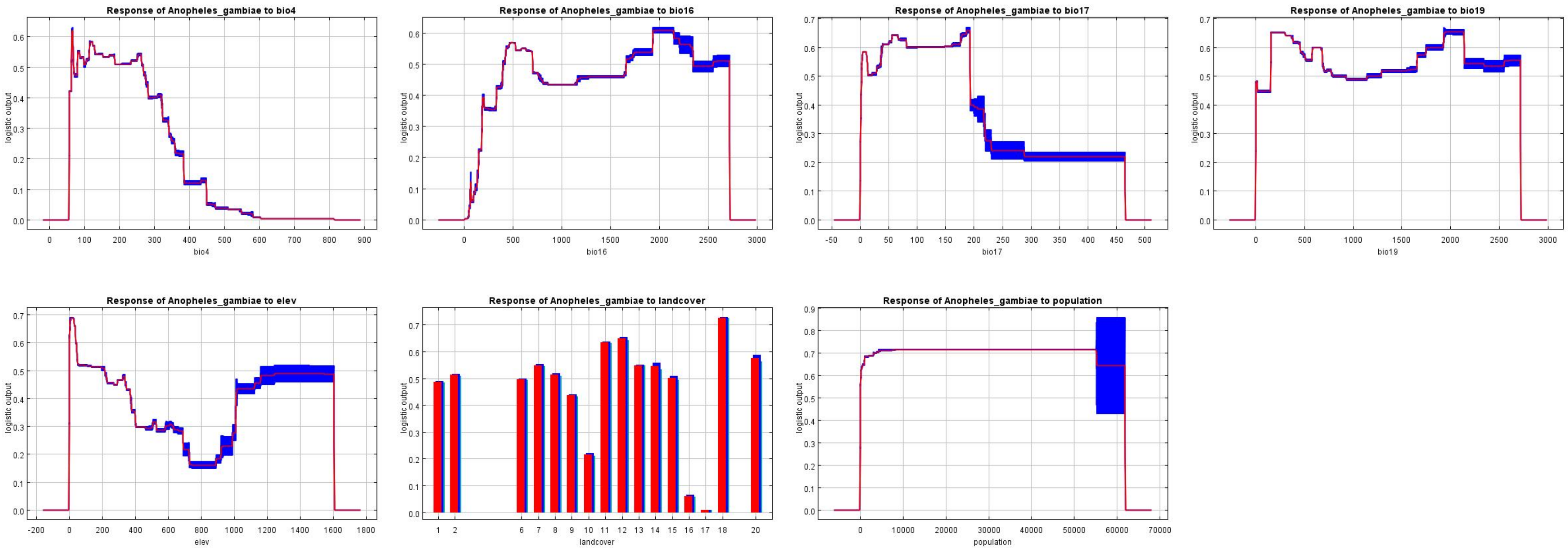

Supplementary Fig. 23. Response curves of environmental variables to probability of *Anopheles gambiae* sensu lato presence. The curves show the mean response of 10 replicate MaxEnt runs (red line) and standard deviation (blue shades).

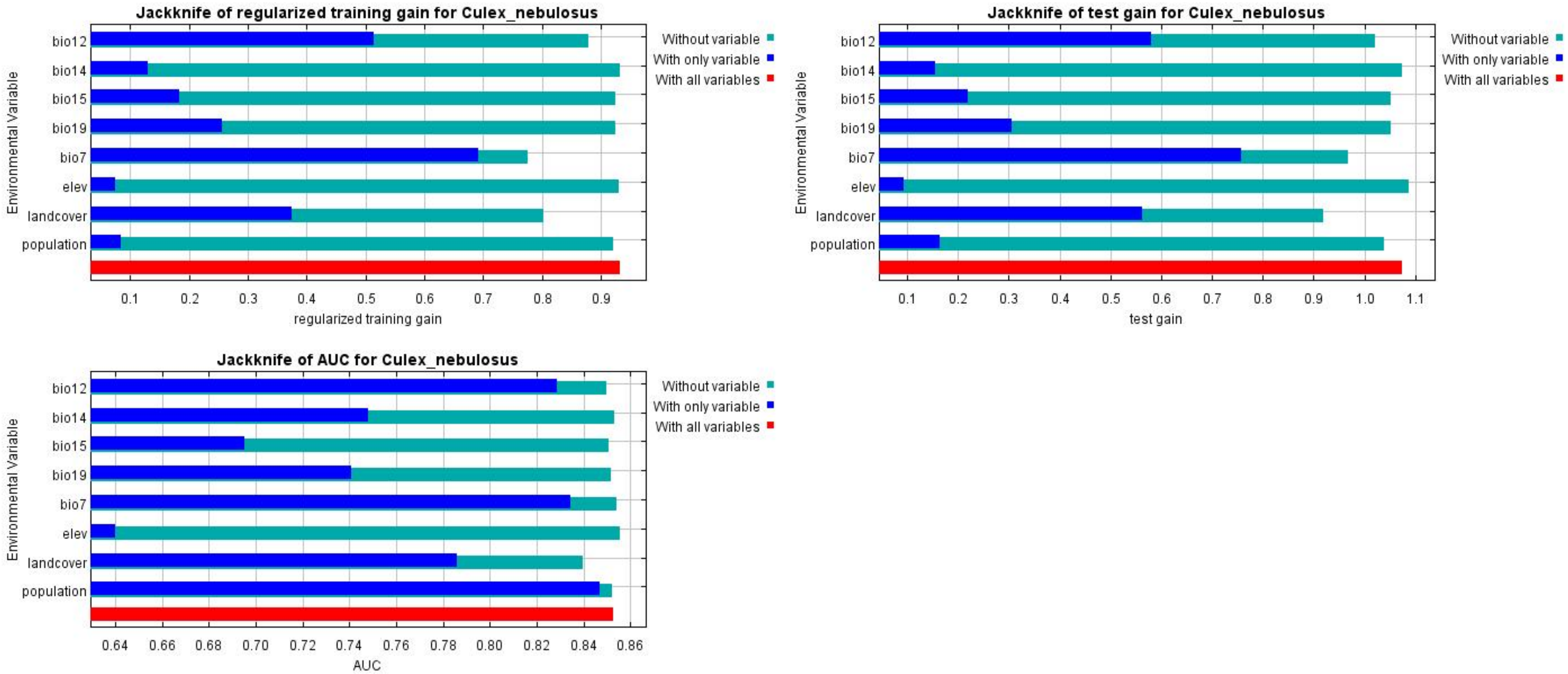

Supplementary Fig. 24. Jackknife plots of Maxent model for *Culex nebulosus* prediction. A. Training gain plot B. test gain plot C. AUC plot.

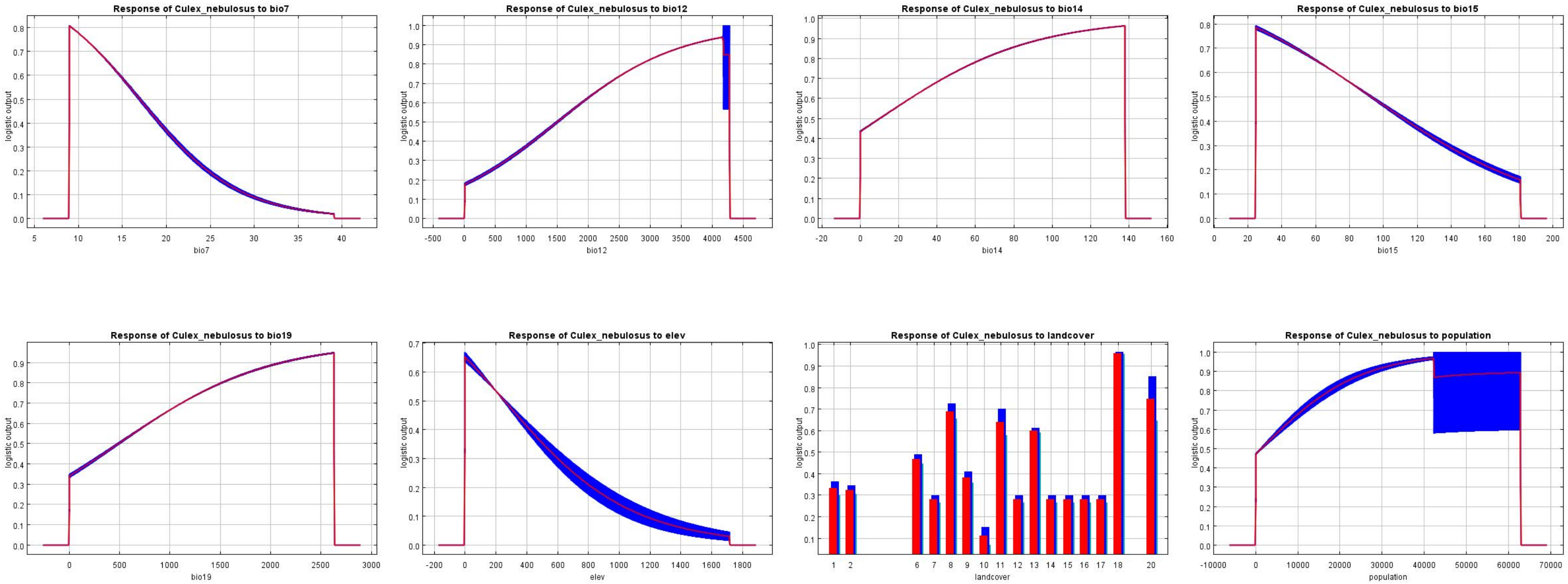

Supplementary Fig. 25. Response curves of environmental variables to probability of *Culex nebulosus* presence. The curves show the mean response of 10 replicate MaxEnt runs (red line) and standard deviation (blue shades).

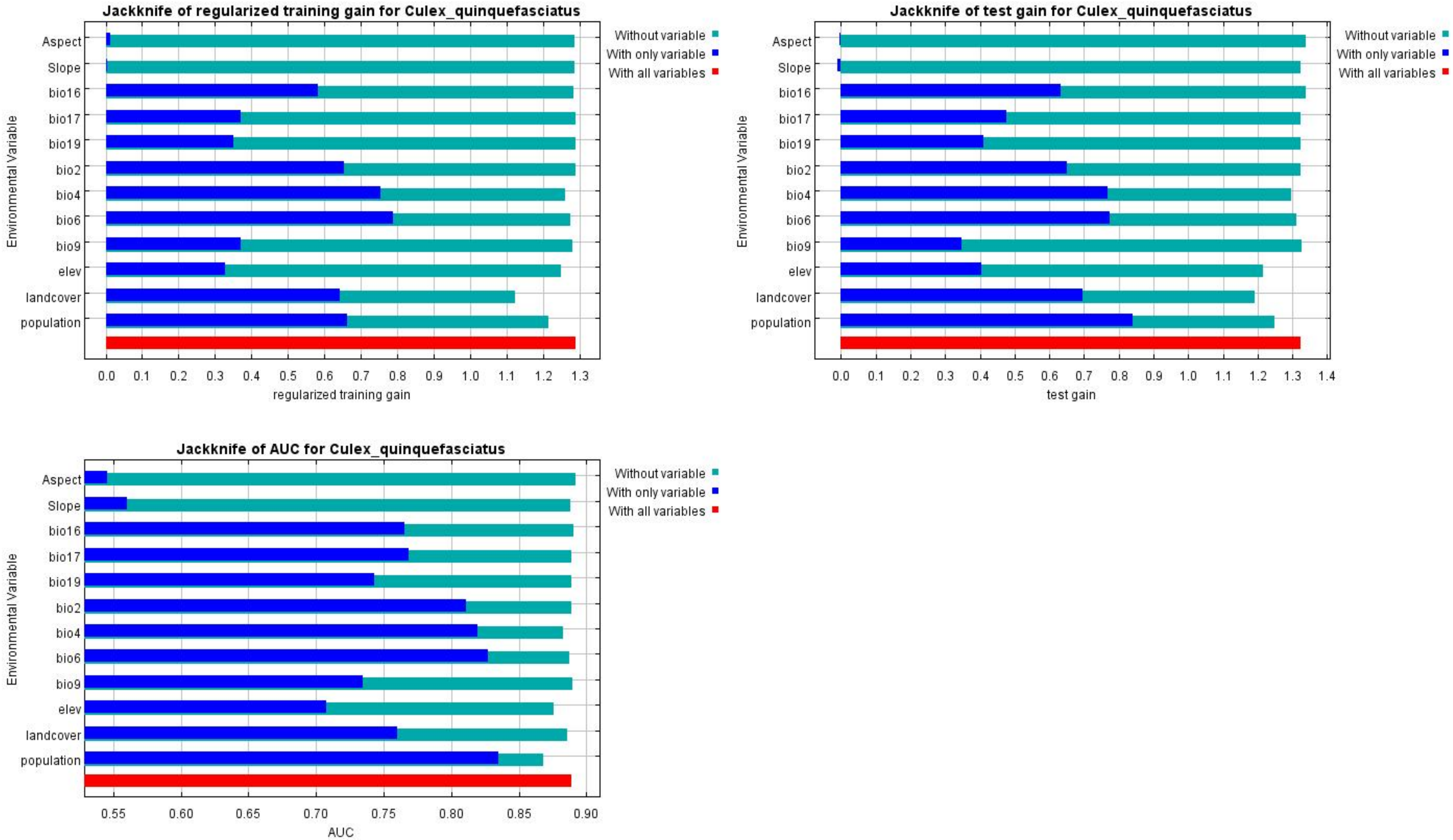

Supplementary Fig. 26. Jackknife plots of Maxent model for *Culex quinquefasciatus* prediction. A. Training gain plot B. test gain plot C. AUC plot.

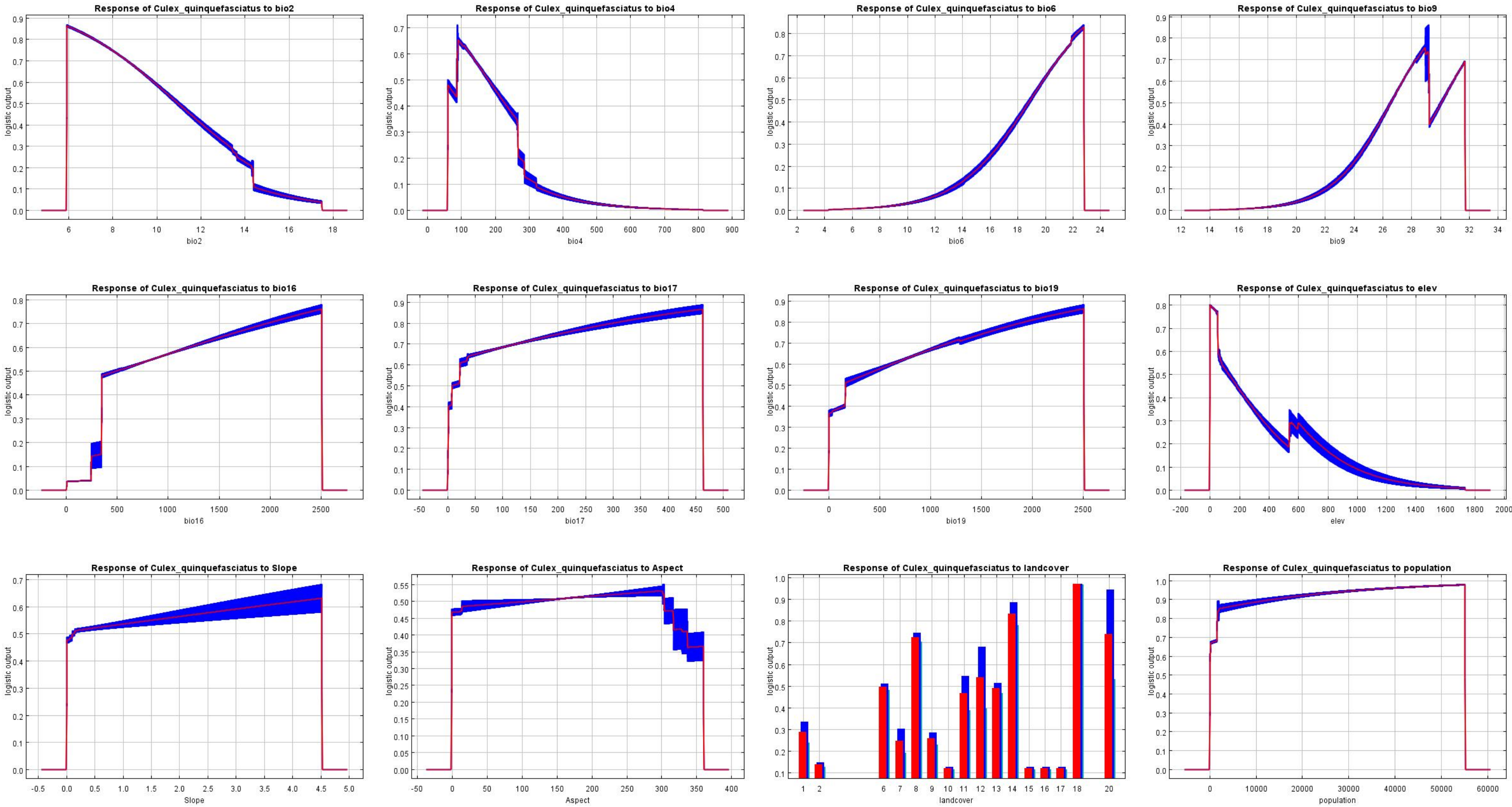

Supplementary Fig. 27. Response curves of environmental variables to probability of *Culex quinquefasciatus* presence. The curves show the mean response of 10 replicate MaxEnt runs (red line) and standard deviation (blue shades).

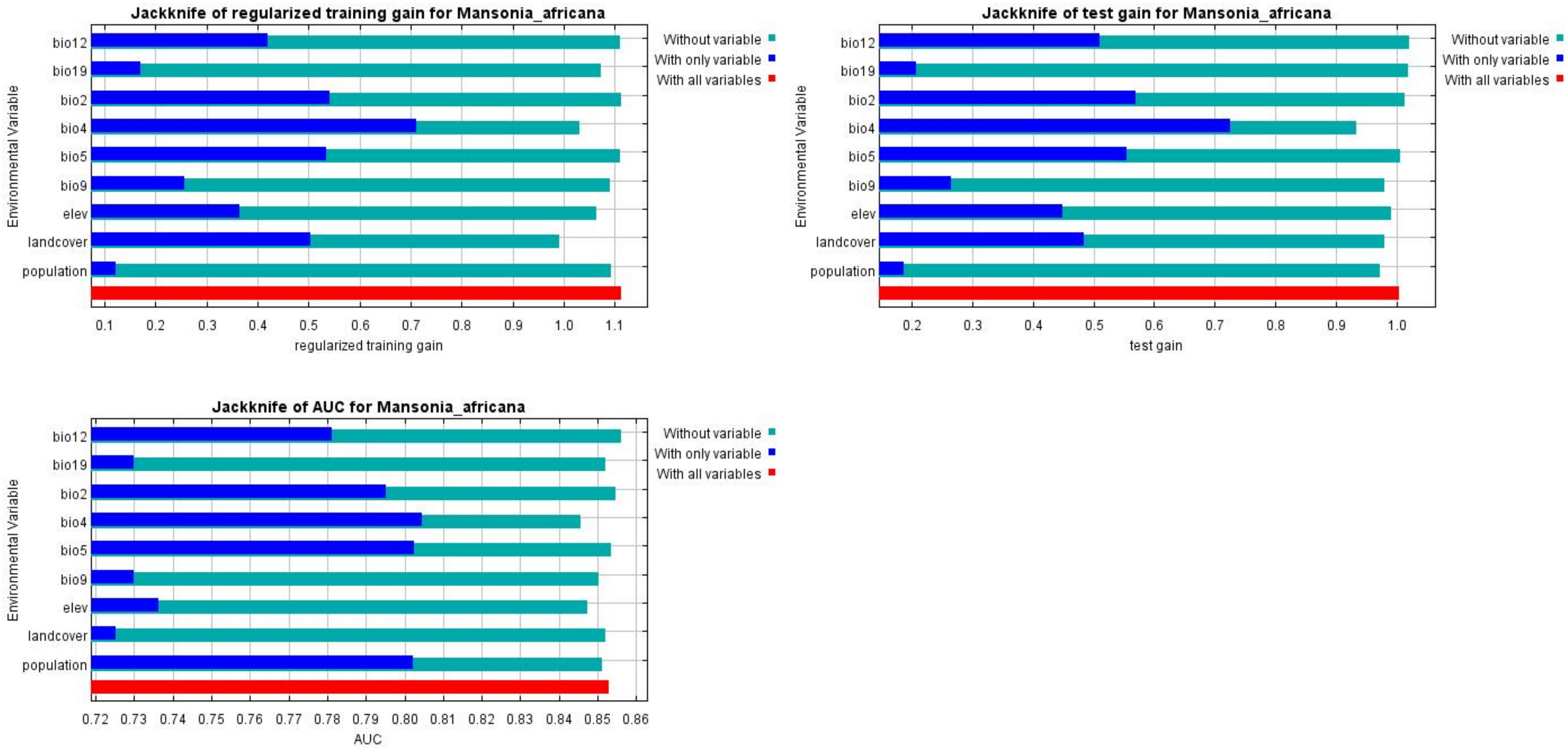

Supplementary Fig. 28. Jackknife plots of Maxent model for *Mansonia africana* prediction. A. Training gain plot B. test gain plot C. AUC plot.

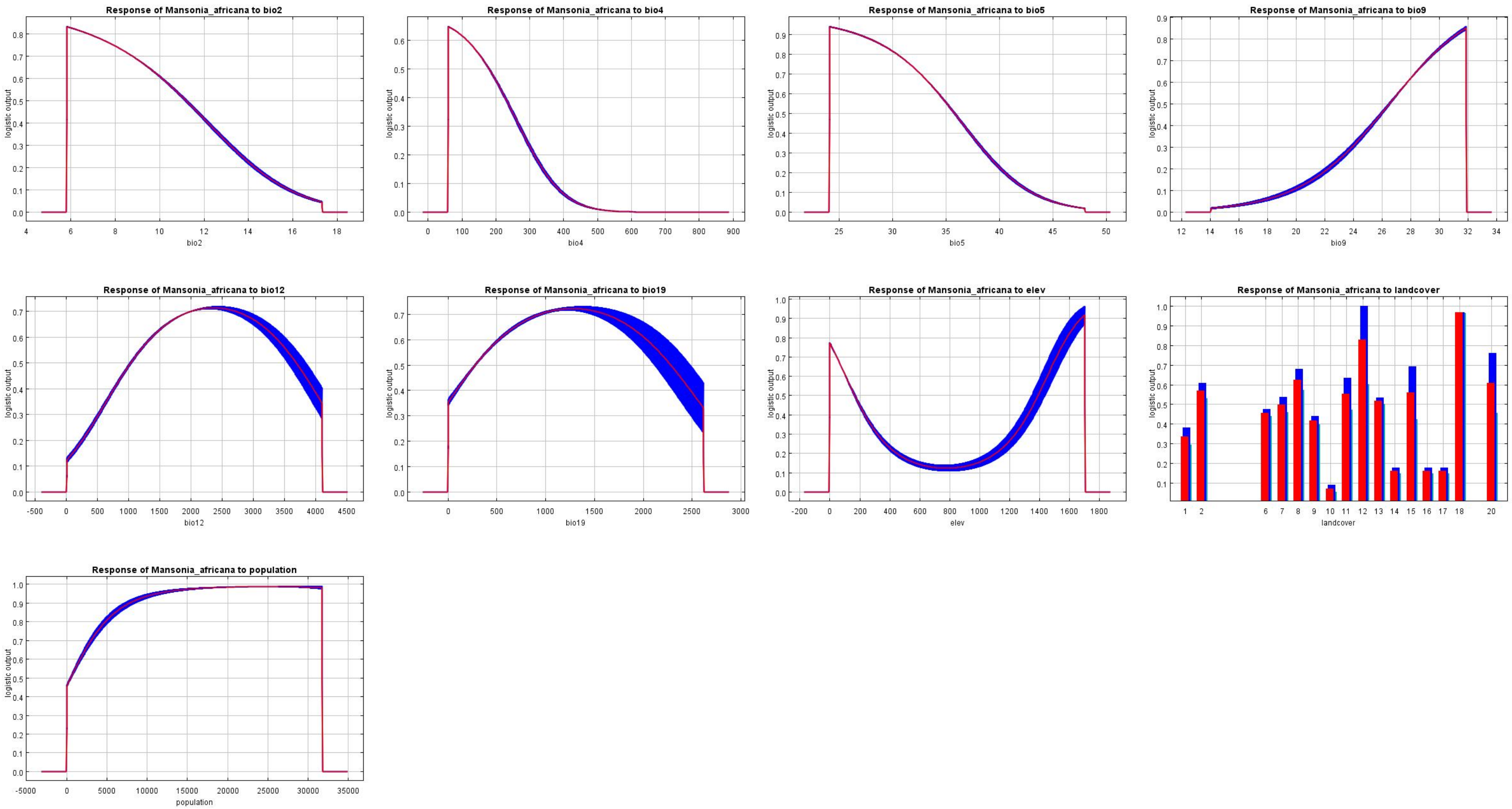

Supplementary Fig. 29. Response curves of environmental variables to probability of *Mansonia africana* presence. The curves show the mean response of 10 replicate MaxEnt runs (red line) and standard deviation (blue shades).

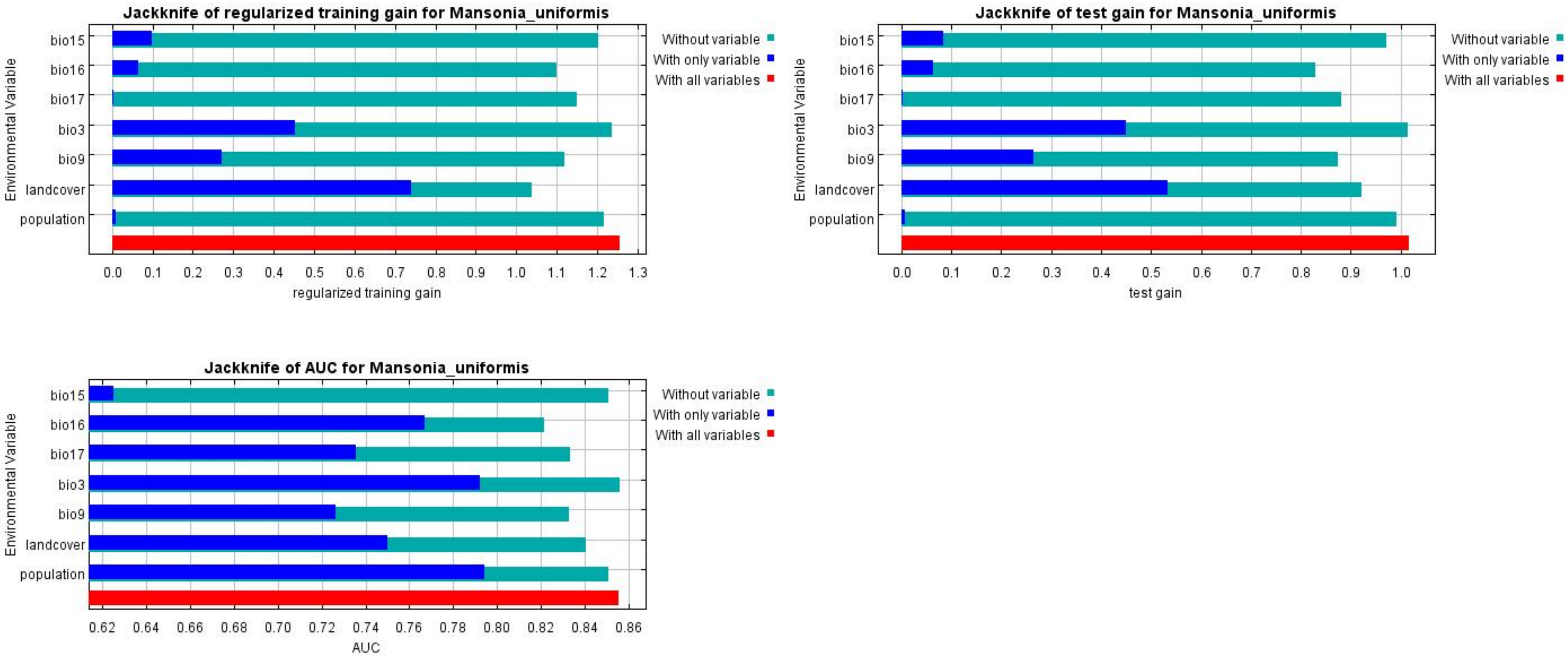

Supplementary Fig. 30. Jackknife plots of Maxent model for *Mansonia uniformis* prediction. A. Training gain plot B. test gain plot C. AUC plot.

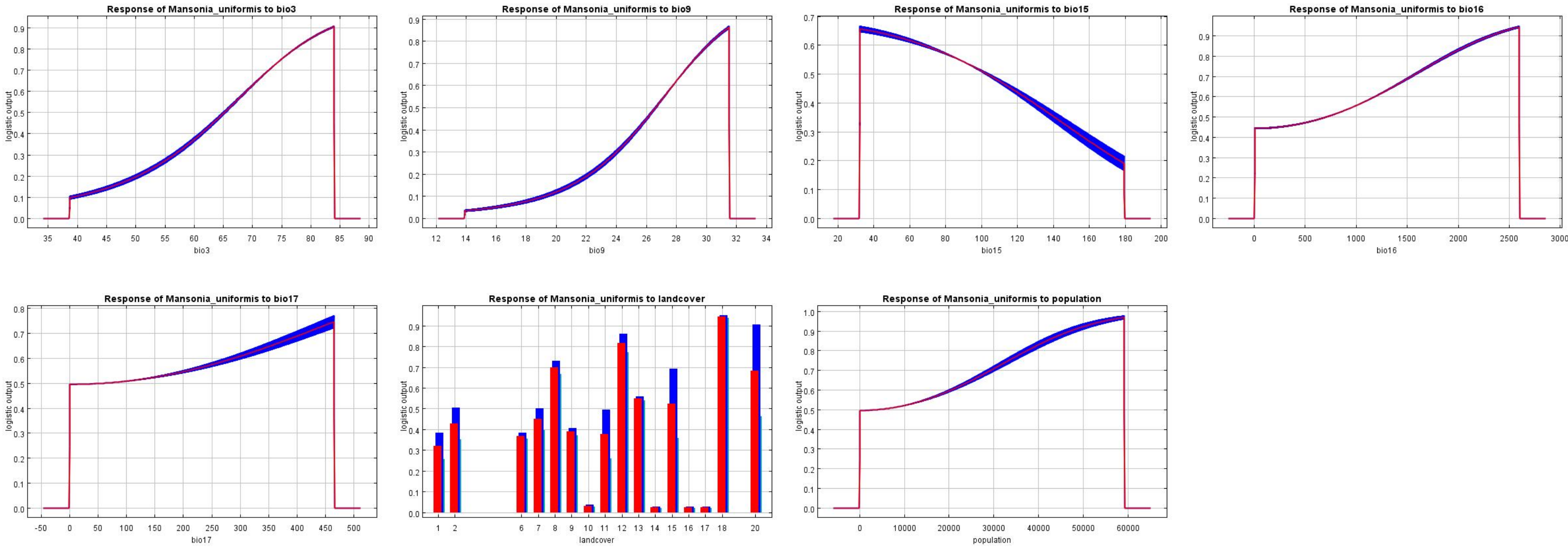

Supplementary Fig. 31. Response curves of environmental variables to probability of *Mansonia uniformis* presence. The curves show the mean response of 10 replicate MaxEnt runs (red line) and standard deviation (blue shades).

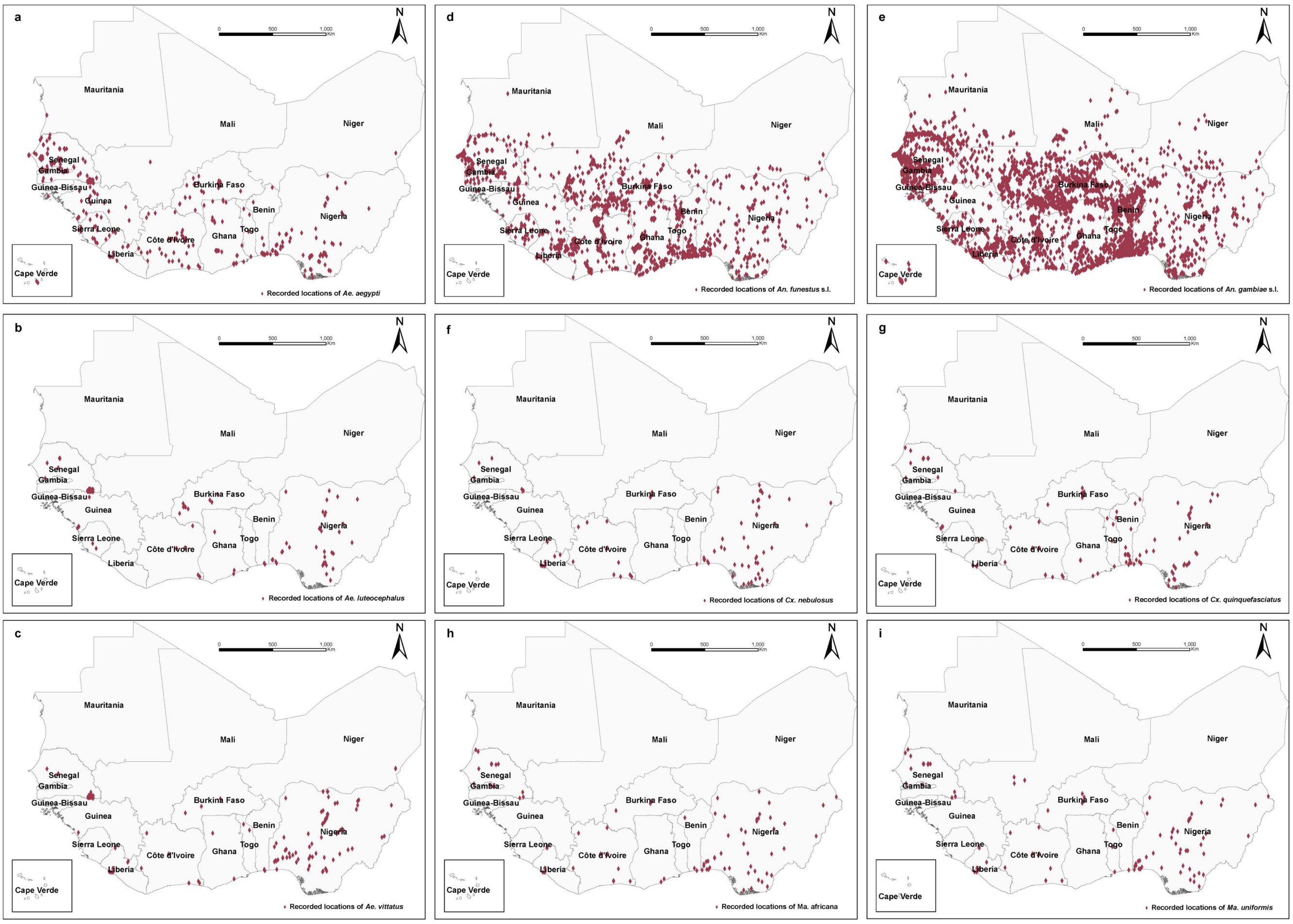

Supplementary Fig. 32. Geographical distribution of nine key mosquitoes in West Africa. **a**, *Aedes aegypti*. **b**, *Aedes luteocephalus*. **c**, *Aedes vittatus*. **d**, *Anopheles funestus* sensu lato. **e**, *Anopheles gambiae* sensu lato. **f**, *Culex nebulosus*. **g**, *Culex quinquefasciatus*. **h**, *Mansonia africana*. **i**, *Mansonia uniformis.* The red diamonds represent the recorded location of mosquito species with precise latitude and longitude.

Supplementary Table 2. Details of search strategy for literature review

Deadline	database	website	Retrieve Content	Search terms	Operator	Number of References
2023-3-6	Embase	https://www.embase.com/search/quick	mosquito:ti,ab,kw	Title, Abstract, Keywords	AND	2202
			sierra leone':ti,ab,kw OR togo:ti,ab,kw OR senegal:ti,ab,kw OR nigeria:ti,ab,kw OR niger:ti,ab,kw OR mauritania:ti,ab,kw OR mali:ti,ab,kw OR liberia:ti,ab,kw OR 'cote d`ivoire':ti,ab,kw OR 'guinea bissau':ti,ab,kw OR guinea:ti,ab,kw OR ghana:ti,ab,kw OR gambia:ti,ab,kw OR benin:ti,ab,kw OR 'burkina faso':ti,ab,kw OR 'cape verde':ti,ab,kw OR 'saint helena':ti,ab,kw OR 'west africa':ti,ab,kw OR 'tristan da cunha':ti,ab,kw OR ascension:ti,ab,kw	Title, Abstract, Keywords		
2023-3-6	Pubmed	https://pubmed.ncbi.nlm.nih.gov/	mosquitoes OR mosquito OR mosquitos OR Culicidae	Title, Abstract	AND	2564
			Togo OR Sierra Leone OR Senegal OR Dahomey OR Nigeria OR Niger OR Mauritania OR Mali OR Liberia OR Ivory Coast OR Côte d'Ivoire OR Guinea-Bissau OR Guinea OR Ghana OR Gambia OR Benin OR Burkina Faso OR Upper Volta OR Cape Verde OR Cabo Verde OR Saint Helena OR Ascension OR Tristan da Cunha OR west africa OR western africa	Title, Abstract		
2023-3-6	ISI Web of science	https://www.webofscience.com/wos/alldb/basic-search	mosquitoes OR mosquito OR mosquitos OR Culicidae	Abstract(AB)	AND	3966
			Togo OR Sierra Leone OR Senegal OR Dahomey OR Nigeria OR Niger OR Mauritania OR Mali OR Liberia OR Ivory Coast OR Côte d'Ivoire OR Guinea-Bissau OR Guinea OR Ghana OR Gambia OR Benin OR Burkina Faso OR Upper Volta OR Cape Verde OR Cabo Verde OR Saint Helena OR Ascension OR Tristan da Cunha OR west africa OR western africa	Abstract(AB)		
2023-3-6	Scoups	https://www.scopus.com/	mosquitoes OR mosquito OR mosquitos OR Culicidae	Title, Abstract, Keywords	AND	4508
			Togo OR Sierra Leone OR Senegal OR Dahomey OR Nigeria OR Niger OR Mauritania OR Mali OR Liberia OR Ivory Coast OR Côte d'Ivoire OR Guinea-Bissau OR Guinea OR Ghana OR Gambia OR Benin OR Burkina Faso OR Upper Volta OR Cape Verde OR Cabo Verde OR Saint Helena OR Ascension OR Tristan da Cunha OR west africa OR western africa	Title, Abstract, Keywords		

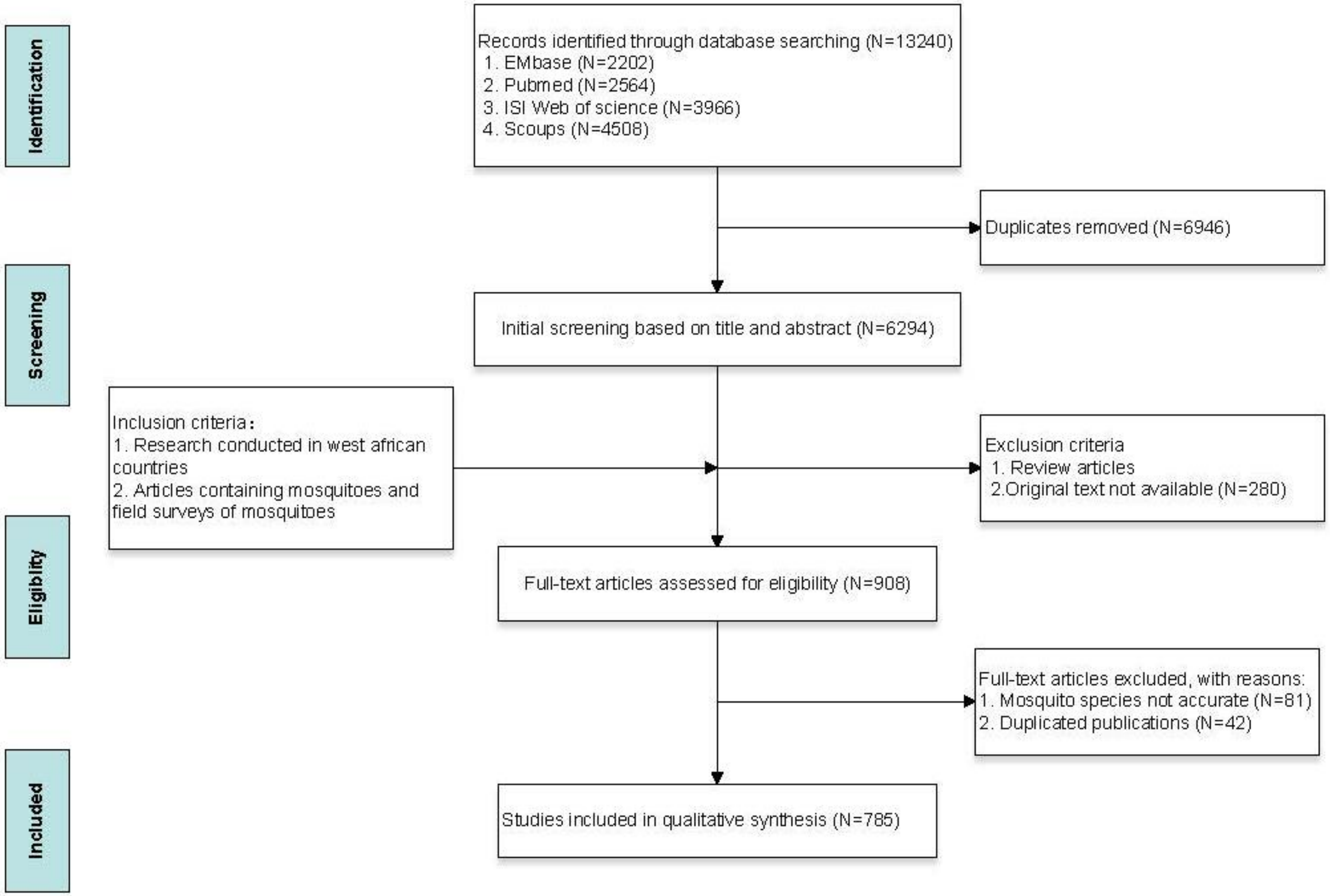

Supplementary Fig. 33. PRISMA flow diagram of study selection process.

Supplementary Text 3. References for mosquitoes and mosquito-associated pathogens in West Africa

A systematic review following PRISMA guidelines was performed. As shown in Supplementary Fig. 33, 13240 articles were identified from literature search, and 785 articles that met our inclusion and exclusion criteria were finally included for excerpts of related information, of which 160 reported the detection of mosquito-associated pathogens. Each article was carefully reviewed to collect the following information using a standard form: basic information of literature, study location, time and location of collection, mosquito species, and detection methods and results for mosquito-associated pathogens.

1. Ibrahim S, Mukhtar M, Muhammad A, Wondji C. 2La Paracentric Chromosomal Inversion and Overexpressed Metabolic Genes Enhance Thermotolerance and Pyrethroid Resistance in the Major Malaria Vector Anopheles gambiae. *Biology* 2021; **10**(6).
2. Davidson G, Hamon J. A Case of Dominant Dieldrin Resistance in *Anopheles gambiae* Giles. *Nature* 1962; **196**(4858): 1012-&.
3. Service MW, Sulaiman S, Esena R. A chemical aquatic light trap for mosquito larvae (Diptera: Culicidae). *J Med Entomol* 1983; **20**(6): 659-63.
4. Service MW, Highton RB. A chemical light trap for mosquitoes and other biting insects. *J Med Entomol* 1980; **17**(2): 183-5.
5. Riehle MM, Guelbeogo WM, Gneme A, et al. A cryptic subgroup of Anopheles gambiae is highly susceptible to human malaria parasites. *Science* 2011; **331**(6017): 596-8.
6. Service MW, Davidson G. A High Incidence of Dieldrin-resistance in Anopheles gambiae Giles from an Unsprayed Area in Northern Nigeria. *Nature* 1964; **203**: 209-10.
7. Lucas ER, Rockett KA, Lynd A, et al. A high throughput multi-locus insecticide resistance marker panel for tracking resistance emergence and spread in Anopheles gambiae. *Scientific Reports* 2019; **9**(1).
8. Foll CV, Pant CP, Lietaert PE. A large-scale field trial with dichlorvos as a residual fumigant insecticide in Northern Nigeria. *Bull World Health Organ* 1965; **32**(4): 531-&.
9. Bomblies A, Duchemin J-B, Eltahir EAB. A mechanistic approach for accurate simulation of village scale malaria transmission. *Malar J* 2009; **8**(1).
10. Service MW. A new Anopheles (Dipt., Culicidae) from Nigeria and a note on A. pretoriensis (Theobald). *Bull Entomol Res* 1970; **59**(4): 647-9.
11. Vontas J, Rowland M, Boko P, et al. A New Long-Lasting Indoor Residual Formulation of the Organophosphate Insecticide Pirimiphos Methyl for Prolonged Control of Pyrethroid-Resistant Mosquitoes: An Experimental Hut Trial in Benin. *PLoS ONE* 2013; **8**(7).
12. Service MW. A newAnopheles(Dipt., Culicidae) from Nigeria together with a note onAn. brohieriEdwards. *Annals of Tropical Medicine & Parasitology* 2016; **71**(2): 233-6.
13. Thomas TCE. A Note on the Occurrence ofCulex (Culex) Pipiens Fatigansin Sierra Leone. *Annals of Tropical Medicine & Parasitology* 2016; **50**(4): 421-5.
14. Müller GC, Junnila A, Traore MM, et al. A novel window entry/exit trap for the study of endophilic behavior of mosquitoes. *Acta Tropica* 2017; **167**: 137-41.
15. Talom AD, Essoung MA, Gbankoto A, et al. A preliminary analysis on the effect of copper on Anopheles coluzzii insecticide resistance in vegetable farms in Benin. *Scientific Reports* 2020; **10**(1).
16. Coleman S, Dadzie SK, Seyoum A, et al. A reduction in malaria transmission intensity in

Northern Ghana after 7 years of indoor residual spraying. *Malar J* 2017; **16**(1).
17. Ossè RA, Aïkpon R, Gbédjissi GL, et al. A shift from indoor residual spraying (IRS) with bendiocarb to long-lasting insecticidal (mosquito) nets (LLINs) associated with changes in malaria transmission indicators in pyrethroid resistance areas in Benin. *Parasit Vectors* 2013; **6**: 73.
18. Gordon RM, Hicks EP, Davey TH, Watson M. A Study of the House-Haunting Culicidae Occurring in Freetown, Sierra Leone; and of the Part Played by them in the Transmission of Certain Tropical Diseases, Together with Observations on the Relationship of Anophelines to Housing, and the Effects of Antilarval Measures in Freetown. *Annals of Tropical Medicine & Parasitology* 2016; **26**(3): 273-345.
19. Milligan PJM, Phillips A, Broomfield G, Molyneux DH, Touré Y, Coluzzi M. A study of the use of gas chromatography of cuticular hydrocarbons for identifying members of the Anopheles gambiae (Diptera: Culicidae) complex. *Bulletin of Entomological Research* 1993; **83**(4): 613-24.
20. Ant T, Foley E, Tytheridge S, et al. A survey of Anopheles species composition and insecticide resistance on the island of Bubaque, Bijagos Archipelago, Guinea-Bissau. *Malar J* 2020; **19**(1).
21. Service MW. A taxonomic study of Anopheles funestus funestus Giles (Diptera: Culicidae) from Southern and Northern Nigeria with notes on its varieties and synonyms *Syst Entomol* 1960; **29**(5-6): 77-84.
22. Pant CP, Joshi GP, Rosen P, et al. A village-scale trial of OMS-214 (Dicapthon) for the control of Anopheles gambiae and Anopheles funestus in northern Nigeria. *Bull World Health Organ* 1969; **41**(2): 311-&.
23. Barzeev M, Bracha P. A village-scale trial with the insecticides carbaryl and folithion in Southern Nigeria. *Bull World Health Organ* 1965; **33**(4): 461-70.
24. Oduola AO, Olojede JB, Oyewole IO, Otubanjo OA, Awolola TS. Abundance and diversity of Anopheles species (Diptera: Culicidae) associated with malaria transmission in human dwellings in rural and urban communities in Oyo State, Southwestern Nigeria. *Parasitology Research* 2013; **112**(10): 3433-9.
25. Leal SDV, Fernandes Varela IB, Lopes Gonçalves AA, et al. Abundance and Updated Distribution of Aedes aegypti (Diptera: Culicidae) in Cabo Verde Archipelago: A Neglected Threat to Public Health. *International Journal of Environmental Research and Public Health* 2020; **17**(4).
26. Djogbénou L, Labbé P, Chandre F, Pasteur N, Weill M. Ace-1 duplication in Anopheles gambiae: a challenge for malaria control. *Malar J* 2009; **8**(1).
27. Essandoh J, Yawson AE, Weetman D. Acetylcholinesterase (Ace-1) target site mutation 119S is strongly diagnostic of carbamate and organophosphate resistance in Anopheles gambiae s.s. and Anopheles coluzzii across southern Ghana. *Malar J* 2013; **12**(1).
28. Keïta M, Kané F, Thiero O, et al. Acetylcholinesterase (ace-1R) target site mutation G119S and resistance to carbamates in Anopheles gambiae (sensu lato) populations from Mali. *Parasites & Vectors* 2020; **13**(1).
29. Pinto J, Jones CM, Toé HK, et al. Additional Selection for Insecticide Resistance in Urban Malaria Vectors: DDT Resistance in Anopheles arabiensis from Bobo-Dioulasso, Burkina Faso. *PLoS ONE* 2012; **7**(9).
30. Chwatt LJ. Aedes (Stegomyia) pseudoafricanus sp. nov., a new species of Aedes from the coast of Nigeria, British West Africa. *Nature* 1949; **163**(4151): 808.
31. Lee VH. Aedes (Stegomyia) spp. utilizing Euphorbia kamerunica as larval habitat in Nigeria. *MOSQUITO NEWS* 1974; **34**(2): 229-31.

32. Konan LY, Coulibaly IZ, Kone BA, et al. Aedes aegypti Susceptibility to Insecticide from Abidjan City, Cote D'ivoire. *Vector-Borne and Zoonotic Diseases* 2012; **12**(4): 325-9.
33. Mint Lekweiry K, Ould Ahmedou Salem MS, Ould Brahim K, et al. Aedes aegypti(Diptera: Culicidae) in Mauritania: First Report on the Presence of the Arbovirus Mosquito Vector in Nouakchott. *Journal of Medical Entomology* 2015; **52**(4): 730-3.
34. Aedes albopictus introduction into continental Africa, 1991. *MMWR Morb Mortal Wkly Rep* 1991; **40**(48): 836-8.
35. Lehmann T, Yaro AS, Dao A, et al. Aestivation of the African Malaria Mosquito, Anopheles gambiae in the Sahel. *The American Journal of Tropical Medicine and Hygiene* 2010; **83**(3): 601-6.
36. Brunhes J, Le Goff G, Geoffroy B. Afro-tropical anopheline mosquitoes. III. Description of three new species: Anopheles carnevalei sp. nov., An. hervyi sp. nov., and An. dualaensis sp. nov., and resurrection of An. rageaui Mattingly and Adam. *J Am Mosq Control Assoc* 1999; **15**(4): 552-8.
37. Jones CM, Sanou A, Guelbeogo WM, Sagnon N, Johnson PC, Ranson H. Aging partially restores the efficacy of malaria vector control in insecticide-resistant populations of Anopheles gambiae s.l. from Burkina Faso. *Malar J* 2012; **11**: 24.
38. Jarju LBS, Fillinger U, Green C, Louca V, Majambere S, Lindsay SW. Agriculture and the promotion of insect pests: rice cultivation in river floodplains and malaria vectors in The Gambia. *Malar J* 2009; **8**(1).
39. Diallo M, Ba Y, Sall AA, et al. Amplification of the sylvatic cycle of dengue virus type 2, Senegal, 1999–2000: entomologic findings and epidemiologic considerations. *Emerging infectious diseases* 2003; **9**(3): 362.
40. Service MW. An analysis of individual and seasonal variations occurring in the wing ornamentation of Anopheles (Cellia) funestus Giles (Diptera: Culicidae). *Syst Entomol* 1964; **33**(7-8): 139-43.
41. Cansado-Utrilla C, Jeffries CL, Kristan M, et al. An assessment of adult mosquito collection techniques for studying species abundance and diversity in Maferinyah, Guinea. *Parasites & Vectors* 2020; **13**(1).
42. N'Guessan R, Asidi A, Boko P, et al. An experimental hut evaluation of PermaNet® 3.0, a deltamethrin–piperonyl butoxide combination net, against pyrethroid-resistant Anopheles gambiae and Culex quinquefasciatus mosquitoes in southern Benin. *Transactions of the Royal Society of Tropical Medicine and Hygiene* 2010; **104**(12): 758-65.
43. Bruce-Chwatt LJ. An unusual epidemiology of malaria in South-Eastern Nigeria. *Transactions of the Royal Society of Tropical Medicine and Hygiene* 1957; **51**(5): 411-8.
44. Doucoure S, Thiaw O, Wotodjo AN, et al. Anopheles arabiensis and Anopheles funestus biting patterns in Dielmo, an area of low level exposure to malaria vectors. *Malar J* 2020; **19**(1).
45. Chitnis N, Soma DD, Zogo BM, et al. Anopheles bionomics, insecticide resistance and malaria transmission in southwest Burkina Faso: A pre-intervention study. *Plos One* 2020; **15**(8).
46. Sovi A, Keita C, Sinaba Y, et al. Anopheles gambiae (s.l.) exhibit high intensity pyrethroid resistance throughout Southern and Central Mali (2016–2018): PBO or next generation LLINs may provide greater control. *Parasites & Vectors* 2020; **13**(1).
47. Quiñones ML, Lines JD, Thomson MC, Jawara M, Morris J, Greenwood BM. Anopheles gambiae gonotrophic cycle duration, biting and exiting behaviour unaffected by permethrin-impregnated bednets in The Gambia. *Med Vet Entomol* 1997; **11**(1): 71-8.
48. Fane M, Cissé O, Traore CSF, Sabatier P. Anopheles gambiae resistance to pyrethroid-treated nets

in cotton versus rice areas in Mali. *Acta Tropica* 2012; **122**(1): 1-6.
49. Assogba BS, Sillah S, Opondo KO, et al. Anopheles gambiae s.l. swarms trapping as a complementary tool against residual malaria transmission in eastern Gambia. *Scientific Reports* 2022; **12**(1).
50. Awolola TS, Oduola AO, Obansa JB, Chukwurar NJ, Unyimadu JP. Anopheles gambiae s.s. breeding in polluted water bodies in urban Lagos, southwestern Nigeria. *J Vector Borne Dis* 2007; **44**(4): 241-4.
51. Dery DB, Segbaya S, Asoalla V, et al. Anopheles gambiae(Diptera: Culicidae) Susceptibility to Insecticides and Knockdown Resistance Genes Prior to Introduction of Indoor Residual Spraying in 11 Districts in Ghana. *Journal of Medical Entomology* 2016; **53**(6): 1403-9.
52. Gelfand HM. Anopheles gambias Giles and Anopheles melas Theobald in a coastal area of Liberia, West Africa. *Transactions of the Royal Society of Tropical Medicine and Hygiene* 1955; **49**(6): 508-27.
53. Labbo R, Czeher C, Djibrila A, Arzika I, Jeanne I, Duchemin JB. Anopheles hervyi in Niger: no evidence for a role in Plasmodium falciparum transmission. *Med Vet Entomol* 2010; **24**(1): 62-5.
54. Froud MD. Anopheles JebudensisSp. Nov., A New Anopheline Mosquito from Southern Nigeria. *Annals of Tropical Medicine & Parasitology* 2016; **38**(1): 73-7.
55. Carrara GC, Petrarca V, Niang M, Coluzzi M. Anopheles pharoensis and transmission of Plasmodium falciparum in the Senegal River delta, West Africa. *Med Vet Entomol* 1990; **4**(4): 421-4.
56. Egbuche CM, Onyido AE, Umeanaeto PU, et al. Anopheles species composition and some climatic factors that influence their survival and population abundance in Anambra East LGA, Anambra State, Nigeria. *Nigerian Journal of Parasitology* 2020; **41**(2): 240-50.
57. Zogo B, Soma DD, Tchiekoi BNC, et al. Anophelesbionomics, insecticide resistance mechanisms, and malaria transmission in the Korhogo area, northern Côte d'Ivoire: a pre-intervention study. *Parasite* 2019; **26**.
58. Traoré A, Badolo A, Guelbeogo MW, et al. Anopheline species composition and the 1014F-genotype in different ecological settings of Burkina Faso in relation to malaria transmission. *Malar J* 2019; **18**(1).
59. Charles LJ, Vincke IH, Vanderka.Hj, Brady J. The appearance of pyrimethamine resistance in Plasmodium falciparum following self-medication by a rural community in Ghana. *Bull World Health Organ* 1962; **26**(1): 103-&.
60. Ndiaye EH, Diallo D, Fall G, et al. Arboviruses isolated from the Barkedji mosquito-based surveillance system, 2012-2013. *BMC Infectious Diseases* 2018; **18**(1).
61. Ba Y, Diallo D, Kebe CMF, Dia I, Diallo M. Aspects of bioecology of two rift valley fever virus vectors in Senegal (West Africa): Aedes vexans and Culex poicilipes (Diptera: Culicidae). *Journal of Medical Entomology* 2005; **42**(5): 739-50.
62. Dossou-Yovo J, Ouattara A, Doannio JM, Riviere F, Chauvancy G, Meunier JY. [Aspects of malaria in a village of the humid savannah of the Ivory Coast]. *Medecine tropicale : revue du Corps de sante colonial* 1994; **54**(4): 331-6.
63. Kpanou CD, Sagbohan HW, Sovi A, et al. Assessing Insecticide Susceptibility and Resistance Intensity of Anopheles gambiae s.l. Populations From Some Districts of Benin Republic, West Africa. *Journal of Medical Entomology* 2022; **59**(3): 949-56.
64. Kouassi BL, de Souza DK, Goepogui A, et al. Assessing the presence of Wuchereria bancrofti in vector and human populations from urban communities in Conakry, Guinea. *Parasites & Vectors* 2015; **8**(1).

65. Pi-Bansa S, Osei J, Kartey-Attipoe W, et al. Assessing the Presence of Wuchereria bancrofti Infections in Vectors Using Xenomonitoring in Lymphatic Filariasis Endemic Districts in Ghana. *Tropical Medicine and Infectious Disease* 2019; **4**(1).
66. Coulibaly S, Sawadogo SP, Nikièma AS, et al. Assessment of Culicidae collection methods for xenomonitoring lymphatic filariasis in malaria co-infection context in Burkina Faso. 2022.
67. Idowu OA, Adeleke MA, Oriaku EC. Assessment of natural and artificial breeding sites of mosquitoes in rural and urban areas of Abeokuta, Ogun State, Nigeria. *Munis Entomology & Zoology* 2014; **9**(2): 832-7.
68. Sanou R, Maïga H, Bilgo EM, et al. Assessment of novel Lehmann's funnel entry trap prototypes performance to control malaria mosquito populations. *Malar J* 2021; **20**(1).
69. Dabire KR, Sawadodgo S, Diabate A, et al. Assortative mating in mixed swarms of the mosquitoAnopheles gambiae s.s. M and S molecular forms, in Burkina Faso, West Africa. *Medical and Veterinary Entomology* 2013; **27**(3): 298-312.
70. Ebenezer A, Ben HIB, Enaregha EB. Atial distribution and indoor-resting density of mosquito species in the lowland rainforest of Bayelsa state, Nigeria. *International Journal of Tropical Medicine* 2013; **8**(4): 87-91.
71. Zoure AA, Sare AR, Yameogo F, et al. Bacterial communities associated with the midgut microbiota of wild Anopheles gambiae complex in Burkina Faso. *Molecular Biology Reports* 2019; **47**(1): 211-24.
72. Salako AS, Ahogni I, Kpanou C, et al. Baseline entomologic data on malaria transmission in prelude to an indoor residual spraying intervention in the regions of Alibori and Donga, Northern Benin, West Africa. *Malar J* 2018; **17**(1).
73. Dery DB, Asante KP, Zandoh C, et al. Baseline malaria vector transmission dynamics in communities in Ahafo mining area in Ghana. *Malar J* 2015; **14**(1).
74. Oduola AO, Awe OO. Behavioural biting preference of Culex quinquefasciatus in human host in Lagos metropolis Nigeria. *J Vector Borne Dis* 2006; **43**(1): 16-20.
75. Guelbeogo WM, Sagnon N, Liu F, Besansky NJ, Costantini C. Behavioural divergence of sympatric Anopheles funestus populations in Burkina Faso. *Malar J* 2014; **13**: 65.
76. Perugini E, Guelbeogo WM, Calzetta M, et al. Behavioural plasticity of Anopheles coluzzii and Anopheles arabiensis undermines LLIN community protective effect in a Sudanese-savannah village in Burkina Faso. *Parasites & Vectors* 2020; **13**(1).
77. Gimonneau G, Pombi M, Dabiré RK, Diabaté A, Morand S, Simard F. Behavioural responses of Anopheles gambiae sensu stricto M and S molecular form larvae to an aquatic predator in Burkina Faso. *Parasit Vectors* 2012; **5**: 65.
78. Wahedi JA, Ande AT, Oduola AO, Obembe A. Bendiocarb resistance and, *kdr* associated deltamethrin and DDT resistance in *Anopheles gambiae s.l.* populations from North Eastern Adamawa State, Nigeria. *Ceylon Journal of Science* 2021; **50**(1).
79. Aïkpon R, Agossa F, Ossè R, et al. Bendiocarb resistance in Anopheles gambiae s.l. populations from Atacora department in Benin, West Africa: a threat for malaria vector control. *Parasit Vectors* 2013; **6**: 192.
80. Akogbéto MC, Padonou GG, Gbénou D, Irish S, Yadouleton A. Bendiocarb, a potential alternative against pyrethroid resistant Anopheles gambiae in Benin, West Africa. *Malar J* 2010; **9**: 204.
81. Yusuf MA, Vatandoost H, Oshaghi MA, et al. Biochemical Mechanism of Insecticide Resistance in Malaria Vector, Anopheles gambiae s.l in Nigeria. *Iran J Public Health* 2021; **50**(1): 101-10.

82. Abagli AZ, T.b.c A. Biocontrol of culex quinquefasciatus using the insect parasitic nematode, romanomermis iyengari (Nematoda: Mermithidae). *Tropical Biomedicine* 2019; **36**(4): 1003-13.

83. Diallo D, Diagne CT, Buenemann M, et al. Biodiversity Pattern of Mosquitoes in Southeastern Senegal, Epidemiological Implication in Arbovirus and Malaria Transmission. *Journal of Medical Entomology* 2019; **56**(2): 453-63.

84. Dambach P, Winkler V, Bärnighausen T, et al. Biological larviciding against malaria vector mosquitoes with Bacillus thuringiensis israelensis (Bti) – Long term observations and assessment of repeatability during an additional intervention year of a large-scale field trial in rural Burkina Faso. *Global Health Action* 2020; **13**(1).

85. Guindo-Coulibaly N, Diakite NR, Adja AM, et al. Biology of two larval morphological phenotypes ofAedes aegyptiin Abidjan, Côte d'Ivoire. *Bulletin of Entomological Research* 2017; **108**(4): 540-6.

86. Dia I, Diop T, Rakotoarivony I, Kengne P, Fontenille D. Bionomics of *Anopheles gambiae* Giles, *An. arabiensis* Patton, *An. funestus* Giles and *An. nili* (Theobald) (Diptera: Culicidae) and Transmission of *Plasmodium falciparum* in a Sudano-Guinean Zone (Ngari, Senegal). *Journal of Medical Entomology* 2003; **40**(3): 279-83.

87. Dia I, Konate L, Samb B, et al. Bionomics of malaria vectors and relationship with malaria transmission and epidemiology in three physiographic zones in the Senegal River Basin. *Acta Tropica* 2008; **105**(2): 145-53.

88. Norris D, Dadzie SK, Adjei Boakye D, et al. Biting Behavior and Molecular Identification of Aedes aegypti (Diptera: Culicidae) Subspecies in Some Selected Recent Yellow Fever Outbreak Communities in Northern Ghana. *Journal of Medical Entomology* 2020; **57**(4): 1239-45.

89. Sougoufara S, Diédhiou SM, Doucouré S, et al. Biting by Anopheles funestus in broad daylight after use of long-lasting insecticidal nets: A new challenge to malaria elimination. *Malar J* 2014; **13**(1).

90. Akogbéto MC, Salako AS, Dagnon F, et al. Blood feeding behaviour comparison and contribution of Anopheles coluzzii and Anopheles gambiae, two sibling species living in sympatry, to malaria transmission in Alibori and Donga region, northern Benin, West Africa. *Malar J* 2018; **17**(1).

91. Sene NM, Diouf B, Gaye A, et al. Blood Feeding Patterns of Aedes aegypti Populations in Senegal. *The American Journal of Tropical Medicine and Hygiene* 2022; **106**(5): 1402-5.

92. Tuno N, Kjaerandsen J, Badu K, Kruppa T. Blood-Feeding Behavior of *Anopheles gambiae* and *Anopheles melas* in Ghana, Western Africa. *Journal of Medical Entomology* 2010; **47**(1): 28-31.

93. Diallo D, Chen R, Diagne CT, et al. Bloodfeeding patterns of sylvatic arbovirus vectors in southeastern Senegal. *Transactions of the Royal Society of Tropical Medicine and Hygiene* 2013; **107**(3): 200-3.

94. Nwakanma DC, Neafsey DE, Jawara M, et al. Breakdown in the Process of Incipient Speciation inAnopheles gambiae. *Genetics* 2013; **193**(4): 1221-31.

95. Blacklock B. Breeding places of anopheline mosquitoes in freetown, sierra leone. *Ann Trop Med Parasitol* 1921; **15**(4): 463-71.

96. Blacklock DB, Evans AM. Breeding places of anopheline mosquitos in and around freetown, sierra leone. *Ann Trop Med Parasitol* 1926; **20**(1): 59-84.

97. Syme T, Gbegbo M, Obuobi D, et al. Can pyrethroid-piperonyl butoxide (PBO) nets reduce the efficacy of indoor residual spraying with pirimiphos-methyl against pyrethroid-resistant malaria vectors? ; 2021.

98. Moiroux N, Gomez MB, Pennetier C, et al. Changes in Anopheles funestus Biting Behavior

Following Universal Coverage of Long-Lasting Insecticidal Nets in Benin. *The Journal of Infectious Diseases* 2012; **206**(10): 1622-9.
99. Okoye PN, Brooke BD, Koekemoer LL, Hunt RH, Coetzee M. Characterisation of DDT, pyrethroid and carbamate resistance in Anopheles funestus from Obuasi, Ghana. *Transactions of the Royal Society of Tropical Medicine and Hygiene* 2008; **102**(6): 591-8.
100. Owusu EDA, Buabeng V, Dadzie S, Brown CA, Grobusch MP, Mens P. Characteristics of asymptomatic Plasmodium spp. parasitaemia in Kwahu-Mpraeso, a malaria endemic mountainous district in Ghana, West Africa. *Malar J* 2016; **15**(1).
101. Kabula BI, Attah PK, Wilson MD, Boakye DA. Characterization of Anopheles gambiae s.l. and insecticide resistance profile relative to physicochemical properties of breeding habitats within Accra Metropolis, Ghana. *Tanzania Journal of Health Research* 2011; **13**(3): 163-87.
102. Tchouassi DP, Quakyi IA, Addison EA, et al. Characterization of malaria transmission by vector populations for improved interventions during the dry season in the Kpone-on-Sea area of coastal Ghana. *Parasit Vectors* 2012; **5**: 212.
103. Kpanou CD, Sagbohan HW, Dagnon F, et al. Characterization of resistance profile (intensity and mechanisms) of Anopheles gambiae in three communes of northern Benin, West Africa. *Malar J* 2021; **20**(1).
104. Assogba BS, Djogbénou L, Saizonou J, et al. Characterization of swarming and mating behaviour between Anopheles coluzzii and Anopheles melas in a sympatry area of Benin. *Acta Tropica* 2014; **132**: S53-S63.
105. Stica C, Jeffries CL, Irish SR, et al. Characterizing the molecular and metabolic mechanisms of insecticide resistance in Anopheles gambiae in Faranah, Guinea. *Malar J* 2019; **18**(1).
106. Sow A, Faye O, Diallo M, et al. Chikungunya Outbreak in Kedougou, Southeastern Senegal in 2009–2010. *Open Forum Infectious Diseases* 2018; **5**(1).
107. Costantini C, Sagnon N, Ilboudo-Sanogo E, Coluzzi M, Boccolini D. Chromosomal and bionomic heterogeneities suggest incipient speciation in Anopheles funestus from Burkina Faso. *Parassitologia* 1999; **41**(4): 595-611.
108. Guelbeogo WM, Grushko O, Boccolini D, et al. Chromosomal evidence of incipient speciation in the Afrotropical malaria mosquito Anopheles funestus. *Med Vet Entomol* 2005; **19**(4): 458-69.
109. Guedes DRD, Gomes ETB, Paiva MHS, et al. Circulation of DENV2 and DENV4 in Aedes aegypti (Diptera: Culicidae) mosquitoes from Praia, Santiago Island, Cabo Verde. *Journal of Insect Science* 2017; **17**(4).
110. Ngufor C, N'Guessan R, Boko P, et al. Combining indoor residual spraying with chlorfenapyr and long-lasting insecticidal bed nets for improved control of pyrethroid-resistant Anopheles gambiae: an experimental hut trial in Benin. *Malar J* 2011; **10**: 343.
111. Ngufor C, Chouaïbou M, Tchicaya E, et al. Combining organophosphate-treated wall linings and long-lasting insecticidal nets fails to provide additional control over long-lasting insecticidal nets alone against multiple insecticide-resistant Anopheles gambiae in Côte d'Ivoire: an experimental hut trial. *Malar J* 2014; **13**: 396.
112. Armel D, Fabrice C, Razaki O, Joseph C, Martin A, Vincent C. Community use of long-lasting insecticidal net in combination with carbamate treated plastic sheeting for insecticide resistance management in malaria vectors. *American Journal of Tropical Medicine and Hygiene* 2010; **83**(5): 52.
113. Ngufor C, Fagbohoun J, Agbevo A, et al. Comparative efficacy of two pyrethroid-piperonyl butoxide nets (Olyset Plus and PermaNet 3.0) against pyrethroid resistant malaria vectors: a non-

inferiority assessment. *Malar J* 2022; **21**(1).
114. Fanello C, Carneiro I, Ilboudo-Sanogo E, Cuzin-Ouattara N, Badalo A, Curtis CF. Comparative evaluation of carbosulfan- and permethrin-impregnated curtains for preventing house-entry by the malaria vector Anopheles gambiae in Burkina Faso. *MEDICAL AND VETERINARY ENTOMOLOGY* 2003; **17**(3): 333-8.
115. Sokhna CS, Diagne N, Lochouarn L, et al. [Comparative evaluation of the plasmodial infection of Anopheles using ELISA and dissection. Consequences for the estimation of the transmission of malaria in 1995 in Ndiop, Senegal]. *Parasite* 1998; **5**(3): 273-9.
116. Hogg JC, Thomson MC, Hurd H. Comparative fecundity and associated factors for two sibling species of the Anopheles gambiae complex occurring sympatrically in The Gambia. *Med Vet Entomol* 1996; **10**(4): 385-91.
117. Tchouakui M, Assatse T, Mugenzi LMJ, et al. Comparative study of the effect of solvents on the efficacy of neonicotinoid insecticides against malaria vector populations across Africa. *Infectious Diseases of Poverty* 2022; **11**(1).
118. Aïzoun N, Aïkpon R, Azondekon R, Asidi A, Akogbéto M. Comparative susceptibility to permethrin of two Anopheles gambiae s.l. populations from Southern Benin, regarding mosquito sex, physiological status, and mosquito age. *Asian Pacific Journal of Tropical Biomedicine* 2014; **4**(4): 312-7.
119. Ndiath MO, Cohuet A, Gaye A, et al. Comparative susceptibility to Plasmodium falciparum of the molecular forms M and S of Anopheles gambiae and Anopheles arabiensis. *Malar J* 2011; **10**.
120. Amusan AAS, Idowu AB, Arowolo FS. Comparative toxicity effect of bush tea leaves (*Hyptis suaveolens*) and orange peel (*Citrus sinensis*) oil extract on larvae of the yellow fever mosquito *Aedes aegypti*. *Tanzania Journal of Health Research* 2006; **7**(3).
121. Lemasson JJ, Fontenille D, Lochouarn L, et al. Comparison of behavior and vector efficiency of Anopheles gambiae and An. arabiensis (Diptera:Culicidae) in Barkedji, a Sahelian area of Senegal. *J Med Entomol* 1997; **34**(4): 396-403.
122. Jawara M, Pinder M, Cham B, Walraven G, Rowley J. Comparison of deltamethrin tablet formulation with liquid deltamethrin and permethrin for bednet treatment in The Gambia. *Trop Med Int Health* 2001; **6**(4): 309-16.
123. Coulibaly YI, Sangare M, Dolo H, et al. Comparison of Different Sampling Methods to Catch Lymphatic Filariasis Vectors in a Sudan Savannah Area of Mali. *The American Journal of Tropical Medicine and Hygiene* 2022; **106**(4): 1247-53.
124. Awono-Ambene HP, Diawara L, Robert V. Comparison of direct and membrane feeding methods to infect Anopheles arabiensis with Plasmodium falciparum. *American Journal of Tropical Medicine and Hygiene* 2001; **64**(1-2): 32-4.
125. Gouagna LC, Yao F, Yameogo B, Dabiré RK, Ouédraogo J-B. Comparison of field-based xenodiagnosis and direct membrane feeding assays for evaluating host infectiousness to malaria vector Anopheles gambiae. *Acta Tropica* 2014; **130**: 131-9.
126. Fontenille D, Faye O, Konate L, Sy N, Collins FH. [Comparison of PCR and cytogenetic methods for the identification of mosquito species of the Anopheles gambiae complex in Senegal]. *Ann Parasitol Hum Comp* 1993; **68**(5-6): 239-40.
127. Aïzoun N, Ossè R, Azondekon R, et al. Comparison of the standard WHO susceptibility tests and the CDC bottle bioassay for the determination of insecticide susceptibility in malaria vectors and their correlation with biochemical and molecular biology assays in Benin, West Africa. *Parasit Vectors* 2013;

6: 147.
128. Dia I, Diallo D, Duchemin JB, et al. Comparisons of human-landing catches and odor-baited entry traps for sampling malaria vectors in Senegal. *J Med Entomol* 2005; **42**(2): 104-9.
129. Lanzaro GC, Touré YT, Carnahan J, et al. Complexities in the genetic structure of Anopheles gambiae populations in west Africa as revealed by microsatellite DNA analysis. *Proc Natl Acad Sci U S A* 1998; **95**(24): 14260-5.
130. Cuesta EB, Coulibaly B, Bukhari T, et al. Comprehensive Ecological and Geographic Characterization of Eukaryotic and Prokaryotic Microbiomes in African Anopheles. *Frontiers in Microbiology* 2021; **12**.
131. Diallo D, Fall G, Diagne CT, et al. Concurrent amplification of Zika, chikungunya, and yellow fever virus in a sylvatic focus of arboviruses in Southeastern Senegal, 2015. *BMC Microbiology* 2020; **20**(1).
132. Gadiaga L, MacHault V, Pagès F, et al. Conditions of malaria transmission in Dakar from 2007 to 2010. *Malar J* 2011; **10**.
133. Foll CV, Pant CP. The conditions of malaria transmission in Katsina Province, Northern Nigeria, and a discussion of the effects of dichlorvos application. *Bull World Health Organ* 1966; **34**(3): 395-&.
134. Huho B, Briët O, Seyoum A, et al. Consistently high estimates for the proportion of human exposure to malaria vector populations occurring indoors in rural Africa. *International Journal of Epidemiology* 2013; **42**(1): 235-47.
135. Adja AM, N'goran EK, Koudou BG, et al. Contribution ofAnopheles funestus,An. gambiaeandAn. nili(Diptera: Culicidae) to the perennial malaria transmission in the southern and western forest areas of Côte d'Ivoire. *Annals of Tropical Medicine & Parasitology* 2013; **105**(1): 13-24.
136. Jaenson TG, Gomes MJ, Barreto dos Santos RC, et al. Control of endophagic Anopheles mosquitoes and human malaria in Guinea Bissau, West Africa by permethrin-treated bed nets. *Trans R Soc Trop Med Hyg* 1994; **88**(6): 620-4.
137. N'Guessan R, Boko P, Odjo A, Knols B, Akogbeto M, Rowland M. Control of pyrethroid-resistantAnopheles gambiaeandCulex quinquefasciatusmosquitoes with chlorfenapyr in Benin. *Tropical Medicine & International Health* 2009; **14**(4): 389-95.
138. Yadouleton A, Martin T, Padonou G, et al. Cotton pest management practices and the selection of pyrethroid resistance in Anopheles gambiae population in northern Benin. *Parasit Vectors* 2011; **4**: 60.
139. Soumaila H, Hamani B, Arzika II, et al. Countrywide insecticide resistance monitoring and first report of the presence of the L1014S knock down resistance in Niger, West Africa. *Malar J* 2022; **21**(1).
140. Sovi A, Gnanguenon V, Azondekon R, et al. Coverage, Usage, Physical Integrity, and Bio-efficacy of Olyset Nets in the Plateau Region, South Benin Following the 2011 Nationwide Distribution. *Journal of Medical Entomology* 2022; **59**(1): 337-49.
141. Lombardi S, Esposito F, Zavala F, et al. CS antigen localization in malaria vectors: hypothetical refractoriness to transmission observed in the field. *Parassitologia* 1986; **28**(2-3): 113-6.
142. Taconet P, Porciani A, Soma DD, et al. Data-driven and interpretable machine-learning modeling to explore the fine-scale environmental determinants of malaria vectors biting rates in rural Burkina Faso. *Parasites & Vectors* 2021; **14**(1).
143. Padonou GG, Gbedjissi G, Yadouleton A, et al. Decreased proportions of indoor feeding and endophily in Anopheles gambiae s.l. populations following the indoor residual spraying and insecticide-treated net interventions in Benin (West Africa). *Parasit Vectors* 2012; **5**: 262.
144. Traore-Lamizana M, Zeller H, Monlun E, et al. Dengue 2 outbreak in southeastern Senegal during

1990: virus isolations from mosquitoes (Diptera: Culicidae). *J Med Entomol* 1994; **31**(4): 623-7.
145. Robin Y, Cornet M, Heme G, Le Gonidec G. Dengue virus isolation in Senegal. *Annales de l'Institut Pasteur Virologie* 1980; **131**(2): 149-54.
146. Costantini C, Li SG, Della Torre A, Sagnon N, Coluzzi M, Taylor CE. Density, survival and dispersal of Anopheles gambiae complex mosquitoes in a west African Sudan savanna village. *Med Vet Entomol* 1996; **10**(3): 203-19.
147. Lee Y, Meneses CR, Fofana A, Lanzaro GC. Desiccation Resistance Among Subpopulations ofAnopheles gambiaes.s. From Selinkenyi, Mali. *Journal of Medical Entomology* 2009; **46**(2): 316-20.
148. Tchouakui M, Assatse T, Tazokong HR, et al. Detection of a reduced susceptibility to chlorfenapyr in the malaria vector Anopheles gambiae contrasts with full susceptibility in Anopheles funestus across Africa. *Scientific Reports* 2023; **13**(1).
149. da Cruz DL, Paiva MHS, Guedes DRD, Alves J, Gómez LF, Ayres CFJ. Detection of alleles associated with resistance to chemical insecticide in the malaria vector Anopheles arabiensis in Santiago, Cabo Verde. *Malar J* 2019; **18**(1).
150. Sy O, Sarr PC, Assogba BS, et al. Detection of kdr and ace-1 mutations in wild populations of Anopheles arabiensis and An. melas in a residual malaria transmission area of Senegal. *Pesticide Biochemistry and Physiology* 2021; **173**.
151. Appawu MA, Bosompem KM, Dadzie S, et al. Detection of malaria sporozoites by standard ELISA and VecTestTM dipstick assay in field-collected anopheline mosquitoes from a malaria endemic site in Ghana. *Trop Med Int Health* 2003; **8**(11): 1012-7.
152. Agwu EJ, Isaac C, Igbinosa IB. Detection of yellow fever and dengue viruses in mosquitoes between 2014 and 2015 in Bayelsa and Benue states of Nigeria. *Acta Entomologica Serbica* 2019; **24**(1): 59-78.
153. Amoa-Bosompem M, Kobayashi D, Itokawa K, et al. Determining vector competence of Aedes aegypti from Ghana in transmitting dengue virus serotypes 1 and 2. *Parasites & Vectors* 2021; **14**(1).
154. Yadouleton AWM, Asidi A, Djouaka RF, Braïma J, Agossou CD, Akogbeto MC. Development of vegetable farming: a cause of the emergence of insecticide resistance in populations of Anopheles gambiae in urban areas of Benin. *Malar J* 2009; **8**(1).
155. Fryxell RT, Nieman CC, Fofana A, et al. Differential Plasmodium falciparum infection of Anopheles gambiae s.s. molecular and chromosomal forms in Mali. *Malar J* 2012; **11**: 133.
156. Wilson MD, Ofosu-Okyere A, Okoli AU, McCall PJ, Snounou G. Direct comparison of microscopy and polymerase chain reaction for the detection of Plasmodium sporozoites in salivary glands of mosquitoes. *Transactions of the Royal Society of Tropical Medicine and Hygiene* 1998; **92**(5): 482-3.
157. Hermanns K, Zirkel F, Kopp A, et al. Discovery of a novel alphavirus related to Eilat virus. *Journal of General Virology* 2017; **98**(1): 43-9.
158. Marklewitz M, Zirkel F, Rwego IB, et al. Discovery of a Unique Novel Clade of Mosquito-Associated Bunyaviruses. *Journal of Virology* 2013; **87**(23): 12850-65.
159. McCall PJ, Kawada H, Higa Y, et al. Discovery of Point Mutations in the Voltage-Gated Sodium Channel from African Aedes aegypti Populations: Potential Phylogenetic Reasons for Gene Introgression. *PLOS Neglected Tropical Diseases* 2016; **10**(6).
160. Sabatinelli G, Rossi P, Belli A. [Dispersion of Anopheles gambiae s.l. in an urban zone of Ouagadougou (Burkina Faso)]. *Parassitologia* 1986; **28**(1): 33-9.
161. Ikpa GI, Mafuyai HB, Ikpa TF. Distribution and infectivity of anopheles mosquitoes and

asymptomatic malaria infection in a home-setting of Gboko, Nigeria. *Nigerian Journal of Parasitology* 2017; **38**(1).
162. Amaechi EC, Mkpola Ukpai O, Chima Ohaeri C, et al. Distribution and seasonal abundance of Anopheline mosquitoes and their association with rainfall around irrigation and non-irrigation areas in Nigeria. *UNED Research Journal* 2018; **10**(2): 267-72.
163. Guindo-Coulibaly N, Adja AM, Koudou BG, et al. Distribution and seasonal variation of aedes aegypti in the health district of Abidjan (Côte d'Ivoire). *Eur J Sci Res* 2010; **40**(4): 522-30.
164. Ahoua Alou LP, Koffi AA, Adja MA, et al. Distribution of ace-1R and resistance to carbamates and organophosphates in Anopheles gambiae s.s. populations from Côte d'Ivoire. *Malar J* 2010; **9**: 167.
165. Dabiré KR, Diabaté A, Namontougou M, et al. Distribution of insensitive acetylcholinesterase (ace-1R) inAnopheles gambiaes.l. populations from Burkina Faso (West Africa). *Tropical Medicine & International Health* 2009; **14**(4): 396-403.
166. Yayock HC, Ndams IS, Kogi E, Ahmed AB, George JB. Distribution of mosquito species in Kaduna Metropolis, Kaduna State Northern Nigeria. *American Journal of Tropical Medicine and Hygiene* 2019; **101**(5): 50.
167. Chandre F, Darriet F, Doannio JM, Rivière F, Pasteur N, Guillet P. Distribution of organophosphate and carbamate resistance in Culex pipiens quinquefasciatus (Diptera: Culicidae) in West Africa. *J Med Entomol* 1997; **34**(6): 664-71.
168. Dabiré KR, Diabaté A, Namountougou M, et al. Distribution of pyrethroid and DDT resistance and the L1014F kdr mutation in Anopheles gambiae s.l. from Burkina Faso (West Africa). *Transactions of the Royal Society of Tropical Medicine and Hygiene* 2009; **103**(11): 1113-20.
169. Dabiré KR, Diabaté A, Agostinho F, et al. Distribution of the members of Anopheles gambiae and pyrethroid knock-down resistance gene (kdr) in Guinea-Bissau, West Africa. *Bull Soc Pathol Exot* 2008; **101**(2): 119-23.
170. Awolola TS, Oyewole IO, Amajoh CN, et al. Distribution of the molecular forms of Anopheles gambiae and pyrethroid knock down resistance gene in Nigeria. *Acta Tropica* 2005; **95**(3): 204-9.
171. Roux O, Diabaté A, Simard F, Wilson K. Divergence in threat sensitivity among aquatic larvae of cryptic mosquito species. *Journal of Animal Ecology* 2014; **83**(3): 702-11.
172. Quiñones ML, Drakeley CJ, Müller O, Lines JD, Haywood M, Greenwood BM. Diversion of Anopheles gambiae from children to other hosts following exposure to permethrin-treated bednets. *Medical and Veterinary Entomology* 2000; **14**(4): 369-75.
173. Fournet F, Cussac M, Ouari A, et al. Diversity in anopheline larval habitats and adult composition during the dry and wet seasons in Ouagadougou (Burkina Faso). *Malar J* 2010; **9**.
174. Mattah PAD, Futagbi G, Amekudzi LK, et al. Diversity in breeding sites and distribution of Anopheles mosquitoes in selected urban areas of southern Ghana. *Parasites & Vectors* 2017; **10**(1).
175. Toe KH, Müller P, Badolo A, et al. Do bednets including piperonyl butoxide offer additional protection against populations of Anopheles gambiae s.l. that are highly resistant to pyrethroids? An experimental hut evaluation in Burkina Fasov. *Medical and Veterinary Entomology* 2018; **32**(4): 407-16.
176. Niang A, Epopa PS, Sawadogo SP, et al. Does extreme asymmetric dominance promote hybridization between Anopheles coluzzii and Anopheles gambiae s.s. in seasonal malaria mosquito communities of West Africa? *Parasites & Vectors* 2015; **8**(1).
177. Afrane YA, Klinkenberg E, Drechsel P, Owusu-Daaku K, Garms R, Kruppa T. Does irrigated urban agriculture influence the transmission of malaria in the city of Kumasi, Ghana? *Acta Tropica*

2004; **89**(2): 125-34.
178. Djouaka RF, Bakare AA, Bankole HS, et al. Does the spillage of petroleum products in Anopheles breeding sites have an impact on the pyrethroid resistance? *Malar J* 2007; **6**(1).
179. Johnson WB. Domestic mosquitos of the northern provinces of nigeria. *Bulletin of Entomological Research* 1919; **9**(4): 325-32.
180. Akogbeto M, Gbedjissi GL, Padonou GG, Gazard DK, Bankole HS. Dramatic Decrease in Malaria Transmission after Large-Scale Indoor Residual Spraying with Bendiocarb in Benin, an Area of High Resistance of Anopheles gambiae to Pyrethroids. *The American Journal of Tropical Medicine and Hygiene* 2011; **85**(4): 586-93.
181. Jawara M, Pinder M, Drakeley CJ, et al. Dry season ecology of Anopheles gambiae complex mosquitoes in The Gambia. *Malar J* 2008; **7**(1).
182. Oforka CL, Adeleke MA. DRY SEASON LARVAL HABITATS OF MOSQUITO VECTORS (CULICIDAE) IN AJEROMI-IFELODUN AREA OF LAGOS, NIGERIA. *Munis Entomology & Zoology* 2022; **17**(1): 252-9.
183. Coulibaly YI, Coulibaly SY, Dolo H, et al. Dynamics of antigenemia and transmission intensity of Wuchereria bancrofti following cessation of mass drug administration in a formerly highly endemic region of Mali. *Parasites & Vectors* 2016; **9**(1).
184. Namountougou M, Dabire RK, Etang J, et al. Dynamics of insecticide resistance in Anopheles gambiae S.L. according to cotton cultivation schemes in Burkina Faso, West Africa. *American Journal of Tropical Medicine and Hygiene* 2011; **85**(6): 133.
185. Djègbè I, Boussari O, Sidick A, et al. Dynamics of insecticide resistance in malaria vectors in Benin: first evidence of the presence of L1014S kdr mutation in Anopheles gambiae from West Africa. *Malar J* 2011; **10**: 261.
186. Awolola TS, Oduola AO, Oyewole IO, et al. Dynamics of knockdown pyrethroid insecticide resistance alleles in a field population of Anopheles gambiae s.s. in southwestern Nigeria. *J Vector Borne Dis* 2007; **44**(3): 181-8.
187. Tiono AB, Guelbeogo MW, Sagnon NF, et al. Dynamics of malaria transmission and susceptibility to clinical malaria episodes following treatment of Plasmodium falciparum asymptomatic carriers: results of a cluster-randomized study of community-wide screening and treatment, and a parallel entomology study. *BMC Infect Dis* 2013; **13**: 535.
188. Dynamics of malaria vector indices in two vegetation zones within North Eastern Adamawa State, Nigeria. *Tropical Biomedicine* 2020; **37**(3): 637-49.
189. Dabiré KR, Diabaté A, Djogbenou L, et al. Dynamics of multiple insecticide resistance in the malaria vector Anopheles gambiae in a rice growing area in South-Western Burkina Faso. *Malar J* 2008; **7**(1).
190. Awolola TS, Okwa O, Hunt RH, Ogunrinade AF, Coetzee M. Dynamics of the malaria-vector populations in coastal Lagos, south–western Nigeria. *Annals of Tropical Medicine & Parasitology* 2013; **96**(1): 75-82.
191. Ndiath MO, Brengues C, Konate L, et al. Dynamics of transmission of Plasmodium falciparum by Anopheles arabiensis and the molecular forms M and S of Anopheles gambiae in Dielmo, Senegal. *Malar J* 2008; **7**(1).
192. Biteye B, Fall AG, Ciss M, et al. Ecological distribution and population dynamics of Rift Valley fever virus mosquito vectors (Diptera, Culicidae) in Senegal. *Parasites & Vectors* 2018; **11**(1).
193. Omar K, Thabet HS, TagEldin RA, et al. Ecological niche modeling for predicting the potential

geographical distribution of Aedes species (Diptera: Culicidae): A case study of Enugu State, Nigeria. *Parasite Epidemiology and Control* 2021; **15**.
194. Richman R, Diallo D, Diallo M, et al. Ecological niche modeling of Aedes mosquito vectors of chikungunya virus in southeastern Senegal. *Parasites & Vectors* 2018; **11**(1).
195. Bang YH, Bown DN, Arata AA. Ecological studies on Aedes africanus (Diptera: Culicidae) and associated species in southeastern Nigeria. *J Med Entomol* 1980; **17**(5): 411-6.
196. Bown DN, Bang YH. Ecological studies on Aedes simpsoni (Diptera: Culicidae) in southeastern Nigeria. *J Med Entomol* 1980; **17**(4): 367-74.
197. Hinne IA, Attah SK, Forson AO, Afrane YA. Ecology of anopheles mosquito larvae in different ecological zones in Ghana. *American Journal of Tropical Medicine and Hygiene* 2020; **103**(5 SUPPL): 166.
198. Robert V, Awono-Ambene HP, Thioulouse J. Ecology of larval mosquitoes, with special reference to Anopheles arabiensis (Diptera: Culcidae) in market-garden wells in urban Dakar, Senegal. *J Med Entomol* 1998; **35**(6): 948-55.
199. Favia G, Bimbilé Somda NS, Poda BS, et al. Ecology of reproduction of Anopheles arabiensis in an urban area of Bobo-Dioulasso, Burkina Faso (West Africa): Monthly swarming and mating frequency and their relation to environmental factors. *Plos One* 2018; **13**(11).
200. Labbo R, Fandeur T, Jeanne I, et al. Ecology of urban malaria vectors in Niamey, Republic of Niger. *Malar J* 2016; **15**(1).
201. C. Ojukwu K, Chabi J, Frempong K, et al. Ecology, Distribution and Risk of Transmission of Viral Haemorrhagic Fevers by <i>Aedes</i> Mosquitoes around the Port Areas of Tema in Southern Ghana. *Advances in Entomology* 2022; **10**(02): 135-48.
202. Paul R, Zahouli JBZ, Koudou BG, et al. Effect of land-use changes on the abundance, distribution, and host-seeking behavior of Aedes arbovirus vectors in oil palm-dominated landscapes, southeastern Côte d'Ivoire. *Plos One* 2017; **12**(12).
203. Gomes FM, Hixson BL, Tyner MDW, et al. Effect of naturally occurring Wolbachia in Anopheles gambiae s.l. mosquitoes from Mali on Plasmodium falciparum malaria transmission. *Proceedings of the National Academy of Sciences* 2017; **114**(47): 12566-71.
204. Bøgh C, Clarke SE, Pinder M, Sanyang F, Lindsay SW. Effect of Passive Zooprophylaxis on Malaria Transmission in the Gambia. *Journal of Medical Entomology* 2001; **38**(6): 822-8.
205. Ojianwuna CC, Enwemiwe VN, Erhunmwun SE. Effect of Petroleum Products on the Larvicidal Activity of Aedes Mosquitoes in Ika North-East LGA, Delta State, Nigeria. *The Open Environmental Research Journal* 2021; **14**(1): 24-30.
206. Tchicaya ES, Koudou BG, Keiser J, et al. Effect of Repeated Application of Microbial Larvicides on Malaria Transmission in Central Côte d'Ivoire. *Journal of the American Mosquito Control Association* 2009; **25**(3): 382-5.
207. Kirby MJ, Ameh D, Bottomley C, et al. Effect of two different house screening interventions on exposure to malaria vectors and on anaemia in children in The Gambia: a randomised controlled trial. *The Lancet* 2009; **374**(9694): 998-1009.
208. Diédhiou SM, Konaté L, Doucouré S, et al. Effectiveness of three biological larvicides and of an insect growth regulator against Anopheles arabiensis in Senegal. *Bulletin de la Societe de Pathologie Exotique* 2017; **110**(2): 102-15.
209. Sawadogo SP, Diabaté A, Toé HK, et al. Effects of Age and Size on *Anopheles gambiae* s.s. Male Mosquito Mating Success. *Journal of Medical Entomology* 2013; **50**(2): 285-93.

210. Magbity EB, Marbiah NT, Maude G, et al. Effects of community-wide use of lambdacyhalothrin-impregnated bednets on malaria vectors in rural Sierra Leone. *Med Vet Entomol* 1997; **11**(1): 79-86.
211. Koudou BG, Doumbia M, Janmohamed N, et al. Effects of seasonality and irrigation on malaria transmission in two villages in Côte d'Ivoire. *Annals of Tropical Medicine & Parasitology* 2013; **104**(2): 109-21.
212. Ndiath MO, Cailleau A, Diedhiou SM, et al. Effects of the kdr resistance mutation on the susceptibility of wild Anopheles gambiae populations to Plasmodium falciparum: A hindrance for vector control. *Malar J* 2014; **13**(1).
213. Agossa FR, Padonou GG, Koukpo CZ, et al. Efficacy of a novel mode of action of an indoor residual spraying product, SumiShield® 50WG against susceptible and resistant populations of Anopheles gambiae (s.l.) in Benin, West Africa. *Parasites & Vectors* 2018; **11**(1).
214. Mosqueira B, Chabi J, Chandre F, et al. Efficacy of an insecticide paint against malaria vectors and nuisance in West Africa - Part 2: Field evaluation. *Malar J* 2010; **9**(1).
215. Nicolas L, Darriet F, Hougard JM. Efficacy of Bacillus sphaericus 2362 against larvae of Anopheles gambiae under laboratory and field conditions in West Africa. *Med Vet Entomol* 1987; **1**(2): 157-62.
216. Winkler MS, Tchicaya E, Koudou BG, et al. Efficacy of ICON® Maxx in the laboratory and against insecticide-resistant Anopheles gambiae in central Côte d'Ivoire. *Malar J* 2012; **11**(1).
217. Gianotti RL, Bomblies A, Dafalla M, Issa-Arzika I, Duchemin J-B, Eltahir EAB. Efficacy of local neem extracts for sustainable malaria vector control in an African village. *Malar J* 2008; **7**(1).
218. Koffi AA, Ahoua Alou LP, Djenontin A, et al. Efficacy of Olyset®Duo, a permethrin and pyriproxyfen mixture net against wild pyrethroid-resistantAnopheles gambiae s.s.from Côte d'Ivoire: an experimental hut trial. *Parasite* 2015; **22**.
219. Ngufor C, N'Guessan R, Fagbohoun J, et al. Efficacy of the Olyset Duo net against insecticide-resistant mosquito vectors of malaria. *Sci Transl Med* 2016; **8**(356): 356ra121.
220. Czeher C, Labbo R, Djibrilla A, Here L, Arzika I, Duchemin JB. ELISA study of oocyst-sporozoite transition in malaria vectors. *Parasite* 2006; **13**(3): 257-61.
221. Wilson AL, Pinder M, Bradley J, et al. Emergence of knock-down resistance in the Anopheles gambiae complex in the Upper River Region, The Gambia, and its relationship with malaria infection in children. *Malar J* 2018; **17**(1).
222. Ndiath MO, Cailleau A, Orlandi-Pradines E, et al. Emerging knock-down resistance in Anopheles arabiensis populations of Dakar, Senegal: first evidence of a high prevalence of kdr-e mutation in West African urban area. *Malar J* 2015; **14**(1).
223. Pålsson K, Jaenson TGT, Dias F, Laugen AT, Björkman A. EndophilicAnophelesMosquitoes in Guinea Bissau, West Africa, in Relation to Human Housing Conditions. *Journal of Medical Entomology* 2004; **41**(4): 746-52.
224. Padonou GG, Ossè R, Salako AS, et al. Entomological assessment of the risk of dengue outbreak in Abomey-Calavi Commune, Benin. *Tropical Medicine and Health* 2020; **48**(1).
225. Amoa-Bosompem M, Kobayashi D, Murota K, et al. Entomological Assessment of the Status and Risk of Mosquito-borne Arboviral Transmission in Ghana. *Viruses* 2020; **12**(2).
226. Agwu EJ, Igbinosa IB, Isaac C. Entomological assessment of yellow fever-epidemic risk indices in Benue State, Nigeria, 2010–2011. *Acta Tropica* 2016; **161**: 18-25.
227. Niang A, Sawadogo SP, Millogo AA, et al. Entomological baseline data collection and power analyses in preparation of a mosquito swarm-killing intervention in south-western Burkina Faso. *Malar*

J 2021; **20**(1).
228. Tokponnon TF, Ossè R, Padonou GG, et al. Entomological Characteristics of Malaria Transmission across Benin: An Essential Element for Improved Deployment of Vector Control Interventions. *Insects* 2023; **14**(1).
229. Dahoui MMC, Adou KA, Coulibaly B, et al. Entomological drivers of uneven malaria transmission in urban lowland areas in Bouaké, Côte d'Ivoire. *Malar J* 2023; **22**(1).
230. Thomson MC, Adiamah JH, Connor SJ, et al. Entomological evaluation of the Gambia's National Impregnated Bednet Programme. *Annals of Tropical Medicine & Parasitology* 2016; **89**(3): 229-41.
231. Kone AB, Konan YL, Coulibaly ZI, et al. [Entomological evaluation of the risk of urban outbreak of yellow fever in 2008 in Abidjan, Côte d'Ivoire]. *Med Sante Trop* 2013; **23**(1): 66-71.
232. Wolie RZ, Koffi AA, Ayuk-Taylor L, et al. Entomological indicators of malaria transmission prior to a cluster-randomized controlled trial of a 'lethal house lure' intervention in central Côte d'Ivoire. *Malar J* 2022; **21**(1).
233. Noutcha MA, Anumdu CI. Entomological indices of Anopheles gambiae sensu lato at a rural community in south-west Nigeria. *J Vector Borne Dis* 2009; **46**(1): 43-51.
234. Konan YL, Koné AB, Ekra KD, Doannio JM, Odéhouri KP. [Entomological investigation following the re-emergence of yellow fever in 2008 in Abidjan area (Côte d'Ivoire)]. *Parasite* 2009; **16**(2): 149-52.
235. Konan YL, Fofana D, Coulibaly ZI, et al. [Entomological investigations conducted around ten cases of yellow fever in 2009 in the Denguélé sanitary region, Côte-d'Ivoire]. *Bull Soc Pathol Exot* 2011; **104**(4): 296-302.
236. Kouassi BL, Edi C, Ouattara AF, et al. Entomological monitoring data driving decision-making for appropriate and sustainable malaria vector control in Côte d'Ivoire. *Malar J* 2023; **22**(1).
237. Kudom AA. Entomological surveillance to assess potential outbreak of Aedes-borne arboviruses and insecticide resistance status of Aedes aegypti from Cape Coast, Ghana. *Acta Tropica* 2020; **202**.
238. Carnevale P, Toto JC, Guibert P, Keita M, Manguin S. Entomological survey and report of a knockdown resistance mutation in the malaria vector Anopheles gambiae from the Republic of Guinea. *Transactions of the Royal Society of Tropical Medicine and Hygiene* 2010; **104**(7): 484-9.
239. Ngom EHM, Virgillito C, Manica M, et al. Entomological Survey Confirms Changes in Mosquito Composition and Abundance in Senegal and Reveals Discrepancies among Results by Different Host-Seeking Female Traps. *Insects* 2021; **12**(8).
240. Weiser J, Prasertphon S. Entomopathogenic spore-formers from soil samples of mosquito habitats in Northern Nigeria. *Zentralblatt für Mikrobiologie* 1984; **139**(1): 49-55.
241. Miller BR, Monath TP, Tabachnick WJ, Ezike VI. Epidemic yellow fever caused by an incompetent mosquito vector. *Trop Med Parasitol* 1989; **40**(4): 396-9.
242. Decock KM, Nasidi A, Enriquez J, et al. Epidemic yellow fever in eastern Nigeria, 1986. *Lancet* 1988; **1**(8586): 630-3.
243. Diakité NR, Adja AM, von Stamm T, Utzinger J, N'Goran EK. [Epidemiological baseline situation before the construction of a small dam in five villages of Bouaké, central Côte-d'Ivoire]. *Bull Soc Pathol Exot* 2010; **103**(1): 22-8.
244. Adebote DA, Kogi E, Oniye SJ, Akoje F. Epidemiological significance of the breeding of mosquitoes in discarded automobile tyres in Zaria, Northern Nigeria. *J Commun Dis* 2011; **43**(3): 183-92.
245. Ba O, Sow A, Dahdi S. Epidemiology of Malaria in the city of Kaedi-Mauritania. *Tunis Med* 2018;

96(1): 54-8.
246. Vercruysse J, Jancloes M, Vandevelden L. Epidemiology of seasonal falciparum malaria in an urban area of Senegal. *Bull World Health Organ* 1983; **61**(5): 821-31.
247. Anosike JC, Nwoke BE, Okere AN, et al. Epidemiology of tree-hole breeding mosquitoes in the tropical rainforest of Imo State, south-east Nigeria. *Ann Agric Environ Med* 2007; **14**(1): 31-8.
248. Gnémé A, Guelbéogo WM, Riehle MM, et al. Equivalent susceptibility of Anopheles gambiae M and S molecular forms and Anopheles arabiensis to Plasmodium falciparum infection in Burkina Faso. *Malar J* 2013; **12**: 204.
249. Mugenzi LMJ, Akosah-Brempong G, Tchouakui M, et al. Escalating pyrethroid resistance in two major malaria vectors Anopheles funestus and Anopheles gambiae (s.l.) in Atatam, Southern Ghana. *BMC Infectious Diseases* 2022; **22**(1).
250. Entonu ME, Muhammad A, Ndams IS, Franciosa G. Evaluation of Actin-1 Expression in Wild Caught Wuchereria bancrofti-Infected Mosquito Vectors. *Journal of Pathogens* 2020; **2020**: 1-9.
251. Oumbouke WA, Rowland M, Koffi AA, Alou LPA, Camara S, N'Guessan R. Evaluation of an alpha-cypermethrin + PBO mixture long-lasting insecticidal net VEERALIN® LN against pyrethroid resistant Anopheles gambiae s.s.: an experimental hut trial in M'bé, central Côte d'Ivoire. *Parasites & Vectors* 2019; **12**(1).
252. Faye O, Diallo S, Gaye O, Faye O, Mouchet J. Evaluation of fenitrothion (Sumithion® PM 40) efficacy on vector density and malaria prevalence of Pout (Thies, Senegal). *ANN SOC BELG MED TROP* 1992; **72**(2): 103-12.
253. Sanou A, Moussa Guelbéogo W, Nelli L, et al. Evaluation of mosquito electrocuting traps as a safe alternative to the human landing catch for measuring human exposure to malaria vectors in Burkina Faso. *Malar J* 2019; **18**(1).
254. Huijben S, Oumbouke WA, Koffi AA, Alou LPA, Rowland M, N'Guessan R. Evaluation of standard pyrethroid based LNs (MiraNet and MagNet) in experimental huts against pyrethroid resistant Anopheles gambiae s.l. M'bé, Côte d'Ivoire: Potential for impact on vectorial capacity. *Plos One* 2019; **14**(4).
255. N'Guessan R, Rowland M, Moumouni T-L, Kesse NB, Carnevale P. Evaluation of synthetic repellents on mosquito nets in experimental huts against insecticide-resistant Anopheles gambiae and Culex quinquefasciatus mosquitoes. *Transactions of the Royal Society of Tropical Medicine and Hygiene* 2006; **100**(12): 1091-7.
256. Ossè R, Aikpon R, Padonou GG, Oussou O, Yadouléton A, Akogbéto M. Evaluation of the efficacy of bendiocarb in indoor residual spraying against pyrethroid resistant malaria vectors in Benin: results of the third campaign. *Parasit Vectors* 2012; **5**: 163.
257. Gueye M, Dia I, Diedhiou S, et al. Evaluation of the Efficacy of Fludora® Fusion WP-SB 56.25 (Mixture of Clothianidin and Deltamethrin) against Anopheles coluzzii Laboratory and An. arabiensis Wild Colonies. *Tropical Medicine and Infectious Disease* 2022; **7**(10).
258. Diallo D, Ba Y, Dia I, Sall AA, Diallo M. Evaluation of the efficiency of bird-baited traps for sampling potential West Nile Fever mosquito vectors (Diptera: Culicidae) in Senegal. *Parasite* 2010; **17**(2): 143-7.
259. Diallo D, Ndiaye EH, Fall G, et al. Evaluation of the Performance of Different Traps for Sampling Usutu and West Nile Viruses and Mosquito (Diptera: Culicidae) Vectors in Senegal. *Journal of Medical Entomology* 2019; **56**(1): 149-55.
260. Matthews SD, Meehan LJ, Onyabe DY, et al. Evidence for late Pleistocene population expansion

of the malarial mosquitoes, Anopheles arabiensis and Anopheles gambiae in Nigeria. *Med Vet Entomol* 2007; **21**(4): 358-69.
261. Jeffries CL, Cansado-Utrilla C, Beavogui AH, et al. Evidence for natural hybridization and novel Wolbachia strain superinfections in the Anopheles gambiae complex from Guinea. *Royal Society Open Science* 2021; **8**(4).
262. Govoetchan R, Gnanguenon V, Azondékon R, et al. Evidence for perennial malaria in rural and urban areas under the Sudanian climate of Kandi, Northeastern Benin. *Parasites and Vectors* 2014; **7**(1).
263. Oduola AO, Idowu ET, Oyebola MK, et al. Evidence of carbamate resistance in urban populations of Anopheles gambiae s.s. mosquitoes resistant to DDT and deltamethrin insecticides in Lagos, South-Western Nigeria. *Parasit Vectors* 2012; **5**: 116.
264. Orsborne J, Mohammed AR, Jeffries CL, et al. Evidence of extrinsic factors dominating intrinsic blood host preferences of major African malaria vectors. *Scientific Reports* 2020; **10**(1).
265. Aïzoun N, Aïkpon R, Akogbéto M. Evidence of increasing L1014F kdr mutation frequency in Anopheles gambiae s.l. pyrethroid resistant following a nationwide distribution of LLINs by the Beninese National Malaria Control Programme. *Asian Pacific Journal of Tropical Biomedicine* 2014; **4**(3): 239-43.
266. Mouhamadou CS, de Souza SS, Fodjo BK, Zoh MG, Bli NK, Koudou BG. Evidence of insecticide resistance selection in wild Anopheles coluzzii mosquitoes due to agricultural pesticide use. *Infectious Diseases of Poverty* 2019; **8**(1).
267. Aïkpon R, Sèzonlin M, Ossè R, Akogbéto M. Evidence of multiple mechanisms providing carbamate and organophosphate resistance in field An. gambiae population from Atacora in Benin. *Parasit Vectors* 2014; **7**: 568.
268. Awolola TS, Oduola OA, Strode C, Koekemoer LL, Brooke B, Ranson H. Evidence of multiple pyrethroid resistance mechanisms in the malaria vector Anopheles gambiae sensu stricto from Nigeria. *Transactions of the Royal Society of Tropical Medicine and Hygiene* 2009; **103**(11): 1139-45.
269. Baldini F, Segata N, Pompon J, et al. Evidence of natural Wolbachia infections in field populations of Anopheles gambiae. *Nature Communications* 2014; **5**(1).
270. Hien AS, Soma DD, Maiga S, et al. Evidence supporting deployment of next generation insecticide treated nets in Burkina Faso: bioassays with either chlorfenapyr or piperonyl butoxide increase mortality of pyrethroid-resistant Anopheles gambiae. *Malar J* 2021; **20**(1).
271. Carvalho LH, Hien AS, Soma DD, et al. Evidence that agricultural use of pesticides selects pyrethroid resistance within Anopheles gambiae s.l. populations from cotton growing areas in Burkina Faso, West Africa. *Plos One* 2017; **12**(3).
272. Djouaka R, Zeukeng F, Daiga Bigoga J, et al. Evidences of the Low Implication of Mosquitoes in the Transmission ofMycobacterium ulcerans, the Causative Pathogen of Buruli Ulcer. *Canadian Journal of Infectious Diseases and Medical Microbiology* 2017; **2017**: 1-12.
273. Diallo M, Kolley ESM, Dia AK, et al. Evolution of the Ace-1 and Gste2 Mutations and Their Potential Impact on the Use of Carbamate and Organophosphates in IRS for Controlling Anopheles gambiae s.l., the Major Malaria Mosquito in Senegal. *Pathogens* 2022; **11**(9).
274. Junglen S, Kurth A, Kuehl H, et al. Examining Landscape Factors Influencing Relative Distribution of Mosquito Genera and Frequency of Virus Infection. *EcoHealth* 2009; **6**(2): 239-49.
275. Guindo-Coulibaly N, Adja AM, Coulibaly JT, Kpan MDS, Adou KA, Zoh DD. Expansion of Aedes africanus (Diptera: Culicidae), a sylvatic vector of arboviruses, into an urban environment of Abidjan, Côte d'Ivoire. *J Vector Ecol* 2019; **44**(2): 248-55.

276. Skovmand O, Sanogo E. Experimental formulations of Bacillus sphaericus and B. thuringiensis israelensis against Culex quinquefasciatus and Anopheles gambiae (Diptera: Culicidae) in Burkina Faso. *J Med Entomol* 1999; **36**(1): 62-7.
277. Asidi AN, N'Guessan R, Hutchinson RA, Traoré-Lamizana M, Carnevale P, Curtis CF. Experimental hut comparisons of nets trated with carbamate or pyrethroid insecticides, washed or unwashed, against pyrethroid-resistant mosquitoes. *Medical and Veterinary Entomology* 2004; **18**(2): 134-40.
278. Asidi AN, Guessan RN, Koffi AA, et al. Experimental hut evaluation of bednets treated with an organophosphate (chlorpyrifos-methyl) or a pyrethroid (lambdacyhalothrin) alone and in combination against insecticide-resistant Anopheles gambiae and Culex quinquefasciatus mosquitoes. *Malar J* 2005; **4**.
279. Huijben S, Bayili K, N'Do S, et al. Experimental hut evaluation of DawaPlus 3.0 LN and DawaPlus 4.0 LN treated with deltamethrin and PBO against free-flying populations of Anopheles gambiae s.l. in Vallée du Kou, Burkina Faso. *Plos One* 2019; **14**(12).
280. Badolo A, Guelbeogo WM, Tiono AB, Traore A, Sagnon NF, Sirima SB. Experimental hut evaluation of Fendona 6SC (R)-treated bednets and Interceptor (R) long-lasting nets against Anopheles gambiae s.l. in Burkina Faso. *J Vector Borne Dis* 2012; **49**(4): 234-41.
281. Miller JE, Lindsay SW, Armstrong JR. Experimental hut trials of bednets impregnated with synthetic pyrethroid or organophosphate insecticide for mosquito control in The Gambia. *Med Vet Entomol* 1991; **5**(4): 465-76.
282. Akoton R, Tchigossou GM, Djègbè I, et al. Experimental huts trial of the efficacy of pyrethroids/piperonyl butoxide (PBO) net treatments for controlling multi-resistant populations of Anopheles funestus s.s. in Kpomè, Southern Benin. *Wellcome Open Research* 2018; **3**.
283. Hawkes FM, Dabiré RK, Sawadogo SP, Torr SJ, Gibson G. Exploiting Anopheles responses to thermal, odour and visual stimuli to improve surveillance and control of malaria. *Scientific Reports* 2017; **7**(1).
284. Ibrahim SS, Mukhtar MM, Irving H, et al. Exploring the Mechanisms of Multiple Insecticide Resistance in a Highly Plasmodium-Infected Malaria Vector Anopheles funestus Sensu Stricto from Sahel of Northern Nigeria. *Genes* 2020; **11**(4).
285. Lindsay SW, Schellenberg JRMA, Zeiler HA, Daly RJ, Salum FM, Wilkins HA. Exposure of Gambian children to Anopheles gambiae malaria vectors in an irrigated rice production area. *Medical and Veterinary Entomology* 1995; **9**(1): 50-8.
286. Chevalier V, Mondet B, Diaite A, Lancelot R, Fall AG, Ponçon N. Exposure of sheep to mosquito bites: possible consequences for the transmission risk of Rift Valley Fever in Senegal. *Med Vet Entomol* 2004; **18**(3): 247-55.
287. Djouaka RF, Bakare AA, Coulibaly ON, et al. Expression of the cytochrome P450s, CYP6P3 and CYP6M2 are significantly elevated in multiple pyrethroid resistant populations of Anopheles gambiae s.s. from Southern Benin and Nigeria. *BMC Genomics* 2008; **9**(1).
288. Dia I, Lochouarn L, Diatta M, Sokhna CS, Fontenille D. [Feeding behaviour of Anopheles funestus resting females in Senegal]. *Bull Soc Pathol Exot* 2001; **94**(2 Pt 2): 210-3.
289. Fall AG, Diaïté A, Lancelot R, et al. Feeding behaviour of potential vectors of West Nile virus in Senegal. *Parasit Vectors* 2011; **4**: 99.
290. Charlwood JD, TomÁS EV, Egyir-Yawson A, Kampango AA, Pitts RJ. Feeding frequency and survival of Anopheles gambiae in a rice-growing area in Ghana. *Medical and Veterinary Entomology*

2012; **26**(3): 263-70.
291. Bilgo E, Vantaux A, Sanon A, et al. Field assessment of potential sugar feeding stations for disseminating bacteria in a paratransgenic approach to control malaria. *Malar J* 2018; **17**(1).
292. Corbel V, Chabi J, Dabiré RK, et al. Field efficacy of a new mosaic long-lasting mosquito net (PermaNet® 3.0) against pyrethroid-resistant malaria vectors: a multi centre study in Western and Central Africa. *Malar J* 2010; **9**(1).
293. Obeta JAN. Field evaluation of Bacillus sphaericus strain 1593 as a mosquito biocide. *J Invertebr Pathol* 1986; **48**(2): 133-8.
294. Cantacessi C, Minetti C, Pilotte N, et al. Field evaluation of DNA detection of human filarial and malaria parasites using mosquito excreta/feces. *PLOS Neglected Tropical Diseases* 2020; **14**(4).
295. Costantini C, Badolo A, Ilboudo-Sanogo E. Field evaluation of the efficacy and persistence of insect repellents DEET, IR3535, and KBR 3023 against Anopheles gambiae complex and other Afrotropical vector mosquitoes. *Transactions of the Royal Society of Tropical Medicine and Hygiene* 2004; **98**(11): 644-52.
296. Kuhlow F. Field experiments on the behavior of malaria vectors in an unsprayed hut and in a hut sprayed with DDT in Northern Nigeria. *Bull World Health Organ* 1962; **26**(1): 93-102.
297. Darriet F. Field trial of the efficacy of 3 pyrethroids in the control of malaria vectors. *Parassitologia* 1991; **33**(2-3): 111-9.
298. Oumbouke WA, Pignatelli P, Barreaux AMG, et al. Fine scale spatial investigation of multiple insecticide resistance and underlying target-site and metabolic mechanisms in Anopheles gambiae in central Côte d'Ivoire. *Scientific Reports* 2020; **10**(1).
299. Poinsignon A, Samb B, Doucoure S, et al. First attempt to validate the gSG6-P1 salivary peptide as an immuno-epidemiological tool for evaluating human exposure to Anopheles funestus bites. *Tropical Medicine & International Health* 2010; **15**(10): 1198-203.
300. Wu JT, Badolo A, Sombié A, et al. First comprehensive analysis of Aedes aegypti bionomics during an arbovirus outbreak in west Africa: Dengue in Ouagadougou, Burkina Faso, 2016–2017. *PLOS Neglected Tropical Diseases* 2022; **16**(7).
301. Edi AVC, N'Dri BP, Chouaibou M, et al. First detection of N1575Y mutation in pyrethroid resistant Anopheles gambiae in Southern Côte d'Ivoire. *Wellcome Open Research* 2017; **2**.
302. Yadouleton A, Hounkanrin G, Tchibozo C, et al. First Detection of the Invasive Mosquito Vector Aedes albopictus (Diptera: Culicidae) in Benin, West Africa, 2021. *Journal of Medical Entomology* 2022; **59**(3): 1090-4.
303. Fournet F, Adja AM, Adou KA, et al. First detection of the malaria vector Anopheles arabiensis in Côte d'Ivoire: urbanization in question. *Malar J* 2022; **21**(1).
304. Fall G, Diallo D, Soumaila H, et al. First Detection of the West Nile Virus Koutango Lineage in Sandflies in Niger. *Pathogens* 2021; **10**(3).
305. Fontenille D, Diallo M, Mondo M, Ndiaye M, Thonnon J. First evidence of natural vertical transmission of yellow fever virus in Aedes aegypti, its epidemic vector. *Trans R Soc Trop Med Hyg* 1997; **91**(5): 533-5.
306. Diallo M, Lochouarn L, Ba K, et al. First isolation of the Rift Valley fever virus from Culex poicilipes (Diptera: Culicidae) in nature. *Am J Trop Med Hyg* 2000; **62**(6): 702-4.
307. Le Port A, Cottrell G, Martin-Prevel Y, Migot-Nabias F, Cot M, Garcia A. First malaria infections in a cohort of infants in Benin: biological, environmental and genetic determinants. Description of the study site, population methods and preliminary results. *BMJ Open* 2012; **2**(2).

308. Fagbohun IK, Idowu ET, Otubanjo OA, Awolola TS. First report of AChE1 (G119S) mutation and multiple resistance mechanisms in Anopheles gambiae s.s. in Nigeria. *Scientific Reports* 2020; **10**(1).
309. Gordicho V, Vicente JL, Sousa CA, et al. First report of an exophilic Anopheles arabiensis population in Bissau City, Guinea-Bissau: recent introduction or sampling bias? *Malar J* 2014; **13**: 423.
310. Oyeniyi AT, Adeogun AO, Idowu ET, et al. First report of N1575Y mutation in pyrethroid resistant Anopheles gambiae s.l. in Nigeria. *Scientific African* 2020; **10**.
311. Niang EHA, Bassene H, Makoundou P, Fenollar F, Weill M, Mediannikov O. First report of natural Wolbachia infection in wild Anopheles funestus population in Senegal. *Malar J* 2018; **17**(1).
312. Chouaïbou M, Kouadio FB, Tia E, Djogbenou L. First report of the East African kdr mutation in an Anopheles gambiae mosquito in Côte d'Ivoire. *Wellcome Open Research* 2017; **2**.
313. da Cruz DL, Paiva MHS, Guedes DRD, et al. First report of the L1014F kdr mutation in wild populations of Anopheles arabiensis in Cabo Verde, West Africa. *Parasites & Vectors* 2021; **14**(1).
314. Namountougou M, Diabaté A, Etang J, et al. First report of the L1014S kdr mutation in wild populations of Anopheles gambiae M and S molecular forms in Burkina Faso (West Africa). *Acta Tropica* 2013; **125**(2): 123-7.
315. Djègbè I, Akoton R, Tchigossou GM, et al. First report of the presence of L1014S Knockdown-resistance mutation in Anopheles gambiae s.s and Anopheles coluzzii from Togo, West Africa. *Wellcome Open Research* 2018; **3**.
316. Harris E, Sylla M, Bosio C, Urdaneta-Marquez L, Ndiaye M, Black WC. Gene Flow, Subspecies Composition, and Dengue Virus-2 Susceptibility among Aedes aegypti Collections in Senegal. *PLoS Neglected Tropical Diseases* 2009; **3**(4).
317. Kallies R, Kopp A, Zirkel F, et al. Genetic Characterization of Goutanap Virus, a Novel Virus Related to Negeviruses, Cileviruses and Higreviruses. *Viruses* 2014; **6**(11): 4346-57.
318. Iyiola OA. Genetic Diversity and Molecular Characterization of Mosquitoes (Diptera: Culicidae) In North-Central Nigeria Using Ribosomal DNA ITS2 and Mitochondrial 16S-DNA Sequences. *The Iraqi Journal of Veterinary Medicine* 2020; **44**(2): 78-91.
319. Isa I, Ndams IS, Aminu M, Chechet G, Dotzauer A, Simon AY. Genetic diversity of Dengue virus serotypes circulating among Aedes mosquitoes in selected regions of northeastern Nigeria. *One Health* 2021; **13**.
320. Obembe A, Oduola AO, Oyeniyi TA, Olakiigbe AK, Awolola ST. Genetic identity, human blood indices, and sporozoite rates of malaria vectors in Gaa-Bolorunduro, Kwara State, Nigeria. *The Journal of Infection in Developing Countries* 2022; **16**(08): 1351-8.
321. Arnal A, Kengne P, Brengues C, et al. Genetic polymorphism at an odorant receptor gene (Or39) among mosquitoes of the Anopheles gambiae complex in Senegal (West Africa). *BMC Res Notes* 2014; **7**: 321.
322. Lucas ER, Nagi SC, Egyir-Yawson A, et al. Genome-wide association studies reveal novel loci associated with pyrethroid and organophosphate resistance in Anopheles gambiae s.l. *bioRxiv* 2023.
323. J P, A EY, Jl V, et al. Geographic population structure of theAfrican malaria vectorAnopheles gambiaesuggests a role for the forest-savannah biome transition as a barrier to gene flow. *Evolutionary Applications* 2013; **6**(6): 910-24.
324. Aïkpon R, Sèzonlin M, Tokponon F, et al. Good performances but short lasting efficacy of Actellic 50 EC Indoor Residual Spraying (IRS) on malaria transmission in Benin, West Africa. *Parasit Vectors* 2014; **7**: 256.
325. Fontenille D, Lochouarn L, Diagne N, et al. High annual and seasonal variations in malaria

transmission by anophelines and vector species composition in Dielmo, a holoendemic area in Senegal. *Am J Trop Med Hyg* 1997; **56**(3): 247-53.
326. Kouadio AMN, Guindo-Coulibaly N, Adja AM, et al. High density in dry season and resistance to pyrethroids of Aedes aegypti populations in Bouaké, Côte d'Ivoire. *International Journal of Tropical Insect Science* 2022; **42**(5): 3395-402.
327. Sombié A, Saiki E, Yaméogo F, et al. High frequencies of F1534C and V1016I kdr mutations and association with pyrethroid resistance in Aedes aegypti from Somgandé (Ouagadougou), Burkina Faso. *Tropical Medicine and Health* 2019; **47**(1).
328. Ibrahim SS, Manu YA, Tukur Z, Irving H, Wondji CS. High frequency of kdr L1014F is associated with pyrethroid resistance in Anopheles coluzzii in Sudan savannah of northern Nigeria. *BMC Infect Dis* 2014; **14**: 441.
329. Pwalia R, Joannides J, Iddrisu A, et al. High insecticide resistance intensity of Anopheles gambiae (s.l.) and low efficacy of pyrethroid LLINs in Accra, Ghana. *Parasites & Vectors* 2019; **12**(1).
330. Ahadji-Dabla KM, Amoudji AD, Nyamador SW, et al. High Levels of Knockdown Resistance in Anopheles coluzzii and Anopheles gambiae (Diptera: Culicidae), Major Malaria Vectors in Togo, West Africa: A 2011 Monitoring Report. *Journal of Medical Entomology* 2019; **56**(4): 1159-64.
331. Ibrahim SS, Mukhtar MM, Irving H, et al. High Plasmodium infection and multiple insecticide resistance in a major malaria vector Anopheles coluzzii from Sahel of Niger Republic. *Malar J* 2019; **18**(1).
332. Lenhart A, Omotayo AI, Dogara MM, et al. High pyrethroid-resistance intensity in Culex quinquefasciatus (Say) (Diptera: Culicidae) populations from Jigawa, North-West, Nigeria. *PLOS Neglected Tropical Diseases* 2022; **16**(6).
333. Adja A, Assouho K, Assi SB, et al. High vectorial transmission of malaria in urban and rural settings in the northern, western and eastern regions of Côte d'Ivoire. *Journal of Vector Borne Diseases* 2022; **59**(3): 275-84.
334. Machault V, Gadiaga L, Vignolles C, et al. Highly focused anopheline breeding sites and malaria transmission in Dakar. *Malar J* 2009; **8**(1).
335. Samy AM, Biteye B, Fall AG, et al. Host-feeding patterns of Aedes (Aedimorphus) vexans arabiensis, a Rift Valley Fever virus vector in the Ferlo pastoral ecosystem of Senegal. *Plos One* 2019; **14**(10).
336. Magbity EB, Lines JD, Marbiah MT, David K, Peterson E. How reliable are light traps in estimating biting rates of adult Anopheles gambiae s.l. (Diptera: Culicidae) in the presence of treated bed nets? *Bulletin of Entomological Research* 2007; **92**(1): 71-6.
337. Djènontin A, Bouraima A, Soares C, Egbinola S, Cottrell G. Human biting rhythm of Anopheles gambiae Giles, 1902 (Diptera: Culicidae) and sleeping behaviour of pregnant women in a lagoon area in Southern Benin. *BMC Research Notes* 2021; **14**(1).
338. Aboagye-Antwi F, Guindo A, Traoré AS, et al. Hydric stress-dependent effects of Plasmodium falciparum infection on the survival of wild-caught Anopheles gambiae female mosquitoes. *Malar J* 2010; **9**: 243.
339. Parry R, Naccache F, Ndiaye EH, et al. Identification and RNAi Profile of a Novel Iflavirus Infecting Senegalese Aedes vexans arabiensis Mosquitoes. *Viruses* 2020; **12**(4).
340. Kent RJ, Norris DE. Identification of mammalian blood meals in mosquitoes by a multiplexed polymerase chain reaction targeting cytochrome B. *Am J Trop Med Hyg* 2005; **73**(2): 336-42.
341. Awolola TS, Oyewole IO, Koekemoer LL, Coetzee M. Identification of three members of the

Anopheles funestus (Diptera: Culicidae) group and their role in malaria transmission in two ecological zones in Nigeria. *Transactions of the Royal Society of Tropical Medicine and Hygiene* 2005; **99**(7): 525-31.
342. Horton AA, Lee Y, Coulibaly CA, et al. Identification of three single nucleotide polymorphisms in Anopheles gambiae immune signaling genes that are associated with natural Plasmodium falciparum infection. *Malar J* 2010; **9**: 160.
343. Fillinger U, Sombroek H, Majambere S, van Loon E, Takken W, Lindsay SW. Identifying the most productive breeding sites for malaria mosquitoes in The Gambia. *Malar J* 2009; **8**(1).
344. Drame PM, Machault V, Diallo A, et al. IgG responses to the gSG6-P1 salivary peptide for evaluating human exposure to Anopheles bites in urban areas of Dakar region, Senegal. *Malar J* 2012; **11**.
345. Jatta E, Carrasco-Tenezaca M, Jawara M, et al. Impact of increased ventilation on indoor temperature and malaria mosquito density: an experimental study in The Gambia. *Journal of The Royal Society Interface* 2021; **18**(178).
346. Sovi A, Govoétchan R, Tokponnon F, et al. Impact of land-use on malaria transmission in the Plateau region, southeastern Benin. *Parasites and Vectors* 2013; **6**(1).
347. Sovi A, Azondékon R, Aïkpon RY, et al. Impact of operational effectiveness of long-lasting insecticidal nets (LLINs) on malaria transmission in pyrethroid-resistant areas. *Parasit Vectors* 2013; **6**(1): 319.
348. Awolola ST, Adeogun AO, Olojede JB, Oduola AO, Oyewole IO, Amajoh CN. Impact of PermaNet 3.0 on entomological indices in an area of pyrethroid resistant Anopheles gambiae in south-western Nigeria. *Parasit Vectors* 2014; **7**: 236.
349. Ouédraogo WM, Toé KH, Sombié A, et al. Impact of physicochemical parameters of Aedes aegypti breeding habitats on mosquito productivity and the size of emerged adult mosquitoes in Ouagadougou City, Burkina Faso. *Parasites & Vectors* 2022; **15**(1).
350. Skovmand O, Ouedraogo TDA, Sanogo E, Samuelsen H, Toé LP, Baldet T. Impact of Slow-ReleaseBacillus sphaericusGranules on Mosquito Populations Followed in a Tropical Urban Environment. *Journal of Medical Entomology* 2009; **46**(1): 67-76.
351. Padonou GG, Sezonlin M, Ossé R, et al. Impact of three years of large scale Indoor Residual Spraying (IRS) and Insecticide Treated Nets (ITNs) interventions on insecticide resistance in Anopheles gambiae s.l. in Benin. *Parasit Vectors* 2012; **5**: 72.
352. Klinkenberg E, McCall PJ, Wilson MD, Amerasinghe FP, Donnelly MJ. Impact of urban agriculture on malaria vectors in Accra, Ghana. *Malar J* 2008; **7**(1).
353. Oyewole IO, Awolola TS. Impact of urbanisation on bionomics and distribution of malaria vectors in Lagos, southwestern Nigeria. *J Vector Borne Dis* 2006; **43**(4): 173-8.
354. Guindo A, Epopa PS, Doumbia S, et al. Improved BioGents® Sentinel trap with heat (BGSH) for outdoor collections of Anopheline species in Burkina Faso and Mali, West Africa. *Parasites & Vectors* 2021; **14**(1).
355. Keïta M, Doumbia S, Sissoko I, et al. Indoor and outdoor malaria transmission in two ecological settings in rural Mali: implications for vector control. *Malar J* 2021; **20**(1).
356. Olayemi IK, Danlami G, Isah B, Odeyemi OM, Ukubuiwe AC, Mustapha OM. Indoor Behaviour Responses of the Principal Malaria Vector, Anopheles gambiae (Diptera: Culicidae), in Relation to Micro-climatic Conditions in Minna, North Central Nigeria. *Research Journal of Parasitology* 2011; **6**(3): 109-15.

357. Oliveira PL, Ngufor C, Fongnikin A, Rowland M, N'Guessan R. Indoor residual spraying with a mixture of clothianidin (a neonicotinoid insecticide) and deltamethrin provides improved control and long residual activity against pyrethroid resistant Anopheles gambiae sl in Southern Benin. *Plos One* 2017; **12**(12).
358. Qualls WA, Müller GC, Traore SF, et al. Indoor use of attractive toxic sugar bait (ATSB) to effectively control malaria vectors in Mali, West Africa. *Malar J* 2015; **14**(1).
359. Furnival-Adams JEC, Camara S, Rowland M, et al. Indoor use of attractive toxic sugar bait in combination with long-lasting insecticidal net against pyrethroid-resistant Anopheles gambiae: an experimental hut trial in Mbé, central Côte d'Ivoire. *Malar J* 2020; **19**(1).
360. Djènontin A, Akogbéto M, Chandre F, et al. Indoor Use of Plastic Sheeting Impregnated with Carbamate Combined with Long-Lasting Insecticidal Mosquito Nets for the Control of Pyrethroid-Resistant Malaria Vectors. *The American Journal of Tropical Medicine and Hygiene* 2010; **83**(2): 266-70.
361. Robert V, Carnevale P. Influence of deltamethrin treatment of bed nets on malaria transmission in the Kou valley, Burkina Faso. *Bull World Health Organ* 1991; **69**(6): 735-40.
362. Ngwu GI, Okafor FC, Eyo JE, Ngwu MI. Influence of geographical location on abundance assortment of Anopheles mosquito species (Diptera: Culicidae) on malaria parasite rate in Enugu State, Nigeria. *Nigerian Journal of Parasitology* 2021; **42**(1): 31-40.
363. Olayemi IK. Influence of land-use on the fitness of anopheles gambiae, the principal vector of malaria in nigeria. *Online J Health Allied Sci* 2008; **7**(4).
364. Opayele AV, Adekunle AJ, Ibrahim KT, Olaleye DO. Influence of Meteorological Variables on Diversity and Abundance of Mosquito Vectors in Two Livestock Farms in Ibadan, Nigeria: Public Health Implications. *Journal of Mosquito Research* 2017.
365. Chouaïbou MS, Fodjo BK, Fokou G, et al. Influence of the agrochemicals used for rice and vegetable cultivation on insecticide resistance in malaria vectors in southern Côte d'Ivoire. *Malar J* 2016; **15**(1).
366. Yaméogo F, Wangrawa DW, Sombié A, Sanon A, Badolo A. Insecticidal activity of essential oils from six aromatic plants against Aedes aegypti, dengue vector from two localities of Ouagadougou, Burkina Faso. *Arthropod-Plant Interactions* 2021; **15**(4): 627-34.
367. Wangrawa DW, Badolo A, Ilboudo Z, et al. Insecticidal Activity of Local Plants Essential Oils Against Laboratory and Field Strains of Anopheles gambiae s. l. (Diptera: Culicidae) From Burkina Faso. *Journal of Economic Entomology* 2018.
368. Ojianwuna CC, Enwemiwe VN. Insecticidal effectiveness of naphthalene and its combination with kerosene against the emergence of Aedes aegypti in Ika North East, LGA, Delta State, Nigeria. *Parasite Epidemiology and Control* 2022; **18**.
369. Sanou A, Nelli L, Guelbéogo WM, et al. Insecticide resistance and behavioural adaptation as a response to long-lasting insecticidal net deployment in malaria vectors in the Cascades region of Burkina Faso. *Scientific Reports* 2021; **11**(1).
370. Taconet P, Soma DD, Zogo B, et al. Insecticide resistance and biting behaviour of malaria vectors in rural West-Africa: a data mining study to adress their fine-scale spatiotemporal heterogeneity, drivers, and predictability. 2022.
371. Magnin M, Marboutin E, Pasteur N. Insecticide resistance in Culex quinquefasciatus (Diptera: Culicidae) in West Africa. *J Med Entomol* 1988; **25**(2): 99-104.
372. Hamid-Adiamoh M, Amambua-Ngwa A, Nwakanma D, D'Alessandro U, Awandare GA, Afrane

YA. Insecticide resistance in indoor and outdoor-resting Anopheles gambiae in Northern Ghana. *Malar J* 2020; **19**(1).
373. Baffour-Awuah S, Annan AA, Maiga-Ascofare O, et al. Insecticide resistance in malaria vectors in Kumasi, Ghana. *Parasites & Vectors* 2016; **9**(1).
374. DjogbÉNou L, Pasteur N, AkogbÉTo M, Weill M, Chandre F. Insecticide resistance in the Anopheles gambiae complex in Benin: a nationwide survey. *Medical and Veterinary Entomology* 2011; **25**(3): 256-67.
375. Armstrong JA, Ramsdale CD, Ramakrishna V. Insecticide Resistance inAnopheles GambiaeGiles in Western Sokoto, Northern Nigeria. *Annals of Tropical Medicine & Parasitology* 2016; **52**(3): 247-56.
376. Apetogbo Y, Ahadji-Dabla KM, Soma DD, et al. Insecticide resistance intensity and efficacy of synergists with pyrethroids in Anopheles gambiae (Diptera: Culicidae) from Southern Togo. *Malar J* 2022; **21**(1).
377. Fuehrer H-P, Badolo A, Sombié A, et al. Insecticide resistance levels and mechanisms in Aedes aegypti populations in and around Ouagadougou, Burkina Faso. *PLOS Neglected Tropical Diseases* 2019; **13**(5).
378. Konan LY, Oumbouke WA, Silué UG, et al. Insecticide Resistance Patterns and Mechanisms in Aedes aegypti (Diptera: Culicidae) Populations Across Abidjan, Côte d'Ivoire Reveal Emergent Pyrethroid Resistance. *Journal of Medical Entomology* 2021; **58**(4): 1808-16.
379. Ononamadu CJ, Datit JT, Imam AA. Insecticide Resistance Profile ofAnopheles gambiaeMosquitoes: A Study of a Residential and Industrial Breeding Sites in Kano Metropolis, Nigeria. *Environmental Health Insights* 2020; **14**.
380. Anto F, Asoala V, Anyorigiya T, et al. Insecticide resistance profiles for malaria vectors in the Kassena-Nankana district of Ghana. *Malar J* 2009; **8**(1).
381. Amoudji AD, Ahadji-Dabla KM, Hien AS, et al. Insecticide resistance profiles of Anopheles gambiae s.l. in Togo and genetic mechanisms involved, during 3-year survey: is there any need for resistance management? *Malar J* 2019; **18**(1).
382. Gueye OK, Tchouakui M, Dia AK, et al. Insecticide Resistance Profiling of Anopheles coluzzii and Anopheles gambiae Populations in the Southern Senegal: Role of Target Sites and Metabolic Resistance Mechanisms. *Genes* 2020; **11**(12).
383. Yadouleton AW, Padonou G, Asidi A, et al. Insecticide resistance status in Anopheles gambiae in southern Benin. *Malar J* 2010; **9**: 83.
384. Yadouléton A, Badirou K, Agbanrin R, et al. Insecticide resistance status in Culex quinquefasciatus in Benin. *Parasites & Vectors* 2015; **8**(1).
385. Salako AS, Ahogni I, Aïkpon R, et al. Insecticide resistance status, frequency of L1014F Kdr and G119S Ace-1 mutations, and expression of detoxification enzymes in Anopheles gambiae (s.l.) in two regions of northern Benin in preparation for indoor residual spraying. *Parasites & Vectors* 2018; **11**(1).
386. Self LS, Pant CP. Insecticide susceptibility and resistance in populations of Anopheles gambiae, Culex fatigans and Aedes aegypti in southern Nigeria. *Bulletin of the World Health Organization* 1966; **34**(6): 960-2.
387. Chabi J, Baidoo PK, Datsomor AK, et al. Insecticide susceptibility of natural populations of Anopheles coluzzii and Anopheles gambiae (sensu stricto) from Okyereko irrigation site, Ghana, West Africa. *Parasites & Vectors* 2016; **9**(1).
388. Okorie PN, Ademowo OG, Irving H, Kelly-Hope LA, Wondji CS. Insecticide susceptibility ofAnopheles coluzziiandAnopheles gambiaemosquitoes in Ibadan, Southwest Nigeria. *Medical and*

Veterinary Entomology 2015; **29**(1): 44-50.
389. Olayemi IK, Ande AT, Chita S, Ibemesi G, Ayanwale VA, Odeyemi OM. Insecticide susceptibility profile of the principal malaria vector, Anopheles gambiae s.l. (Diptera: Culicidae), in north-central Nigeria. *J Vector Borne Dis* 2011; **48**(2): 109-12.
390. Hemingway J, Lindsay SW, Small GJ, Jawara M, Collins FH. Insecticide susceptibility status in individual species of the Anopheles gambiae complex (Diptera: Culicidae) in an area of The Gambia where pyrethroid impregnated bednets are used extensively for malaria control. *Bulletin of Entomological Research* 1995; **85**(2): 229-34.
391. Kura Shehu I, Ahmad HB, Kayode Olayemi I, Solomon D, Hassan Ahmad A, Salim H. Insecticide susceptibility status in two medically important mosquito vectors, Anopheles gambiae, and Culex quinquefasciatus to three insecticides commonly used in Niger State, Nigeria. *Saudi Journal of Biological Sciences* 2023; **30**(2).
392. Fodjo BK, Koudou BG, Tia E, et al. Insecticides Resistance Status of An. gambiae in Areas of Varying Agrochemical Use in Côte D'Ivoire. *BioMed Research International* 2018; **2018**: 1-9.
393. Faye O, Diallo M, Dia I, et al. Integrated approach of yellow fever surveillance: Pilot study in Senegal in 2003-2004. *Bulletin de la Societe de Pathologie Exotique* 2007; **100**(3): 187-92.
394. Sagbohan HW, Kpanou CD, Padonou GG, et al. Intensity and different genes involved in the resistance of An. gambiae s.l. to pyrethroids in four districts representative of the different agricultural production zones of North Benin, West Africa. *International Journal of Mosquito Research* 2022; **9**(2): 15-25.
395. Sagbohan HW, Kpanou CD, Osse R, et al. Intensity and mechanisms of deltamethrin and permethrin resistance in Anopheles gambiae s.l. populations in southern Benin. *Parasites & Vectors* 2021; **14**(1).
396. Terenius O, Chen K, Ponnusamy L, et al. Internal and external microbiota of home-caught Anopheles coluzzii (Diptera: Culicidae) from Côte d'Ivoire, Africa: Mosquitoes are filthy. *Plos One* 2022; **17**(12).
397. Hanemaaijer MJ, Higgins H, Eralp I, et al. Introgression between Anopheles gambiae and Anopheles coluzzii in Burkina Faso and its associations with kdr resistance and Plasmodium infection. *Malar J* 2019; **18**(1).
398. Thiaw O, Doucouré S, Sougoufara S, et al. Investigating insecticide resistance and knock-down resistance (kdr) mutation in Dielmo, Senegal, an area under long lasting insecticidal-treated nets universal coverage for 10 years. *Malar J* 2018; **17**(1).
399. Samb B, Konate L, Irving H, et al. Investigating molecular basis of lambda-cyhalothrin resistance in an Anopheles funestus population from Senegal. *Parasites & Vectors* 2016; **9**(1).
400. Orsborne J, Furuya-Kanamori L, Jeffries CL, et al. Investigating the blood-host plasticity and dispersal of Anopheles coluzzii using a novel field-based methodology. *Parasites & Vectors* 2019; **12**(1).
401. Collins EL, Vaselli NM, Sylla M, et al. Investigating the relationship between insecticide resistance, underlying molecular mechanisms and malaria prevalence in anopheles Gambiae s.l from guinea. *Transactions of the Royal Society of Tropical Medicine and Hygiene* 2019; **113**: S105.
402. Terenius O, Krajacich BJ, Huestis DL, et al. Investigation of the seasonal microbiome of Anopheles coluzzii mosquitoes in Mali. *Plos One* 2018; **13**(3).
403. Ossè RA, Tokponnon F, Padonou GG, et al. Involvement of Anopheles nili in Plasmodium falciparum transmission in North Benin. *Malar J* 2019; **18**(1).

404. Caputo B, Tondossoma N, Virgillito C, et al. Is Côte D'Ivoire a new high hybridization zone for the two major malaria vectors, Anopheles coluzzii and An. gambiae (Diptera, Culicidae)? *Infection, Genetics and Evolution* 2022; **98**.
405. Stolk WA, Pam DD, de Souza DK, et al. Is mass drug administration against lymphatic filariasis required in urban settings? The experience in Kano, Nigeria. *PLOS Neglected Tropical Diseases* 2017; **11**(10).
406. Manoukis NC, Touré MB, Sissoko I, et al. Is Vector Body Size the Key to Reduced Malaria Transmission in the Irrigated Region of Niono, Mali? *Journal of Medical Entomology* 2006; **43**(5): 820-7.
407. Boorman JPT. Isolations of arboviruses in the Lagos area of Nigeria, and a survey of antibodies to them in man and animals. *Transactions of the Royal Society of Tropical Medicine and Hygiene* 1968; **62**(2): 269-77.
408. Traorelamizana M, Zeller HG, Mondo M, Hervy JP, Adam F, Digoutte JP. Isolations of West Nile and Bagaza viruses from mosquitoes (Diptera: Culicidae) in central Senegal (Ferlo). *J Med Entomol* 1994; **31**(6): 934-8.
409. Faiman R, Yaro AS, Dao A, et al. Isotopic evidence that aestivation allows malaria mosquitoes to persist through the dry season in the Sahel. *Nature Ecology & Evolution* 2022; **6**(11): 1687-99.
410. Abdoulaye D, Thierry B, Chandre C, et al. KDR Mutation, a Genetic Marker to Assess Events of Introgression Between the Molecular M and S Forms of *Anopheles gambiae* (Diptera: Culicidae) in the Tropical Savannah Area of West Africa. *Journal of Medical Entomology* 2003; **40**(2): 195-8.
411. Bayili K, Ki HD, Bayili B, et al. Laboratory and experimental hut trial evaluation of VECTRON™ T500 for indoor residual spraying (IRS) against insecticide resistant malaria vectors in Burkina Faso. *Gates Open Research* 2022; **6**.
412. Majori G, Ali A, Sabatinelli G. Laboratory and field efficacy of Bacillus thuringiensis var. Israelensis and Bacillus sphaericus against Anopheles gambiae s.l. and Culex quinquefasciatus in Ouagadougou, Burkina Faso. *J Am Mosq Control Assoc* 1987; **3**(1): 20-5.
413. Traoré A, Niyondiko G, Sanou A, et al. Laboratory and field evaluation of MAÏA®, an ointment containing N,N-diethyl-3-methylbenzamide (DEET) against mosquitoes in Burkina Faso. *Malar J* 2021; **20**(1).
414. Barde AA-H, Umar A, Dogara MM. Laboratory evaluation of entomopathogenic fungus Metarhizium anisopliae for the control of Anopheles gambiae s. L. Larvae in Bauchi Nigeria. *International Journal of Entomology Research* 2017; **2**(3): 10-4.
415. Ibrahim KT, Popoola KO, Akure KO. Laboratory Evaluation of Residual Efficacy of Actellic 300 CS (Pirimiphos-Methyl) and K-Othrine WG 250 (Deltamethrin) on Different Indoor Surfaces. *International Journal of Insect Science* 2017; **9**.
416. Sawadogo SP, Kabore DA, Tibiri EB, et al. Lack of robust evidence for a Wolbachia infection in Anopheles gambiae from Burkina Faso. *Medical and Veterinary Entomology* 2022; **36**(3): 301-8.
417. Gubler DJ, Diallo D, Sall AA, et al. Landscape Ecology of Sylvatic Chikungunya Virus and Mosquito Vectors in Southeastern Senegal. *PLoS Neglected Tropical Diseases* 2012; **6**(6).
418. Traore MM, Junnila A, Traore SF, et al. Large-scale field trial of attractive toxic sugar baits (ATSB) for the control of malaria vector mosquitoes in Mali, West Africa. *Malar J* 2020; **19**(1).
419. Kudom AA. Larval ecology of Anopheles coluzzii in Cape Coast, Ghana: water quality, nature of habitat and implication for larval control. *Malar J* 2015; **14**(1).
420. Hinne IA, Attah SK, Mensah BA, Forson AO, Afrane YA. Larval habitat diversity and Anopheles

mosquito species distribution in different ecological zones in Ghana. *Parasites & Vectors* 2021; **14**(1).
421. Gimonneau G, Pombi M, Choisy M, Morand S, DabirÉ RK, Simard F. Larval habitat segregation between the molecular forms of the mosquito Anopheles gambiae in a rice field area of Burkina Faso, West Africa. *Medical and Veterinary Entomology* 2012; **26**(1): 9-17.
422. Forson AO, Hinne IA, Sraku IK, Afrane YA. Larval habitat stability and productivity in two sites in Southern Ghana. *Malar J* 2023; **22**(1).
423. Adeleke MA, Adebimpe WO, Hassan AO, et al. Larval habitats of mosquito fauna in Osogbo metropolis, Southwestern Nigeria. *Asian Pacific Journal of Tropical Biomedicine* 2013; **3**(9): 673-7.
424. Touré M, Carnevale P, Chandre F. [Late impact of lambdacyhalothrin-treated nets on kdr allelic frequency in Anopheles gambiae s.s. (Diptera: Culicidae) from northern Côte-d'Ivoire]. *Bull Soc Pathol Exot* 2012; **105**(4): 305-10.
425. Digoutte J, Salaun J, Robin Y, Bres P, Cagnard V. Les arboviroses mineures en Afrique Centrale et Occidentale. *Med Trop* 1980; **40**: 5.
426. Edi CAV, Koudou BG, Bellai L, et al. Long-term trends in Anopheles gambiae insecticide resistance in Côte d'Ivoire. *Parasites & Vectors* 2014; **7**(1).
427. Labbo R, Czeher C, Djibrila A, Arzika I, Jeanne I, Duchemin JB. Longitudinal follow-up of malaria transmission dynamics in two villages in a Sahelian area of Niger during a nationwide insecticide-treated bednet distribution programme. *Medical and Veterinary Entomology* 2012; **26**(4): 386-95.
428. Irish SR, N'Guessan R, Boko PM, et al. Loss of protection with insecticide-treated nets against pyrethroid-resistant Culex quinquefasciatus mosquitoes once nets become holed: an experimental hut study. *Parasites & Vectors* 2008; **1**(1).
429. Kouassi BL, de Souza DK, Goepogui A, et al. Low prevalence of Plasmodium and absence of malaria transmission in Conakry, Guinea: prospects for elimination. *Malar J* 2016; **15**(1).
430. Remais JV, Yokoly FN, Zahouli JBZ, et al. Low transmission of Wuchereria bancrofti in cross-border districts of Côte d'Ivoire: A great step towards lymphatic filariasis elimination in West Africa. *Plos One* 2020; **15**(4).
431. Appawu MA, Dadzie SK, Baffoe-Wilmot A, Wilson MD. Lymphatic filariasis in Ghana: entomological investigation of transmission dynamics and intensity in communities served by irrigation systems in the Upper East Region of Ghana. *Trop Med Int Health* 2001; **6**(7): 511-6.
432. Gbakima AA, Appawu MA, Dadzie S, et al. Lymphatic filariasis in Ghana: establishing the potential for an urban cycle of transmission. *Trop Med Int Health* 2005; **10**(4): 387-92.
433. Orlandi-Pradines E, Rogier C, Koffi B, et al. Major variations in malaria exposure of travellers in rural areas: an entomological cohort study in western Côte d'Ivoire. *Malar J* 2009; **8**(1).
434. Wragge S-E, Toure D, Coetzee M, et al. Malaria control at a gold mine in Sadiola District, Mali, and impact on transmission over 10 years. *Transactions of The Royal Society of Tropical Medicine and Hygiene* 2015; **109**(12): 755-62.
435. Ouldabdallahi Moukah M, Ba O, Ba H, et al. Malaria in three epidemiological strata in Mauritania. *Malar J* 2016; **15**(1).
436. Afari EA, Appawu M, Dunyo S, Baffoe-Wilmot A, Nkrumah FK. Malaria infection, morbidity and transmission in two ecological zones Southern Ghana. *Afr J Health Sci* 1995; **2**(2): 312-5.
437. Trape J-F, Tall A, Diagne N, et al. Malaria morbidity and pyrethroid resistance after the introduction of insecticide-treated bednets and artemisinin-based combination therapies: a longitudinal study. *The Lancet Infectious Diseases* 2011; **11**(12): 925-32.

438. Onyido AE, Obinatu SC, Umeanaeto PU, Obiukwu MO, Egbuche MC. Malaria prevalence and mosquito vector abundance in Uli town, Ihiala Local Government Area, Anambra State, Nigeria. *African Journal of Biomedical Research* 2011; **14**(3): 175-82.
439. Girod R, Orlandi-Pradines E, Rogier C, Pages F. Malaria Transmission and Insecticide Resistance of *Anopheles gambiae* (Diptera: Culicidae) in the French Military Camp of Port-Bouët, Abidjan (Côte d'Ivoire): Implications for Vector Control. *Journal of Medical Entomology* 2006; **43**(5): 1082-7.
440. Appawu M, Owusu-Agyei S, Dadzie S, et al. Malaria transmission dynamics at a site in northern Ghana proposed for testing malaria vaccines. *Trop Med Int Health* 2004; **9**(1): 164-70.
441. Basing AW, Tay S. Malaria transmission dynamics of the Anopheles mosquito in Kumasi, Ghana. *International Journal of Infectious Diseases* 2014; **21**.
442. Pagès F, Texier G, Pradines B, et al. Malaria transmission in Dakar: A two-year survey. *Malar J* 2008; **7**(1).
443. Fofana D, Koné A, Konan Y, Konan K, Doannio J, N'Goran E. [Malaria transmission in lagoon areas in Côte d'Ivoire: the villages of N'gatty and Allaba]. *Sante* 2010; **20**(2): 73-9.
444. Dolo G, Briët OJT, Dao A, et al. Malaria transmission in relation to rice cultivation in the irrigated Sahel of Mali. *Acta Tropica* 2004; **89**(2): 147-59.
445. Vezenegho SB, Brooke BD, Hunt RH, Coetzee M, Koekemoer LL. Malaria vector composition and insecticide susceptibility status in Guinea Conakry, West Africa. *Med Vet Entomol* 2009; **23**(4): 326-34.
446. Lamidi BT, Naphtali RS, Alo EB, Oyeniyi AT. Malaria vector population density and man-biting rate in three selected areas of Taraba State, north-east Nigeria. *Nigerian Journal of Parasitology* 2018; **39**(2).
447. Coulibaly B, Kone R, Barry MS, et al. Malaria vector populations across ecological zones in Guinea Conakry and Mali, West Africa. *Malar J* 2016; **15**(1).
448. Appawu MA, Baffoe-Wilmot A, Afari EA, Dunyo S, Koram KA, Nkrumah FK. Malaria vector studies in two ecological zones in southern Ghana. *African Entomology* 2001; **9**(1): 59-65.
449. Yaro CA, Udoidung NI, Ezihe EK, Akaka B, Opara KN, Chikezie FM. Malaria vectors distribution, abundance and assessment of factors influencing acceptance and use of Insecticide Treated Nets in Uyo Akwa Ibom State Nigeria. *African Journal of Biological Sciences* 2021; **3**(1): 165-75.
450. Soma DD, Poda SB, Hien AS, et al. Malaria vectors diversity, insecticide resistance and transmission during the rainy season in peri-urban villages of south-western Burkina Faso. *Malar J* 2021; **20**(1).
451. Djogbénou L, Pasteur N, Bio-Bangana S, et al. Malaria vectors in the Republic of Benin: Distribution of species and molecular forms of the Anopheles gambiae complex. *Acta Tropica* 2010; **114**(2): 116-22.
452. Gnanguenon V, Agossa FR, Badirou K, et al. Malaria vectors resistance to insecticides in Benin: current trends and mechanisms involved. *Parasites & Vectors* 2015; **8**(1).
453. Onyishi GC, Aguzie ION, Nwani CD, Obiezue RNN, Okoye IC. Malaria-Vector Dynamics in a Tropical Urban Metropolis, Nigeria. *Pakistan Journal of Zoology* 2018; **50**(3).
454. Shishika D, Manoukis NC, Butail S, Paley DA. Male motion coordination in anopheline mating swarms. *Scientific Reports* 2014; **4**(1).
455. Fox RM. Man-biting mosquitoes in coastal Liberia. *Am J Trop Med Hyg* 1958; **7**(2): 215-20.
456. Konan YL, Coulibaly ZI, Allali KB, et al. [Management of the yellow fever epidemic in 2010 in Séguéla (Côte d'Ivoire): value of multidisciplinary investigation]. *Sante Publique* 2014; **26**(6): 859-67.

457. Ughasi J, Bekard HE, Coulibaly M, et al. Mansonia africana and Mansonia uniformis are vectors in the transmission of Wuchereria bancrofti lymphatic filariasis in Ghana. *Parasit Vectors* 2012; **5**: 89.
458. Carvalho LH, Ndiaye A, Niang EHA, et al. Mapping the breeding sites of Anopheles gambiae s. l. in areas of residual malaria transmission in central western Senegal. *Plos One* 2020; **15**(12).
459. Djouaka R, Akoton R, Tchigossou GM, et al. Mapping the distribution of Anopheles funestus across Benin highlights a sharp contrast of susceptibility to insecticides and infection rate to Plasmodium between southern and northern populations. *Wellcome Open Research* 2016; **1**.
460. Touré YT, Dolo G, Petrarca V, et al. Mark-release-recapture experiments with Anopheles gambiae s.l. in Banambani Village, Mali, to determine population size and structure. *Med Vet Entomol* 1998; **12**(1): 74-83.
461. Sylla M, Gray M, Chapman PL, Sarr MD, Rasgon JL. Mass drug administration of ivermectin in south-eastern Senegal reduces the survivorship of wild-caught, blood fed malaria vectors. *Malar J* 2010; **9**(1).
462. Bouraima A, Djènontin A, Dossou Y, et al. Measuring entomological parameters before implementing a study on asymptomatic carriers of Plasmodium falciparum in the Zè District in southern Benin. *Malar J* 2023; **22**(1).
463. Kobylinski KC, Sylla M, Black W, Foy BD. Mermithid nematodes found in adult anopheles from southeastern Senegal. *Parasites and Vectors* 2012; **5**(1).
464. Ndiath MO, Mazenot C, Gaye A, et al. Methods to collect Anopheles mosquitoes and evaluate malaria transmission: A comparative study in two villages in Senegal. *Malar J* 2011; **10**.
465. Tchicaya ES, Nsanzabana C, Smith TA, et al. Micro-encapsulated pirimiphos-methyl shows high insecticidal efficacy and long residual activity against pyrethroid-resistant malaria vectors in central Cote d'Ivoire. *Malar J* 2014; **13**.
466. Sovi A, Djègbè I, Soumanou L, et al. Microdistribution of the resistance of malaria vectors to deltamethrin in the region of Plateau (southeastern Benin) in preparation for an assessment of the impact of resistance on the effectiveness of Long Lasting Insecticidal Nets (LLINs). *BMC Infect Dis* 2014; **14**: 103.
467. Akorli J, Akorli EA, Tetteh SNA, et al. Microsporidia MB is found predominantly associated with Anopheles gambiae s.s and Anopheles coluzzii in Ghana. *Scientific Reports* 2021; **11**(1).
468. Straif SC, Mbogo CNM, Toure AM, et al. Midgut Bacteria in Anopheles gambiae and An. funestus (Diptera: Culicidae) from Kenya and Mali. *Journal of Medical Entomology* 1998; **35**(3): 222-6.
469. Obembe A, Popoola KOK, Oduola AO, Awolola ST. Mind the weather: a report on inter-annual variations in entomological data within a rural community under insecticide-treated wall lining installation in Kwara State, Nigeria. *Parasites & Vectors* 2018; **11**(1).
470. Diabaté A, Dabire RK, Kengne P, et al. Mixed Swarms of the Molecular M and S Forms of *Anopheles gambiae* (Diptera: Culicidae) in Sympatric Area from Burkina Faso. *Journal of Medical Entomology* 2006; **43**(3): 480-3.
471. Aïzoun N, Aïkpon R, Padonou GG, et al. Mixed-function oxidases and esterases associated with permethrin, deltamethrin and bendiocarb resistance in Anopheles gambiae s.l. in the south-north transect Benin, West Africa. *Parasit Vectors* 2013; **6**: 223.
472. Moiroux N, Bio-Bangana AS, Djènontin A, Chandre F, Corbel V, Guis H. Modelling the risk of being bitten by malaria vectors in a vector control area in southern Benin, west Africa. *Parasites and Vectors* 2013; **6**(1).

473. Ukubuiwe AC, Olayemi IK, Omalu ICJ, Jibrin A, Oyibo-Usma K. Molecular Bases of Reproductive and Vectorial Fitness of Culex pipiens pipiens (Diptera: Culicidae) Mosquito Populations, for the Transmission of Filariasis in North Central Nigeria. *Journal of Medical Sciences* 2013; **13**(3): 201-7.
474. Tchigossou G, Djouaka R, Akoton R, et al. Molecular basis of permethrin and DDT resistance in an Anopheles funestus population from Benin. *Parasites & Vectors* 2018; **11**(1).
475. D.G.W WC, Mwansat GS, Owino EA, Oyieke FA, Garba LC. Molecular characterization and species composition of anopheline vectors of malaria along an altitudinal gradient on the highlands of Mambilla Plateau Northeast, Nigeria. *African Journal of Biological Sciences* 2020; **02**(01).
476. Djègbè I, Agossa FR, Jones CM, et al. Molecular characterization of DDT resistance in Anopheles gambiae from Benin. *Parasit Vectors* 2014; **7**: 409.
477. Yin J, Yamba F, Zheng C, et al. Molecular Detection of Insecticide Resistance Mutations in Anopheles gambiae from Sierra Leone Using Multiplex SNaPshot and Sequencing. *Frontiers in Cellular and Infection Microbiology* 2021; **11**.
478. della Torre A, Fanello C, Akogbeto M, et al. Molecular evidence of incipient speciation within Anopheles gambiae s.s. in West Africa. *Insect Mol Biol* 2001; **10**(1): 9-18.
479. Onoja AB, Maina MM, Opayele AV. Molecular Identification and Phylogenetic Analysis of Acetylcholinesterase-2 Gene From Culex pipiens And Culex quinquefasciatus (Diptera: Culicine) in Nigeria. *Niger Vet J* 2018; **39**(4): 285-94.
480. Zhao N, Zhao J, Song X, et al. Molecular identification and role in malaria transmission of Anopheles gambiae complex in dry season in Freetown, Sierra Leone,2019-2020. *Disease Surveillance* 2021; **36**(5): 411-6.
481. Akeju AV, Olusi TA, Simon-Oke IA. Molecular identification and wing variations among malaria vectors in Akure North Local Government Area, Nigeria. *Scientific Reports* 2022; **12**(1).
482. Dorkenoo MA, de Souza DK, Apetogbo Y, et al. Molecular xenomonitoring for post-validation surveillance of lymphatic filariasis in Togo: no evidence for active transmission. *Parasites & Vectors* 2018; **11**(1).
483. Silva R, Mavridis K, Vontas J, Rodrigues A, Osório HC. Monitoring and molecular profiling of contemporary insecticide resistance status of malaria vectors in Guinea–Bissau. *Acta Tropica* 2020; **206**.
484. Namountougou M, Soma DD, Balboné M, et al. Monitoring Insecticide Susceptibility in Aedes Aegypti Populations from the Two Biggest Cities, Ouagadougou and Bobo-Dioulasso, in Burkina Faso: Implication of Metabolic Resistance. *Tropical Medicine and Infectious Disease* 2020; **5**(2).
485. Sogoba N, Smith T, Traoré SF, et al. Monitoring of Larval Habitats and Mosquito Densities in the Sudan Savanna of Mali: Implications for Malaria Vector Control. *The American Journal of Tropical Medicine and Hygiene* 2007; **77**(1): 82-8.
486. Shehu IK, Ahmad H, Ishak IH, et al. Monthly assessment of the relative abundance and development of immature stages of Culex mosquitoes (Diptera: Culicidae) of the selected breeding habitats in Niger, Nigeria. *Serangga* 2022; **27**(3): 187-203.
487. Atting I, Akpan M. Morphological and Molecular Characterization of Anopheles Mosquitoes and Their Behavioural Patterns in Uyo, Nigeria. *International Journal of TROPICAL DISEASE & Health* 2016; **14**(2): 1-11.
488. Schneider BS, Sanford MR, Demirci B, et al. Morphological Differentiation May Mediate Mate-Choice between Incipient Species of Anopheles gambiae s.s. *PLoS ONE* 2011; **6**(11).

489. Akeju AV. Morphometric variations in morphological features of Anopheles gambiae complex in Akure North Local Government area of Ondo State Nigeria. *International Journal of Tropical Insect Science* 2022; **42**(5): 3551-61.
490. Hahn M, Wang-Sattler R, Blandin S, et al. Mosaic Genome Architecture of the Anopheles gambiae Species Complex. *PLoS ONE* 2007; **2**(11).
491. Anyanwu GI, Iwuala MOE. Mosquito breeding sites: Distribution and relative abundance of species in the Jos Plateau, Nigeria. *Medical Entomology and Zoology* 1999; **50**(3): 243-9.
492. Oppong J, Avicor SW, Baidoo PK, Addo-Fordjour P. Mosquito community structure in contrasting anthropized landscapes in the Kumasi Metropolis of Ghana. *International Journal of Tropical Insect Science* 2022; **43**(1): 21-31.
493. Umeanaeto PU, Onyido AE, Ifeanyichukwu MO, Anumba JU. Mosquito dynamics and malaria in Alulu-Nike Commnuity, Enugu East Local Government Area, Enugu State, Nigeria. *Nigerian Journal of Parasitology* 2019; **40**(1).
494. Lingenfelser A, Rydzanicz K, Kaiser A, Becker N. Mosquito fauna and perspectives for integrated control of urban vector-mosquito populations in Southern Benin (West Africa). *Ann Agric Environ Med* 2010; **17**(1): 49-57.
495. Adeleke MA, Mafiana CF, Idowu AB, Adekunle MF, Sam-Wobo SO. Mosquito larval habitats and public health implications in Abeokuta, Ogun State, Nigeria. *Tanzan J Health Res* 2008; **10**(2): 103-7.
496. Sullivan DJ, N'Guessan R, Ngufor C, et al. Mosquito Nets Treated with a Mixture of Chlorfenapyr and Alphacypermethrin Control Pyrethroid Resistant Anopheles gambiae and Culex quinquefasciatus Mosquitoes in West Africa. *PLoS ONE* 2014; **9**(2).
497. Mellanby K. Mosquito populations at ibadan in nigeria. *Bulletin of Entomological Research* 1956; **47**(1): 125-36.
498. Costantini C, Gibson G, Sagnon N, Della Torre A, Brady J, Coluzzi M. Mosquito responses to carbon dioxide in a west African Sudan savanna village. *Med Vet Entomol* 1996; **10**(3): 220-7.
499. Oforka CL, Omotayo AI, Adeogun AO, Anikwe JC, Adeleke MA. Mosquito species composition in two urban neighbourhoods of Lagos, Southwest Nigeria, based on larval surveys. *International Journal of Tropical Insect Science* 2022; **42**(3): 2751-60.
500. Beechler BR, Stoek F, Barry Y, et al. Mosquito survey in Mauritania: Detection of Rift Valley fever virus and dengue virus and the determination of feeding patterns. *PLOS Neglected Tropical Diseases* 2022; **16**(4).
501. Diallo M, Nabeth P, Ba K, et al. Mosquito vectors of the 1998-1999 outbreak of Rift Valley Fever and other arboviruses (Bagaza, Sanar, Wesselsbron and West Nile) in Mauritania and Senegal. *Med Vet Entomol* 2005; **19**(2): 119-26.
502. Bertram DS, Mc GI, Mc FJ. Mosquitoes of the Colony and Protectorate of the Gambia. *Trans R Soc Trop Med Hyg* 1958; **52**(2): 135-51.
503. Thomson MC, Connor SJ, Quiñones ML, Jawara M, Todd J, Greenwood BM. Movement of Anopheles gambiae s.l. malaria vectors between villages in The Gambia. *Med Vet Entomol* 1995; **9**(4): 413-9.
504. Toé HK, Zongo S, Guelbeogo MW, et al. Multiple insecticide resistance and first evidence of V410L kdr mutation in Aedes (Stegomyia) aegypti (Linnaeus) from Burkina Faso. *Medical and Veterinary Entomology* 2022; **36**(3): 309-19.
505. Riveron JM, Osae M, Egyir-Yawson A, Irving H, Ibrahim SS, Wondji CS. Multiple insecticide resistance in the major malaria vector Anopheles funestus in southern Ghana: implications for malaria

control. *Parasites & Vectors* 2016; **9**(1).
506. Corbel V, N'Guessan R, Brengues C, et al. Multiple insecticide resistance mechanisms in Anopheles gambiae and Culex quinquefasciatus from Benin, West Africa. *Acta Tropica* 2007; **101**(3): 207-16.
507. Diouf Eh, Niang EhA, Samb B, et al. Multiple insecticide resistance target sites in adult field strains of An. gambiae (s.l.) from southeastern Senegal. *Parasites & Vectors* 2020; **13**(1).
508. Soumaila H, Idrissa M, Akgobeto M, et al. Multiple mechanisms of resistance to pyrethroids in Anopheles gambiae s.l populations in Niger. *Médecine et Maladies Infectieuses* 2017; **47**(6): 415-23.
509. Keïta M, Sogoba N, Kané F, et al. Multiple Resistance Mechanisms to Pyrethroids Insecticides in Anopheles gambiae sensu lato Population From Mali, West Africa. *The Journal of Infectious Diseases* 2021; **223**(Supplement_2): S81-S90.
510. Diabate A, Baldet T, Brengues C, et al. Natural swarming behaviour of the molecular M form of Anopheles gambiae. *Trans R Soc Trop Med Hyg* 2003; **97**(6): 713-6.
511. Vasilakis N, Forrester NL, Palacios G, et al. Negevirus: a Proposed New Taxon of Insect-Specific Viruses with Wide Geographic Distribution. *Journal of Virology* 2013; **87**(5): 2475-88.
512. Wragge S-E, Venter N, Touré D, Hunt RH, Coetzee M. New distribution record of Anopheles rivulorum-like from Sadiola, Mali, with notes on malaria vector insecticide resistance. *Transactions of The Royal Society of Tropical Medicine and Hygiene* 2021; **115**(5): 495-9.
513. Jawara M, Jatta E, Bell D, et al. New Prototype Screened Doors and Windows for Excluding Mosquitoes from Houses: A Pilot Study in Rural Gambia. *The American Journal of Tropical Medicine and Hygiene* 2018; **99**(6): 1475-84.
514. Dambach P, Schleicher M, Korir P, et al. Nightly Biting Cycles of Anopheles Species in Rural Northwestern Burkina Faso. *Journal of Medical Entomology* 2018; **55**(4): 1027-34.
515. Hoerauf A, de Souza DK, Sesay S, et al. No Evidence for Lymphatic Filariasis Transmission in Big Cities Affected by Conflict Related Rural-Urban Migration in Sierra Leone and Liberia. *PLoS Neglected Tropical Diseases* 2014; **8**(2).
516. Kuhlow F. Normal Susceptibility Levels of Anopheles pharoensis Theobald to DDT and Dieldrin. *Nature* 1960; **186**(4722): 410-.
517. Surtees G. Notes on the breeding habits of some culicine mosquitoes (Diptera: Culicidae) in Southern Ghana. *PhysiolEntomol* 1958; **33**(4-6): 88-92.
518. Jeffries CL, Lawrence GG, Golovko G, et al. Novel Wolbachia strains in Anopheles malaria vectors from Sub-Saharan Africa. *Wellcome Open Research* 2018; **3**.
519. Don-Pedro KN, Adegbite TO. Nuvan resistance in a field strain of Aedes aegypti (L.) in Lagos, Nigeria. *Environmental Pollution Series A: Ecological and Biological* 1985; **38**(1): 19-29.
520. Petrarca V, Vercruysse J, Coluzzi M. Observations on the Anopheles gambiae complex in the Senegal River Basin, West Africa. *Med Vet Entomol* 1987; **1**(3): 303-12.
521. Beeuwkes H, Kerr JA, Weathersbee AA, Taylor AW. Observations on the bionomics and comparative prevalence of the vectors of yellow fever and other domestic mosquitoes of west africa, and the epidemiological significance of seasonal variations. *Transactions of the Royal Society of Tropical Medicine and Hygiene* 1933; **26**(5): 425-47.
522. Boorman JPT. Observations on the habits of mosquitos of Plateau Province, Northern Nigeria, with particular reference to Aedes (Stegomyia) vittatus (Bigot). *Bulletin of Entomological Research* 1961; **52**(4): 709-25.
523. Bryan JH. Observations on the member species of the Anopheles gambiae complex in The

Gambia, West Africa. *Trans R Soc Trop Med Hyg* 1979; **73**(4): 463-6.
524. Ogunba EO. Observations onCulex pipiens fatigansin Ibadan, western Nigeria. *Annals of Tropical Medicine & Parasitology* 2016; **65**(3): 399-402.
525. Dabiré KR, Sawadogo PS, Hien DF, et al. Occurrence of natural Anopheles arabiensis swarms in an urban area of Bobo-Dioulasso city, Burkina Faso, West Africa. *Acta Tropica* 2014; **132**: S35-S41.
526. Costantini C, Coluzzi M, Gibson G, et al. Odor-mediated host preferences of West African mosquitoes, with particular reference to malaria vectors. *The American Journal of Tropical Medicine and Hygiene* 1998; **58**(1): 56-63.
527. Paul RE, Ngufor C, N'Guessan R, et al. Olyset Duo® (a Pyriproxyfen and Permethrin Mixture Net): An Experimental Hut Trial against Pyrethroid Resistant Anopheles gambiae and Culex quinquefasciatus in Southern Benin. *PLoS ONE* 2014; **9**(4).
528. Diarra K, Toguebaye BS. On the development cycle and ultrastructure of Vavraia culicis Weiser, 1947 (microsporida, pleistophoridae) with comments on the taxonomy of the genus Vavraia Weiser, 1977. *European Journal of Protistology* 1991; **27**(2): 134-40.
529. Surtees G. On the distribution and seasonal incidence of Culicine mosquitoesS in Southern Nigeria. *PhysiolEntomol* 1959; **34**(7-9): 110-20.
530. Gruner AC, Sandeu MM, Moussiliou A, et al. Optimized Pan-species and Speciation Duplex Real-time PCR Assays for Plasmodium Parasites Detection in Malaria Vectors. *PLoS ONE* 2012; **7**(12).
531. Snounou G, Jawara M, Smallegange RC, et al. Optimizing Odor-Baited Trap Methods for Collecting Mosquitoes during the Malaria Season in The Gambia. *PLoS ONE* 2009; **4**(12).
532. Vazeille M, Yébakima A, Lourenço-de-Oliveira R, et al. Oral Receptivity ofAedes aegyptifrom Cape Verde for Yellow Fever, Dengue, and Chikungunya Viruses. *Vector-Borne and Zoonotic Diseases* 2013; **13**(1): 37-40.
533. Anges Y, Falilath S, Yvette B, Gildas H, Carine T, Lamine B-M. Origin of blood meal sources in Culex quinquefasciatus in northern Benin. *International Journal of Mosquito Research* 2022; **9**(2): 100-3.
534. Bassolé IH, Guelbeogo WM, Nébié R, et al. Ovicidal and larvicidal activity against Aedes aegypti and Anopheles gambiae complex mosquitoes of essential oils extracted from three spontaneous plants of Burkina Faso. *Parassitologia* 2003; **45**(1): 23-6.
535. Zahouli JBZ, Utzinger J, Adja MA, et al. Oviposition ecology and species composition of Aedes spp. and Aedes aegypti dynamics in variously urbanized settings in arbovirus foci in southeastern Côte d'Ivoire. *Parasites & Vectors* 2016; **9**(1).
536. Coleman S, Yihdego Y, Sherrard-Smith E, et al. Partial indoor residual spraying with pirimiphos-methyl as an effective and cost-saving measure for the control of Anopheles gambiae s.l. in northern Ghana. *Scientific Reports* 2021; **11**(1).
537. Aka KG, Traoré DF, Sagna AB, et al. Pattern of antibody responses to Plasmodium falciparum antigens in individuals differentially exposed to Anopheles bites. *Malar J* 2020; **19**(1).
538. Boakye DA, Adasi K, Appawu M, Brown CA, Wilson MD. Patterns of Household Insecticide Use and Pyrethroid Resistance inAnopheles Gambiae Sensu Stricto(Diptera: Culicidae) within the Accra Metropolis of Ghana. *African Entomology* 2009; **17**(2): 125-30.
539. Niang EHA, Konaté L, Diallo M, Faye O, Dia I. Patterns of insecticide resistance and knock down resistance (kdr) in malaria vectors An. arabiensis, An. coluzzii and An. gambiae from sympatric areas in Senegal. *Parasites & Vectors* 2016; **9**(1).
540. Diuk-Wasser MA, Dolo G, Bagayoko M, et al. Patterns of irrigated rice growth and malaria vector

breeding in Mali using multi-temporal ERS-2 synthetic aperture radar. *International Journal of Remote Sensing* 2007; **27**(3): 535-48.
541. Niang A, Maïga H, Sawadogo SP, et al. Perfect association between spatial swarm segregation and the X-chromosome speciation island in hybridizing Anopheles coluzzii and Anopheles gambiae populations. *Scientific Reports* 2022; **12**(1).
542. Keïta M, Sogoba N, Traoré B, et al. Performance of pirimiphos-methyl based Indoor Residual Spraying on entomological parameters of malaria transmission in the pyrethroid resistance region of Koulikoro, Mali. *Acta Tropica* 2021; **216**.
543. Iwuala MOE. Peri-Domestic Ecology of Dry-Season Populations of Aedes (Stegomyia) Mosquitoes in Uyo, Cross River State, Nigeria. *Environ Entomol* 1981; **10**(5): 592-9.
544. Dabiré RK, Diabaté A, Baldet T, et al. Personal protection of long lasting insecticide-treated nets in areas of Anopheles gambiae s.s. resistance to pyrethroids. *Malar J* 2006; **5**(1).
545. Fassinou AJYH, Koukpo CZ, Ossè RA, et al. Pesticides and the evolution of the genetic structure of Anopheles coluzzii populations in some localities in Benin (West Africa). *Malar J* 2019; **18**(1).
546. Medjigbodo AA, Djogbenou LS, Koumba AA, et al. Phenotypic Insecticide Resistance in Anopheles gambiae (Diptera: Culicidae): Specific Characterization of Underlying Resistance Mechanisms Still Matters. *Journal of Medical Entomology* 2021; **58**(2): 730-8.
547. Huestis DL, Artis ML, Armbruster PA, Lehmann T. Photoperiodic responses of Sahelian malaria mosquitoes Anopheles coluzzii and An. arabiensis. *Parasites & Vectors* 2017; **10**(1).
548. Mouline K, Mamai W, Agnew P, et al. Physiology and development of the M and S molecular forms ofAnopheles gambiaein Burkina Faso (West Africa). *Medical and Veterinary Entomology* 2012; **26**(4): 447-54.
549. Mosqueira B, Soma DD, Namountougou M, et al. Pilot study on the combination of an organophosphate-based insecticide paint and pyrethroid-treated long lasting nets against pyrethroid resistant malaria vectors in Burkina Faso. *Acta Tropica* 2015; **148**: 162-9.
550. Brooke B, Fongnikin A, Odjo A, Akpi J, Kiki L, Ngufor C. Pirikool® 300 CS, a new long-lasting capsule suspension formulation of the organophosphate insecticide pirimiphos-methyl for indoor residual spraying against pyrethroid-resistant malaria vectors. *Plos One* 2022; **17**(4).
551. Boudin C, Robert V, Verhave JP, Carnevale P, Ambroisethomas P. Plasmodium falciparum and P. malariae epidemiology in a West African village. *Bull World Health Organ* 1991; **69**(2): 199-205.
552. Salako AS, Ossè R, Padonou GG, et al. Population Dynamics of Anopheles gambiae s.l. and Culex quinquefasciatus in Rural and Urban Settings Before an Indoor Residual Spraying Campaign in Northern Benin. *Vector-Borne and Zoonotic Diseases* 2019; **19**(9): 674-84.
553. Dabire RK, Namountougou M, Sawadogo SP, et al. Population dynamics of Anopheles gambiae s.l. in Bobo-Dioulasso city: bionomics, infection rate and susceptibility to insecticides. *PARASITES & VECTORS* 2012; **5**.
554. Adeleke MA, Mafiana CF, Idowu AB, Sam-Wobo SO, Idowu OA. Population dynamics of indoor sampled mosquitoes and their implication in disease transmission in Abeokuta, south-western Nigeria. *J Vector Borne Dis* 2010; **47**(1): 33-8.
555. Czeher C, Labbo R, Vieville G, et al. Population Genetic Structure of *Anopheles gambiae* and *Anopheles arabiensis* in Niger. *Journal of Medical Entomology* 2010; **47**(3): 355-66.
556. Samb B, Dia I, Konate L, Ayala D, Fontenille D, Cohuet A. Population genetic structure of the malaria vector Anopheles funestus, in a recently re-colonized area of the Senegal River basin and human-induced environmental changes. *Parasit Vectors* 2012; **5**: 188.

557. Getz WM, Baber I, Keita M, et al. Population Size and Migration of Anopheles gambiae in the Bancoumana Region of Mali and Their Significance for Efficient Vector Control. *PLoS ONE* 2010; **5**(4).
558. Fofana M, Mitri C, Diallo D, et al. Possible influence of Plasmodium/Trypanosoma co-infections on the vectorial capacity of Anopheles mosquitoes. *BMC Research Notes* 2020; **13**(1).
559. Dinglasan RR, Gaye A, Wang E, et al. Potential for sylvatic and urban Aedes mosquitoes from Senegal to transmit the new emerging dengue serotypes 1, 3 and 4 in West Africa. *PLOS Neglected Tropical Diseases* 2019; **13**(2).
560. Vontas J, Yovogan B, Sovi A, et al. Pre-intervention characteristics of the mosquito species in Benin in preparation for a randomized controlled trial assessing the efficacy of dual active-ingredient long-lasting insecticidal nets for controlling insecticide-resistant malaria vectors. *Plos One* 2021; **16**(5).
561. Kudom AA, Mensah BA, Froeschl G, Boakye D, Rinder H. Preliminary assessment of the potential role of urbanization in the distribution of carbamate and organophosphate resistant populations of Culex species in Ghana. *Parasites & Vectors* 2015; **8**(1).
562. Hougard JM, Martin T, Guillet PF, et al. Preliminary Field Testing of a Long-Lasting Insecticide-Treated Hammock Against *Anopheles gambiae* and *Mansonia* spp. (Diptera: Culicidae) in West Africa. *Journal of Medical Entomology* 2007; **44**(4): 651-5.
563. Odetoyinbo JA. Preliminary investigation on the use of a light-trap for sampling malaria vectors in the Gambia. *Bull World Health Organ* 1969; **40**(4): 547-+.
564. Costantini C, Diallo M. Preliminary lack of evidence for simian odour preferences of savanna populations of Anopheles gambiae and other malaria vectors. *Parassitologia* 2001; **43**(4): 179-82.
565. Thabet HS, Fawaz EY, Badziklou K, et al. Preliminary Screening of Mosquito Spatial Distribution in Togo: With Special Focus on the Aedes (Diptera: Culicidae) Species. *Journal of Medical Entomology* 2019; **56**(4): 1154-8.
566. Djogbénou L, Akogbéto M, Chandre F. Presence of insensitive acetylcholinesterase in wild populations of Culex pipiens quinquefasciatus from Benin. *Acta Tropica* 2008; **107**(3): 272-4.
567. Bang YH, Bown DN, Onwubiko AO. Prevalence of larvae of potential yellow fever vectors in domestic water containers in south-east Nigeria. *Bull World Health Organ* 1981; **59**(1): 107-14.
568. Bang YH, Bown DN, Onwubiko AO. Prevalence of larvae of potential yellow fever vectors in domestic water containers in south-east Nigeria. *Bull World Health Organ* 1981; **59**(1): 107-14.
569. Safiyanu M, Alhassan A, Imam A, Abdullahi H. Pyretheroids Resistance and Detoxifying Enzymes Activities of Malaria Vector (Anopheles gambiae) Breeding in Auyo Irrigation and Residential Sites, Jigawa State, Nigeria. *Annual Research & Review in Biology* 2017; **17**(2): 1-8.
570. Brengues C, Hawkes NJ, Chandre F, et al. Pyrethroid and DDT cross-resistance in Aedes aegypti is correlated with novel mutations in the voltage-gated sodium channel gene. *Med Vet Entomol* 2003; **17**(1): 87-94.
571. Chandre F, Darriet F, Manguin S, Brengues C, Carnevale P, Guillet P. Pyrethroid cross resistance spectrum among populations of Anopheles gambiae s.s. from Côte d'Ivoire. *Journal of the American Mosquito Control Association* 1999; **15**(1): 53-9.
572. Vontas J, Temu EA, Maxwell C, et al. Pyrethroid Resistance in Anopheles gambiae, in Bomi County, Liberia, Compromises Malaria Vector Control. *PLoS ONE* 2012; **7**(9).
573. Kristan M, Fleischmann H, della Torre A, Stich A, Curtis CF. Pyrethroid resistance/susceptibility and differential urban/rural distribution of Anopheles arabiensis and An. gambiae s.s. malaria vectors in Nigeria and Ghana. *Med Vet Entomol* 2003; **17**(3): 326-32.

574. Ojianwuna CC, Omotayo AI, Enwemiwe VN, et al. Pyrethroid Susceptibility in Culex quinquefasciatus Say. (Diptera: Culicidae) Populations from Delta State, Niger-Delta Region, Nigeria. *Journal of Medical Entomology* 2022; **59**(2): 758-63.
575. Lindsay SW, Adiamah JH, Miller JE, Armstrong JRM. Pyrethroid-treated bednet effects on mosquitoes of the Anopheles gambiae complex in The Gambia. *Medical and Veterinary Entomology* 1991; **5**(4): 477-83.
576. Qiu X, Awolola TS, Adeogun A, et al. Pyrethroids resistance intensity and resistance mechanisms in Anopheles gambiae from malaria vector surveillance sites in Nigeria. *Plos One* 2018; **13**(12).
577. Gillies MT, Wilkes TJ. The range of attraction of animal baits and carbon dioxide for mosquitoes. Studies in a freshwater area of West Africa. *Bull Entomol Res* 1972; **61**(3): 389-+.
578. Gillies MT, Wilkes TJ. The range of attraction of single baits for some West African mosquitoes. *Bull Entomol Res* 1974; **63**(4): 573-&.
579. Camara A, Loua Z, Loua É, et al. Rapid Epidemiological and Entomological Survey for Validation of Reported Indicators and Characterization of Local Malaria Transmission in Guinea, 2017. *The American Journal of Tropical Medicine and Hygiene* 2018; **99**(5): 1134-44.
580. Kalendar R, Chabi J, Van't Hof A, et al. Rapid high throughput SYBR green assay for identifying the malaria vectors Anopheles arabiensis, Anopheles coluzzii and Anopheles gambiae s.s. Giles. *Plos One* 2019; **14**(4).
581. Mouhamadou CS, N'Dri PB, Fodjo BK, Sadia CG, Affoue F-PK, Koudou BG. Rapid spread of double East- and West-African kdr mutations in wild Anopheles coluzzi from Côte d'Ivoire. *Wellcome Open Research* 2019; **4**.
582. Ba Y, Sall AA, Diallo D, et al. Re-Emergence of Rift Valley Fever Virus in Barkedji (Senegal, West Africa) in 2002–2003: Identification of New Vectors and Epidemiological Implications. *Journal of the American Mosquito Control Association* 2012; **28**(3): 170-8.
583. Ndione MHD, Ndiaye EH, Faye M, et al. Re-Introduction of West Nile Virus Lineage 1 in Senegal from Europe and Subsequent Circulation in Human and Mosquito Populations between 2012 and 2021. *Viruses* 2022; **14**(12).
584. Koffi AA, Ahoua Alou LP, Kabran JP, N'Guessan R, Pennetier C. Re-visiting insecticide resistance status in Anopheles gambiae from Côte d'Ivoire: a nation-wide informative survey. *PLoS One* 2013; **8**(12): e82387.
585. N'Guessan R, Corbel V, Akogbéto M, Rowland M. Reduced efficacy of insecticide-treated nets and indoor residual spraying for malaria control in pyrethroid resistance area, Benin. *Emerg Infect Dis* 2007; **13**(2): 199-206.
586. Thonnon J, Fontenille D, Tall A, et al. Reemergence of yellow fever in Senegal in 1995. *Am J Trop Med Hyg* 1998; **59**(1): 108-14.
587. Ebenezer A, Noutcha AE, Okiwelu SN. Relationship of annual entomological inoculation rates to malaria transmission indices, Bayelsa State, Nigeria. *J Vector Borne Dis* 2016; **53**(1): 46-53.
588. Costantini C, Sagnon NF, Sanogo E, Merzagora L, Coluzzi M. Relationship to human biting collections and influence of light and bednet in CDC light-trap catches of West African malaria vectors. *Bulletin of Entomological Research* 1998; **88**(5): 503-11.
589. Brooke B, Niang EHA, Konaté L, Diallo M, Faye O, Dia I. Reproductive Isolation among Sympatric Molecular Forms of An. gambiae from Inland Areas of South-Eastern Senegal. *PLoS ONE* 2014; **9**(8).
590. Ahadji-Dabla KM, Chabi J, Apetogbo YG, Koffi E, Hadi MP, Ketoh GK. Resistance intensity

status of Anopheles gambiae s.l. species at KOLOKOPE, eastern plateau Togo: A potential site to assess new vector control tools. *Heliyon* 2022; **8**(6).
591. Chukwuekezie O, Nwosu E, Nwangwu U, et al. Resistance status of Anopheles gambiae (s.l.) to four commonly used insecticides for malaria vector control in South-East Nigeria. *Parasites & Vectors* 2020; **13**(1).
592. Sovi A, Govoétchan R, Ossé R, et al. Resistance status of Anopheles gambiae s.l. to insecticides following the 2011 mass distribution campaign of long-lasting insecticidal nets (LLINs) in the Plateau Department, south-eastern Benin. *Malar J* 2020; **19**(1).
593. Lindsay SW, Shenton FC, Snow RW, Greenwood BM. Responses of Anopheles gambiae complex mosquitoes to the use of untreated bednets in The Gambia. *Med Vet Entomol* 1989; **3**(3): 253-62.
594. Gillies MT, Wilkes TJ. Responses of host-seeking Mansonia and Anopheles mosquitoes (Diptera: Culicidae) in West Africa to visual features of a target. *J Med Entomol* 1982; **19**(1): 68-71.
595. Diallo D, Diallo M. Resting behavior of Aedes aegypti in southeastern Senegal. *Parasites & Vectors* 2020; **13**(1).
596. Diouf B, Sene NM, Ndiaye EH, et al. Resting Behavior of Blood-Fed Females and Host Feeding Preferences of Aedes aegypti (Diptera: Culicidae) Morphological Forms in Senegal. *Journal of Medical Entomology* 2021; **58**(6): 2467-73.
597. Brady J. Results of age-grouping dissections on four species of Anopheles from southern Ghana. *Bull World Health Organ* 1963; **29**(2): 147-53.
598. Michel K, Socolovschi C, Pages F, Ndiath MO, Ratmanov P, Raoult D. Rickettsia Species in African Anopheles Mosquitoes. *PLoS ONE* 2012; **7**(10).
599. Wilson ML, Chapman LE, Hall DB, et al. Rift Valley fever in rural northern Senegal: Human risk factors and potential vectors. *American Journal of Tropical Medicine and Hygiene* 1994; **50**(6): 663-75.
600. Barry Y, Elbara A, Bollahi MA, et al. Rift Valley fever, Mauritania, 2020: Lessons from a one health approach. *One Health* 2022; **15**.
601. Fontenille D, Traorelamizana M, Zeller H, Mondo M, Diallo M, Digoutte JP. Short report: Rift Valley fever in western Africa: isolations from Aedes mosquitoes during an interepizootic period. *Am J Trop Med Hyg* 1995; **52**(5): 403-4.
602. Yaro JB, Tiono AB, Sanou A, et al. Risk factors associated with house entry of malaria vectors in an area of Burkina Faso with high, persistent malaria transmission and high insecticide resistance. *Malar J* 2021; **20**(1).
603. Kirby MJ, West P, Green C, Jasseh M, Lindsay SW. Risk factors for house-entry by culicine mosquitoes in a rural town and satellite villages in The Gambia. *Parasites & Vectors* 2008; **1**(1).
604. Kirby MJ, Green C, Milligan PM, et al. Risk factors for house-entry by malaria vectors in a rural town and satellite villages in The Gambia. *Malar J* 2008; **7**(1).
605. Fofana D, Beugré JMV, Yao-Acapovi GL, Lendzele SS. Risk of Dengue Transmission in Cocody (Abidjan, Ivory Coast). *Journal of Parasitology Research* 2019; **2019**: 1-7.
606. Suzuki T, Osei JH, Sasaki S, et al. Risk of transmission of viral haemorrhagic fevers and the insecticide susceptibility status of aedes aegypti (linnaeus) in some sites in Accra, Ghana. *Ghana Medical Journal* 2016; **50**(3): 136-41.
607. Hamer G, Chandre F, Poinsignon A, et al. Role of Anopheles gambiae s.s. and Anopheles coluzzii (Diptera: Culicidae) in Human Malaria Transmission in Rural Areas of Bouaké, in Côte d'Ivoire. *Journal of Medical Entomology* 2020; **57**(4): 1254-61.
608. Dadzie SK, Brenyah R, Appawu MA. Role of species composition in malaria transmission by the

Anopheles funestus group (Diptera: Culicidae) in Ghana. *J Vector Ecol* 2013; **38**(1): 105-10.
609. Krajacich BJ, LaBrecque B, Fakoli LS, et al. Sampling Host-Seeking Anthropophilic Mosquito Vectors in West Africa: Comparisons of an Active Human-Baited Tent-Trap Against Gold Standard Methods. *The American Journal of Tropical Medicine and Hygiene* 2015; **92**(2): 415-21.
610. Amusan AAS, Mafiana CF, Idowu AB, Olatunde GO. Sampling mosquitoes with CDC light trap in rice field and plantation communities in Ogun State, Nigeria. *Tanzania Journal of Health Research* 2006; **7**(3).
611. Tinto B, Kaboré DPA, Kagoné TS, et al. Screening of Circulation of Usutu and West Nile Viruses: A One Health Approach in Humans, Domestic Animals and Mosquitoes in Burkina Faso, West Africa. *Microorganisms* 2022; **10**(10).
612. Da Moura AJ, Valadas V, Da Veiga Leal S, Sousa CA, Pinto J. Screening of natural Wolbachia infection in mosquitoes (Diptera: Culicidae) from Cape Verde Islands. 2022.
613. Fagbohun IK, Idowu ET, Awolola TS, Otubanjo OA. Seasonal abundance and larval habitats characterization of mosquito species in Lagos State, Nigeria. *Scientific African* 2020; **10**.
614. Motayo BO, Onoja BA, Faneye AO, Adeniji JA. Seasonal abundance and molecular identification of West Nile virus vectors, Culex pipens and Culex quinquefasciatus (diptera: culicidae) in Abeokuta, South-West, Nigeria. *African Health Sciences* 2016; **16**(1).
615. Ould Lemrabott MA, Ould Ahmedou Salem MS, Ould Brahim K, et al. Seasonal abundance, blood meal sources and insecticide susceptibility in major anopheline malaria vectors from southern Mauritania. *Parasites & Vectors* 2018; **11**(1).
616. Govoetchan R, Sovi A, Ossè R, Akogbéto M. Seasonal change in species composition and target-site mutations in Anopheles gambiae s.l. in the severe drought area of Kandi, North-eastern Benin. *International Journal of Mosquito Research* 2022; **9**(6): 38-43.
617. Getz WM, Manoukis NC, Baber I, Diallo M, Sogoba N, Ribeiro JMC. Seasonal Climate Effects Anemotaxis in Newly Emerged Adult Anopheles gambiae Giles in Mali, West Africa. *PLoS ONE* 2011; **6**(11).
618. Epopa PS, Collins CM, North A, et al. Seasonal malaria vector and transmission dynamics in western Burkina Faso. *Malar J* 2019; **18**(1).
619. Bang YH, Knudsen AB, Onwubiko AO, Bown DN. Seasonal survival of AEDES africanus (Diptera: Culicidae) in Nigeria. *J Med Entomol* 1983; **20**(2): 128-33.
620. Ba O, Sow A, Ba H, Dahdi S, Lo B. [Seasonal transmission of malaria in the Senegal River Valley: case study of the city of Kaedi-Mauritanie]. *Pan Afr Med J* 2019; **34**: 185.
621. Guindo-Coulibaly N, Kpan MDS, Adja AM, et al. Seasonal variation and intra urban heterogeneity of the entomological risk of transmission of dengue and yellow fever in Abidjan, Côte d'Ivoire. *Medical and Veterinary Entomology* 2022; **36**(3): 329-37.
622. Huestis DL, Yaro AS, Traoré AI, et al. Seasonal variation in metabolic rate, flight activity and body size of Anopheles gambiae in the Sahel. *Journal of Experimental Biology* 2012; **215**(12): 2013-21.
623. Lehmann T, Dao A, Yaro AS, et al. Seasonal Variation in Spatial Distributions ofAnopheles gambiaein a Sahelian Village: Evidence for Aestivation. *Journal of Medical Entomology* 2014; **51**(1): 27-38.
624. Hidalgo K, Dujardin J-P, Mouline K, Dabiré RK, Renault D, Simard F. Seasonal variation in wing size and shape between geographic populations of the malaria vector, Anopheles coluzzii in Burkina Faso (West Africa). *Acta Tropica* 2015; **143**: 79-88.
625. Rishikesh N, Dideco MA, Petrarca V, Coluzzi M. Seasonal variations in indoor resting Anopheles

gambiae and Anopheles arabiensis in Kaduna, Nigeria. *Acta Trop* 1985; **42**(2): 165-70.
626. Moreira LA, Akorli J, Gendrin M, et al. Seasonality and Locality Affect the Diversity of Anopheles gambiae and Anopheles coluzzii Midgut Microbiota from Ghana. *Plos One* 2016; **11**(6).
627. Barreaux AMG, Oumbouke WA, Tia IZ, et al. Semi-field evaluation of the cumulative effects of a "Lethal House Lure" on malaria mosquito mortality. *Malar J* 2019; **18**(1).
628. Zahouli JZB, Dibo J-D, Diakaridia F, et al. Semi-field evaluation of the space spray efficacy of Fludora Co-Max EW against wild insecticide-resistant Aedes aegypti and Culex quinquefasciatus mosquito populations from Abidjan, Côte d'Ivoire. *Parasites & Vectors* 2023; **16**(1).
629. Barreaux AMG, Brou NG, Koffi AA, et al. Semi-field studies to better understand the impact of eave tubes on mosquito mortality and behaviour. *Malar J* 2018; **17**(1).
630. Irving-Bell RJ, Okoli EI, Diyelong DY, Lyimo EO, Onyia OC. Septic tank mosquitoes: competition between species in central Nigeria. *Med Vet Entomol* 1987; **1**(3): 243-50.
631. Atadiose EO, Kabir J, Adamu SG, Umoh JU. Serosurvey of West Nile virus in horses and detection of West Nile virus antigen in mosquitoes in Kaduna State, Nigeria. *J Equine Sci* 2020; **31**(3): 61-6.
632. Sougoufara S, Harry M, DoucourÉ S, SembÈNe PM, Sokhna C. Shift in species composition in the Anopheles gambiae complex after implementation of long-lasting insecticidal nets in Dielmo, Senegal. *Medical and Veterinary Entomology* 2016; **30**(3): 365-8.
633. Hien AS, Soma DD, Somé FA, et al. Short Persistence and Vector Susceptibility to Ficam 80WP (bendiocarb active ingredient) During Pilot Application of Indoor Residual Spraying in Burkina Faso, West Africa. *Journal of Medical Entomology* 2021; **58**(2): 781-6.
634. Oringanje C, Alaribe AAA, Oduola AO, et al. Short research communications:Vector abundance and species composition of Anopheles mosquito in Calabar, Nigeria. *Journal of Vector Borne Diseases* 2011; **48**(3): 171-3.
635. Ansell J, Hamilton KA, Pinder M, Walraven GEL, Lindsay SW. Short-range attractiveness of pregnant women to Anopheles gambiae mosquitoes. *Transactions of the Royal Society of Tropical Medicine and Hygiene* 2002; **96**(2): 113-6.
636. Richards FO, Pam DD, Kal A, et al. Significant decrease in the prevalence ofWuchereria bancroftiinfection in anopheline mosquitoes following the addition of albendazole to annual, ivermectin-based, mass treatments in Nigeria. *Annals of Tropical Medicine & Parasitology* 2013; **99**(2): 155-64.
637. Diatta M, Spiegel A, Lochouarn L, Fontenille D. Similar feeding preferences of Anopheles gambiae and A. arabiensis in Senegal. *Transactions of the Royal Society of Tropical Medicine and Hygiene* 1998; **92**(3): 270-2.
638. Akogbéto MC, Aïkpon RY, Azondékon R, et al. Six years of experience in entomological surveillance of indoor residual spraying against malaria transmission in Benin: lessons learned, challenges and outlooks. *Malar J* 2015; **14**(1).
639. Agossa FR, Padonou GG, Fassinou AJYH, et al. Small-scale field evaluation of the efficacy and residual effect of Fludora® Fusion (mixture of clothianidin and deltamethrin) against susceptible and resistant Anopheles gambiae populations from Benin, West Africa. *Malar J* 2018; **17**(1).
640. Boorman JP, Service MW. Some records of mosquitoes (Culicidae, Diptera) from the Niger Delta area, Southern Nigeria. *West Afr Med J* 1960; **9**: 67-72.
641. Edillo FE, Touré YT, Lanzaro GC, Dolo G, Taylor CE. Spatial and Habitat Distribution ofAnopheles gambiaeandAnopheles arabiensis(Diptera: Culicidae) in Banambani Village, Mali.

Journal of Medical Entomology 2002; **39**(1): 70-7.
642. Diakité NR, Guindo-Coulibaly N, Adja AM, et al. Spatial and temporal variation of malaria entomological parameters at the onset of a hydro-agricultural development in central Côte d'Ivoire. *Malar J* 2015; **14**(1).
643. Thabet HS, TagEldin RA, Fahmy NT, et al. Spatial Distribution of PCR-Identified Species of Anopheles gambiae senu lato (Diptera: Culicidae) Across Three Eco-Vegetational Zones in Cross River State, Nigeria. *Journal of Medical Entomology* 2022; **59**(2): 576-84.
644. Ebenezer A, Noutcha AE, Agi PI, Okiwelu SN, Commander T. Spatial distribution of the sibling species of Anopheles gambiae sensu lato (Diptera: Culicidae) and malaria prevalence in Bayelsa State, Nigeria. *Parasit Vectors* 2014; **7**: 32.
645. Kpan MDS, Adja AM, Guindo-Coulibaly N, et al. Spatial Heterogeneity and Seasonal Distribution of Aedes (Stegomyia) aegypti (L) in Abidjan, Côte d'Ivoire. *Vector-Borne and Zoonotic Diseases* 2021; **21**(10): 769-76.
646. Diabaté A, Dao A, Yaro AS, et al. Spatial swarm segregation and reproductive isolation between the molecular forms of
Anopheles gambiae. *Proceedings of the Royal Society B: Biological Sciences* 2009; **276**(1676): 4215-22.
647. Owusu-Asenso CM, Mingle JA, Weetmann D, Afrane YA. Spatio temporal distribution and insecticide resistance status of Aedes mosquitoes in Ghana. *American Journal of Tropical Medicine and Hygiene* 2019; **101**(5): 442.
648. Owusu-Asenso CM, Mingle JAA, Weetman D, Afrane YA. Spatiotemporal distribution and insecticide resistance status of Aedes aegypti in Ghana. *Parasites & Vectors* 2022; **15**(1).
649. Yawson AE, McCall PJ, Wilson MD, Donnelly MJ. Species abundance and insecticide resistance of Anopheles gambiae in selected areas of Ghana and Burkina Faso. *Med Vet Entomol* 2004; **18**(4): 372-7.
650. Nnanna A, Nwabueze O, Kosisochukwu U, Benedicta r, Amauche I. Species Composition and Infection Rate of Mosquito Vectors Following the Indoor Residual Spraying Exercise in three Communities in Awka North L.G.A of Anambra State, Nigeria. *Annual Research & Review in Biology* 2016; **10**(2): 1-8.
651. Hwang J-S, Joannides J, Dzodzomenyo M, et al. Species composition and risk of transmission of some Aedes-borne arboviruses in some sites in Northern Ghana. *Plos One* 2021; **16**(6).
652. Okorie PN, Popoola KO, Awobifa OM, Ibrahim KT, Ademowo GO. Species composition and temporal distribution of mosquito populations in Ibadan, Southwestern Nigeria. *J Entomol Zool Stud* 2014; **2**(4): 164-9.
653. Oduwole OA, Oringanje CM, Oduola AO, et al. Species Composition of Anopheles (Diptera: Culicidae) in Selected Forested Tourist Areas of Nigeria Endemic for Malaria. *Journal of Medical Entomology* 2020; **57**(6): 2007-10.
654. Keita K, Camara D, Barry Y, et al. Species Identification and Resistance Status of Anopheles gambiae s.l. (Diptera: Culicidae) Mosquitoes in Guinea. *Journal of Medical Entomology* 2017; **54**(3): 677-81.
655. Fofana D, Konan KL, Djohan V, et al. [Specific diversity and culicidian nuisance in the villages of N'gatty and Allaba in laguna area of Ivory Coast]. *Bull Soc Pathol Exot* 2010; **103**(5): 333-9.
656. Opondo KO, Jawara M, Cham S, et al. Status of insecticide resistance in Anopheles gambiae (s.l.) of The Gambia. *Parasites & Vectors* 2019; **12**(1).

657. Betson M, Jawara M, Awolola TS. Status of insecticide susceptibility in Anopheles gambiae s.l. from malaria surveillance sites in The Gambia. *Malar J* 2009; **8**(1).
658. Aïzoun N, Aïkpon R, Gnanguenon V, et al. Status of organophosphate and carbamate resistance in Anopheles gambiae sensu lato from the south and north Benin, West Africa. *Parasit Vectors* 2013; **6**: 274.
659. Ahoua Alou LP, Koffi AA, Adja MA, Assi SB, Kouassi PK, N'Guessan R. Status of pyrethroid resistance in Anopheles gambiae s. s. M form prior to the scaling up of Long Lasting Insecticidal Nets (LLINs) in Adzopé, Eastern Côte d'Ivoire. *Parasit Vectors* 2012; **5**: 289.
660. Manoukis NC, Diabate A, Abdoulaye A, et al. Structure and Dynamics of Male Swarms ofAnopheles gambiae. *Journal of Medical Entomology* 2009; **46**(2): 227-35.
661. Ramsdale CD, Leport GH. STUDIES OF ANOPHELES GAMBIAE COMPLEX IN WEST AFRICA. *Bull World Health Organ* 1967; **36**(3): 494-&.
662. Amuzu H, Wilson MD, Boakye DA. Studies of Anopheles gambiae s.l (Diptera: Culicidae) exhibiting different vectorial capacities in lymphatic filariasis transmission in the Gomoa district, Ghana. *Parasit Vectors* 2010; **3**: 85.
663. Snow WF. Studies of house-entering habits of mosquitoes in The Gambia, West Africa: experiments with prefabricated huts with varied wall apertures. *Med Vet Entomol* 1987; **1**(1): 9-21.
664. Thomson RCM. Studies on Anopheles gambiae and Anopheles melas in and around Lagos. *Bull Entomol Res* 1948; **38**(4): 527-+.
665. Majori G, Sabatinelli G, Villani F, Petrarca V. Studies on insecticide susceptibility of Anopheles gambiae s.1. and Culex quinquefasciatus in the area of Ouagadougou, Burkina Faso (west Africa). *J Am Mosq Control Assoc*1986; **2**(3): 305-9.
666. Adebote DA, Oniye SJ, Muhammed YA. Studies on mosquitoes breeding in rock pools on inselbergs around Zaria, northern Nigeria. *J Vector Borne Dis* 2008; **45**(1): 21-8.
667. Boreham PFL, Lenahan JK, Boulzaguet R, et al. Studies on multiple feeding by anopheles gambae s.l. in a sudan savanna area of north nigeria. *Transactions of the Royal Society of Tropical Medicine and Hygiene* 1979; **73**(4): 418-23.
668. Service MW. Studies on sampling larval populations of the Anopheles gambiae complex. *Bull World Health Organ* 1971; **45**(2): 169-+.
669. Adebote DA, Abolude DS, Oniye SJ, Wayas OS. Studies on Some Physicochemical Factors Affecting the Breeding and Abundance of Mosquitoes (Diptera: Culicidae) in Phytotelmata on Delonix regia (Leguminosae: Caesalpinoidea). *Journal of Biological Sciences* 2008; **8**(8): 1304-9.
670. Charlwood JD, Tomás EVE, Salgueiro P, Egyir-Yawson A, Pitts RJ, Pinto J. Studies on the behaviour of peridomestic and endophagic M form Anopheles gambiae from a rice growing area of Ghana. *Bulletin of Entomological Research* 2011; **101**(5): 533-9.
671. Service MW. Studies on the biology and taxonomy of Aedes (Stegomyia) vittatus (Bigot) (Diptera: Culicidae) in Northern Nigeria. *Ecol Entomol* 1970; **122**(4): 101-43.
672. Thomson RCM. Studies on the Breeding Places and Control of *Anopheles gambiae* and *A. gambiae* var. *mêlas* in coastal Districts of Sierra Leone. *Bull Entomol Res* 1945; **36**(2): 185-+.
673. Aïzoun N, Azondekon R, Aïkpon R, et al. Study of the efficacy of a Wheaton coated bottle with permethrin and deltamethrin in laboratory conditions and a WHO impregnated paper with bendiocarb in field conditions. *Asian Pacific Journal of Tropical Biomedicine* 2014; **4**(6): 492-7.
674. Campos M, Ward D, Morales RF, et al. Surveillance of Aedes aegypti populations in the city of Praia, Cape Verde: Zika virus infection, insecticide resistance and genetic diversity. *Parasites &*

Vectors 2020; **13**(1).
675. Tchibozo C, Hounkanrin G, Yadouleton A, et al. Surveillance of arthropod-borne viruses in Benin, West Africa 2020–2021: detection of dengue virus 3 in Aedes aegypti (Diptera: Culicidae). *Military Medical Research* 2022; **9**(1).
676. Service MW. Survey of the relative prevalence of potential yellow fever vectors in north-west Nigeria. *Bull World Health Organ* 1974; **50**(6): 487-94.
677. Ouattara LPE, Sangaré I, Namountougou M, et al. Surveys of Arboviruses Vectors in Four Cities Stretching Along a Railway Transect of Burkina Faso: Risk Transmission and Insecticide Susceptibility Status of Potential Vectors. *Frontiers in Veterinary Science* 2019; **6**.
678. Edillo FE, Touré YT, Lanzaro GC, Dolo G, Taylor CE. Survivorship and Distribution of ImmatureAnopheles gambiaes.l. (Diptera: Culicidae) in Banambani Village, Mali. *Journal of Medical Entomology* 2004; **41**(3): 333-9.
679. Olayemi IK, Ande AT. Survivorship of anopheles gambiae in relation to malaria transmission in Ilorin, Nigeria. *Online J Health Allied Sci* 2008; **7**(3).
680. Kouassi BL, Edi C, Tia E, et al. Susceptibility of Anopheles gambiae from Côte d'Ivoire to insecticides used on insecticide-treated nets: evaluating the additional entomological impact of piperonyl butoxide and chlorfenapyr. *Malar J* 2020; **19**(1).
681. Egyir N, Lawson B, Desewu K, Ampofo-Bekoe R, Kumalo H, Amoako D. Susceptibility of Anopheles Mosquito to Agricultural Insecticides in the Adansi North District, Ghana. *Journal of Pure and Applied Microbiology* 2019; **13**(2): 677-88.
682. Umar A, Kabir BGJ, Amajoh CN, et al. Susceptibility test of female anopheles mosquitoes to ten insecticides for indoor residual spraying (IRS) baseline data collection in Northeastern Nigeria. *Journal of Entomology and Nematology* 2014; **6**(7): 98-103.
683. Adasi K, Hemingway J. Susceptibility to three pyrethroids and detection of knockdown resistance mutation in Ghanaian Anopheles gambiaesensu stricto. *Journal of Vector Ecology* 2008; **33**(2): 255-62.
684. Sawadogo PS, Namountougou M, Toé KH, et al. Swarming behaviour in natural populations of Anopheles gambiae and An. coluzzii: Review of 4 years survey in rural areas of sympatry, Burkina Faso (West Africa). *Acta Tropica* 2014; **132**: S42-S52.
685. Williams J, Ingham VA, Morris M, et al. Sympatric Populations of the Anopheles gambiae Complex in Southwest Burkina Faso Evolve Multiple Diverse Resistance Mechanisms in Response to Intense Selection Pressure with Pyrethroids. *Insects* 2022; **13**(3).
686. Platt N, Kwiatkowska RM, Irving H, Diabaté A, Dabire R, Wondji CS. Target-site resistance mutations (kdr and RDL), but not metabolic resistance, negatively impact male mating competiveness in the malaria vector Anopheles gambiae. *Heredity* 2015; **115**(3): 243-52.
687. E.B L, L.E Da, G.D G. Taxonomy of Anopheles Mosquitoes in Ganawuri Chiefdom, Riyom Local Government Area, Plateau State, Nigeria. *International Journal of Zoological Investigations* 2021; **7**(2): 519-26.
688. Byrne I, Chan K, Manrique E, et al. Technical Workflow Development for Integrating Drone Surveys and Entomological Sampling to Characterise Aquatic Larval Habitats of Anopheles funestus in Agricultural Landscapes in Côte d'Ivoire. *Journal of Environmental and Public Health* 2021; **2021**: 1-14.
689. Diallo D, Talla C, Ba Y, Dia I, Sall AA, Diallo M. Temporal distribution and spatial pattern of abundance of the Rift Valley fever and West Nile fever vectors in Barkedji, Senegal. *J Vector Ecol* 2011; **36**(2): 426-36.

690. Aigbodion FI, Uyi OO. Temporal Distribution of and Habitat Diversification by Some Mosquitoes (Diptera: Culicidae) Species in Benin City, Nigeria. *Journal of Entomology* 2012; **10**(1): 13-23.
691. Edith I-S, Alfred TB, N′falé S, Nadine CO, Issa N, B SS. Temporal Dynamics of Malaria Transmission in Two Rural Areas of Burkina Faso With Two Ecological Differences. *Journal of Medical Entomology* 2010; **47**(4): 618-24.
692. Ibrahim SS, Mukhtar MM, Datti JA, et al. Temporal escalation of Pyrethroid Resistance in the major malaria vector Anopheles coluzzii from Sahelo-Sudanian Region of northern Nigeria. *Scientific Reports* 2019; **9**(1).
693. Mukhtar MM, Ibrahim SS. Temporal Evaluation of Insecticide Resistance in Populations of the Major Arboviral Vector Aedes Aegypti from Northern Nigeria. *Insects* 2022; **13**(2).
694. Adeogun AO, Brooke BD, Olayanju DR, et al. Test for association between dieldrin resistance and 2La inversion polymorphism in Anopheles coluzzii from Lagos, Nigeria. *Trop Biomed* 2019; **36**(3): 587-93.
695. Diarra RA, Traore MM, Junnila A, et al. Testing configurations of attractive toxic sugar bait (ATSB) stations in Mali, West Africa, for improving the control of malaria parasite transmission by vector mosquitoes and minimizing their effect on non-target insects. *Malar J* 2021; **20**(1).
696. Snow WF. The attractiveness of some birds and mammals for mosquitoes in The Gambia, West Africa. *Annals of Tropical Medicine & Parasitology* 2016; **77**(6): 641-51.
697. Service MW. The behaviour of malaria vectors in huts sprayed with DDT and with a mixture of DDT and malathion in Northern Nigeria. *Transactions of the Royal Society of Tropical Medicine and Hygiene* 1964; **58**(1): 72-9.
698. Adebote AD, Oniye JS, Ndams SI, Nache KM. The Breeding of Mosquitoes (Diptera: Culicidae) in Peridomestic Containers and Implication in Yellow Fever Transmission in Villages Around Zaria, Northern Nigeria. *Journal of Entomology* 2006; **3**(2): 180-8.
699. Koukpo CZ, Fassinou AJYH, Ossè RA, et al. The current distribution and characterization of the L1014F resistance allele of the kdr gene in three malaria vectors (Anopheles gambiae, Anopheles coluzzii, Anopheles arabiensis) in Benin (West Africa). *Malar J* 2019; **18**(1).
700. Zoh DD, Ahoua Alou LP, Toure M, et al. The current insecticide resistance status of Anopheles gambiae (s.l.) (Culicidae) in rural and urban areas of Bouaké, Côte d'Ivoire. *Parasites & Vectors* 2018; **11**(1).
701. Snow WF. The direction of flight of mosquitoes (Diptera, Culicidae) near the ground in West African savanna in relation to wind direction, in the presence and absence of bait. *Bulletin of Entomological Research* 1976; **65**(4): 555-62.
702. Boreham PFL, Port GR. The distribution and movement of engorged females of Anopheles gambiae Giles (Diptera: Culicidae) in a Gambian village. *Bulletin of Entomological Research* 1982; **72**(3): 489-95.
703. Taylor AW. The Domestic Mosquitos of Gadau, Northern Nigeria, and Their Relation to Malaria and Filariasis. *Annals of Tropical Medicine & Parasitology* 2016; **24**(3): 425-35.
704. Afrane YA, Lawson BW, Brenya R, Kruppa T, Yan G. The ecology of mosquitoes in an irrigated vegetable farm in Kumasi, Ghana: abundance, productivity and survivorship. *Parasit Vectors* 2012; **5**: 233.
705. Service MW. The ecology of the mosquitos of the Northern Guinea Savannah of Nigeria. *Bulletin of Entomological Research* 2009; **54**(3): 601-32.
706. Shiff C, Maia MF, Abonuusum A, et al. The Effect of Deltamethrin-treated Net Fencing around

Cattle Enclosures on Outdoor-biting Mosquitoes in Kumasi, Ghana. *PLoS ONE* 2012; **7**(9).
707. Lindsay SW, Adiamah JH, Armstrong JRM. The effect of permethrin-impregnated bednets on house entry by mosquitoes (Diptera: Culicidae) in The Gambia. *Bulletin of Entomological Research* 2009; **82**(1): 49-55.
708. Norris D, Lehmann T, Faiman R, et al. The Effects of High-Altitude Windborne Migration on Survival, Oviposition, and Blood-Feeding of the African Malaria Mosquito, Anopheles gambiae s.l. (Diptera: Culicidae). *Journal of Medical Entomology* 2020.
709. Thomson RC. The effects of house spraying with pyrethrum and with DDT on Anopheles gambiae and A. melas in West Africa. *Bull Entomol Res* 1947; **38**(3): 449-64.
710. Snow WF. The height and direction of flight of mosquitoes in West African savanna, in relation to wind speed and direction. *Bulletin of Entomological Research* 1977; **67**(2): 271-9.
711. Snow WF, Boreham PFL. The host-feeding patterns of some culicine mosquitoes (Diptera: Culicidae) in The Gambia. *Bulletin of Entomological Research* 2009; **68**(4): 695-706.
712. Sougoufara S, Thiaw O, Cailleau A, et al. The Impact of Periodic Distribution Campaigns of Long-Lasting Insecticidal-Treated Bed Nets on Malaria Vector Dynamics and Human Exposure in Dielmo, Senegal. *The American Journal of Tropical Medicine and Hygiene* 2018; **98**(5): 1343-52.
713. Coulibaly YI, Nutman TB, Soumaoro L, et al. The Impact of Six Annual Rounds of Mass Drug Administration on Wuchereria bancrofti Infections in Humans and in Mosquitoes in Mali. *The American Journal of Tropical Medicine and Hygiene* 2015; **93**(2): 356-60.
714. Yadouléton A, N'Guessan R, Allagbé H, et al. The impact of the expansion of urban vegetable farming on malaria transmission in major cities of Benin. *Parasit Vectors* 2010; **3**: 118.
715. Erlank E, Koekemoer LL, Coetzee M. The importance of morphological identification of African anopheline mosquitoes (Diptera: Culicidae) for malaria control programmes. *Malar J* 2018; **17**(1).
716. Anyanwu IN, Agbede RI, Ajanusi OJ, Umoh JU, Ibrahim ND. The incrimination of Aedes (stegomyia) aegypti as the vector of Dirofilaria repens in Nigeria. *Vet Parasitol* 2000; **92**(4): 319-27.
717. Muller GC, Junnila A, Traore MM, et al. The invasive shrub Prosopis juliflora enhances the malaria parasite transmission capacity of Anopheles mosquitoes: a habitat manipulation experiment. *Malar J* 2017; **16**(1).
718. Lewis DJ. The Medical Entomology of the Tonkolili Valley, Sierra Leone. *Annals of Tropical Medicine & Parasitology* 2016; **50**(3): 299-313.
719. Fall AG, DiaÏTÉ A, Etter E, Bouyer J, Ndiaye TD, KonatÉ L. The mosquito Aedes (Aedimorphus) vexans arabiensis as a probable vector bridging the West Nile virus between birds and horses in Barkedji (Ferlo, Senegal). *Medical and Veterinary Entomology* 2012; **26**(1): 106-11.
720. Peters W. THE MOSQUITOES OF LIBERIA (DIPTERA: CULICIDAE). *Syst Entomol* 1955; **24**(5-6): 81-90.
721. Hanney PW. The mosquitos of Zaria Province, Northern Nigeria. *Bulletin of Entomological Research* 1960; **51**(1): 145-71.
722. Campos M, Hanemaaijer M, Gripkey H, et al. The origin of island populations of the African malaria mosquito, Anopheles coluzzii. *Communications Biology* 2021; **4**(1).
723. Pålsson K, Pinto J, do Rosario VE, Jaenson TGT. The palpal ratio method compared with PCR to distinguish between Anopheles gambiae s.s. and A. melas from Guinea Bissau, West Africa. *Acta Tropica* 1998; **70**(1): 101-7.
724. Toé KH, N'Falé S, Dabiré RK, Ranson H, Jones CM. The recent escalation in strength of pyrethroid resistance in Anopheles coluzzi in West Africa is linked to increased expression of multiple

gene families. *BMC Genomics* 2015; **16**(1).
725. Carrasco-Tenezaca M, Jawara M, Abdi MY, et al. The relationship between house height and mosquito house entry: an experimental study in rural Gambia. *Journal of The Royal Society Interface* 2021; **18**(178).
726. Port GR, Boreham PFL, Bryan JH. The relationship of host size to feeding by mosquitoes of the Anopheles gambiae Giles complex (Diptera: Culicidae). *Bulletin of Entomological Research* 2009; **70**(1): 133-44.
727. Service MW. The replacement of Culex nebulosus Theo. by Culex pipiens fatigans Wied. (Diptera, Culicidae) in towns in Nigeria. *Bull Entomol Res* 1966; **56**(3): 407-15.
728. Gibson G, Costantini C, Sagnon F, Torre AD, Coluzzi M. The responses of Anopheles gambiae, and other mosquitoes in Burkina Faso, to CO2—the start of a search for synthetic human odour. *Annals of Tropical Medicine & Parasitology* 2016; **91**(sup1): S123-S4.
729. Forson AO, Hinne IA, Dhikrullahi SB, et al. The resting behavior of malaria vectors in different ecological zones of Ghana and its implications for vector control. *Parasites & Vectors* 2022; **15**(1).
730. Trape J-F, Tall A, Sokhna C, et al. The rise and fall of malaria in a west African rural community, Dielmo, Senegal, from 1990 to 2012: a 22 year longitudinal study. *The Lancet Infectious Diseases* 2014; **14**(6): 476-88.
731. King SA, Onayifeke B, Akorli J, et al. The Role of Detoxification Enzymes in the Adaptation of the Major Malaria Vector Anopheles gambiae (Giles; Diptera: Culicidae) to Polluted Water. *Journal of Medical Entomology* 2017; **54**(6): 1674-83.
732. Barreaux AMG, Oumbouke WA, Brou NG, et al. The role of human and mosquito behaviour in the efficacy of a house-based intervention. *Philosophical Transactions of the Royal Society B: Biological Sciences* 2020; **376**(1818).
733. Fahmy NT, Villinski JT, Bolay F, et al. The Seasonality and Ecology of the Anopheles gambiae complex (Dipetra: Culicidae) in Liberia Using Molecular Identification. *Journal of Medical Entomology* 2015; **52**(3): 475-82.
734. Pombi M, Guelbeogo WM, Kreppel K, et al. The Sticky Resting Box, a new tool for studying resting behaviour of Afrotropical malaria vectors. *Parasit Vectors* 2014; **7**: 247.
735. Ramakrishna V, Elliott R. The vectorial capacity of an insecticide-resistant strain of A. gambiae. *The West African medical journal* 1960; **9**: 105-10.
736. Gillies MT, Wilkes TJ. The vertical distribution of mosquitoes flying over open farmland in the Gambia. *Trans R Soc Trop Med Hyg* 1974; **68**(4): 268-9.
737. Gillies MT, Wilkes TJ. The vertical distribution of some West African mosquitoes (Diptera, Culicidae) over open farmland in a freshwater area of the Gambia. *Bulletin of Entomological Research* 2009; **66**(1): 5-15.
738. Snow WE, Wilkes TJ. The vertical distribution, and age, of mosquito populations in West African savanna. *Transactions of the Royal Society of Tropical Medicine and Hygiene* 1972; **66**(4): 536-7.
739. Badolo A, Traore A, Jones CM, et al. Three years of insecticide resistance monitoring in Anopheles gambiae in Burkina Faso: resistance on the rise? *Malar J* 2012; **11**: 232.
740. Wagbatsoma VA, Ogbeide O. Towards malaria control in Nigeria: a qualitative study on the population of mosquitoes. *J R Soc Health* 1995; **115**(6): 363-5.
741. Aboaba S, Ibrahim K, Omotoso O. Toxicity and mosquito larvicidal activities of the essential oils from the leaves of Acalypha ornata and Acalypha ciliata in southwest Nigeria. *J Vector Borne Dis* 2012; **49**(2): 114-6.

742. Favia G, Bilgo E, Lovett B, et al. Transgenic Metarhizium pingshaense synergistically ameliorates pyrethroid-resistance in wild-caught, malaria-vector mosquitoes. *Plos One* 2018; **13**(9).
743. Okwa OO, Akinmolayan FI, Carter V, Hurd H. Transmission dynamics of malaria in four selected ecological zones of Nigeria in the rainy season. *Ann Afr Med* 2009; **8**(1): 1-9.
744. Aboagye-Antwi F, Kwansa-Bentum B, Dadzie SK, et al. Transmission indices and microfilariae prevalence in human population prior to mass drug administration with ivermectin and albendazole in the Gomoa District of Ghana. *Parasites & Vectors* 2015; **8**(1).
745. Arez AP, Do RosÁRio VE, PÅLsson K, Jaenson TGT, Snounou G, Pinto J. Transmission of Mixed Plasmodium Species and Plasmodium Falciparum Genotypes. *The American Journal of Tropical Medicine and Hygiene* 2003; **68**(2): 161-8.
746. Gnanguenon V, Govoetchan R, Agossa FR, et al. Transmission patterns of Plasmodium falciparum by Anopheles gambiae in Benin. *Malar J* 2014; **13**: 444.
747. Service MW. Trials with dichlorvos (DDVP) against malaia vectors in huts in Northern Nigeria. *Transactions of the Royal Society of Tropical Medicine and Hygiene* 1965; **59**(2): 153-62.
748. Service MW. Two albinoid females of Anopheles gambiae Giles (Diptera: Culicidae) from Northern Nigeria. *Syst Entomol* 1964; **33**(5-6): 101-2.
749. Soma DD, Kassié D, Sanou S, et al. Uneven malaria transmission in geographically distinct districts of Bobo-Dioulasso, Burkina Faso. *Parasites & Vectors* 2018; **11**(1).
750. Koffi AA, Alou LP, Adja MA, Koné M, Chandre F, N'Guessan R. Update on resistance status of Anopheles gambiae s.s. to conventional insecticides at a previous WHOPES field site, "Yaokoffikro", 6 years after the political crisis in Côte d'Ivoire. *Parasit Vectors* 2012; **5**: 68.
751. Faye O, Ba Y, Faye O, et al. Urban Epidemic of Dengue Virus Serotype 3 Infection, Senegal, 2009. *Emerging Infectious Diseases* 2014; **20**(3).
752. Matthys B, Vounatsou P, Raso G, et al. Urban farming and malaria risk factors in a medium-sized town in Cote d'Ivoire. *Am J Trop Med Hyg* 2006; **75**(6): 1223-31.
753. Brieger WR, Sesay HR, Adesina H, et al. Urban malaria treatment behaviour in the context of low levels of malaria transmission in Lagos, Nigeria. *Afr J Med Med Sci* 2001; **30 Suppl**: 7-15.
754. Chen C-C, Zahouli JBZ, Koudou BG, et al. Urbanization is a main driver for the larval ecology of Aedes mosquitoes in arbovirus-endemic settings in south-eastern Côte d'Ivoire. *PLOS Neglected Tropical Diseases* 2017; **11**(7).
755. Nartey R, Owusu-Dabo E, Kruppa T, et al. Use of Bacillus thuringiensis var israelensis as a viable option in an Integrated Malaria Vector Control Programme in the Kumasi Metropolis, Ghana. *Parasit Vectors* 2013; **6**: 116.
756. Diallo D, Ba Y, Dia I, Lassana K, Diallo M. [Use of insecticide-treated cattle to control Rift Valley fever and West Nile virus vectors in Senegal]. *Bull Soc Pathol Exot* 2008; **101**(5): 410-7.
757. Tandina F, Niaré S, Laroche M, et al. Using MALDI-TOF MS to identify mosquitoes collected in Mali and their blood meals. *Parasitology* 2018; **145**(9): 1170-82.
758. Yé Y, Sauerborn R, Séraphin S, Hoshen M. Using modelling to assess the risk of malarial infection during the dry season, on a local scale in an endemic area of rural Burkina Faso. *Annals of Tropical Medicine & Parasitology* 2013; **101**(5): 375-89.
759. Lindsay SW, Adiamah JH, Miller JE, Pleass RJ, Armstrong JR. Variation in attractiveness of human subjects to malaria mosquitoes (Diptera: Culicidae) in The Gambia. *J Med Entomol* 1993; **30**(2): 368-73.
760. Huestis DL, Yaro AS, Traoré AI, et al. Variation in metabolic rate ofAnopheles gambiaeandA.

arabiensisin a Sahelian village. *Journal of Experimental Biology* 2011; **214**(14): 2345-53.
761. Diuk-Wasser MA, Toure MB, Dolo G, et al. Vector abundance and malaria transmission in rice-growing villages in Mali. *Am J Trop Med Hyg* 2005; **72**(6): 725-31.
762. Oringanje C, Alaribe AA, Oduola AO, et al. Vector abundance and species composition of Anopheles mosquito in Calabar, Nigeria. *J Vector Borne Dis* 2011; **48**(3): 171-3.
763. Weaver SC, Dickson LB, Sanchez-Vargas I, Sylla M, Fleming K, Black WC. Vector Competence in West African Aedes aegypti Is Flavivirus Species and Genotype Dependent. *PLoS Neglected Tropical Diseases* 2014; **8**(10).
764. Diagne CT, Weaver SC, Dia I, et al. Vector Competence of Aedes aegypti and Aedes vittatus (Diptera: Culicidae) from Senegal and Cape Verde Archipelago for West African Lineages of Chikungunya Virus. *The American Journal of Tropical Medicine and Hygiene* 2014; **91**(3): 635-41.
765. Ndiaye EH, Fall G, Gaye A, et al. Vector competence of Aedes vexans (Meigen), Culex poicilipes (Theobald) and Cx. quinquefasciatus Say from Senegal for West and East African lineages of Rift Valley fever virus. *Parasites & Vectors* 2016; **9**(1).
766. Gaye A, Diagne MM, Ndiaye EH, et al. Vector competence of anthropophilic mosquitoes for a new mesonivirus in Senegal. *Emerging Microbes & Infections* 2020; **9**(1): 496-504.
767. Boakye DA, Wilson MD, Appawu MA, Gyapong J. Vector competence, forWuchereria bancrofti, of theAnophelespopulations in the Bongo district of Ghana. *Annals of Tropical Medicine & Parasitology* 2013; **98**(5): 501-8.
768. Trape J-F, Salem G, Bouganali H, et al. Vector Density Gradients and the Epidemiology of Urban Malaria in Dakar, Senegal. *The American Journal of Tropical Medicine and Hygiene* 1992; **47**(2): 181-9.
769. Norris D, Yapi A, Poinsignon A, et al. Vectorial Transmission of Malaria in Major Districts of Côte d'Ivoire. *Journal of Medical Entomology* 2020; **57**(3): 908-14.
770. Diallo M, Thonnon J, Traore-Lamizana M, Fontenille D. Vectors of Chikungunya virus in Senegal: current data and transmission cycles. *Am J Trop Med Hyg* 1999; **60**(2): 281-6.
771. Lee VH, Moore DL. Vectors of the 1969 yellow fever epidemic on the Jos Plateau, Nigeria. *Bull World Health Organ* 1972; **46**(5): 669-73.
772. Govoetchan R, Fongnikin A, Syme T, et al. VECTRON™ T500, a new broflanilide insecticide for indoor residual spraying, provides prolonged control of pyrethroid-resistant malaria vectors. *Malar J* 2022; **21**(1).
773. Miller JE, Lindsay SW, Armstrong JRM, et al. Village trial of bednets impregnated with wash-resistant permethrin compared with other pyrethroid formulations. *Medical and Veterinary Entomology* 1995; **9**(1): 43-9.
774. Tchigossou GM, Akoton R, Yessoufou A, et al. Water source most suitable for rearing a sensitive malaria vector, Anopheles funestus in the laboratory. *Wellcome Open Research* 2017; **2**.
775. Cuzin-Ouattara N, Van den Broek AH, Habluetzel A, et al. Wide-scale installation of insecticide-treated curtains confers high levels of protection against malaria transmission in a hyperendemic area of Burkina Faso. *Trans R Soc Trop Med Hyg* 1999; **93**(5): 473-9.
776. Olatunbosun-Oduola A, Abba E, Adelaja O, Taiwo-Ande A, Poloma-Yoriyo K, Samson-Awolola T. Widespread Report of Multiple Insecticide Resistance in Anopheles gambiae s.l. Mos-quitoes in Eight Communities in Southern Gombe, North-Eastern Nigeria. *Journal of Arthropod-Borne Diseases* 2019.
777. Sow A, Faye O, Ba Y, et al. Widespread Rift Valley Fever Emergence in Senegal in 2013–2014. *Open Forum Infectious Diseases* 2016; **3**(3).

778. Michel K, Xu J, Hillyer JF, et al. Wild Anopheles funestus Mosquito Genotypes Are Permissive for Infection with the Rodent Malaria Parasite, Plasmodium berghei. *PLoS ONE* 2013; **8**(4).
779. Shaw WR, Marcenac P, Childs LM, et al. Wolbachia infections in natural Anopheles populations affect egg laying and negatively correlate with Plasmodium development. *Nature Communications* 2016; **7**(1).
780. Dabiré KR, Diabaté A, Paré-Toé L, et al. Year to year and seasonal variations in vector bionomics and malaria transmission in a humid savannah village in west Burkina Faso. *Journal of Vector Ecology* 2008; **33**(1): 70-5.
781. Germain M, Francy DB, Monath TP. Yellow fever in the Gambia, 1978-1979: Entomological aspects and epidemiological correlations. *American Journal of Tropical Medicine and Hygiene* 1980; **29**(5): I.
782. Attoui H, Diallo D, Sall AA, et al. Zika Virus Emergence in Mosquitoes in Southeastern Senegal, 2011. *PLoS ONE* 2014; **9**(10).
783. Diouf B, Gaye A, Diagne CT, Diallo M, Diallo D. Zika virus in southeastern Senegal: survival of the vectors and the virus during the dry season. *BMC Infectious Diseases* 2020; **20**(1).
784. Konaté L, Faye O, Gaye O, et al. [Zoophagia and alternative host selection of the marlaia vectors in Senegal]. *Parasite* 1999; **6**(3): 259-67.
785. Müller GC, Tsabari O, Traore MM, et al. First record of Aedes albopictus in inland Africa along the River Niger in Bamako and Mopti, Mali. *Acta Trop.* 2016;**162**:245-247.

Supplementary Table 3. Characteristics of environmental data used for ecological niche modeling

Category	Indicator	Description	Data source	Resolution used in the model
Climate—temperature	Bio1	Annual mean temperature (℃)	WorldClim database version 2.1 (https://worldclim.org/)	2.5 arc-minutes (approximately 5 km at the equator)
Climate—temperature	Bio2	Mean diurnal range (Mean of monthly (max temp-min temp)) (℃)	WorldClim database version 2.1 (https://worldclim.org/)	2.5 arc-minutes (approximately 5 km at the equator)
Climate—temperature	Bio3	Isothermality (Bio2/Bio7) (*100)	WorldClim database version 2.1 (https://worldclim.org/)	2.5 arc-minutes (approximately 5 km at the equator)
Climate—temperature	Bio4	Temperature Seasonality (standard deviation ×100)	WorldClim database version 2.1 (https://worldclim.org/)	2.5 arc-minutes (approximately 5 km at the equator)
Climate—temperature	Bio5	Max Temperature of Warmest Month (℃)	WorldClim database version 2.1 (https://worldclim.org/)	2.5 arc-minutes (approximately 5 km at the equator)
Climate—temperature	Bio6	Min Temperature of Coldest Month (℃)	WorldClim database version 2.1 (https://worldclim.org/)	2.5 arc-minutes (approximately 5 km at the equator)
Climate—temperature	Bio7	Temperature Annual Range (Bio5-Bio6) (℃)	WorldClim database version 2.1 (https://worldclim.org/)	2.5 arc-minutes (approximately 5 km at the equator)
Climate—temperature	Bio8	Mean Temperature of Wettest Quarter (℃)	WorldClim database version 2.1 (https://worldclim.org/)	2.5 arc-minutes (approximately 5 km at the equator)
Climate—temperature	Bio9	Mean Temperature of Driest Quarter (℃)	WorldClim database version 2.1 (https://worldclim.org/)	2.5 arc-minutes (approximately 5 km at the equator)
Climate—temperature	Bio10	Mean Temperature of Warmest Quarter (℃)	WorldClim database version 2.1 (https://worldclim.org/)	2.5 arc-minutes (approximately 5 km at the equator)
Climate—temperature	Bio11	Mean Temperature of Coldest Quarter (℃)	WorldClim database version 2.1 (https://worldclim.org/)	2.5 arc-minutes (approximately 5 km at the equator)
Climate—precipitation	Bio12	Annual Precipitation (mm)	WorldClim database version 2.1 (https://worldclim.org/)	2.5 arc-minutes (approximately 5 km at the equator)
Climate—precipitation	Bio13	Precipitation of Wettest Month (mm)	WorldClim database version 2.1 (https://worldclim.org/)	2.5 arc-minutes (approximately 5 km at the equator)
Climate—precipitation	Bio14	Precipitation of Driest Month (mm)	WorldClim database version 2.1 (https://worldclim.org/)	2.5 arc-minutes (approximately 5 km at the equator)
Climate—precipitation	Bio15	Precipitation Seasonality (Coefficient of Variation)	WorldClim database version 2.1 (https://worldclim.org/)	2.5 arc-minutes (approximately 5 km at the equator)
Climate—precipitation	Bio16	Precipitation of Wettest Quarter (mm)	WorldClim database version 2.1 (https://worldclim.org/)	2.5 arc-minutes (approximately 5 km at the equator)
Climate—precipitation	Bio17	Precipitation of Driest Quarter (mm)	WorldClim database version 2.1 (https://worldclim.org/)	2.5 arc-minutes (approximately 5 km at the equator)
Climate—precipitation	Bio18	Precipitation of Warmest Quarter (mm)	WorldClim database version 2.1 (https://worldclim.org/)	2.5 arc-minutes (approximately 5 km at the equator)
Climate—precipitation	Bio19	Precipitation of Coldest Quarter (mm)	WorldClim database version 2.1 (https://worldclim.org/)	2.5 arc-minutes (approximately 5 km at the equator)
Topography	Elevation	Elevation (m)	WorldClim database version 2.1 (https://worldclim.org/)	2.5 arc-minutes (approximately 5 km at the equator)
Topography	Aspect	Terrain aspect (degrees)	Derived from elevation data	2.5 arc-minutes (approximately 5 km at the equator)
Topography	Slope	Terrain slope (degrees)	Derived from elevation data	2.5 arc-minutes (approximately 5 km at the equator)

**Category**	**Indicator**	**Description**	**Data source**	**Resolution used in the model**
Land cover	Land cover	Global Land Cover by National Mapping Organizations	Resource and Environmental Science and Data Center of Global Map (https://globalmaps.github.io/)	2.5 arc-minutes (approximately 5 km at the equator)
Demographic	Population count	Estimated ambient population count (persons per grid cell)	LandScan Global 2010, Oak Ridge National Laboratory (https://landscan.ornl.gov/)	2.5 arc-minutes (approximately 5 km at the equator)

Supplementary Table 4. Legend for Land Cover type

Code	Class Name
1	Broadleaf Evergreen Forest
2	Broadleaf Deciduous Forest
3	Needleleaf Evergreen Forest
4	Needleleaf Deciduous Forest
5	Mixed Forest
6	Tree Open
7	Shrub
8	Herbaceous
9	Herbaceous with Sparse Tree/Shrub
10	Sparse vegetation
11	Cropland
12	Paddy field
13	Cropland/Other Vegetation Mosaic
14	Mangrove
15	Wetland
16	Bare area, consolidated (gravel, rock)
17	Bare area, unconsolidated (sand)
18	Urban
19	Snow/Ice
20	Water bodies
